# Ferroptosis is a targetable detrimental factor in metabolic dysfunction-associated steatotic liver disease

**DOI:** 10.1038/s41418-024-01348-9

**Published:** 2024-07-26

**Authors:** Cédric Peleman, Stig Hellemans, Geraldine Veeckmans, Wout Arras, Hao Zheng, Ine Koeken, Emily Van San, Behrouz Hassannia, Magali Walravens, Edissa Kayirangwa, Nateneal Tamerat Beyene, Mikhaïl Alfons Van Herck, Winnok Harald De Vos, Isabel Pintelon, Luc van Nassauw, Baptiste Oosterlinck, Annemieke Smet, Lieve Vits, Eveline Dirinck, An Verrijken, Joris De Man, Annelies Van Eyck, Wilhelmus Josephus Kwanten, Luisa Vonghia, Ann Driessen, Koen Augustyns, Shinya Toyokuni, Benedicte De Winter, Christophe Van Steenkiste, Sven Francque, Tom Vanden Berghe

**Affiliations:** 1 Laboratory of Experimental Medicine and Pediatrics, Infla-Med Centre of Excellence, University of Antwerp, Antwerp, Belgium; 2 Department of Gastroenterology and Hepatology, Antwerp University Hospital, Antwerp, Belgium; 3 Cell Death Signaling Lab, Department of Biomedical Sciences, University of Antwerp, Antwerp, Belgium; 4 Department of Pathology and Biological Responses, Nagoya University Graduate School of Medicine, Nagoya, Japan; 5 VIB-UGent Center for Inflammation Research, Ghent, Belgium; 6 Department of Biomedical Molecular Biology, Ghent University, Ghent, Belgium; 7 Laboratory of Cell Biology & Histology, Department of Veterinary Sciences, University of Antwerp, Antwerp, Belgium; 8 Antwerp Centre for Advanced Microscopy (ACAM), University of Antwerp, Antwerp, Belgium; 9 µNEURO Research Excellence Consortium on Multimodal Neuromics, University of Antwerp, Antwerp, Belgium; 10 Department of ASTARC, Faculty of Medicine and Health Sciences, University of Antwerp, Antwerp, Belgium; 11 Department of Endocrinology, Diabetology and Metabolism, Antwerp University Hospital, Edegem, Belgium; 12 Department of Pediatrics, Antwerp University Hospital, Edegem, Belgium; 13 Department of Pathology, Antwerp University Hospital, Antwerp, Belgium; 14 Department of Molecular Imaging, Pathology, Radiotherapy, Oncology, Faculty of Medicine and Health Sciences, University of Antwerp, Antwerp, Belgium; 15 Laboratory of Medicinal Chemistry, University of Antwerp, Antwerp, Belgium; 16 Center for Low-temperature Plasma Sciences, Nagoya University, Nagoya, Japan

**Keywords:** Cell biology, Gastrointestinal diseases, Metals

## Abstract

There is an unmet clinical need for pharmacologic treatment for metabolic dysfunction-associated steatotic liver disease (MASLD). Hepatocyte cell death is a hallmark of this highly prevalent chronic liver disease, but the dominant type of cell death remains uncertain. Here we report that ferroptosis, an iron-catalyzed mode of regulated cell death, contributes to MASLD. Unsupervised clustering in a cohort of biopsy-proven MASLD patients revealed a subgroup with hepatic ferroptosis signature and lower glutathione peroxidase 4 (GPX4) levels. Likewise, a subgroup with reduced ferroptosis defenses was discerned in public transcriptomics datasets. Four weeks of choline-deficient L-amino acid-defined high-fat diet (CDAHFD) induced MASLD with ferroptosis in mice. *Gpx4* overexpression did not affect steatohepatitis, instead CDAHFD protected from morbidity due to hepatocyte-specific *Gpx4* knockout. The ferroptosis inhibitor UAMC-3203 attenuated steatosis and alanine aminotransferase in CDAHFD and a second model, i.e., the high-fat high-fructose diet (HFHFD). The effect of monounsaturated and saturated fatty acids supplementation on ferroptosis susceptibility was assessed in human HepG2 cells. Fat-laden HepG2 showed a drop in ferroptosis defenses, increased phosphatidylglycerol with two polyunsaturated fatty acid (PUFA) lipid tails, and sustained ferroptosis sensitivity. In conclusion, this study identified hepatic ferroptosis as a detrimental factor in MASLD patients. Unexpectedly, non-PUFA supplementation to hepatocytes altered lipid bilayer composition to maintain ferroptosis sensitivity. Based on findings in in vivo models, ferroptosis inhibition represents a promising therapeutic target in MASLD.

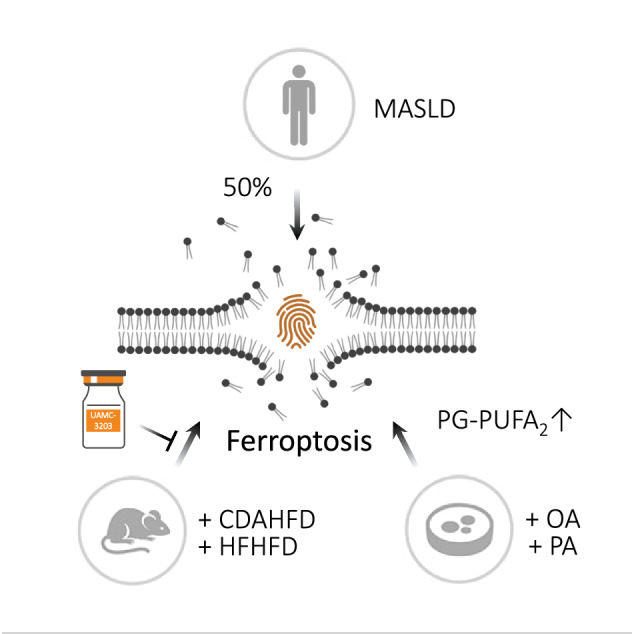

## Introduction

Metabolic dysfunction-associated steatotic liver disease (MASLD) is defined by the presence of steatosis in more than 5% of hepatocytes. Most patients suffer from isolated steatosis (MASL), but a minority displays metabolic dysfunction-associated steatohepatitis (MASH) wherein steatosis is accompanied by ballooning and lobular inflammation. MASH predisposes to liver fibrosis and cirrhosis with its complications [1]. It is estimated that 30% of the global adult population has MASLD which is the most rapidly rising indication for liver transplantation in the USA [2, 3]. This chronic liver disease is driven by hepatic and extrahepatic mechanisms such as adipose tissue insulin resistance and in turn the liver promotes systemic complications [4]. Despite major recent breakthroughs in clinical trials, there is a lack of approved pharmacological therapies for MASLD next to resmetirom [5].

Cell death is a hallmark of MASH, but the nature of hepatocellular demise remains unclear. Apoptosis, pyroptosis and necroptosis have been studied in MASLD, but pan-caspase inhibitor emricasan proved unsuccessful in patients [6–8]. To date, evidence is accumulating for a regulated necrotic cell death in MASLD executed by lethal accumulation of iron-catalyzed lipid peroxides, called ferroptosis [9]. Lipid peroxidation constitutes peroxidation of polyunsaturated fatty acids (PUFA) incorporated into membrane phospholipids and is hypothesized to drive MASLD progression [10]. The discovery of ferroptosis led to novel insights in pathway regulation and new therapeutic options. Indeed, ferroptosis can be prevented using iron chelators and lipophilic radical trapping antioxidants, such as vitamin E, ferrostatin-1 and liproxstatin-1 [11, 12]. We previously developed a third-generation ferrostatin analog, UAMC-3203, with superior pharmacokinetic properties which has already been used in in vivo models of multiple sclerosis, atherosclerosis and multiple organ dysfunction syndrome [13–16].

Ferroptosis ensues when the physiological ferroptosis defense glutathione peroxidase 4 (GPX4) (which employs reduced glutathione) is overridden, resulting in the release of lipid peroxidation breakdown products such as malondialdehyde (MDA) and 4-hydroxynonenal (4HNE). Additional ferroptosis defenses have been described, including ferroptosis suppressor protein 1 (FSP1) and GTP cyclohydrolase 1 (GCH1) which produce membrane-bound reduced ubiquinol/vitamin K and tetrahydrobiopterin (BH4), respectively, that halt lipid peroxidation [17]. The role of these ferroptosis defenses remains unexplored in MASLD. Moreover, it is unclear why lipid-laden hepatocytes would be sensitive to ferroptosis. Indeed, supplementation with non-PUFA, which are the most abundantly increased fatty acids in plasma of MASLD patients, generally induces ferroptosis resistance in vitro [18, 19]. Hence, the effect of lipid accumulation in hepatocytes with regard to ferroptosis sensitivity needs to be further explored.

In this study, we assessed the presence of hepatic ferroptosis in human MASLD and explored its therapeutic potential in two different murine MASLD models through administration of ferroptosis inhibitor UAMC-3203. Moreover, we explored the role of ferroptosis defense GPX4 in MASLD pathogenesis by overexpression and hepatocyte-specific knockout of this protein. Lastly, we evaluated the ferroptosis-sensitizing effect of different fatty acid species on an in vitro hepatocyte model.

## Materials and methods

To ease the presentation of results, some aspects of methodology and rationale thereof are described along with the results. Additional methods are provided in the Supplementary Materials.

### Patient cohort

Serum total cytokeratin-18 (CK-18) fragments (reflecting total epithelial cell death) and caspase-cleaved CK-18 fragments (reflecting apoptosis) were measured in a prospectively collected cohort. We included 76 patients who visited the outpatient hepatology clinic of the Antwerp University Hospital (a tertiary referral center) in 2014–2020 for elevated liver tests and/or obesity (BMI ≥ 30 kg/m^2^) necessitating liver biopsy. Fifty-seven patients displayed biopsy-proven MASLD with different stages of disease severity, while 19 cases had normal liver histology on first evaluation or on repeat biopsy after laparoscopic gastric bypass (controls). MASLD patients with cirrhosis were excluded. Other etiologies of liver disease, including alcohol-induced or drug-induced liver disease, viral or auto-immune hepatitis, metabolic and cholestatic liver diseases, were excluded using specific clinical, biochemical, histological and/or radiographic criteria. No history of alcohol abuse with average daily consumption of ≤20 g. From the same cohort, needle liver biopsies from 27 patients were chosen at random and interrogated for ferroptosis markers. Human data were obtained from patients who gave written consent for collection of material; protocols were conform to ethical guidelines of the Declarations of Helsinki and Istanbul. This study was approved by the Ethical Committee of Antwerp University Hospital (references 6/25/125 and 15/21/227).

### Laboratory mice

All animal experiments presented were approved by the Ethical Committee of Animal Experimentation of University of Antwerp (Protocol Numbers: 2019-42, 2020-25, 2022-57, 2023-61) and Animals Ethics Committee of Ghent University (Protocol Number: 2022-032). Animals received humane care in accordance with “Guide for the Care and Use of Laboratory Animals (Eighth Edition)” published by National Institutes of Health. The ARRIVE guideline for animal pre-clinical studies was used during manuscript preparation [20]. Mice were housed (and bred) under conventional or specific pathogen-free conditions (for GPX4 overexpression) in temperature-controlled (21 °C) animal facilities in 12 h light-dark cycles and provided cage enrichment, water, and fed ad libitum. Male C57Bl/6J mice were purchased from Janvier Labs (Le Genest-Saint-Isle, France) and acclimatized for 1 week. Mice with constitutional whole-body GPX4 overexpression (*Gpx4*^*Tg/+*^), due to hemizygous presence of human GPX4 alleles, were kindly provided by Prof. Dr. Q. Ran [21]. An inducible hepatocyte-specific *Gpx4* deficient mouse line (*Gpx4*^*fl/fl*^ *AlbCreERT2*^*Tg/+*^) was obtained by crossing *Gpx4*^*fl/fl*^ mice, provided by Prof. Dr. M. Conrad [22], with *AlbCreERT2*^*Tg/+*^ mice received from Prof. Dr. D. Metzger [23]. Genetically modified mice were compared to wild-type littermates. Animals were included in experiments at 8 weeks of age with randomization into different experimental groups without blinding. Sacrifice occurred through exsanguination by cardiac puncture under isoflurane. Sample sizes for animal experiment were based on data for mean and effect size from pilot studies to ensure adequate statistical power.

### Statistical analysis

After testing for normality continuous variables were presented as mean and standard deviation or median and interquartile range (IQR); percentages and ratios were used for categorical variables. 95% confidence intervals are mentioned where possible. Means of two groups were assessed with Mann–Whitney *U* test, while Kruskal–Wallis test compared means of three or more groups of interest. Benjamini–Hochberg correction for multiple hypothesis testing when appropriate. Two-way ANOVA was used to assess the effect of ferroptosis modulation in vivo with diet and intervention (genotype or pharmacological treatment) as first and secondary factors. In case of significant interaction, one-way ANOVA was performed with Tukey’s multiple comparisons test. Two-way analysis of covariance (ANCOVA) was used for ferroptosis inhibition in the second MASLD model with adjustment for weight reduction. Kaplan–Meier curves plotted survival data which were compared with log-rank test. Longitudinal bodyweight data were analyzed using generalized estimating equations with correlation structure chosen based on quasi information criterion and correlation information criterion with “geepack” in Rstudio. Statistical analyses in GraphPad Prism 10 (GraphPad Software, CA, USA) and R version 4.1.1 expanded with packages “dplyr” and “car” [24]. Figures generated in GraphPad Prism 10 and RStudio.

## Results

### A subgroup of patients with MASL and MASH displays a signature of hepatic ferroptosis

Based on the hypothesis that ferroptosis is present in MASLD, we investigated whether a signature of this necrotic cell death could be detected in serum of a cohort of biopsy-proven MASLD patients and controls (*n* = 76). MASLD patients had a higher proportion of males with higher HOMA-IR, ALT, AST, and lower HDL cholesterol, compared to controls. Total serum CK-18 fragments and caspase-cleaved CK-18 were increased in MASLD patients compared to controls (Table S1). Total CK-18 levels were increased in 15 (28.3%) MASLD patients, all of whom also displayed increased caspase-cleaved CK-18 (except for one patient) (Fig. S1A). Hence, in MASLD patients displaying evidence of hepatocyte cell death based on serum CK-18, there was a proportion showing cleaved CK-18, which might indicate occurrence of apoptosis.

**Fig. 1:**
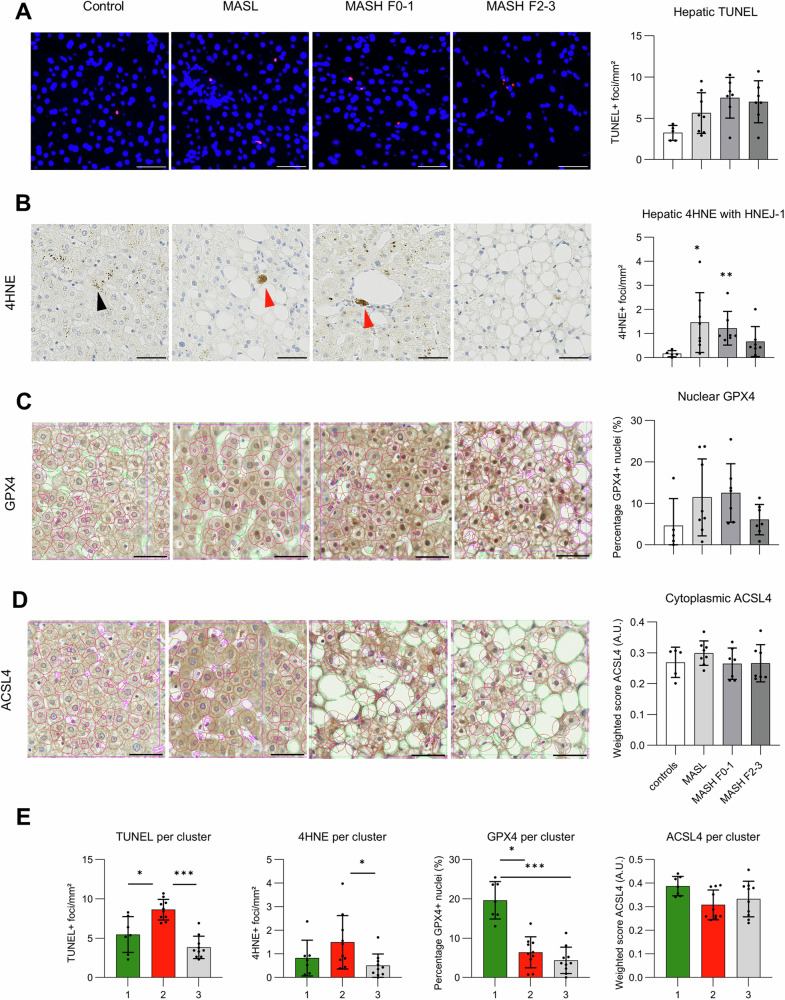
Signature of hepatic ferroptosis in subset of MASLD patients. Ferroptosis markers in liver biopsies from controls (*n* = 5), MASLD patients with isolated steatosis (MASL, *n* = 8), metabolic dysfunction-associated steatohepatitis without (MASH F0-1, *n* = 7) or with significant fibrosis (MASH F2-3, *n* = 7). **A** Terminal deoxynucleotidyl transferase dUTP nick end labeling (TUNEL) stain (red) with DAPI (blue). **B** Immunohistochemistry (IHC) for 4-hydroxynonenal (4HNE). Red arrows indicate aggregates of ferroptosis breakdown products. **C**, **D** IHC glutathione peroxidase 4 (GPX4) and acyl-CoA synthetase long-chain family member 4 (ACSL4). Contours of nuclei, cytoplasm and lipid vacuoles annotated. Magnification 400×, scale bar 50 µm. **E** TUNEL, 4HNE, GPX4 and ACSL4 in clusters 1, 2 and 3 as determined by k-prototypes partitioning clustering. Mean ± standard deviation. *p < 0.05; **p < 0.01; ***p < 0.001; Kruskal–Wallis with Dunn’s test.

Because serum MDA (ferroptosis breakdown product) was similar among controls and MASLD (Fig. S1B), we investigated whether a signature of ferroptosis could be discerned in liver biopsies from a subset of this cohort (*n* = 27). An increased number of terminal deoxynucleotidyl transferase dUTP nick end labeling (TUNEL) positive foci (general indicator of cell death) was present in several MASLD patients, both isolated steatosis (MASL) and MASH with or without significant fibrosis (Fig. 1A; Fig. S2). Several MASL and MASH F0-1 patients displayed intense 4HNE positive aggregates between hepatocytes, sometimes adjacent to inflammatory infiltrates, which were absent in controls. These foci of ferroptosis breakdown products are different from granular 4HNE positive lipofuscin in hepatocyte cytoplasm observed in some patients (Fig. 1B; Fig. S3). Next, immunohistochemistry (IHC) was performed for ferroptosis defense GPX4 and acyl-CoA synthetase long-chain family member 4 (ACSL4) which can promote ferroptosis by enabling PUFA esterification into the cellular membrane. To quantify nuclear and cytoplasmic presence of these two epitopes, we reconstructed around 10,000 hepatocytes per patient’s needle liver biopsy with machine learning (see Supplementary Methods). An increased proportion of GPX4 positive nuclei was present in several MASLD patients, compared to controls (Fig. 1C; S4A). No significant difference was detected in nuclear or cytoplasmic levels of ACSL4 between different histologic groups (Fig. 1D; Fig. S4B). Staining for non-heme ferrous iron revealed sinusoidal deposits of this catalyst of ferroptosis in some MASLD patients, but not in controls (Fig. S5). Based on the assessment of ferroptosis-related markers, unsupervised clustering defined three clusters (Fig. S6). Cluster “1” (green) consists of MASLD patients without cell death nor ferroptosis, evidenced by low TUNEL positivity and 4HNE deposits and absence of ferrous iron, accompanied by high nuclear GPX4. The ferroptosis defense GPX4 is lowered in cluster “2” (red) which displays the highest levels of cell death, 4HNE deposits and ferrous iron. Cluster “3” (gray) comprises controls and some MASLD patients who display low levels of all markers. ACSL4 expression is not different among the clusters (Fig. 1E). Hence, cluster “2” bears a signature of hepatic ferroptosis and trend toward lower HDL cholesterol, higher ALT and total CK-18 levels. Cluster “2“ comprises 40–60% of patients with MASL and MASH with and without significant fibrosis (Table 1). This implies that up to 50% of MASLD patients in this cohort displays a “ferroptosis signature”.

**Table 1:** Characteristics of three clusters of MASLD and controls derived from unsupervised clustering based on hepatic markers of ferroptosis.

Characteristics	Cluster 1	Cluster 2	Cluster 3	*p* value	Adjusted *p* value
	(*n* = 7)	(*n* = 10)	(*n* = 10)		
Liver histology					
Controls, *n* (%)	1 (20)	0 (0)	4 (80)	0.1459	
MASL, *n* (%)	3 (37.5)	3 (37.5)	2 (25)		
MASH F0-1, *n* (%)	3 (42.8)	3 (42.8)	1 (14.4)		
MASH F2-3, *n* (%)	0 (0)	4 (57.2)	3 (42.8)		
Positive ferrous iron stain, *n* (%)	1 (14.3)	7 (70)	1 (10)	0.014	
Age, year	49 (39.8–58.5)	53 (48.0–58.0)	46 (36–56.5)	0.516	0.695
Gender, male, *n* (%)	3 (42.9)	8 (80)	5 (50)	0.298	0.596
BMI, kg/m^2^	29.0 (27.0–33.0)	29.6 (29.4–31.6)	38.0 (35.3–44.5)	0.546	0.695
Type 2 diabetes mellitus, %	4 (57.1)	5 (50)	8 (80)	0.471	0.695
HOMA-IR	4.6 (3.2–7.3)	5.9 (4.1–9.8)	3.7 (2.9–5.7)	0.39	0.683
Total cholesterol, mg/dL	202 (179.3–219.8)	141 (137.0–207.0)	189 (171.5–208.0)	0.679	0.76
LDL cholesterol, mg/dL	105 (92.9–130.0)	99 (90.0–169.0)	114 (101.5–151.0)	0.706	0.76
HDL cholesterol, mg/dL	50 (43.5–60.8)	33 (29.5–38.0)	50 (44.0–63.5)	0.022*	0.308
Ferritin, µg/L	155 (103.5–205.3)	236 (122.0–262.08)	113 (66.0–407.5)	0.76	0.76
Perls stain, positive, *n* (%)	2 (28.6)	0 (0)	1 (10)	0.238	0.555
AST, U/L	21 (17.3–29.5)	37 (34.0–49.0)	42 (20.0–49.5)	0.115	0.403
ALT, U/L	35 (31.0–47.5)	67 (57.0–106.0)	48 (32.0–98.5)	0.099	0.403
Cytokeratin-18 M65, U/L	246.4 (187.1–819.8)	371.8 (257.9–469.6)	178.3 (115.3–364.2)	0.162	0.454
Cytokeratin-18 M30, U/L	286.3 (161.4–379.9)	341.1 (218.0–546.9)	184.8 (136.3–255.8)	0.1	0.403

Data presented as median (interquartile range) unless otherwise specified. Kruskal–Wallis and Fisher’s exact tests to compare patient characteristics among different histological groups.

*BMI* body mass index, *HOMA-IR* homeostasis model assessment–insulin resistance, *LDL* low-density lipoprotein, *HDL* high-density lipoprotein, *AST* aspartate aminotransferase, *ALT* alanine aminotransferase.

*P  <  0.05.

### Publicly available transcriptomics datasets confirm decreased ferroptosis defenses in MASLD patients from all histologic severity grades

**Fig. 2:**
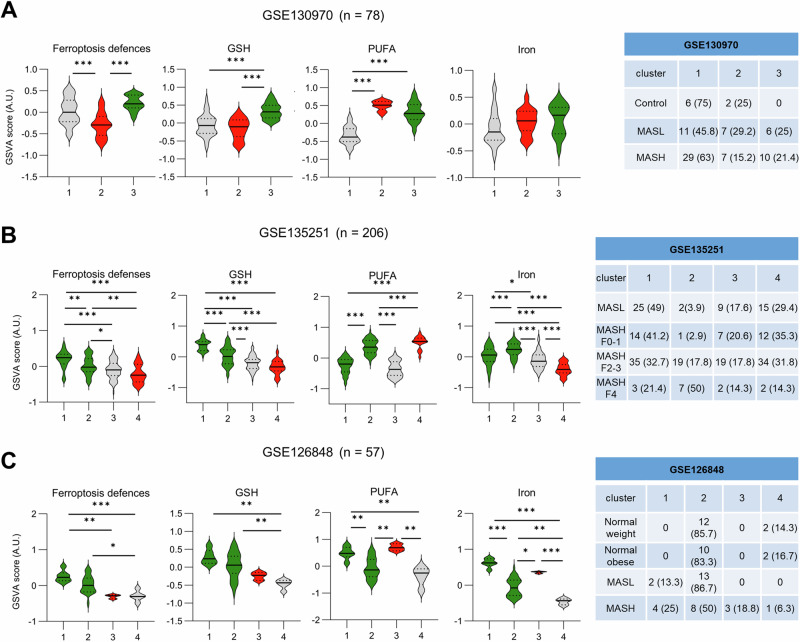
Public transcriptomics datasets reveal subset of MASLD patients with hepatic ferroptosis susceptibility signature. Hepatic expression of ferroptosis-related genes studied in three cohorts of MASLD patients and controls. Genes were grouped in gene sets for which each patient received a gene set variation analysis (GSVA) score. Higher GSVA scores for “ferroptosis defenses” and “GSH” indicate upregulated ferroptosis defenses and increased glutathione (GSH) synthesis, respectively. Higher “PUFA” and “Iron” scores indicate drive toward more PUFA in phospholipids for ferroptosis. **A** GSVA scores for patients in three clusters determined by Gaussian Mixture modeling in GSE130970 (*n* = 78). Table displaying distribution of histologic groups across different clusters. **B**, **C** Patients from GSE135251 (*n* = 206) and GSE126848 (*n* = 57) received GSVA scores for four gene sets and cluster membership. Red clusters represent ferroptosis-sensitizing PUFA incorporation together with lowered ferroptosis defenses; green clusters exhibit highly expressed ferroptosis defenses. Violin plots show median and quartiles. *p < 0.05; **p < 0.01; ***p < 0.001; Kruskal–Wallis with Dunn’s test.

To explore the validity of this ferroptosis signature in liver biopsy specimens in larger patient cohorts, we accessed three public transcriptomics datasets to investigate the expression of 30 ferroptosis-related genes. The latter were grouped into following gene sets to facilitate interpretation: “ferroptosis defenses” (including *GPX4*, *FSP1* and *GCH1*), “GSH” (production of glutathione employed by GPX4), “PUFA” (increased incorporation of PUFA into membranes promoting ferroptosis), and “Iron” (increased ferrous iron to catalyze ferroptosis) (Table S2). By means of gene set variation analysis (GSVA) each patient received a score for each of the four sets, wherein higher GSVA scores indicate higher expression of all genes in the set. In dataset GSE130970 (*n* = 78) there was no difference in GSVA scores among the histologic groups, but three latent subgroups were found based on these scores by Gaussian Mixture modeling (Figs. S7 and S8A–D). The first cluster (gray) included controls and MASLD patients with low PUFA incorporation and moderate ferroptosis defenses. The third cluster (green) displayed gene expression compatible with more PUFA incorporation into membranes but also upregulation of ferroptosis defenses, while the second cluster (red) comprises MASLD patients with ferroptosis-sensitizing PUFA incorporation combined with lowered ferroptosis defenses (Fig. 2A). Likewise, in datasets GSE135251 (*n* = 206) and GSE126848 (*n* = 57) unsupervised machine learning discerned a “red” cluster with increased gene expression for PUFA incorporation, theoretically sensitizing toward ferroptosis, combined with decreased expression of ferroptosis defenses. This subgroup with ferroptosis susceptibility signature differs from gray and green clusters with no sensitization toward ferroptosis or increased expression of ferroptosis defenses (Fig. 2B, C; Figs. S9, S10A–D, S11, S12A–D). Thus, three transcriptomic datasets of 341 MASLD patients reveal a subgroup (up to 30% of patients) with ferroptosis susceptibility signature, highlighting again patient heterogeneity and need for patient stratification.

### Overexpression of Gpx4 has no effect in a MASH mouse model

**Fig. 3:**
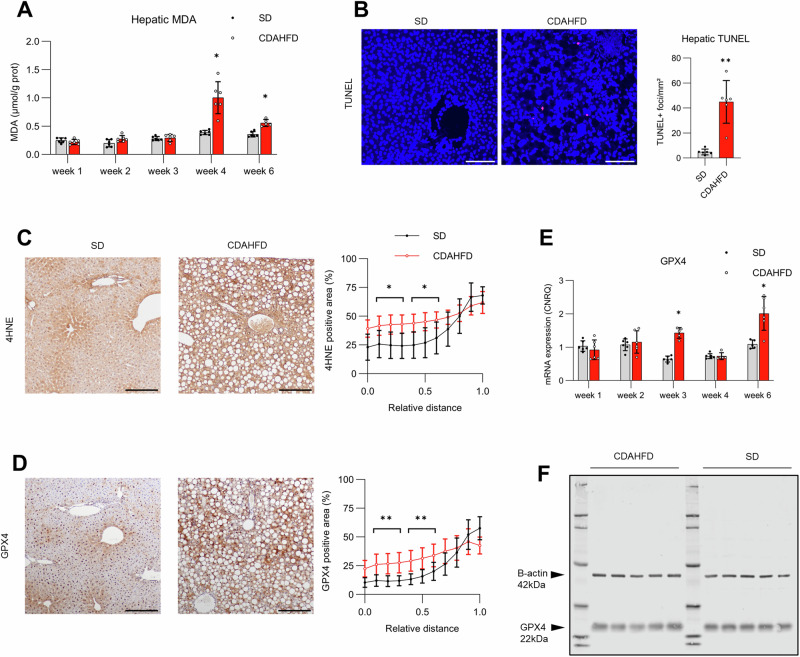
Detection of ferroptosis markers in CDAHFD. Markers of hepatic cell death and ferroptosis were measured in groups of mice fed the standard diet (SD) or choline-deficient L-amino acid-defined high-fat diet (CDAHFD) with an emphasis on the 4-week timepoint (*n* = 6 per group). **A** Hepatic MDA measured in mice on SD or CDAHFD for 1, 2, 3, 4 and 6 weeks. **B** Representative images and quantification of the terminal deoxynucleotidyl transferase dUTP nick end labeling (TUNEL) stain (red), with DAPI counterstain (blue), in mice fed SD or CDAHFD for 4 weeks. **C**, **D** Representative images of immunohistochemistry (IHC) for 4HNE and Gpx4 in mice on SD or CDAHFD for 4 weeks. To quantify the change in distribution of these epitopes, liver lobules were reconstructed based on the Voronoi principle. The proportion of area positive for 4HNE or Gpx4 is plotted with regards to the relative distance (0–1) within the liver lobule, between the portal triads (value 0) to the centrolobular veins (value 1 on *x*-axis). Slides for IHC were pooled from two independent experiments. **E** Hepatic mRNA expression of Gpx4 in mice on SD or CDAHFD on different time points, expressed as calibrated normalized relative quantities (CNRQ). **F** Western blot to visually inspect the protein level of Gpx4 in mice on SD or CDAHFD for 4 weeks. Results are presented as mean ± standard deviation. Mann–Whitney *U* test with correction for multiple hypothesis testing; *p < 0.05; **p < 0.01. Magnification 100×, scale bar represents 200 µm.

To elucidate the role of ferroptosis in MASLD, we aimed to modulate this cell death in a murine MASH model with hepatic ferroptosis. The choline-deficient L-amino acid-defined high-fat diet (CDAHFD) induces histologic features of MASH after 1 week and significant liver fibrosis after 4 weeks, accompanied by increased ALT levels, as published previously [25]. Hepatic MDA was increased in CDAHFD, compared to standard diet (SD), at 4 and 6 weeks. Increased hepatic MDA after 4 weeks CDAHFD was confirmed in a second independent experiment and we focused on this timepoint (Fig. 3A; Fig. S13A). The number of TUNEL-positive foci (a general indicator of cell death) was higher after 4 weeks of CDAHFD; this cell death was not apoptosis given absence of cleaved caspase-3 (Fig. 3B; Fig. S13B). The distribution of 4HNE on IHC was altered as this epitope appeared in all zones of the liver lobule in CDAHFD but remained confined to the pericentral region in SD. This was confirmed by quantification of the 4HNE positive area per relative distance within reconstructed liver lobules in whole slide liver images (see Supplementary Methods; Fig. 3C; Fig. S13C) [26]. Likewise, Gpx4 positivity increased in midzone and periportal regions in CDAHFD compared to SD (Fig. 3D). However, no difference was noted in hepatic mRNA expression or total hepatic protein levels of Gpx4 after 4 weeks on CDAHFD, nor was there any difference in hepatic mRNA expression of *Fsp1* and *Gch1* (Fig. 3E, F; Fig. S13D).

**Fig. 4:**
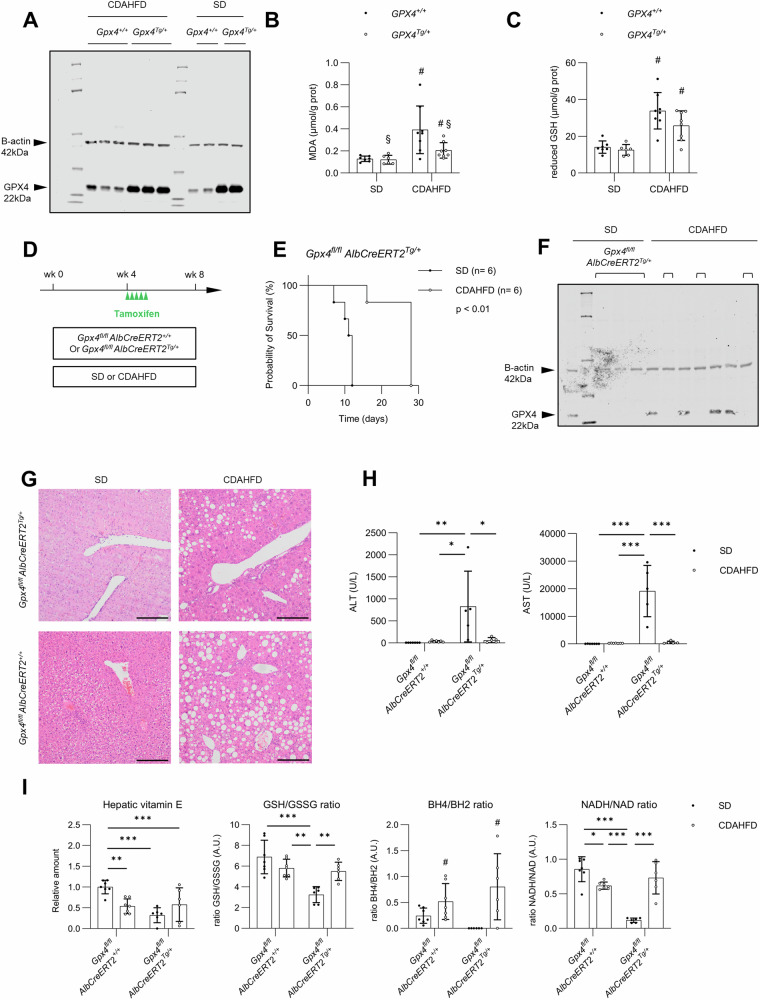
While Gpx4 overexpression has no effect, CDAHFD-induced MASH protects against loss of hepatocyte Gpx4. Role of glutathione peroxidase 4 (Gpx4) in choline-deficient L-amino acid-defined diet (CDAHFD) was explored using transgenic mice with whole-body GPX4 overexpression (*Gpx4*^*Tg/*+^) or tamoxifen-inducible hepatocyte-specific *Gpx4* knockout (*Gpx4*^*fl/fl*^ *AlbCreERT2*^*Tg/+*^). **A** Western blot for hepatic Gpx4 in *Gpx4*^*Tg/*+^ and wild-type littermates (*Gpx4*^*+/+*^) fed CDAHFD or standard diet (SD). **B**, **C** Hepatic MDA and reduced glutathione (GSH) normalized for protein content (*n* = 6 or 8). **D** Experimental design for hepatocyte-specific *Gpx4* knockout (*n* = 5 or 7). **E** Kaplan–Meier curves of *Gpx4*^*fl/fl*^ *AlbCreERT2*^*Tg/+*^ fed SD or CDAHFD; day 0 equals first day of tamoxifen. **F** Western blot of hepatic Gpx4 in *Gpx4*^*fl/fl*^ *AlbCreERT2*^*Tg/+*^ (brackets) and controls (*Gpx4*^*fl/fl*^ *AlbCreERT2*^*+/+*^, without brackets) after tamoxifen. **G** H&E stain of *Gpx4*^*fl/fl*^ *AlbCreERT2*^*Tg/+*^ and controls fed SD or CDAHFD at spontaneous morbidity or experiment termination. Magnification 100×, scale bar 200 µm. **H** Serum alanine aminotransferase (ALT), aspartate aminotransferase (AST). **I** Mass spectrometry for hepatic vitamin E, ratios of reduced-on-oxidized glutathione (GSH/GSSG), tetrahydrobiopterin-on-7,8-dihydro-L-biopterin (BH4/BH2) and oxidized-on-reduced nicotinamide adenine dinucleotide (NADH/NAD). Data from two independent experiments, mean ± standard deviation. Scale bar 200 µm. *p < 0.05; **p < 0.01; ***p < 0.001; ^#^*p* < 0.05 for factor diet; ^§^*p* < 0.05 for factor genotype. Log-rank test, two-way ANOVA with post-hoc test.

Given the increased hepatic MDA after 4 weeks of CDAHFD, we attempted to inhibit ferroptosis through overexpression of physiologic ferroptosis defense Gpx4 with chimeric mice that express both murine and human *Gpx4* genes (*Gpx4*^*Tg/+*^). Western blot confirmed increased hepatic Gpx4 protein bands in *Gpx4*^*Tg/+*^ animals fed CDAHFD or SD for 4 weeks, but not in their control littermates (*Gpx4*^*+/+*^) (Fig. 4A). Hepatic MDA increased in CDAHFD, while *Gpx4*^*Tg/+*^ reduced this biomarker (Fig. 4B). Reduced glutathione (GSH), which is employed by Gpx4, was increased in CDAHFD but unaffected by *Gpx4*^*Tg/+*^ (Fig. 4C). Moreover, Gpx4 overexpression had no effect on serum ALT and AST, hepatomegaly or histologic abnormalities induced by CDAHFD (Fig. S14A–E). Thus, Gpx4 overexpression reduced the hepatic MDA levels without improvement in liver damage or histology.

### CDAHFD protects against conditional hepatocyte-specific Gpx4 deficiency-induced death

As a reverse strategy, we explored the effect of depleting Gpx4 in CDAHFD-induced MASH through tamoxifen-inducible hepatocyte-specific *Gpx4* deficiency. Loss of hepatocyte Gpx4 is incompatible with life [16]. Indeed, *Gpx4*^*fl/fl*^ *AlbCreERT2*^*Tg/+*^ on SD displayed clinical deterioration necessitating sacrifice by day 7–12 after the first tamoxifen injection. This differed from *Gpx4*^*fl/fl*^ *AlbCreERT2*^*Tg/+*^ mice fed CDAHFD for 4 weeks prior to tamoxifen, which did not display morbidity until termination of the experiment after 8 weeks except for one mouse. Controls without Cre recombinase (*Gpx4*^*fl/fl*^ *AlbCreERT2*^*+/+*^) displayed no morbidity after tamoxifen (Fig. 4D, E). Liver tissue collected during clinical deterioration or after 8 weeks of diet confirmed reduced hepatic *Gpx4* mRNA and absence of hepatic Gpx4 protein bands in *Gpx4*^*fl/fl*^ *AlbCreERT2*^*Tg/+*^ (Fig. S15A; Fig. 4F). Deficiency of hepatocyte Gpx4 in SD caused widespread hepatocyte necrosis with nuclear fading, with sparing of the periportal region and moderate inflammatory infiltrates. However, in livers of *Gpx4*^*fl/fl*^ *AlbCreERT2*^*Tg/+*^ animals fed CDAHFD, loss of hepatocyte Gpx4 caused no alterations (other than diet-induced MASH) (Fig. 4G). Livers from deteriorating *Gpx4*^*fl/fl*^ *AlbCreERT2*^*Tg/+*^ on SD displayed marked hepatomegaly (Fig. S15B). Moreover, serum ALT and AST in *Gpx4*^*fl/fl*^ *AlbCreERT2*^*Tg/+*^ on SD increased exuberantly (Fig. 4H). In summary, CDAHFD seems to protect against ferroptosis and acute liver injury induced by hepatic Gpx4 loss.

We sought to further explain this observation by investigating other ferroptosis defenses with mass spectrometry. Upon loss of hepatocyte Gpx4 under SD, we observed a decrease in hepatic vitamin E, GSH/GSSG ratios, BH4/BH2 ratios as well as NADH/NAD ratios, possibly due to their oxidation during hepatic ferroptosis (Fig. 4I). Hepatic levels of ferroptosis defenses ubiquinol and vitamin K were undetectable but mRNA expression of FSP1 dropped upon Gpx4 depletion under SD (Fig. S15C). The drop in anti-ferroptotic defense mechanisms is restored in *Gpx4*^*fl/fl*^ *AlbCreERT2*^*Tg/+*^ on CDAHFD (Fig. 4I; Fig. S15D). Relative abundances of individual metabolites are displayed in Fig. S15E. Hence, MASH livers under metabolic stress display plasticity to uphold other ferroptosis defenses which prevent spontaneous ferroptosis after the loss of Gpx4.

### Ferroptosis inhibition protects against hepatocyte cell damage and macrovesicular steatosis in two MASLD mouse models

**Fig. 5:**
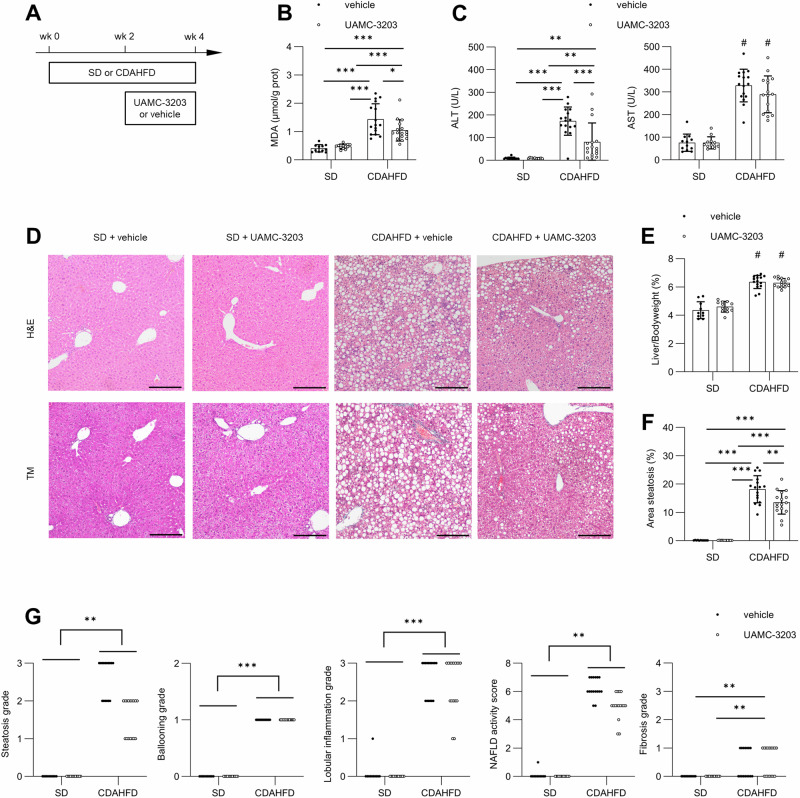
Therapeutic ferroptosis inhibition attenuates hepatocyte damage and steatosis in CDAHFD. **A** Experimental design of UAMC-3203 (12.35 mg/kg bodyweight) or 0.9% NaCl (vehicle) via subcutaneous osmotic minipumps (*n* = 12 or 16) in animals fed CDAHFD or SD for 4 weeks. **B** Hepatic MDA. **C** Serum ALT, AST. **D** H&E and Masson’s trichrome. Magnification 100×, scale bar 200 µm. **E** Liver-on-bodyweight ratio. **F** Quantification of liver area enveloped by macrovesicular steatosis. **G** Scoring of steatosis, ballooning, lobular inflammation, NAFLD activity score and fibrosis. Mean ± standard deviation. Data from two independent experiments combined. *p < 0.05; **p < 0.01; ***p < 0.001; ^#^*p* < 0.05 for factor diet. Two-way ANOVA with post-hoc test. Kruskal–Wallis with post-hoc testing for ordinal histologic scoring.

Given that synthetic lipophilic radical traps are much more potent in protecting against excessive lipid peroxidation than *Gpx4* overexpression, we explored the potential of pharmacologic ferroptosis inhibition with UAMC-3203 in CDAHFD. Pharmacokinetic studies showed excellent distribution of this compound to the liver after bolus administration (Table S3), as well as via osmotic minipumps [15]. First, UAMC-3203 or 0.9% NaCl (vehicle) was administered once daily intraperitoneally (i.p.) for 4 weeks in animals fed CDAHFD or SD (preventive setup) (Fig. S16A). CDAHFD increased hepatic MDA, ALT/AST and liver mass, which was lowered by UAMC-3203 (Fig. S16B–D). However, daily i.p. injection of UAMC-3203 did not affect histologic abnormalities of MASH (Fig. S16E–G). Given the suboptimal half-time of UAMC-3203 in mice (*T*^1/2^ = 3–4 h), UAMC-3203 or vehicle was administered in therapeutic setting via osmotic minipumps in mice fed CDAHFD or SD for 4 weeks (Fig. 5A). Similar to the preventive setting, increased MDA and ALT/AST in CDAHFD was lowered in animals treated with UAMC-3203, compared to vehicle (Fig. 5B, C). Despite the lack of effect on hepatomegaly, therapeutic UAMC-3203 in CDAHFD reduced steatosis, but not lobular inflammation, ballooning or fibrosis (Fig. 5D–G).

**Fig. 6:**
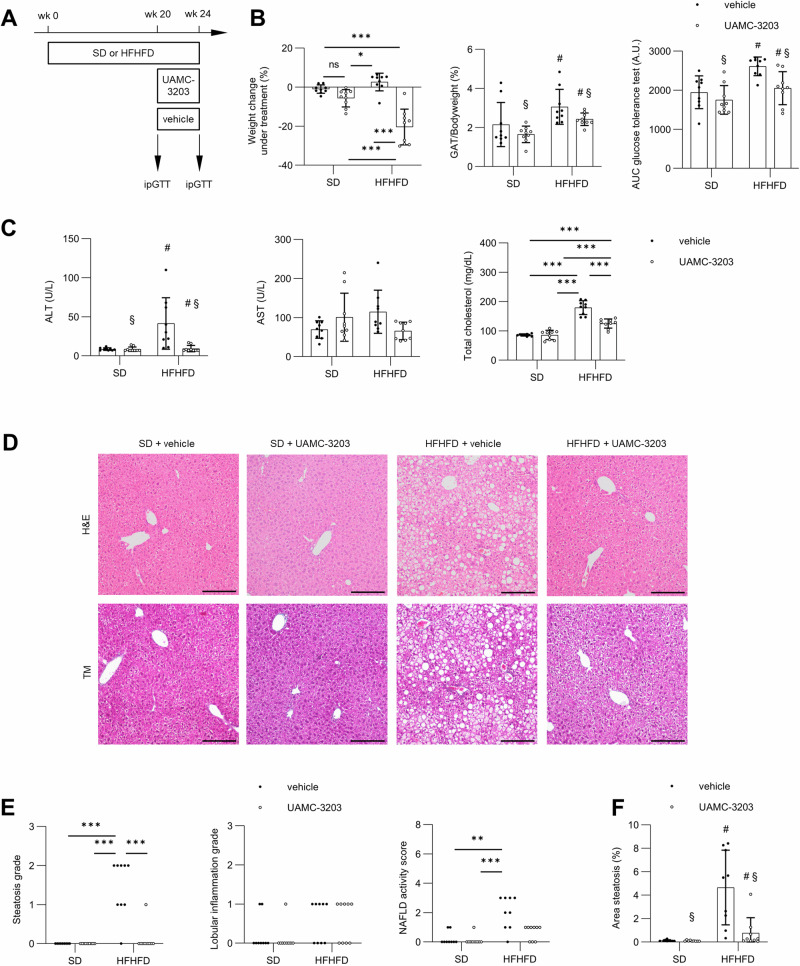
Therapeutic ferroptosis inhibition attenuates steatosis and features of metabolic syndrome in HFHFD. **A** Experimental design of therapeutic administration of UAMC-3203 or 0.9% NaCl (vehicle) via subcutaneous osmotic minipumps in animals fed high-fat high-fructose diet (HFHFD) or SD for 24 weeks. **B** Relative weight change during pharmacologic treatment. Area under curve (AUC) for intraperitoneal glucose tolerance test, adjusted for AUC before pharmacological intervention. Gonadal adipose tissue-on-bodyweight ratio. **C** Serum alanine aminotransferase (ALT), aspartate aminotransferase (AST), total cholesterol. **D** H&E and Masson’s trichrome. Magnification 100×, scale bar 200 µm. **E** Scoring of steatosis, lobular inflammation and NAFLD activity score. **F** Quantification of liver area enveloped by macrovesicular steatosis. Data from two independent experiments combined (*n* = 9). *p < 0.05; **p < 0.01; ***p < 0.001; ^#^*p* < 0.05 for factor diet; ^§^*p* < 0.05 for factor intervention. Analysis of covariance (ANOVA) with adjustment for relative weight change during pharmacologic intervention with post-hoc test if appropriate. Kruskal–Wallis with post-hoc testing for ordinal histologic scoring.

While CDAHFD induces MASH with a high disease activity and fibrosis, so reminiscent of human MASH, mice on this diet lack most features of the metabolic syndrome. Hence, we explored ferroptosis inhibition in a second model, the high-fat high-fructose diet (HFHFD) model, which causes MASH with obesity and decreased glucose tolerance [27]. We detected higher levels of ferroptosis breakdown product MDA, but not 4HNE, in mice fed HFHFD for 20–24 weeks (Fig. S17A, B). UAMC-3203 or vehicle treatment was administered for 4 weeks via sequential osmotic minipumps in mice fed HFHFD or SD for 24 weeks (Fig. 6A). UAMC-3203 induced (around 20%) weight loss in HFHFD compared to vehicle, but not in SD, and attenuated gonadal adipose tissue mass. Post-treatment glucose tolerance was improved in UAMC-3203 treated groups after adjustment for pre-treatment glucose tolerance and weight reduction (Fig. 6B). Ferroptosis inhibition attenuated ALT and total cholesterol, but not AST, in HFHFD independent from the effect on bodyweight on ANCOVA analysis (Fig. 6C). Histologic examination showed marked reduction of steatosis, but not lobular inflammation, ballooning or fibrosis, in HFHFD treated with UAMC-3203 (Fig. 6D–F). Ferroptosis inhibition also affected serum glucose, HDL cholesterol and free fatty acids, but not LDL cholesterol or triglycerides (Fig. S18A–I). In summary, ferroptosis targeting protected against HFHFD-induced liver injury and steatosis, independent from its effect on total bodyweight.

### SFA/MUFA supplementation in HepG2 cells boosts increase in PG-PUFA_2_ levels similar to PUFA supplementation, which might explain sustained ferroptosis susceptibility

**Fig. 7:**
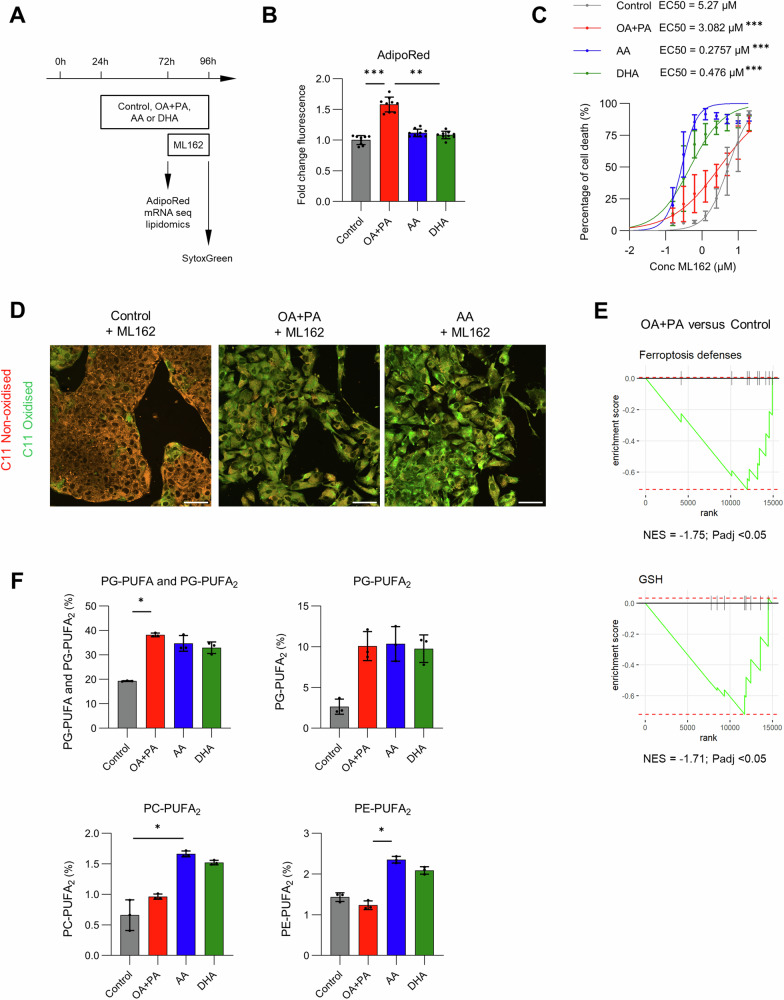
Oleic and palmitic acid supplementation in HepG2 increases PG-PUFA2 leading to ferroptosis susceptibility. To study the effect of lipid accumulation on ferroptosis sensitivity, HepG2 cells were exposed to glucose, insulin, three cytokines and different species of fatty acids, i*.*e., oleic and palmitic acid (OA/PA), arachidonic acid (AA 20:4, ω-6 PUFA) or docosahexaenoic acid (DHA 22:6, ω-3 PUFA). **A** Experimental design of fatty acid incubation and ferroptosis induction with GPX4 inhibitor ML162. **B** Neutral lipids measured with AdipoRed. **C** Percentage of cell death (with SytoxGreen) at increasing ML162 concentrations. After fitting non-linear regressions per condition, best-fit half maximal effective concentration (EC50) values were compared using Aikake Information Coefficient and one-way ANOVA with post-hoc testing. **D** Representative images of C11-BODIPY (581/591) dye in HepG2. Oxidized dye (green) indicates lipid radical oxygen species 2 h after ML162, as opposed to the normal reduced form (red). **E** Gene set enrichment analysis (GSEA) plots for gene sets “ferroptosis defenses” and “GSH” in HepG2 treated with OA/PA compared to solvent control (Control), with normalized enrichment score (NES) (*n* = 6). **F** Percentage of phosphatidylglycerol (PG) esterified with one or two polyunsaturated fatty acids, i.e., PG-PUFA and PG-PUFA_2_. Percentage of phosphatidylcholine and phosphatidylethanolamine with two PUFA, i.e., PC-PUFA_2_ and PE-PUFA_2_, respectively (*n* = 3). Data pooled from three independent experiments, performed in triplicate, except for C11-BODIPY (581/591). *p < 0.05; **p < 0.01; ***p < 0.001. One-way ANOVA with post-hoc test.

We sought to explain the increased propensity toward ferroptosis in MASH. Supplementation with monounsaturated fatty acids (MUFA) is known to reduce ferroptosis sensitivity, while PUFA promote this cell death [19, 28]. Hence, we investigated ferroptosis sensitivity of hepatocytes exposed to MASH environment (high glucose, insulin, tumor necrosis factor-α, interleukin-1β and transforming growth factor-β) with different species of fatty acids, i.e., oleic acid (OA 18:1, MUFA) and palmitic acid (PA 16:0, SFA), arachidonic acid (AA 20:4, ω-6 PUFA) or docosahexaenoic acid (DHA 22:6, ω-3 PUFA). Forty-eight hours exposure to MASH environment with OA/PA, but not AA or DHA, induced intracellular lipid droplet accumulation in HepG2 cells (Fig. 7A, B). AA and DHA both sensitized toward ferroptosis induced by GPX4 inhibitor ML162 (Fig. 7C). Unexpectedly, OA/PA-supplemented HepG2 cells did not show reduced ferroptosis sensitivity, instead cell death sensitivity (Fig. 7C; Fig. S19) and lipid ROS were slightly increased (Fig. 7D).

Transcriptomics and untargeted lipidomics were performed to unravel the mode-of-action of OA/PA supplementation on ferroptosis sensitivity. Most ferroptosis-related genes were lowered in OA/PA-supplemented HepG2 (Fig. S20A–D; Table S4), which was further underscored by negative normalized enrichment scores on gene set enrichment analysis (GSEA) using genes set of “ferroptosis defenses” or “GSH” (Fig. 7E; Fig. S21). Lipidomics on OA/PA-supplemented HepG2 indicated no major changes in SFA, MUFA and PUFA, while AA- or DHA-supplemented HepG2 showed strong increase in PUFA-containing lipids (Fig. S22). Instead, a strong increase in triacylglycerol (TAG) was prominent in OA/PA-supplemented HepG2 (Figs. S23–S25), which is in line with the observed increase in lipid droplets (Fig. 7B). Since PUFA-containing phospholipids (PL-PUFA) constitute the “fuel” for ferroptosis, analysis was directed to these types of lipids. Although low in abundance, ether phospholipids (PL-ePUFA) [29] or PLs with two PUFA-containing lipid tails (PL-PUFA_2_) were recently suggested to control ferroptosis sensitivity [30, 31]. In line, we found elevated levels of PC-ePUFA, PE-ePUFA as well as PE-PUFA_2_, PC-PUFA_2_ and PG-PUFA_2_ in HepG2 supplemented with AA or DHA (Fig. 7F; Fig. S26A, B). Interestingly, PG-PUFA_2_ was also increased in OA/PA-supplemented HepG2, although no PUFA were supplied in this condition (Fig. 7F). This finding suggests that SFA/MUFA supplementation can lead to changes related to PL-PUFA_2_, which affect ferroptosis sensitivity.

## Discussion

Several types of cell death are present in human MASLD livers, including apoptosis, necroptosis and pyroptosis, but their relative importance is difficult to study [6, 7]. Lipid peroxidation is a known detrimental process in MASLD and has sparked interest as it triggers ferroptosis with new therapeutic options.

Assessment of human liver biopsies and public datasets with unsupervised clustering showed a subgroup of MASLD patients with clear ferroptosis signature. The presence of hepatic ferroptosis in up to 50% of MASLD cases was not linked to histologic steatohepatitis severity grade, as ferroptosis was not only found in MASH patients but also in patients with isolated steatosis. In contrast, previous studies report cell death (by TUNEL-positive foci) and apoptosis in MASH livers but less so in MASL [6, 32]. Likewise, 4HNE positivity on IHC in MASLD was found to correlate with disease activity in some studies [33–36]. An explanation for our findings could be use of the HNEJ-1 primary antibody in our study with higher specificity for ferroptosis detection in tissues [37]. Also the dynamic nature of steatohepatitis disease activity is most likely underestimated in MASLD, as liver biopsy only provides a snapshot assessment [38]. In addition, semi-quantitative scoring for ballooning may lack sensitivity [39]. Our study showed that large cohorts and advanced research tools can detect patients with different disease drivers despite histologically similar disease.

This patient heterogeneity must be considered when translating results from pre-clinical studies to clinical trials. For instance, the pan-caspase inhibitor emricasan failed to reach its primary endpoint in the phase II randomized clinical trial [8]. This could indicate that caspase-mediated apoptosis and pyroptosis are not primordial in MASLD or point toward inadequate patient stratification. Also, given that ferroptosis is not a common feature of all patients, treatment with ferroptosis inhibitors in MASLD patients without hepatic ferroptosis might lead to negative trial results.

Ferroptosis inhibition in mice had a direct effect on the liver but also improved upstream metabolic drivers of the liver disease. In CDAHFD and HFHFD, therapeutic administration of UAMC-3203 attenuated ALT and steatosis but had little effect on ballooning, lobular inflammation and fibrosis. Of note, reinforcing the physiologic defense GPX4 was insufficiently potent to alter liver histology. UAMC-3203 acts as a lipophilic radical trapping antioxidant which halts the chain reaction of lipid peroxidation, thereby inhibiting ferroptosis, upon its insertion into plasma membranes, as demonstrated previously [13, 16]. Its beneficial effect on hepatocyte damage and steatosis can be explained by release of oxidized phospholipids and their breakdown products during ferroptosis. Indeed, truncated species of oxidized phosphatidylcholine inhibited mitochondrial oxygen consumption leading to lipid droplet formation in hepatocytes in a MASH mouse model [40]. Likewise, oxidized phospholipids are ligands of peroxisome proliferator activated receptor alpha, thereby influencing lipid metabolism of neighboring hepatocytes [41]. 4HNE released from ferroptotic cells induces stress response pathways and Akt signaling in hepatocytes, resulting in lipid droplet accumulation and insulin resistance [42]. In essence, it remains to be explored how ferroptotic cells mutually interact with the MASH microenvironment. Moreover, metabolites from the L-amino acid oxidase interleukin-4-induced-1 (IL4i1), secreted by myeloid cells, have anti-ferroptotic effects [43, 44]. However, this has not been investigated in this study. Our findings align with previous studies with radical trapping antioxidants, i.e., ferrostatin-1 and liproxstatin-1, in MASLD models. However, in those studies ferroptosis inhibition attenuated hepatic inflammatory infiltrates and fibrosis [45, 46]. Interestingly, ferroptosis inhibition exerted an independent beneficial effect on the liver in HFHFD, after statistical adjustment for weight reduction. Importantly, ferroptosis inhibition did not impact bodyweight in lean animals in this or any other study [14–16].

Another important finding in this study is the dynamic nature of ferroptosis defenses in chronic liver disease. Damaged MASH livers with lipid peroxidation pressure appear more plastic and uphold their remaining ferroptosis defenses (namely, vitamin E, FSP1 and BH4) after loss of hepatocyte GPX4. This finding was surprising as normal livers succumb to spontaneous fatal ferroptosis upon loss of GPX4 because baseline lipid peroxidation pressure builds up. In those livers, we confirmed marked pericentral hepatocyte necrosis with relatively mild inflammatory infiltrates [16, 47]. This dynamic of lipid peroxidation and homeostatic mechanisms help explain the observation that one ferroptosis defense (GPX4) becomes dispensable.

Supplementation of HepG2 with OA/PA boosted PG-PUFA_2_, perhaps to maintain plasma membrane fluidity during overloading with these non-PUFA. Indeed, lower membranous PUFA content reduces fluidity, leading to cell death in a liver fibrosis model [48]. Since mammalian cells cannot perform de novo PUFA synthesis, the PUFA for PG could be derived from greater uptake and elongation of essential PUFA from medium [49]. Importantly, increased PG-PUFA_2_ explains how hepatocytes remain susceptible to ferroptosis, despite abundant accumulation of non-PUFA in MASLD [50].

A limitation of this study is that we could not identify the cell type(s) that commit to ferroptosis. This could be solved by advancements in intravital microscopy with cell type-specific markers to record cell death, although this technique poses great technical challenges [51]. Next, nuclear expression of GPX4 dropped in the subset of MASLD patients with ferroptosis, whereas this epitope became panlobularly expressed in CDAHFD. This highlights interspecies difference, although both displayed hepatic ferroptosis which could be modulated in the pre-clinical model. In addition, we could not compare UAMC-3203 with other ferroptosis inhibitors, since ferrostatin-1 is rapidly cleared from plasma (precluding its use in vivo in models with uncompromised kidney function) and chronic use of liproxstatin-1 proved cumbersome in pilot experiments [52].

In summary, we observed a signature of hepatic ferroptosis in a subgroup of MASLD patients (in 20–50% of subjects) using machine learning. Ferroptosis inhibition in vivo reveals that this cell death is a driver of hepatocyte damage and steatosis, while MASLD livers employ redundant ferroptosis defenses. The therapeutic potential of ferroptosis inhibition in MASLD merits further research, bearing in mind the potential need of patient stratification.

### Supplementary information

Supplementary info

Original western blot 3F

Original western blot 4A

Original western blot 4F

## Data Availability

The next-generation sequencing data have been deposited in NCBI's Gene Expression Omnibus and are accessible through GEO Series accession number GSE267694. R coding for the unsupervised clustering is available at: https://github.com/cedricpeleman. The plugin for FIJI for zonated quantification in liver slides is available at: https://github.com/DeVosLab/Steatosis_ZonatedPortality. Full and uncropped western blots can be found in Fig. S27.
