# Supplementary material for: Ferroptosis is a targetable detrimental factor in metabolic dysfunction-associated steatotic liver disease
Source: Cell Death Differ. 2024 Jul 26;31(9):1113–26. doi: 10.1038/s41418-024-01348-9 (PMC11369286; doi:10.1038/s41418-024-01348-9)
Supplement: Supplementary file 1 — Supplementary info [file 41418_2024_1348_MOESM1_ESM.docx]

**Ferroptosis is a targetable detrimental factor in metabolic dysfunction-associated steatotic liver disease.**

Authors: Cédric Peleman, Stig Hellemans, Geraldine Veeckmans, Wout Arras, Hao Zheng, Ine Koeken, Emily Van San, Behrouz Hassannia, Magali Walravens, Edissa Kayirangwa, Nateneal Tamerat Beyene, Mikhaïl Alfons Van Herck, Winnok Harald De Vos, Isabel Pintelon, Luc van Nassauw, Baptiste Oosterlinck, Annemieke Smet, Lieve Vits, Eveline Dirinck, An Verrijken, Joris De Man, Annelies Van Eyck, Wilhelmus Josephus Kwanten, Luisa Vonghia, Ann Driessen, Koen Augustyns, Shinya Toyokuni, Benedicte De Winter, Christophe Van Steenkiste, Sven Francque, Tom Vanden Berghe.

Table of contents

Supplementary methods..............................................................................................................2

List of antibodies.......................................................................................................................16

Supplementary figures...............................................................................................................17

Supplementary tables................................................................................................................63

Supplementary references.........................................................................................................71

**Supplementary Methods**

*Patient demographics and standard laboratory assessment*

Following patient demographics were collected: age, gender, BMI calculated as bodyweight divided by height squared (kg/m^2^), and the diagnosis of type 2 diabetes mellitus defined according to the American Diabetes Association criteria [1]. Blood samples were collected after overnight fasting, centrifuged and standard laboratory evaluation was performed in the clinical laboratory of the Antwerp University Hospital by an Atellica modular analyzer (Siemens Healthineers, Erlangen, Germany): alanine aminotransferase (ALT), aspartate aminotransferase (AST), γ-glutamyl transpeptidase (GGT), thrombocytes, triglycerides, total cholesterol, HDL high-density lipoprotein (HDL) cholesterol, low-density lipoprotein (LDL) cholesterol, glucose, insulin, ferritin. The following formula was used to calculate homeostasis model assessment–insulin resistance (HOMA-IR): insulin (μU/ml) × glucose (mg/dl)/405 [2]. Additional analyses were performed on serum stored at −80°C in the biobank of the Antwerp University Hospital (BE 71,030,031,000; BBMR-ERIC, Belgian no. access: 1, Last: April 10, 2021 [BIORESOURCE]).

*Cytokeratin-18 fragments with M65 and M30 ELISA*

Caspase-cleaved and total CK-18 fragments were measured in patient serum using enzyme-linked immunosorbent assays, the M30-Apoptosense ELISA (10010) and M65 ELISA (10020, PEVIVA AB, Bromma, Sweden), respectively, as per manufacturer’s instructions. Caspase-cleaved fragments indicate apoptosis of hepatocytes, while total CK-18 fragments correlate with the total amount of cell death, *i.e.* both apoptotic and necrotic. Measurements were performed in duplicate and samples with a coefficient of variation above 15% were excluded from further analysis. The threshold for increased CK-18 values was given by the manufacturer, namely 251 U/L and 413 U/L for the M30-Apoptosense ELISA and M65 ELISA, respectively.

*Genotyping*

*Gpx4^Tg/+^* mice were characterized using PCR primers: 5′-CGTGGAACTGTGAGCTTTGTG-3′ and 5′- AAGGATCACAGAGCTGAGGCTG-3′, yielding a 300 bp DNA fragment upon overexpression of *Gpx4*. Concerning *Gpx4^fl/fl^ AlbCreERT2^Tg/+^* mice: the presence of floxed *Gpx4* alleles were confirmed using PCR primers: 5′- CGTGGAACTGTGAGCTTTGTG and 5′- AAGGATCACAGAGCTGAGGCTG, yielding a 240 bp DNA fragment for floxed *Gpx4*. The presence of *Cre* was evaluated with PCR primer 5′- GCCTGCATTACCGGTCGATGCAACGA and 5′- GTGGCAGATGGCGCGGCAACACCATT, yielding an 800 bp DNA fragment upon presence of Cre.

*Study design for in vivo* experiments.

Concerning the characterization experiments of the MASLD models, C57BL/6J mice were randomized to receive choline-deficient L-amino acid defined diet (CDAHFD, A06071302, Research Diets, NJ, USA) or standard diet (SD, ssniff R/M-H, ssniff Spezialdiäten GmbH, Soest, Germany) for 1, 2, 3, 4 or 6 weeks (n = 5-6). Of note, CDAHFD contains 66mg D,L-α-tocopheryl acetate/kg diet and SD contains 125mg D,L-α-tocopheryl acetate/kg diet. In the context of reduction of the 3Rs, animals from the CDAHFD characterization experiment were reused from a previous experiment where all experimental subjects underwent a parenchymal oxygen measurement of the liver with an oxygen microsensor, as published elsewhere [3]. Likewise, liver samples for characterization of ferroptosis markers were obtained from a prior experiment where C57BL/6J mice were randomized to receive high-fat high-fructose diet (HFHFD, D16042610, Research Diets, NJ, USA) or SD (ssniff R/M-H, ssniff Spezialdiäten GmbH, Soest, Germany) for 20-24 weeks (n = 5-6), as published previously [4]. The practice was adopted in the light of reduction of the 3Rs.

To assess the effect of *Gpx4* overexpression in CDAHFD, *Gpx4^Tg/+^* and wild-type littermates were randomized to receive either CDAHFD or SD for 4 weeks, after which the animals were sacrificed (n = 6-8). Likewise, *Gpx4^fl/fl^ AlbCreERT2^Tg/+^*and their control littermates (*Gpx4^fl/fl^ AlbCreERT2^+/+^)* were randomized to receive CDAHFD or SD. After 4 weeks on their respective diets, all animals received 5 intraperitoneal (i.p.) injections of 100mg/kg tamoxifen (T5648, Sigma-Aldrich, MA, USA) (n = 6-7), dissolved in corn oil with 10% ethanol, on 5 consecutive days to disrupt the loxP-flanked *Gpx4* allele through Cre activation (if possible). Weight, temperature and signs of morbidity were monitored daily and animals were sacrificed under general anesthesia in case of elevated clinical disease score, body temperature below 28°C and/or acute weight loss. In case of no disease signs, animals were sacrificed after a total of 8 weeks on their respective diets.

To evaluate the effect of preventive ferroptosis inhibition, animals fed CDAHFD or SD were randomized to receive i.p. UAMC-3203 once daily at a concentration of 2mM with an injection volume of 100µL per 10g body weight (equivalent to 12.35mg/kg/day), based on previous studies, or 0.9% sterile NaCl (vehicle) [5,6]. After 4 weeks on their respective diets and pharmacologic treatment, animals were sacrificed (n = 10). In the therapeutic setting, animals on CDAHFD or SD for a total duration of 4 weeks were randomized to receive 12.35mg/kg/day UAMC-3203 or vehicle via osmotic minipumps (model 1004, Alzet osmotic pumps, CA, USA) for the last two weeks of the experiment (n = 12-16). Osmotic minipumps were implanted subcutaneously under general anesthesia with isoflurane on a heating pad. After disinfecting the implantation site, a subcutaneous pocket was made on the left dorsal side to place the pump, followed by closure of the dorsal incision with separate sutures (Ethilon 5-0, Ethicon, NJ, USA). Similarly, C57BL/6J mice on HFHFD or SD for 20 weeks received two sequential subcutaneous osmotic minipumps (model 1002, Alzet osmotic pumps, CA, USA) with UAMC-3203 (12.35mg/kg/day) or vehicle. After 2 weeks the first osmotic pump on the left dorsal side was removed and a second osmotic pump placed on the right dorsal side under general anesthesia. After a total of 24 weeks on their respective diets, the experiment was terminated (n = 9).

*Quantification of serum liver tests and lipid parameters in mice*

The blood collected from exsanguination was centrifuged (10 min, 10,000 rpm, 21 °C) to obtain serum. ALT, AST, total cholesterol, HDL cholesterol, LDL cholesterol and triglycerides were measured in the clinical laboratory of the Antwerp University Hospital by an Atellica modular analyzer (Siemens Healthineers, Erlangen, Germany). Non-esterified free fatty acids were measured using the NEFA R1 kit (434-91795, Fujifilm Wako, Neuss, Germany) with read-out on SpectraMax Plus384 (Molecular Devices, CA, USA).

*Histological analysis of MASLD and quantification of macrovesicular steatosis*

Hematoxylin-eosin stain, Sirius red stain, reticulin stain and Perls’ iron stain were routinely performed on all patient biopsies and analysed by an experienced pathologist (A.D.) blinded to any clinical data. Murine livers were fixed in 10% neutral buffered formalin, embedded in paraffin, and cut at 5 µm thickness. Subsequently, sections were stained with hematoxylin and eosin and Masson’s trichrome. Bright-field imaging was performed using the BX43 microscope module (Olympus, Tokyo, Japan) using a 10x/NA 0.25 objective. Histologic features of MASLD, including liver fibrosis, were assessed using the NASH Clinical Research Network (NASH CRN) Scoring System [7]. The combined presence of some degree of steatosis, hepatocellular ballooning and lobular inflammation was necessary to diagnose MASH [8]. These scoring systems were used to partition patients into four groups based on liver histology: no MASLD (controls), metabolic dysfunction-associated steatotic liver (MASL), MASH without significant fibrosis (MASH F0-1), MASH with significant fibrosis (MASH F2-3).

The area of lipid vacuoles, which constitute macrovesicular steatosis, in mouse livers was quantified with methodology adapted from others using FIJI image processing freeware [9,10]. Briefly, 10 random bright-field images were taken with BX43 microscope module (Olympus, Tokyo, Japan) per mouse liver slide at 100x magnification. Potential lipid vacuoles from hepatocytes with macrovesicular steatosis are detected in each image by applying a fixed threshold to the grey values in 8-bit images. Next, lipid vacuoles were distinguished using the ‘analyze particle’ function based on size (95-1512 µm²) and roundness (circularity 0.50-1.00), in order to remove large tissue tears as well as very small objects such as interstitial spaces. These settings were selected by visual examination of 20 random images to avoid false positives, while accepting false negatives, and were applied uniformly to all images. The mean of the ratio of steatotic area to total tissue area, *i.e.* the percentage of liver area pertaining to macrovesicular steatosis, was calculated per experimental subject.

*TUNEL and ferrous iron stain*

The terminal deoxynucleotidyl transferase dUTP nick end labelling (TUNEL) assay was performed to quantify the total amount of cell death in human and mouse formalin-fixed paraffin-embedded (FFPE) liver tissue using the In Situ Cell Death Detection Kit TMR red (12156792910, Roche, Basel, Switzerland) according to the manufacturer’s instructions. Briefly, after deparaffinization tissue slides were pre-treated with proteinase K and exposed to enzyme and labelling solution to visualize DNA strand breaks, followed by counterstaining with DAPI. Random fluorescence micrographs, excitation 620/60 nm for TUNEL and 480/40 nm for DAPI, per experimental subject were acquired with the Leica DMLB microscopy system using the 20x/NA 0.5 objective and Leica Application Suite v4.5.0 as imaging software (Leica, Wetzlar, Germany). The number of TUNEL positive foci per mm² were counted manually.

Non-heme ferrous iron which catalyzes ferroptosis was detected in human liver biopsy specimens using the Turnbull method as described elsewhere [11]. In short, after deparaffinization ferricyanide reacts with ferrous iron to form an insoluble precipitate, enhanced by 3,3′-Diaminobenzidine (DAB) and counterstained with hematoxylin. Imaging was performed with the Nikon Ti2-U Inverted microscope using a 60x/NA 1.2 objective and camera DS-FI2 high definition color camera (Nikon Instruments, Tokyo Japan). The positivity for ferrous iron in sinusoidal cells in human liver biopsies was scored as present or absent.

*Immunohistochemistry*

Sections of FFPE human and mouse liver tissue (5µm thick) were stained with immunohistochemistry (IHC) for several epitopes. After rehydration, heat-induced epitope retrieval was performed with pressure cooker in 0.01M sodium citrate buffer (pH 6.0) for 4HNE, acyl-CoA synthetase long chain family member 4 (ACSL4) and cleaved caspase 3 (CC3), and Tris-EDTA (pH 9.0) for GPX4; endogenous peroxidases and biotin were blocked. Nonspecific binding was blocked with 10% normal goat serum. Primary antibodies for 4HNE (ab46545, abcam, MA, USA), GPX4 (ab125066, abcam, MA, USA), ACSL4 (ab155282, abcam, MA, USA) and CC3 (9661, Cell Signaling Techology, MA, USA) were incubated overnight at room temperature. Binding of the primary antibody was visualized with VECTASTAIN® ABC-HRP Kit (PK-4001, Vector Laboratories, CA, USA) and 3,3'-diaminobenzidine (DAB, D8001, Sigma-Aldrich, MO, USA). IHC for 4HNE in human liver specimens was kindly performed by Hao Zheng and Prof. Dr. Shinya Toyokuni with the HNEJ-1 antibody [12,13]. The number of extracellular intense 4HNE positive aggregates were counted manually and expressed per mm². These aggregates are considered accumulations of ferroptosis breakdown products, as opposed to intracellular lipofuscin granules in hepatocyte cytoplasm which can be 4HNE positive [14].

*Hepatocyte-based quantification of IHC in human liver biopsies*

Human liver biopsy specimens were digitalized with the Zeiss Axioscan Z1 automated whole slide scanner (Zeiss, Oberkochen, Germany) using a 20x objective lens (pixel size 0,22 x 0,22 µm). To quantify nuclear and cytoplasmic expression of epitopes, hepatocytes were reconstructed in needle liver biopsies based on artificial intelligence (AI)-aided recognition of nuclei and their cytoplasm. Whole slide images were loaded into QuPath version 0.4.4. to be processed with the image analysis script available at: https://github.com/stighellemans [15]. Briefly, tissue area for the analysis, without tears and large vessels structures, was annotated manually and divided into tiles. Nuclei were detected using the StarDist2D version 0.3.0 with pretrained model for brightfield microscopy and 0.5 as threshold for detection probability [16]. A perimeter around the nucleus was drawn to approximate the corresponding cytoplasm, while excluding pixels pertaining to lipid vacuoles as detected with a user-trained pixel classifier in QuPath. Data on size, mean and maximal DAB intensity of nuclei and cytoplasm was exported for further processing in R by means of the R-package ”dplyr” for grouped summary statistics [17]. Cells with nuclear area <11.5µm² were excluded from further analysis to filter out immune cells and debris. To calculate the percentage of epitope positive nuclei per patient, the mean DAB intensity was binarized with 0.7 optical density as threshold. Concerning the cytoplasmic expression of epitopes, cytoplasmic mean DAB intensity was binned into three grades of intensity (score 1, 2 or 3) based on the 33.3 and 66.6 percentiles, resembling evaluation by a pathologist. Next, the weighted score was calculated to reflect cytoplasmic expression of an epitope in all hepatocytes per patient.

*Zonated quantification of IHC in mice*

To quantify the lobular distribution of IHC epitopes in murine livers, whole liver slides were digitalized with the same slide scanner as indicated above. Quantification in different zones of reconstructed liver lobules was performed in FIJI with an image analysis script developed previously [10,18]. Briefly, color deconvolution was applied to isolate the positive DAB signal by means of a threshold. As liver lobules are organized according to Voronoi diagrams, they were reconstructed after manual annotation of central veins and liver capsule [19]. Pixels within each lobule received a relative distance value (0 at the lobular edge to 1 in the centrolobular vein) and DAB signal. The results were processed in RStudio with the R-package ”dplyr” for grouped summary statistics to calculate the percentage of DAB positive in different zones of all liver lobules per experimental subject [17].

*Colorometric MDA assay*

Serum MDA from patients, reflecting total body lipid peroxidation, and hepatic MDA in mouse livers was measured using the N-methyl-2-phenylindole colorometric assay, as described elsewhere [5,20]. Murine liver tissue was lysed by mechanical disruption in phosphate-buffered saline and supernatant collected. Briefly, 100µL of patient serum or murine liver supernatant was combined with 10mM N-methyl 2-phenylindole (444808, Sigma-Aldrich, MA, USA), dissolved in a mixture of acetonitrile and methanol, to form a chromogen. The reaction was initiated by adding 37% hydrochloric acid and incubating at 70 °C for 45 min. The 595 nm absorbance was measured with the SPECTROstar Nano (BMG Labtech, Ortenberg, Germany), and MDA concentration was calculated with a standard of 1,1,3,3-tetramethoxypropane (108383, Sigma-Aldrich, MA, USA) as a source of MDA. Hepatic MDA levels were normalized for the protein content as measured with Bradford assay per manufacturer’s instructions (5000006, Bio-Rad, CA, USA).

*Serum vitamin E measurement in mice*

Serum vitamin E, reflecting whole body vitamin E levels, was measured in mouse serum using the Vitamin A & E reagent set (1080 kit M VAE, diagnotix BV, Appingedam, Netherland) on the QTRAP5500 (AB Sciex, MA, USA) liquid chromatography tandem-mass spectrometry (LC-MS/MS) system in the clinical laboratory of the Antwerp University Hospital. Briefly, after separation by chromatography on an analytical C-18 column, samples are analyzed using positive ion electrospray in multiple reaction monitoring mode. An isotope labelled internal standard is included. The amount of serum vitamin E (µM) is derived from comparison to a standard.

*Targeted metabolomics for BH4, BH2, NAD(P)(H) in mouse liver*

Mass Spectrometry measurements were performed using Vanquish LC System (Thermo Scientific) coupled via heated electrospray ionization to a Q Exactive Orbitrap Focus mass spectrometer (Thermo Scientific) operated in negative mode. 10 μl sample was taken from an MS vial and injected onto a 15cm Poroshell 120 HILIC-Z PEEK Column (Agilent InfinityLab). A linear gradient was carried out starting with 90% solvent A (acetonitrile with 5µM medronic acid) and 10% solvent B (10 mM NH4-formate in milli-Q water, pH 3.8). From 2 to 12 min the gradient changed to 60% B. The gradient was kept on 60% B for 3 minutes and followed by a decrease to 10% B. The chromatography was stopped at 25 min. The flow was kept constant at 0.25 ml/min and the column was kept at 25°C throughout the analysis. The HESI-source operated at negative polarity mode using a spray voltage of 3 kV, sheath gas at 45, auxiliary gas at 10, the latter heated to 260°C. The ion transfer capillary temperature was 320°C. The mass spectrometer operated in full scan (range [70.0000-1050.0000]) and AGC target was set at 3.0E+006 using a resolution of 70000. Data collection was performed using the Xcalibur software (Thermo Scientific). The data analyses were performed by integrating the peak areas (El-Maven – Polly - Elucidata).

*Targeted metabolomics for vitamin E, GSH and GSSG in mouse liver*

Mass Spectrometry measurements were performed using a Dionex UltiMate 3000 LC System (Thermo Scientific Bremen, Germany) coupled via heated electrospray ionization to a Q Exactive Orbitrap to a Q Exactive Orbitrap mass spectrometer (Thermo Scientific). 10 μl sample was taken from an MS vial and injected onto a 15cm C-18 column (Acquity UPLC -HSS T3 1. 8 μm; 2.1 x 150 mm, Waters). A step gradient was carried out using solvent A (10 mM TBA and 15 mM acetic acid in milliQ) and solvent B (100% methanol). The gradient started with 5% of solvent B and 95% solvent A and remained at 5% B until 2 min post injection. A linear gradient to 37% B was carried out until 7 min and increased to 41% until 14 min. Between 14 and 26 minutes the gradient increased to 95% of B and remained at 95% B until 30 minutes. Then the gradient increased further to 99%B in 6 seconds, then remained at 99% until 38 minutes. At 38 min the gradient returned to 5% B within 1 minute. The chromatography was stopped at 48 min. The flow was kept constant at 0.25 mL/min and the column was kept at 40°C throughout the analysis. The HESI-source operated at negative polarity mode using a spray voltage of 4.8 kV, sheath gas at 40, auxiliary gas at 10, the latter heated to 260°C. The ion transfer capillary temperature was 300°C. The mass spectrometer operated in full scan (range [70.0000-1050.0000]) and AGC target was set at 3.0E+006 using a resolution of 140000. Data collection was performed using the Xcalibur software (Thermo Scientific). The data analyses were performed by integrating the peak areas (El-Maven – Polly - Elucidata).

*Colorometric test for reduced GSH measurement in mouse liver*

Mouse hepatic reduced glutathione (GSH) levels were measured using the Quantichrom Glutathione Assay Kit (DIGT-250, BioAssay Systems, CA, USA) according to the manufacturer’s instructions. In short, murine liver tissue was lysed mechanically in phosphate-buffered saline to collect supernatant. After deproteination of the supernatant, 5,5’-dithiobis-nitrobenzoic acid combines with reduced GSH to form a chromogen. Optical density was measured at 412nm with the SPECTROstar Nano (BMG Labtech, Ortenberg, Germany) and the absolute amount determined by comparison with a standard of reduced GSH (G4251, Sigma-Aldrich, MA, USA). Hepatic reduced glutathione levels were normalized for the protein content as measured with Bradford assay per manufacturer’s instructions (5000006, Bio-Rad, CA, USA).

*Real-time quantitative PCR on mouse tissue*

Total RNA was isolated from murine liver tissue with the NucleoSpin RNA plus kit (Macherey-Nagel, Düren, Germany) following the manufacturer’s instructions. RNA concentration and quality parameters were measured using the NanoDrop ND-1000 (Thermo Fisher Scientific, MA, USA). By means of the SensiFAST™ cDNA Synthesis Kit (Meridian Bioscience, OH, USA) 800ng RNA was converted to cDNA by reverse transcription. For SYBR Green real-time quantitative PCR (RT-qPCR) cDNA was added to a final volume of 20µL with GoTaq qPCR master mix (A6002, Promega, WI, USA) and primers (Sigma-Aldrich, MA, USA) in the QuantStudio 3 Real-Time PCR system (Thermo Fisher Scientific, MA, USA). The following mouse-specific primers were used: *Gpx4* forward, 5′- GCAGGAGCCAGGAAGTAATC -3′, *Gpx4* reverse, 5′- GGCTGGACTTTCATCCATTT -3′; *Fsp1* forward, 5′- CATGGTGATTGTGTGCAATGG -3′, *Fsp1* reverse, 5′- TGGTCAGTCTCTGGCTTGTAAG -3′; *Gch1* forward, 5′- ACTTCACCAAGGGATACCAGG -3′, *Gch1* reverse, 5′- CTTGCTTGTTAGGAAGATAGCCA -3′; *Ptgs2* forward, 5′- TGAGCAACTATTCCAAACCAGC -3′, *Ptgs2* reverse, 5′- GCACGTAGTCTTCGATCACTATC -3′; *Hprt1* forward, 5′- TCAGTCAACGGGGGACATAAA -3′; *Hprt1* reverse, 5′- GGGGCTGTACTGCTTAACCAG -3′; *Gapdh* forward, 5′- TGACCTCAACTACATGGTCTACA -3′, *Gapdh* reverse, 5′- CTTCCCATTCTCGGCCTTG -3′; *Actb* forward, 5′- GTGACGTTGACATCCGTAAAGA -3′, *Actb* reverse, 5′- GCCGGACTCATCGTACTCC -3′ and *Rpl4* forward, 5’- CCGTCCCCTCATATCGGTG -3′, *Rpl4* reverse, 5′- GCATAGGGCTGTCTGTTGTTTTT -3′. RT-qPCR assays were run in duplicate and results were exported from Thermo Fisher Connect and analysed with the delta-delta Ct method using qbase+ (Biogazelle, Ghent, Belgium), expressed as calibrated normalized relative quantities (CNRQ), with normalization for an appropriate combination of housekeeping genes.

*Western blot analysis*

Proteins were extracted from mouse liver tissue in NP-40-based lysis buffer supplemented with protease (cOmplete™ Protease Inhibitor Cocktail, Roche, Basel, Switzerland) and phosphatase inhibitors (PhosSTOP, Roche, Basel, Switzerland). After 4 min heating at 95°C and dilution in Laemmli buffer (S3401, Sigma-Aldrich, MA, USA) with β-mercaptoethanol, 10µg of liver protein lysate was loaded onto NuPAGE 12% Bis-Tris gel (Thermo Fisher Scientific, MA, USA) for protein separation by gel electrophoresis in MOPS-based running buffer. Proteins were transferred to an Immobilon PVDF membrane (Millipore, MA, USA) by wet blotting in Tris-methanol-based buffer, followed by blocking with Intercept (PBS) Blocking Buffer (LI-COR Biosciences, NE, USA). Overnight incubation at 4°C with primary antibodies against GPX4 (1:1000, ab125066, abcam, MA, USA) and β-actin (1:5000, ab8226, abcam, MA, USA), followed by washing and labeling with IRDye® 680RD Goat anti-Rabbit IgG Secondary Antibody (1:20,000, 926-68071, LI-COR Biosciences, NE, USA) and IRDye® 800CW Goat anti-Mouse IgG Secondary Antibody (1:20,000, 926-32210, LI-COR Biosciences, NE, USA) for 1 hour at room temperature to detect the target GPX4 and loading control β-actin, respectively, with the Odyssey imager system (LI-COR Biosciences, NE, USA) in the near-infrared spectrum. Image Studio Software v4.0 (LI-COR Biosciences, NE, USA) was used for blot visualization.

*Cell line and in vitro MASH environment*

Human hepatoma HepG2 cells, a kind gift from Prof. Dr. Wim Wuyts, were grown at 37°C in 5% O_2_ in Eagle's Minimum Essential Medium (EMEM, 30-2003, ATCC, VA, USA) supplemented with 10% heat-inactivated fetal bovine serum (16000044, Gibco, Thermo Fisher Scientific, MA, USA), 100 U/mL penicillin and 100 µg/mL streptomycin (15140122, Gibco, Thermo Fisher Scientific, MA, USA). This constituted the standard medium. Cell line authenticity was carried out by short-tandem repeat profiling (last performed on 20/10/2021). Mycoplasma contamination testing was performed regularly using the Mycoplasma Detection Kit (13100-01, SouthernBiotech, AL, USA). All *in vitro* experiments were performed in triplicate.

For the *in vitro* MASH environment, HepG2 cells were seeded in 96-well plates at a concentration of 200,000 cells/ml and medium replaced after 24 hours with standard medium supplemented with 3.5 mg/mL glucose, 100 nM insulin, 50 ng/mL tumor necrosis factor (TNF)-α (300-01A, PeproTech, London, UK), 25 ng/mL interleukin (IL)-1β (200-01B, PeproTech, London, UK) and 8ng/mL transforming growth factor (TGF)-β (100-21, PeproTech, London, UK), as well as different types of free fatty acids, *i.e.* the combination of 100µM oleic (OA, O7501, Sigma-Aldrich, MA, USA) and 50µM palmitic acid (PA, P9767, Sigma-Aldrich, MA, USA), 10µM arachidonic acid (AA 20:4, A3611, Sigma-Aldrich, MA, USA) or 10µM docosahexaenoic acid (DHA 22:6, D2534, Sigma-Aldrich, MA, USA), as adapted from the work of other authors [21]. OA was conjugated beforehand to 10% BSA (10735078001, Roche, Basel, Switzerland), while PA and AA/DHA were dissolved in methanol and ethanol, respectively. Standard medium supplemented with 0.33% BSA, 0.0333% methanol and 0.01% ethanol was included as solvent control (control). After 48 hours incubation in MASH environment with different types of FFA or control media, the amount of intracellular neutral lipids was measured using AdipoRed Assay Reagent (Lonza, Basel, Switzerland), as per manufacturer’s instruction, and cell pellet was collected on ice and stored at minus 80°C for further analysis.

*Cell death and lipid peroxidation assays in vitro*

Total cell death was measured with SytoxGreen Nucleic Acid stain (S7020, Invitrogen, MA, USA) using the FLUOstar Omega (BMG Labtech GmbH, Ortenberg, Germany) [22]. After 48 hours in standard medium supplemented with different species of free fatty acids or solvent control (control), cells in 96-well plates were treated with increasing concentrations of GPX4 inhibitor ML162 (20455, Cayman Chemical, MI, USA) in the presence of 2µM SytoxGreen. Twenty-four hours later the fluorescence intensity was measured with excitation/emission filter 485/520 nm for SytoxGreen. The percentage of cell death per well was calculated by dividing through the signal intensity of a well exposed to 0.05% Triton X-100, or the highest intensity from another well if more appropriate. The relation between the percentage of cell death and concentration of ML162 was described with a non-linear regression with GraphPad Prism 10 (GraphPad Software, CA, USA) and best-fit logarithms of the half maximal effective concentration (EC50) values were compared between different conditions using Aikake Information Coefficient, followed by one-way ANOVA with post-hoc testing. Moreover, cell death was confirmed through visual inspection of SytoxGreen fluorescence combined with bright field images of the Olympus CKX53 microscope. Images were acquired with the ToupView software (ToupTek, Hangzhou, P.R. China) and combined with the image calculator function in FIJI [10]. All *in vitro* cell death experiments were performed three independent times.

In order to confirm the presence of ferroptosis by pharmacological GPX4 inhibition in HepG2, lipid radical oxygen species in cell membranes was visualized with the ratiometric dye C11-BODIPY (581/591) (D3861, Thermo Fisher Scientific, MA, USA) [23]. Briefly, cells were seeded on 96 Well Black/Clear Bottom Plate (165305, Thermo Fisher Scientific, MA, USA) and incubated for 48 hours with standard medium with different species of free fatty acids or control. Subsequently, cells were incubated with 5µM ML162 or 100µM tert-butyl hydroperoxide for 2 hours in the presence of 10µM C11-BODIPY (581/591) for the last 30 minutes. Finally, all media and dyes were replaced by HEPES/Tris-based imaging buffer and imaged using excitation/emission 561/617-673 for the reduced form and 488/520-535 for the oxidized form of C11-BODIPY (581/591) with the Nikon CSU-W1 SoRa spinning disk confocal microscope (Nikon Instruments, Tokyo, Japan). This experiment was performed once to provide visual evaluation.

*Poly(A) mRNA-sequencing on in vitro experiments*

Total RNA was isolated from cell pellets from 3 independent experiments with the NucleoSpin RNA plus kit (Macherey-Nagel, Düren, Germany) following the manufacturer’s instructions, followed by measurement of total RNA with Qubit 2.0 (ThermoFisher Scientific, MA, USA). Quality control of RNA integrity was deemed sufficient (RINe > 6.8) as measured with the 4200 Tapestation system with TapeStation Analysis Software 3.2 (Agilent Technologies, CA, USA). Next, library preparation started with isolation of intact poly(A) RNA by means of NEBNext poly(A) mRNA magnetic isolation (E7490, New England Biolabs, MA, USA), followed by setup of directional RNA libraries by means of NEBNext Ultra II directional RNA library prep kit (E7760) in combination with NEBNext multiplex oligos for Illumina (E7335, New England Biolabs, MA, USA). Quality control of the library was performed with removal of adapter dimers by means of E-gel 2% agarose/ZymoClean gel DNA recovery kit (D4007, CA, USA) and visualization of final library traces by means of 4200 TapeStation (Agilent Technologies, CA, USA). Before next-generation sequencing, the concentration of the library was measured by means of Qubit 2.0 to ensure equimolar pooling of all samples. Sequencing was performed using the Illumina NovaSeq6000 (Illumina, CA, USA) at a depth of 30 million paired-end (150bp) reads per sample. The sequencing information was basecalled into raw FASTQ files. Subsequently, adapter sequences and low-quality parts of the raw reads were trimmed with SeqPurge.

In a Linux environment FastQC was used to check quality of reads before mapping to the indexed human reference genome GRCh38 (hg38) using STAR to produce a BAM file. The aligned reads in BAM files were sorted and indexed using SAMtools, followed by quantification of the number of reads per gene per sample with featureCounts. Read counts were processed in RStudio with packages “DESeq2”, “fgsea” and “pheatmap”. Only genes with counts over 10 in at least 3 samples were retained and count normalization was performed by DESeq2’s median of ratios normalization based on total counts per sample to allow for gene count comparisons between samples (not based on gene length). Expression levels of ferroptosis-related genes were visualized with a heatmap scaled per gene. Differential gene expression analysis with DESeq2, while gene set enrichment analysis (GSEA) was used to assess the expression of the 4 user-defined gene sets (mentioned above) between the two conditions: MASH environment with OA and PA vs. control.

*Untargeted lipidomics*

Lipidomics on cell pellet was performed as described earlier [24]. Briefly, 700 μl of homogenized cells was mixed with 800 μl 1 N HCl:CH3OH 1:8 (v/v), 900 μl CHCl3, 200 μg/ml of the antioxidant 2,6-di-tert-butyl-4-methylphenol and 3 μl of SPLASH LIPIDOMIX Mass Spec Standard (330,707, Avanti Polar Lipids, AL, USA). The organic fraction was collected and evaporated; the remaining lipid pellet was stored at—20 °C under argon. Following lipid extraction, lipid pellets were reconstituted in 100% ethanol. Lipid species were analyzed by electrospray ionization LC-MS/MS on a Nexera X2 UHPLC system (Shimadzu, Kyoto, Japan) coupled with hybrid triple quadrupole/linear ion trap mass spectrometer (6500 + QTRAP system, AB SCIEX, MA, USA). Chromatographic separation was performed on a XBridge amide column (150 mm × 4.6 mm, 3.5 μm; Waters Corp, MA, USA). Cholesterol esters and ceramides were measured in positive ion mode. Triglyceride and diacylglycerides were measured in positive ion mode with a neutral loss scan for one of the fatty acyl moieties. Phospholipid species were measured in negative ion mode by fatty acyl fragment ions. Lipid quantification was performed by scheduled multiple reactions monitoring. Peak integration was performed with the MultiQuant™ software version 3.0.3. Lipid species signals were corrected for isotopic contributions (calculated with Python Molmass 2019.1.1) and were quantified based on internal standard signals and adheres to the guidelines of the Lipidomics Standards Initiative.

*Unsupervised clustering of MASLD patients and controls*

To study patients with a hepatic ferroptosis signature, unsupervised clustering by means of k-prototypes partitioning clustering was performed to find discrete subgroups among the MASLD patients and controls in whom cell death markers were measured in liver biopsy specimens [25]. This allows for the detection of latent classes based on independent variables without selection for a dependent variable. The optimal number of clusters was chosen based on the elbow method for the total within-cluster sum of squares metric, which indicates that increasing the number of clusters improves the similarity of samples within one cluster, but only to a given point. Hence, three clusters were defined based on patient’s histologic results for 4HNE, TUNEL, GPX4, ACSL4 and ferrous iron stain (four continuous variables and one categorical variable). The unsupervised clustering was performed with a script in RStudio expanded with packages “clustMixType” and “tidyverse” which can be found at: <https://github.com/cedricpeleman> [17].

*mRNA sequencing analysis in publicly available databases with unsupervised clustering*

Individual SRA files were downloaded from their deposits in NCBI’s Gene Expression Omnibus (GEO) [26] accessible through GEO Series accession numbers GSE135251 [27], GSE130970 [28] and GSE126848 [29]. In a Linux environment SRA toolkit converted SRA into FASTQ files and FastQC was used to check read quality. STAR was used to map reads to the indexed human reference genome GRCh38 (hg38) in a sorted BAM file and the reads per gene per sample were determined using featureCounts which were processed in Rstudio, expanded with packages “dplyr”, “DESeq2”, “sva” and “GSVA” [10]. Only genes with counts over 10 in at least 3 samples were retained. Variance stabilizing transformation was followed by correction for batch effects with surrogate variable analysis in order to prepare data for entry into gene set variation analysis (GSVA) [30]. The latter generates a GSVA enrichment score (in arbitrary units) for the expression of a user-defined gene set per individual patient in comparison to all other subjects for use in downstream analysis [31]. The following 4 gene sets were defined in relation to the execution of ferroptosis: ‘ferroptosis defenses’ whose increased expression counters ferroptosis, ‘GSH’ with genes that promote reduced glutathione (GSH) used by GPX4 to counter ferroptosis, ‘PUFA’-related genes which increase PUFA incorporation into membrane phospholipids promoting ferroptosis, and the ‘iron’ gene set which would lead to higher ferrous iron that catalyzes ferroptosis. The genes included in these 4 gene sets can be found in Table S2.

Subsequently, unsupervised clustering was used to discern latent subgroups among MASLD patients and controls based on the GSVA scores per gene set for each patient. Model-based clustering, more precisely Gaussian Mixture models, was used to visualize clusters with certain volume, shape and orientation, using the “mClust” package in RStudio which employs the expectation-maximization algorithm [32]. Additional packages used were “factoextra” and “clusterSim”. The optimal number of clusters in every patient data set was chosen based on the lowest Bayesian Information Criterion (BIC) and lowest Integrated Complete-data Likelihood (ICL) [33,34]. In addition, the Davies-Bouldin index was calculated per given number of clusters and the elbow method applied [35]. Model-based clustering is frequently used to discern subgroups with distinct profiles of independent variables within heterogenous patient cohorts [36–38].

**List of antibodies**

| Name | Supplier | Cat no. | Clone no. |
| --- | --- | --- | --- |
| Anti-4HNE | Prof. Dr. S. Toyokuni | HNEJ-1 | monoclonal |
| Anti-4HNE | abcam | Ab46545 | Polyclonal |
| Anti-GPX4 | abcam | Ab125066 | Monoclonal - EPNCIR144 |
| Anti-ACSL4 | abcam | Ab155282 | Monocloncal - EPR8640 |
| Anti-CC3 | Cell Signaling Technology | 9661 | Polyclonal |
| Anti-β -actin | abcam | Ab8226 | Monoclonal - mAbcam 8226 |

**Supplementary Figures**

Figure S1


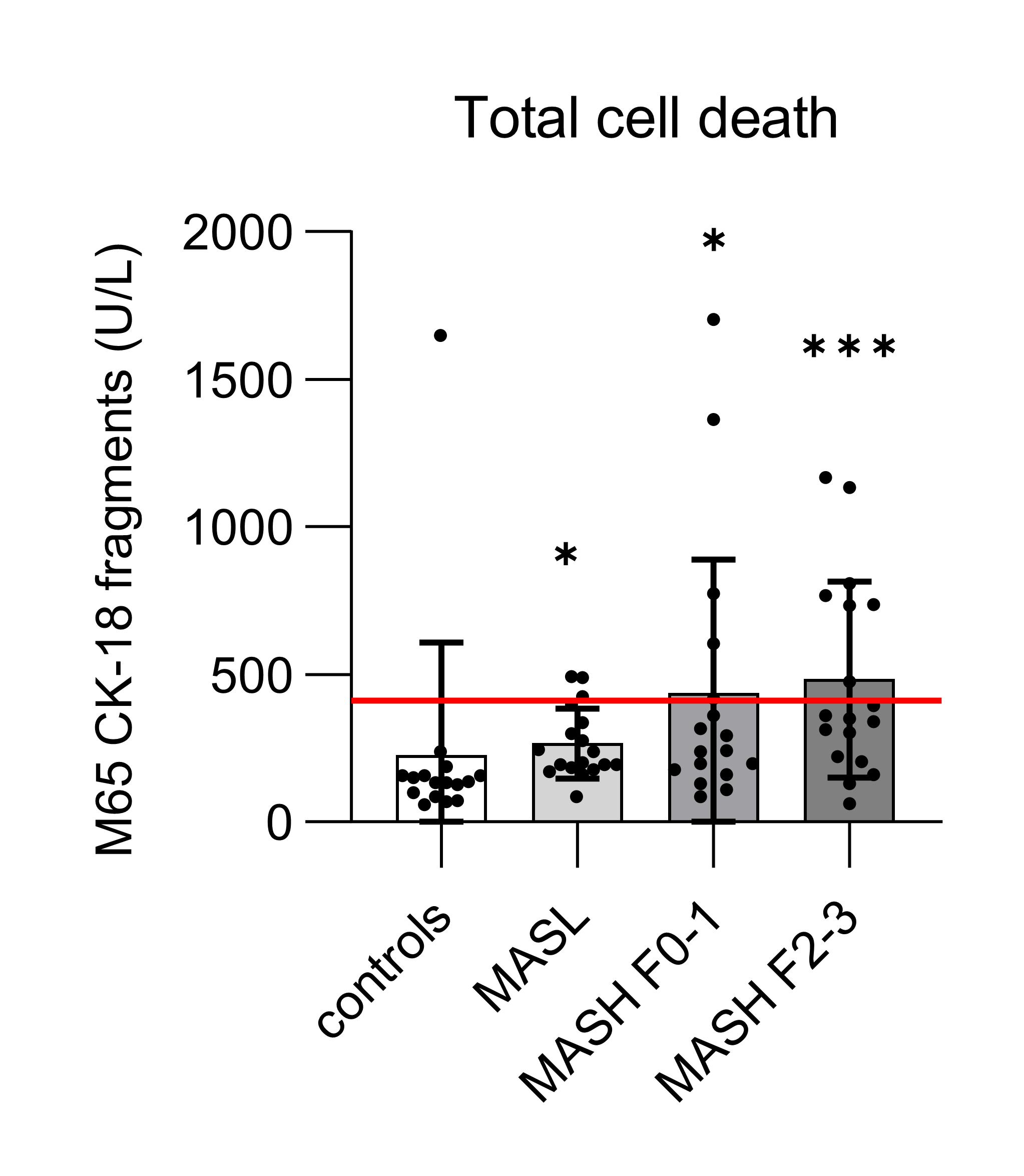

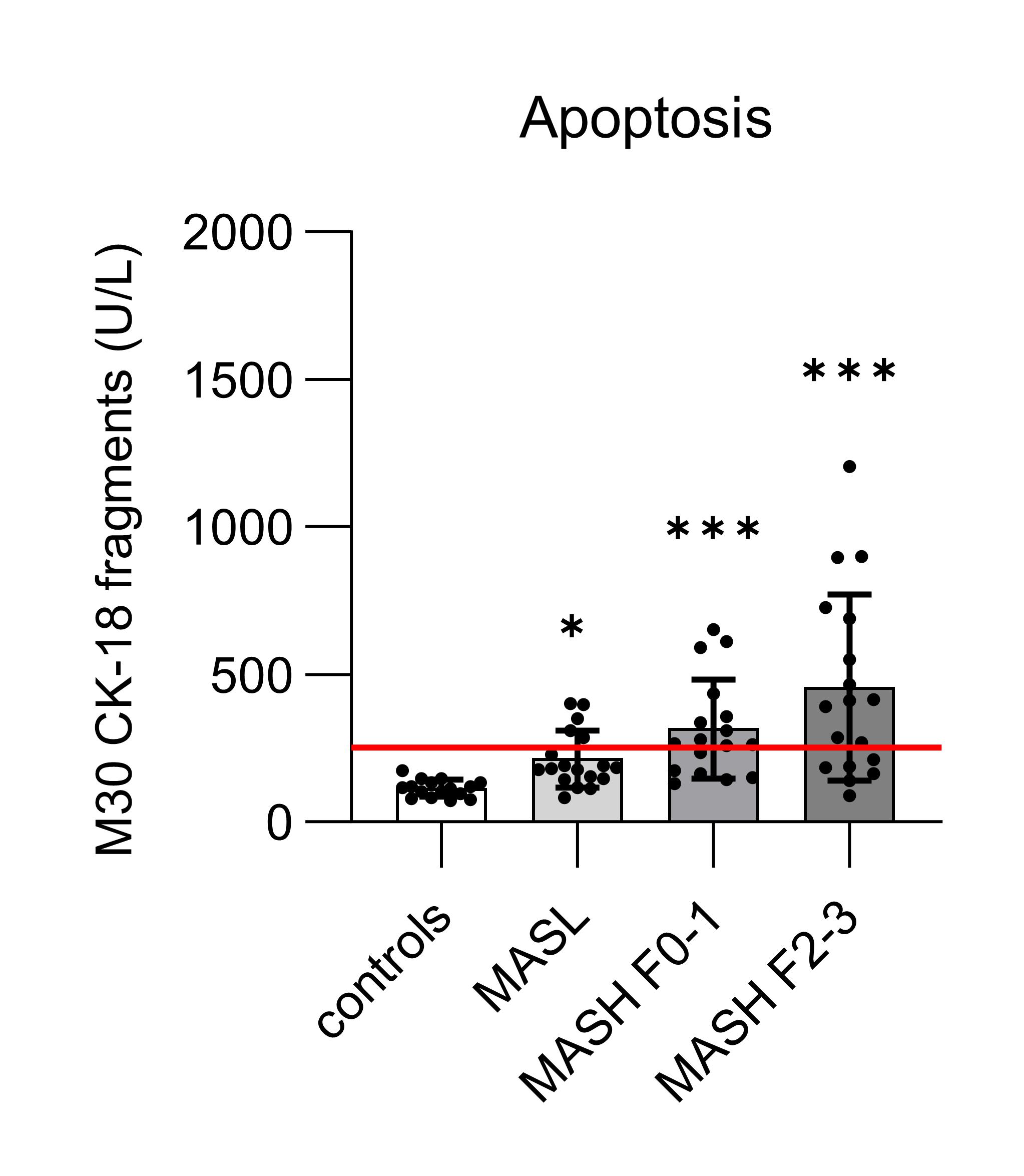

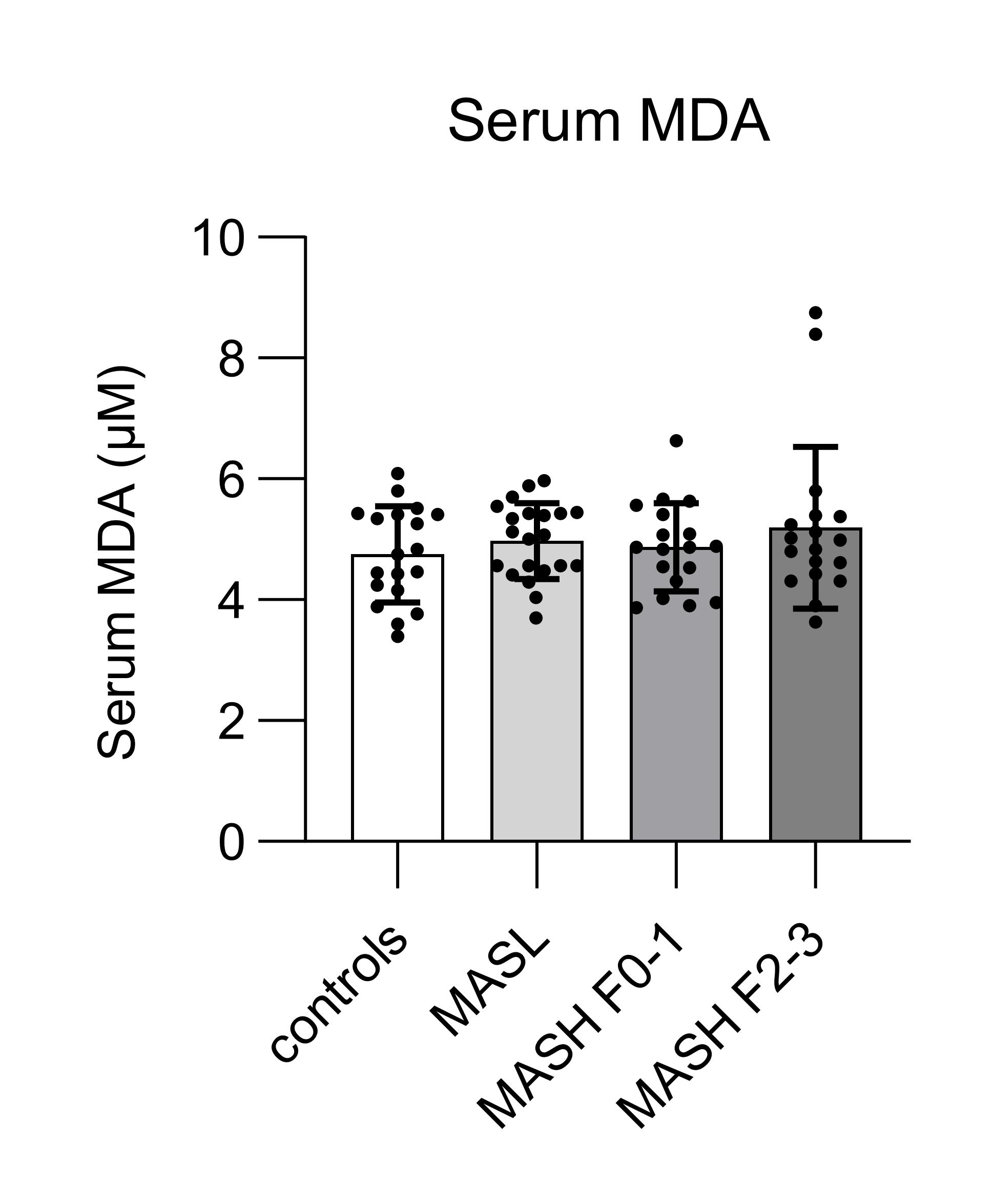


A

B

p = 0.74

**Fig. S1. Serum cytokeratin-18 fragments and serum MDA in MASLD patients and controls.**

(A) Cytokeratin-18 (CK18) fragments derived from total cell death and apoptosis of hepatocytes were measured by means of the M65 and M30 Apoptosense ELISA kits, respectively, in controls (n = 16) and MASLD patients (n = 53). The latter were divided according to liver histology in isolated steatosis (MASL, n = 18), metabolic dysfunction-associated steatohepatitis without significant fibrosis (MASH F0-1, n = 17) and metabolic dysfunction-associated steatohepatitis with significant fibrosis (MASH F2-3, n = 18). The horizontal red line presents the upper limit of normal values as per manufacturer’s instructions. (B) By means of a colorometric assay with N-methyl-2-phenylindole, the ferroptosis breakdown product malondialdehyde (MDA) was measured in serum of the same groups of subjects (p = 0.74). Data presented as mean ± standard deviation. *p <0.05; ***p <0.001; Kruskal-Wallis test with Dunn’s multiple comparisons test.

Figure S2.

*Gpx4^fl/fl^ AlbCreERT2^Tg/+^* Omission of labelling enzyme

B

A


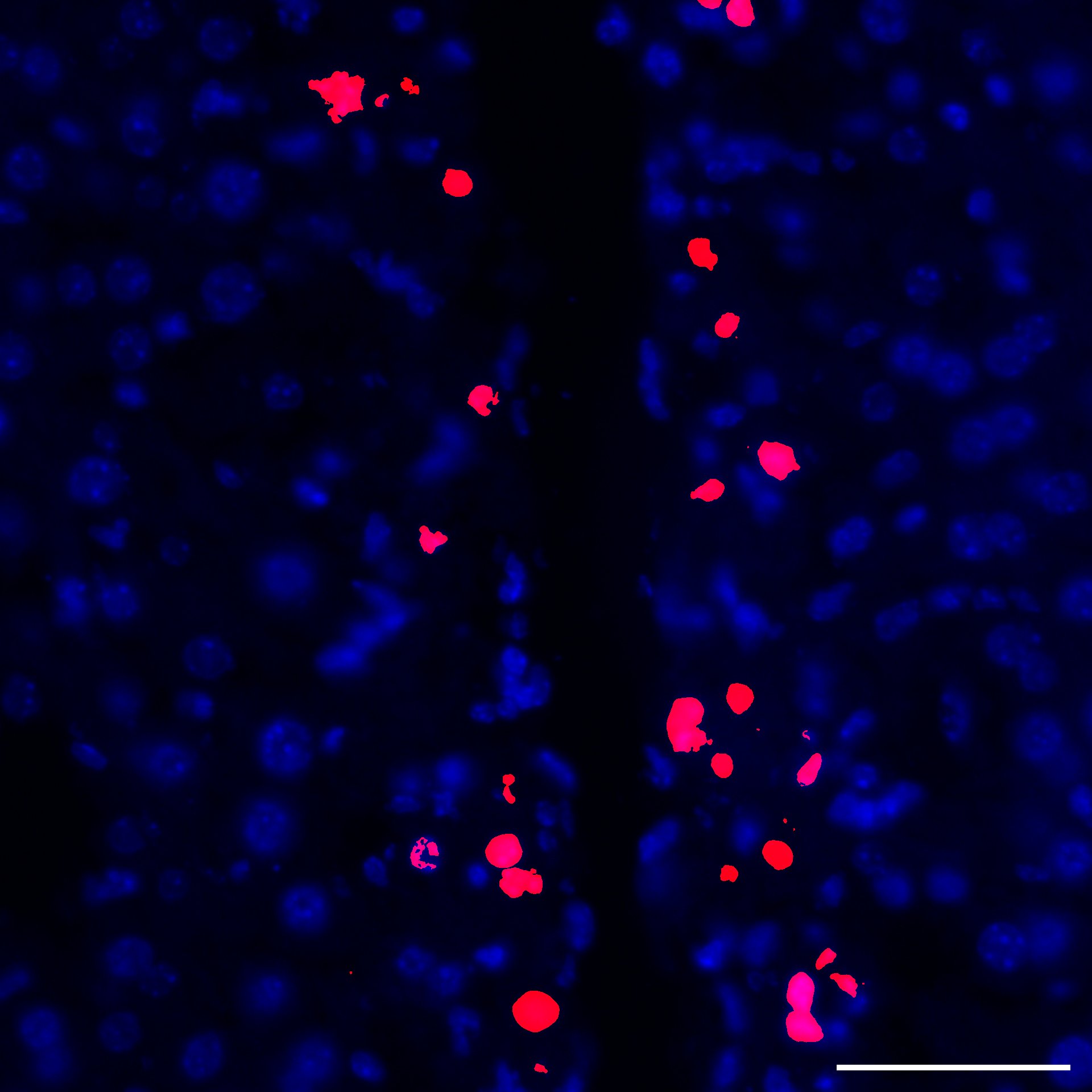

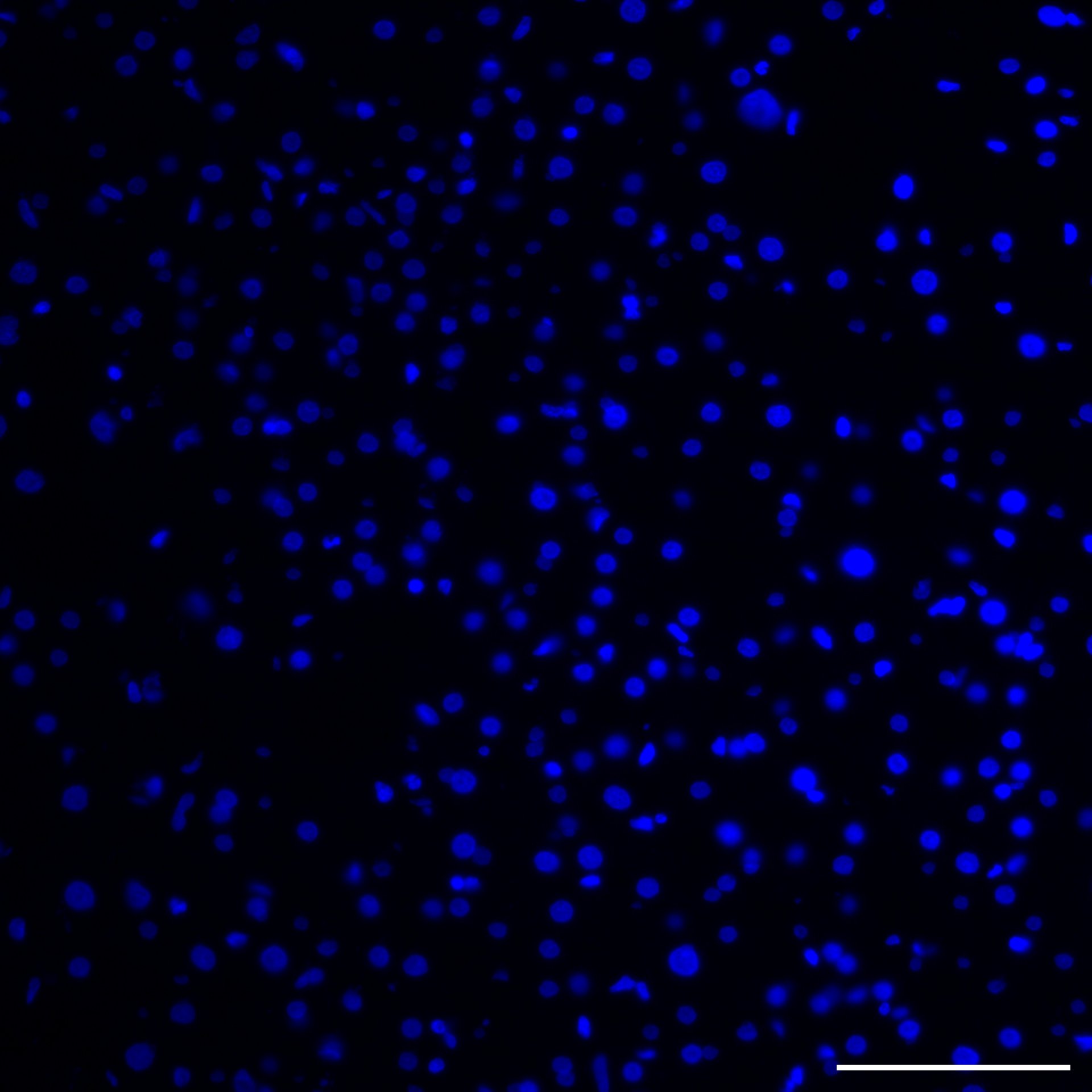


TUNEL

**Fig. S2. Positive control and control with omission of labelling enzyme for TUNEL in human liver biopsy specimens.**

(A)TUNEL in liver tissue from *Gpx4^fl/fl^ AlbCreERT2^Tg/+^* mouse fed the standard diet and sacrificed upon clinical deterioration after intraperitoneal administration of tamoxifen (100mg/kg bodyweight). Nuclei in the pericentral region are terminal deoxynucleotidyl transferase dUTP nick end labelling (TUNEL) positive (red), while all nuclei are counterstained with DAPI (blue). (B) Upon omission of the labelling enzyme solution, no TUNEL positivity is observed. Scale bar 200µm, 100x magnification.

Figure S3.

HNEJ-1 Omission primary antibody

Control


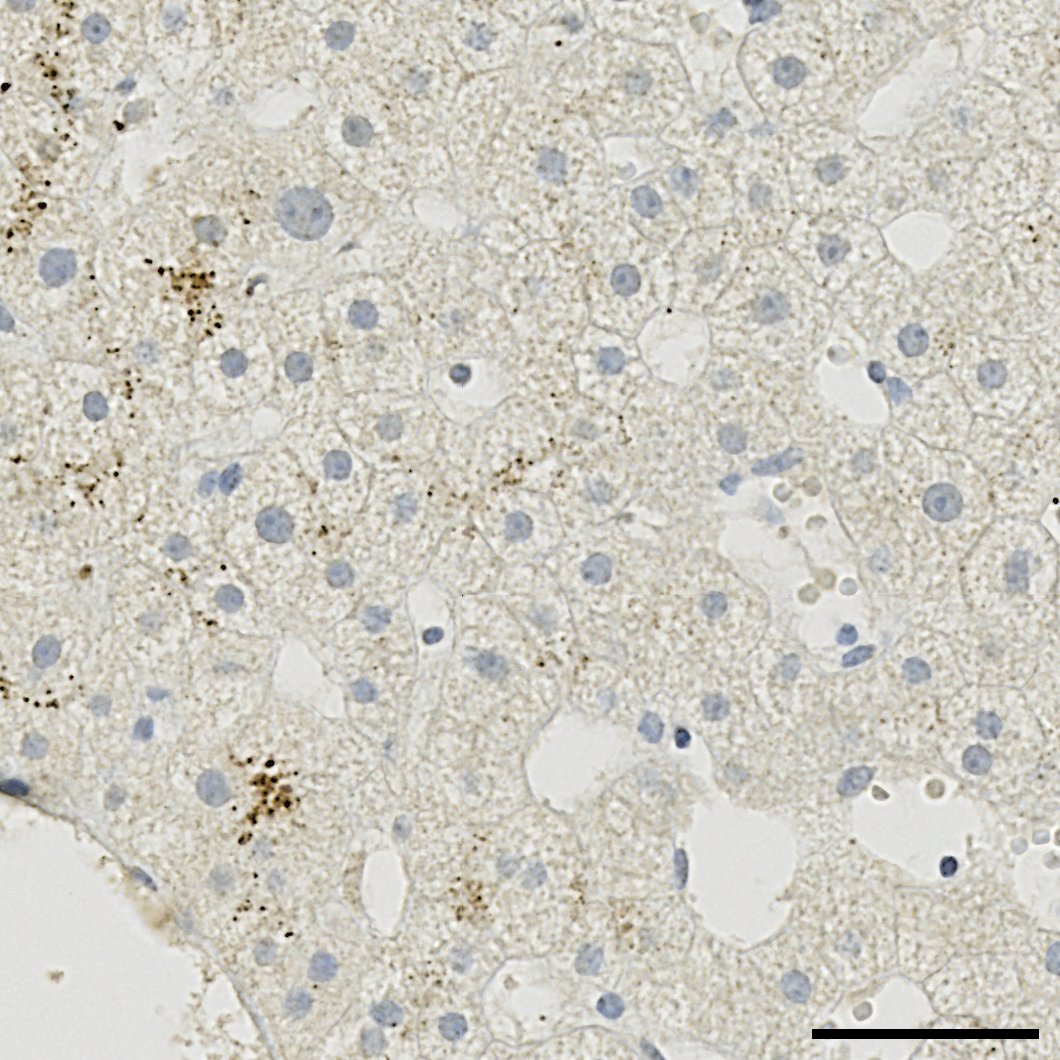

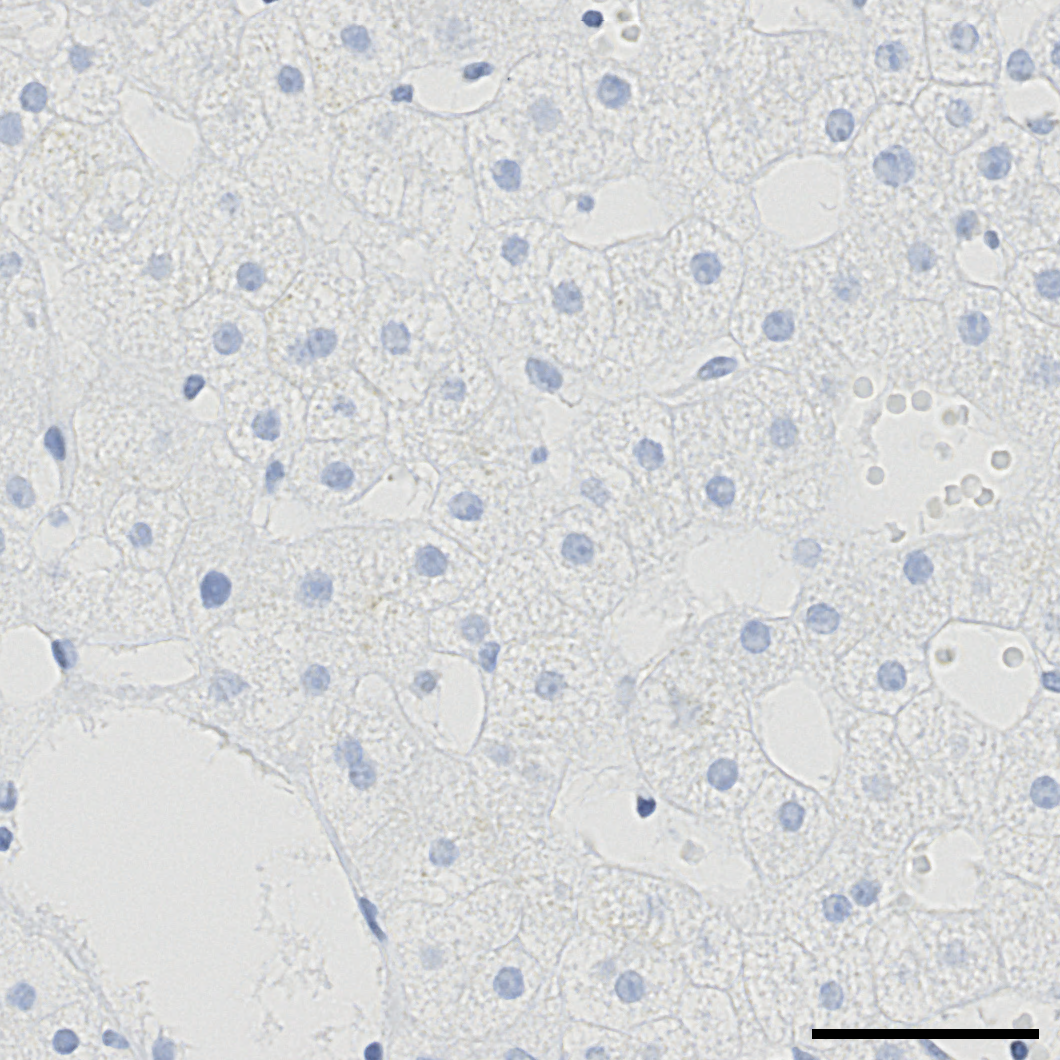


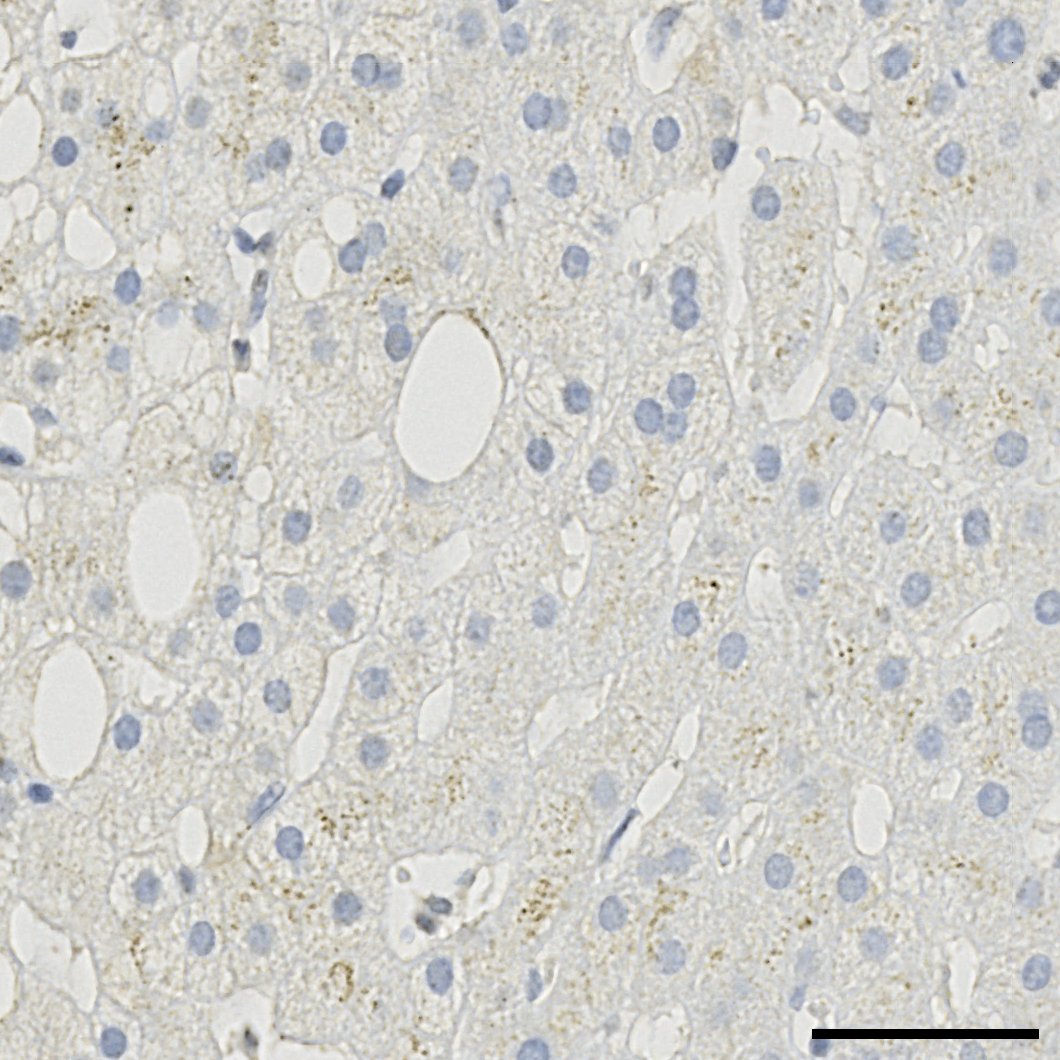

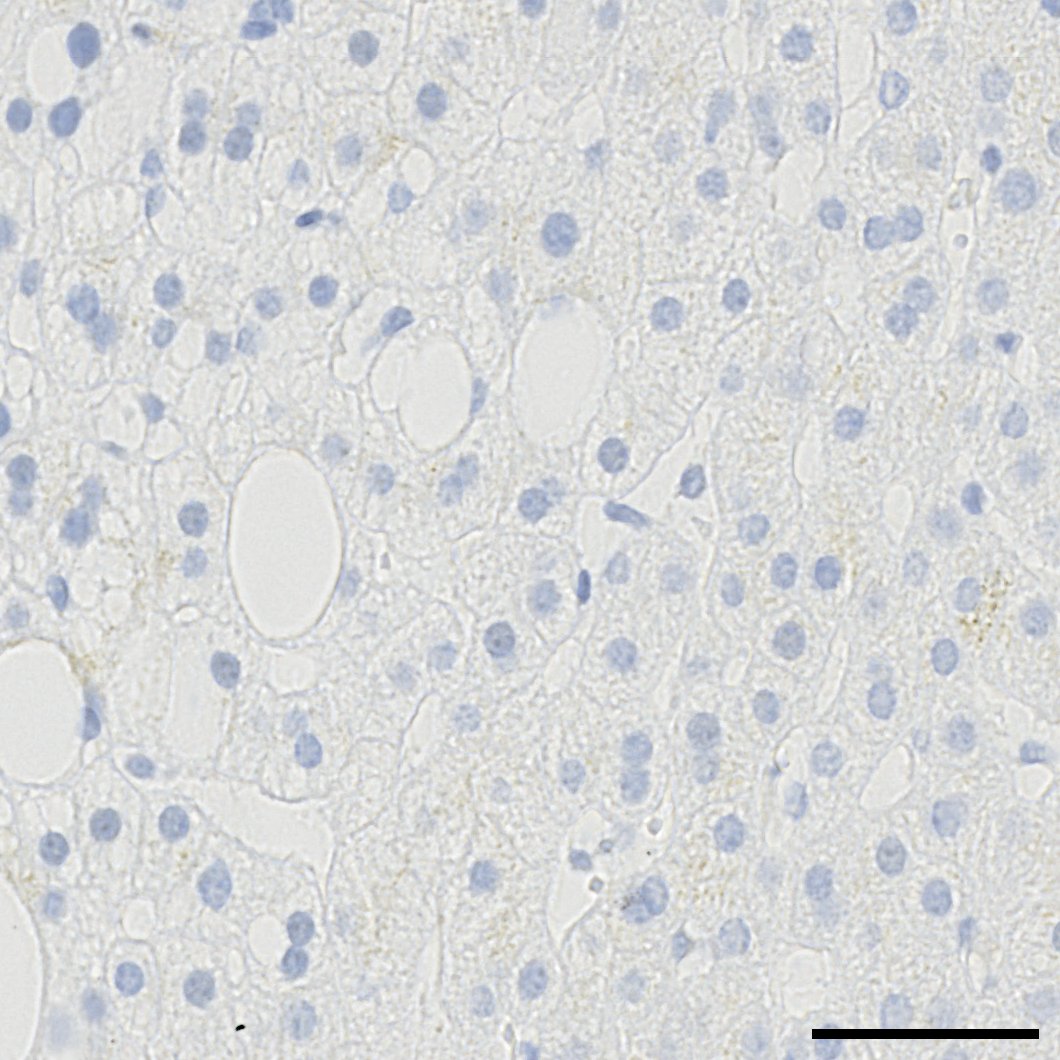


MASL


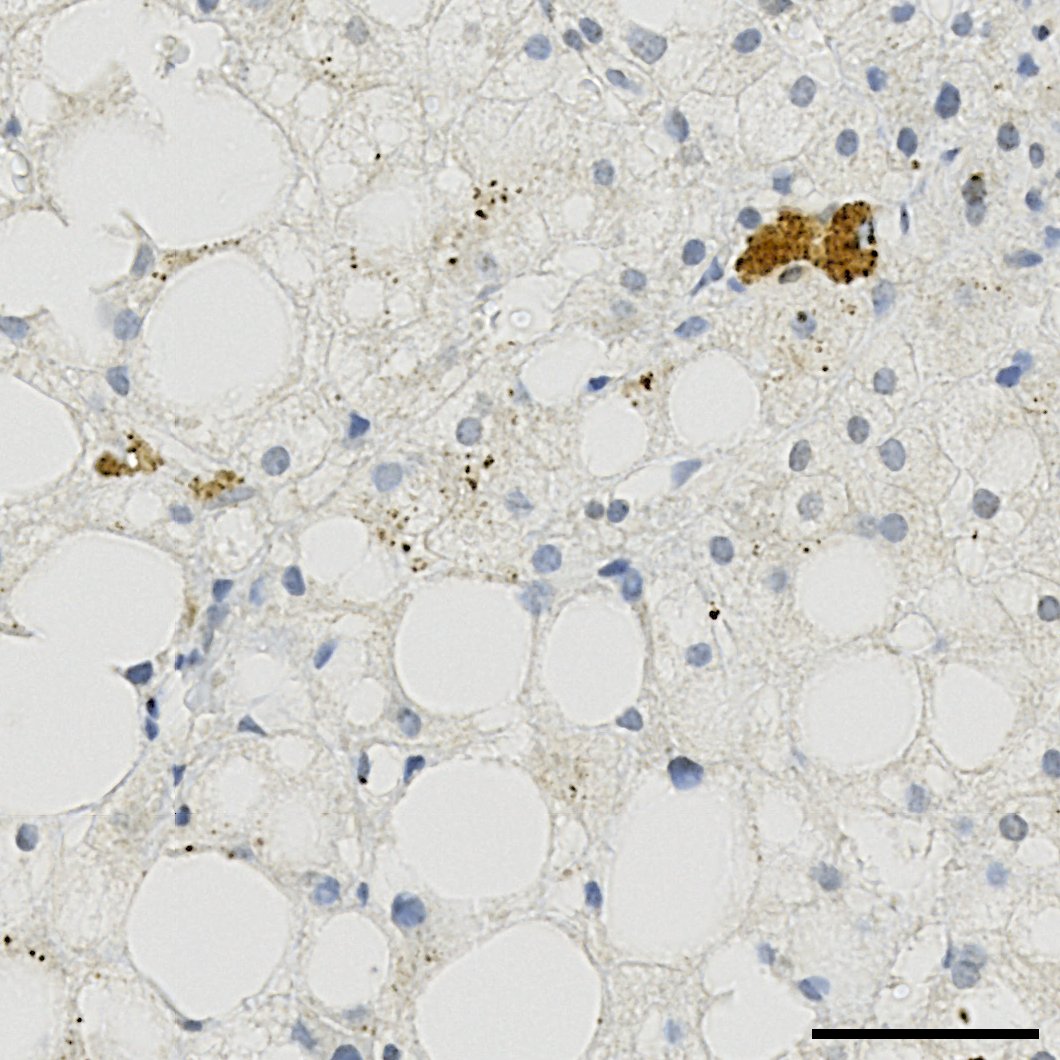

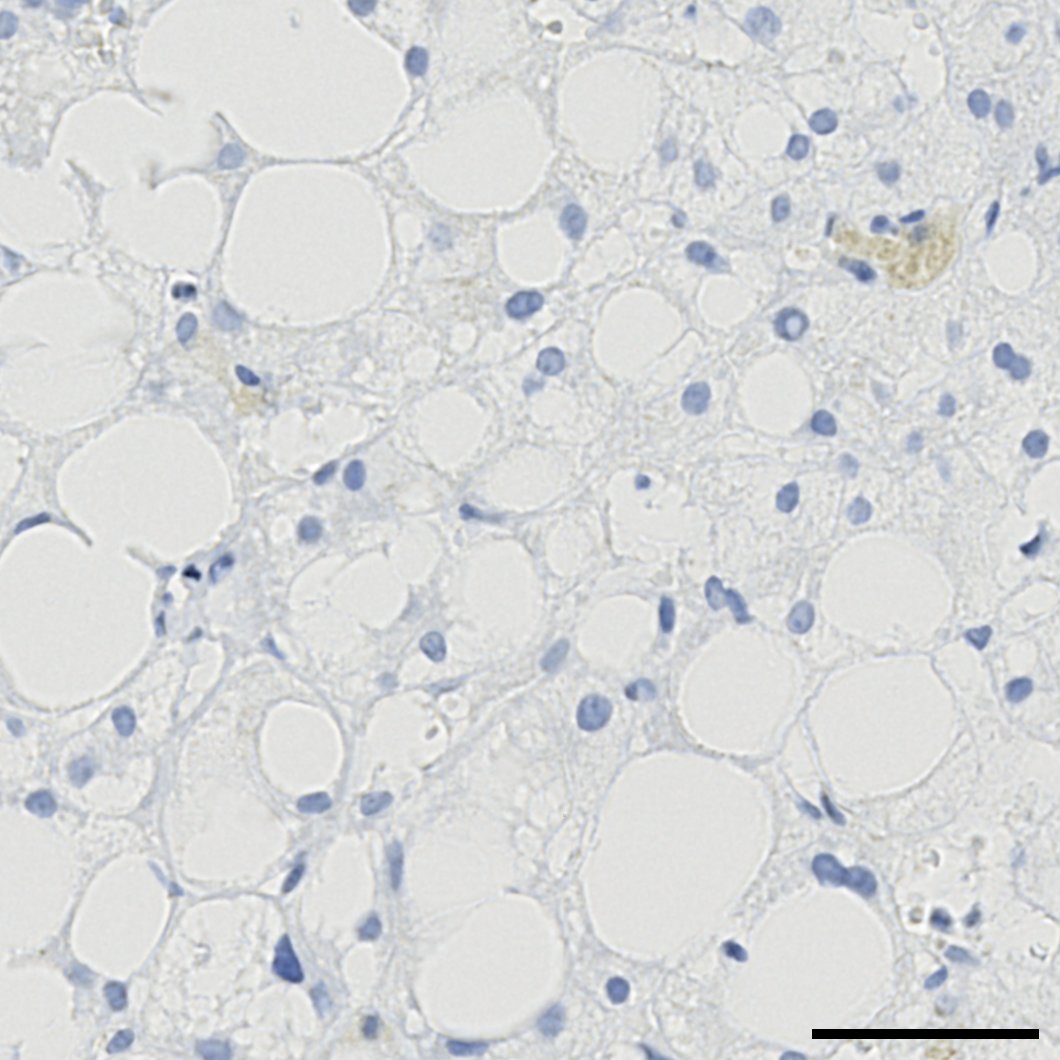


MASH F0-1


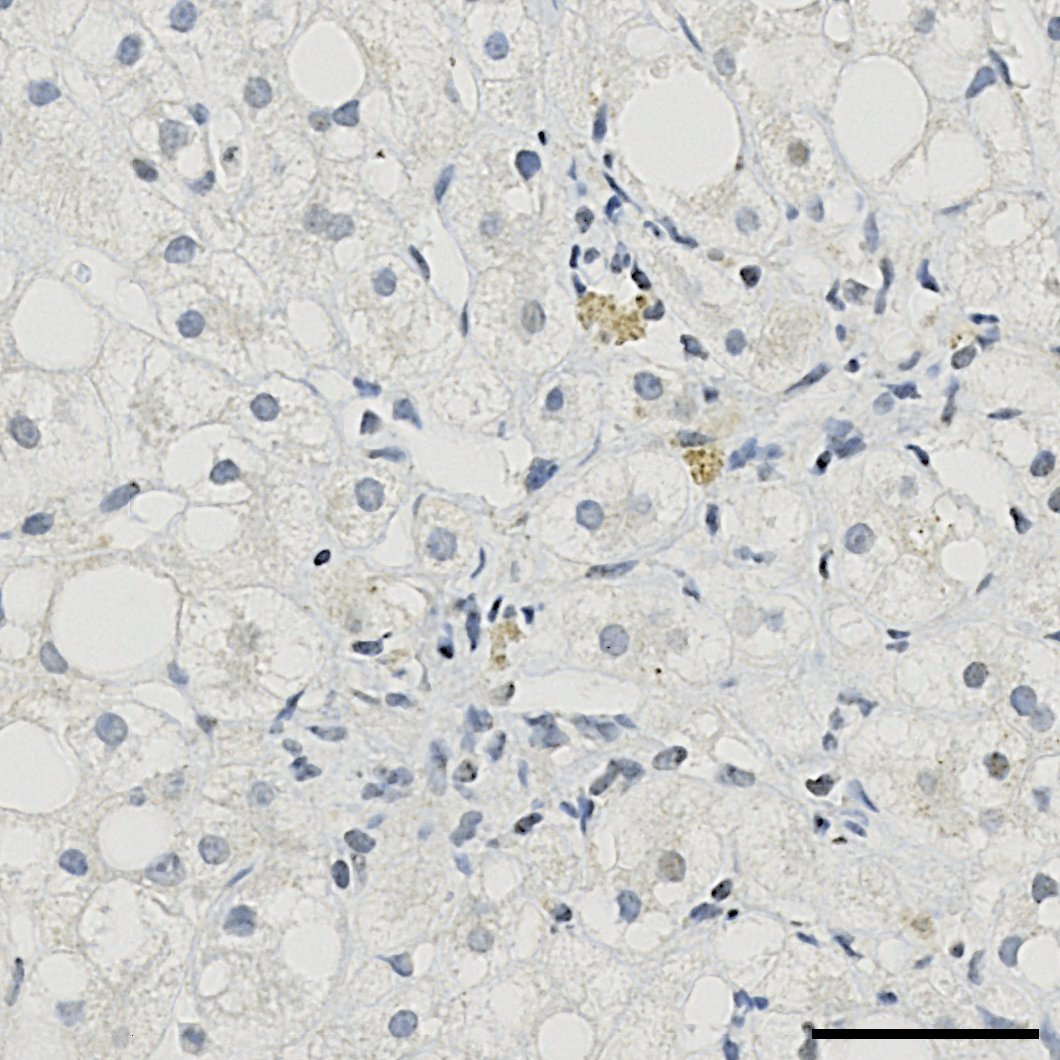

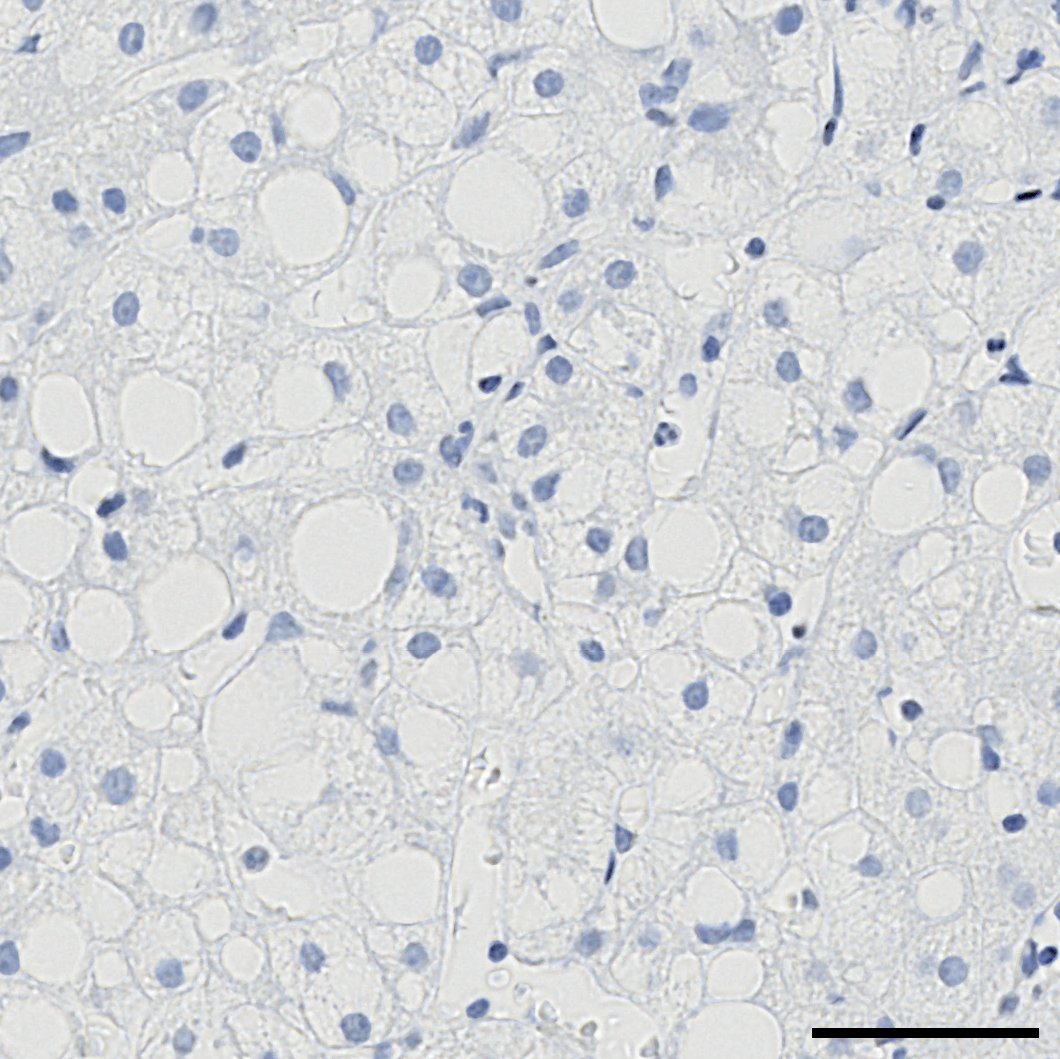


MASH F2-3

**Fig. S3. Immunohistochemistry for hepatic 4HNE in human liver biopsies and controls with omission of primary antibody HNEJ-1.**

Representative images from immunohistochemistry (IHC) for 4HNE, using the HNEJ-1 primary antibody, in patients with normal liver histology (Control), isolated steatosis (MASL), metabolic dysfunction-associated steatohepatitis without significant fibrosis (MASH F0-1) and metabolic dysfunction-associated steatohepatitis with significant fibrosis (MASH F2-3). Extracellular dense aggregates of 4HNE positivity were recognized as remnants of ferroptotic cell death (red arrows). 4HNE positive fine cytoplasmic granules in hepatocytes were considered lipofuscin (black arrow). Magnification 400x, scale bar 50µm.

Figure S4


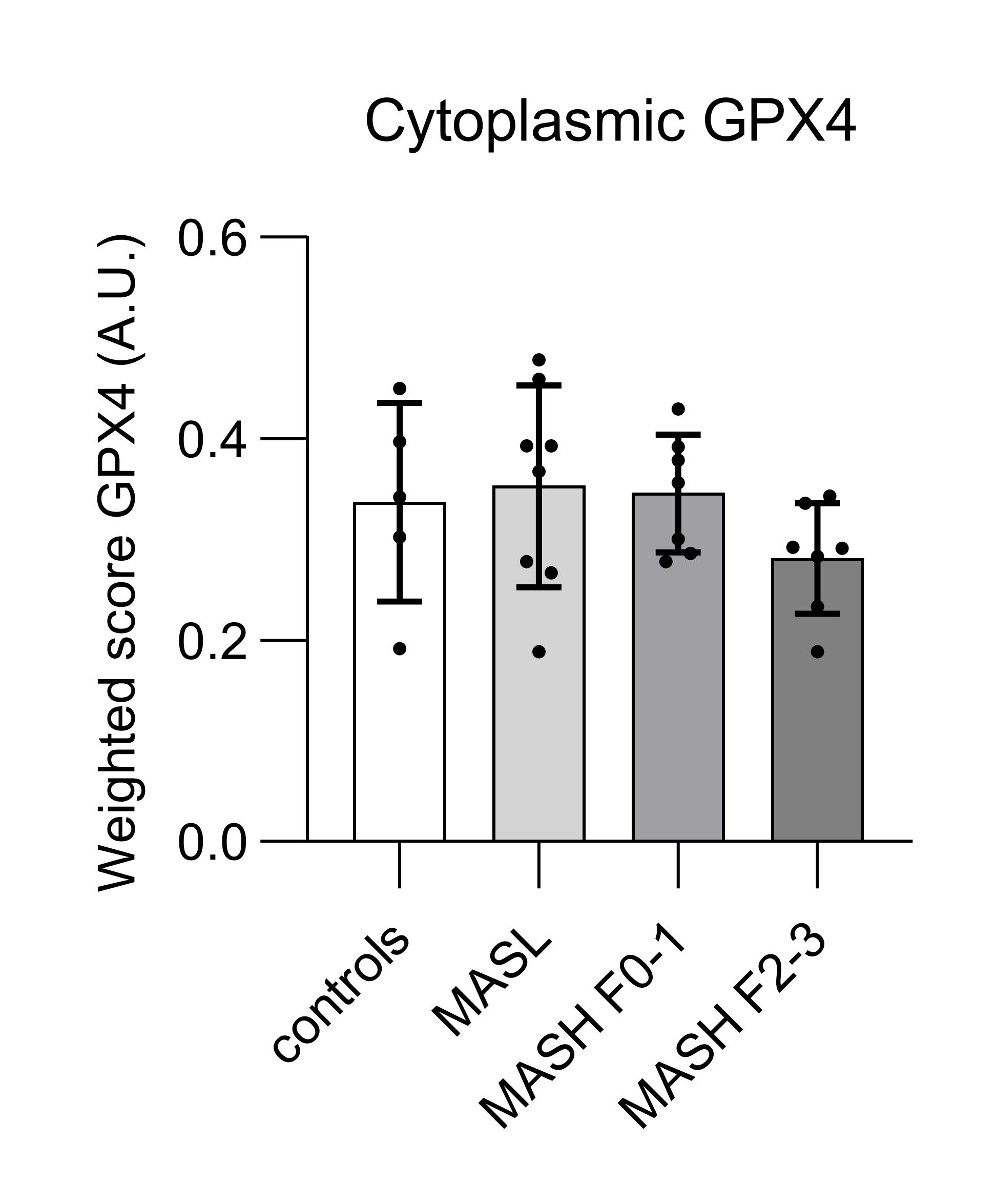

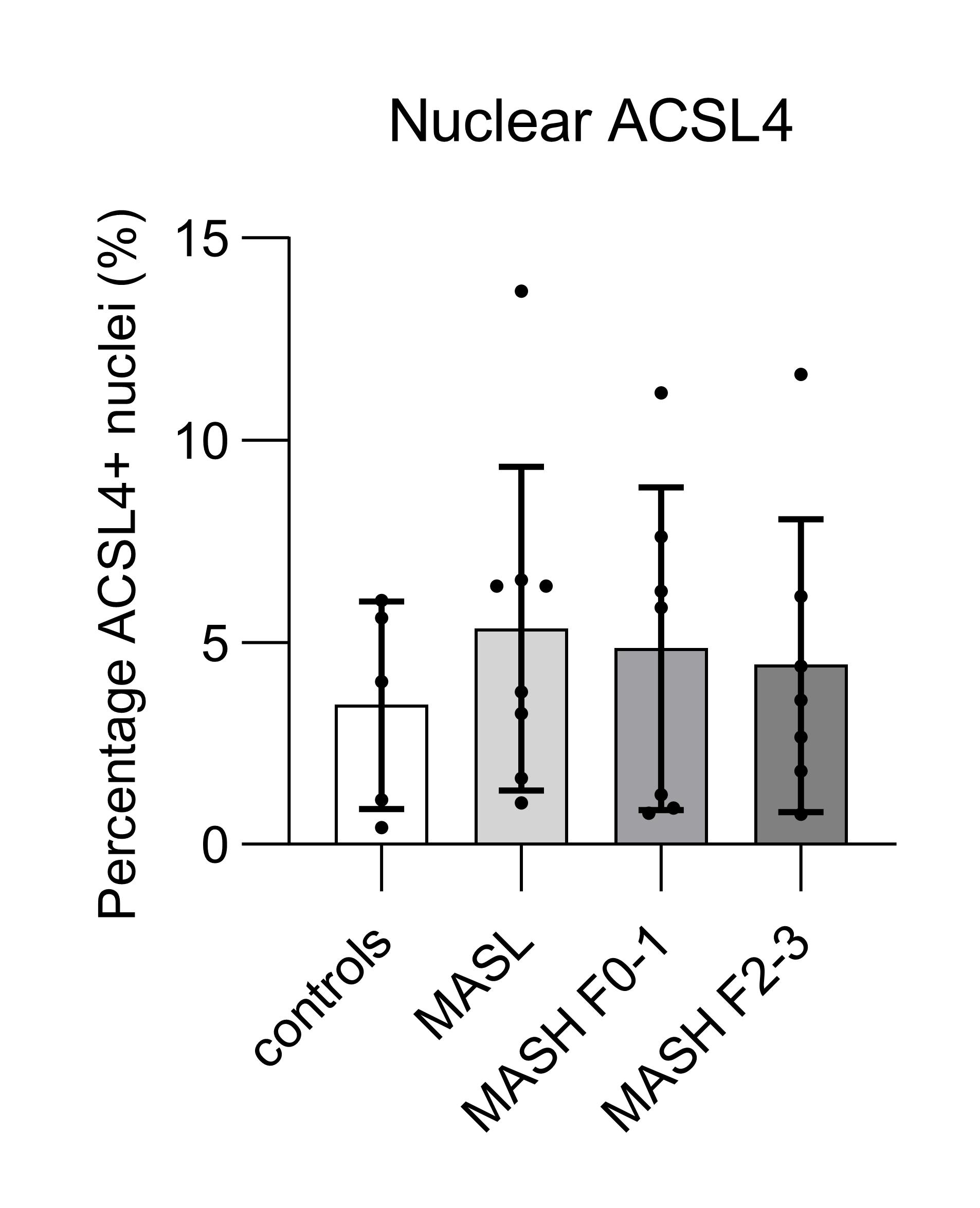


B

A

**Fig. S4. Quantification of nuclear GPX4 and cytoplasmic ACSL4 in human MASLD and controls.**

After reconstruction of hepatocyte in human needle liver biopsies with nuclei and accompanying cytoplasm, the presence of epitopes glutathione peroxidase 4 (GPX4) (A) and acyl-CoA synthetase long chain family member 4 (ACSL4) (B) was quantified in cytoplasm and nuclei, respectively. Data presented as mean ± standard deviation. Kruskal-Wallis test: p = 0.3118 and p = 0.7002 for cytoplasmic GPX4 and nuclear ACSL4, respectively.

Figure S5

A

Positive control Omission of Ferricyanide


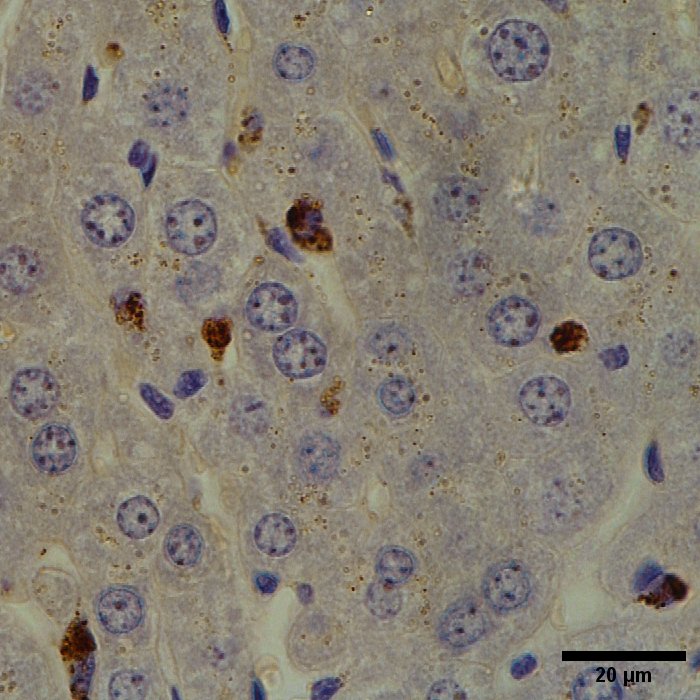

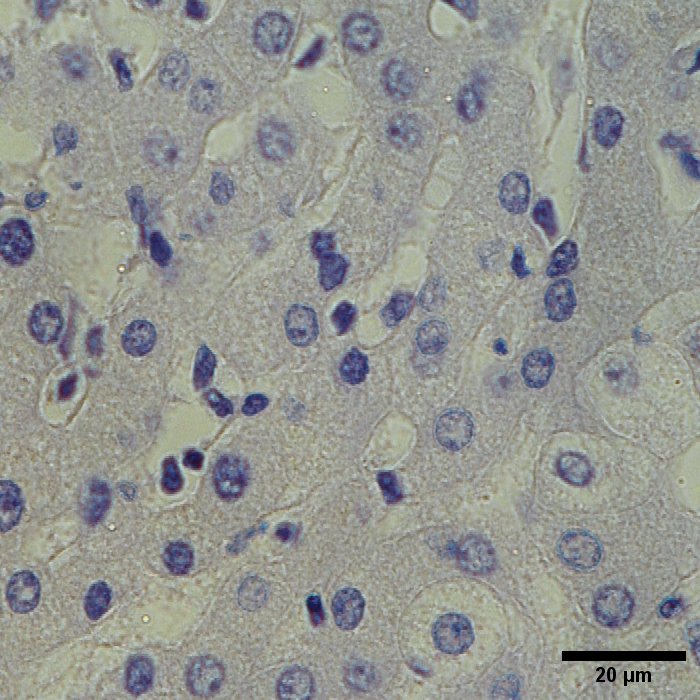


Control MASL


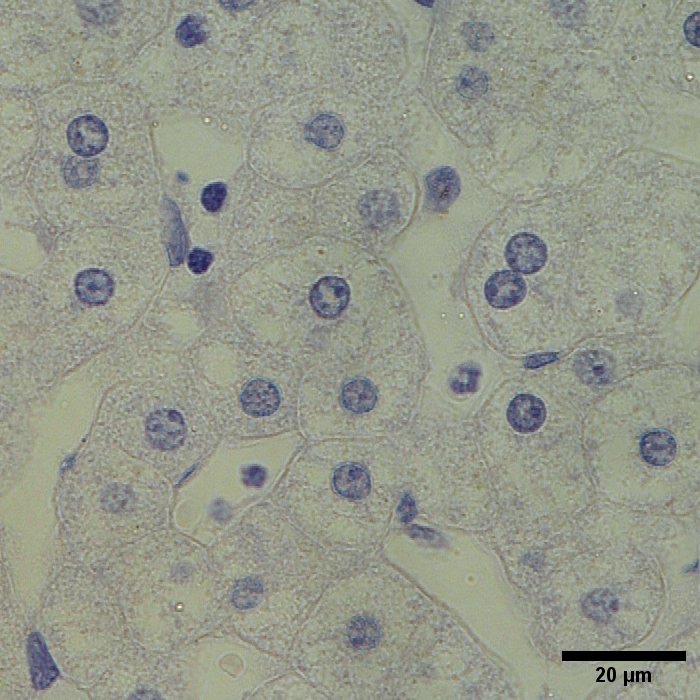

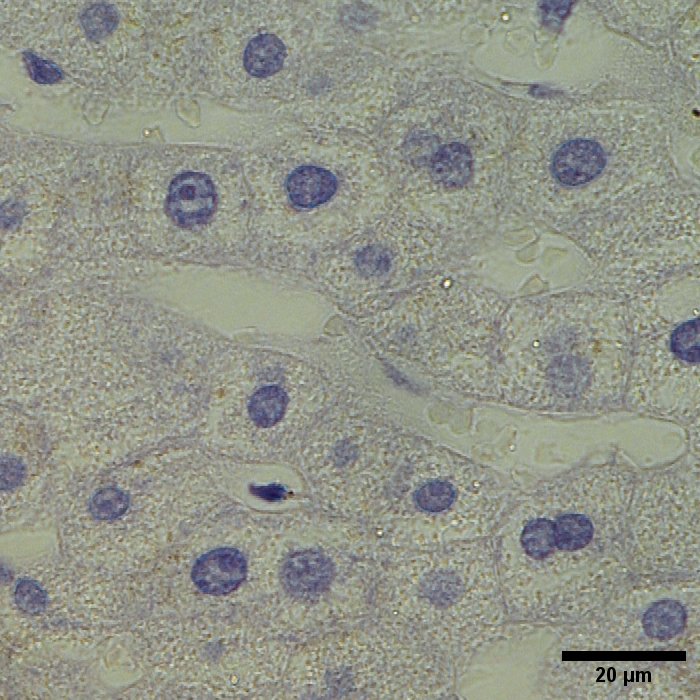


MASH F0-1 MASH F2-3


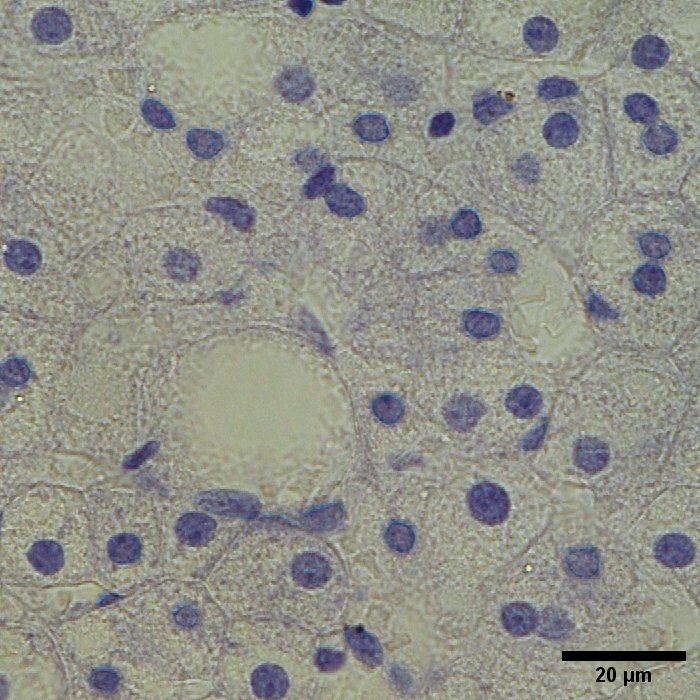

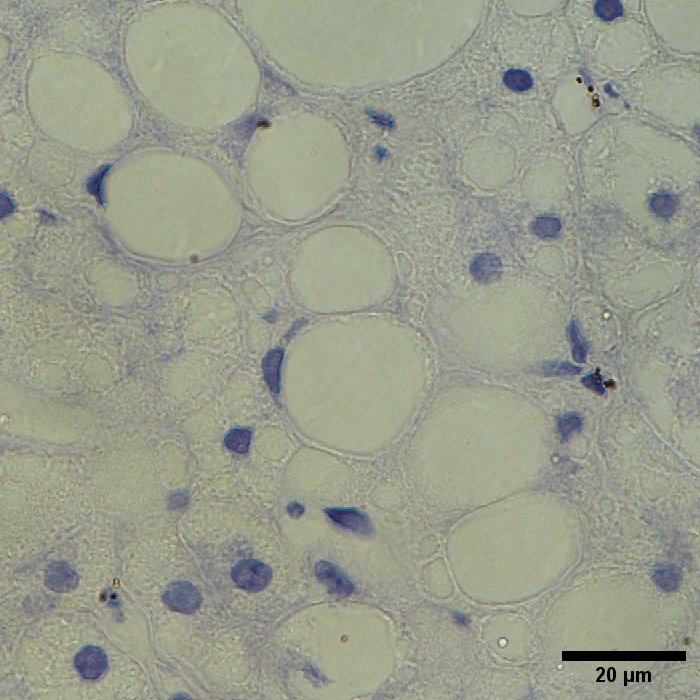


| Non-heme ferrous iron | | |
| --- | --- | --- |
|  | No | Yes |
| Control | 5 (100) | 0 (0) |
| MASL | 5 (71.4) | 2 (28.6) |
| MASH F0-1 | 3 (42.9) | 4 (57.1) |
| MASH F2-3 | 5 (62.5) | 3 (37.5) |

B

**Fig. S5. A subset of human MASLD patients displays aggregates of sinusoidal ferrous iron in liver biopsy specimens.**

(A) Representative images from non-heme ferrous iron stain in liver needle biopsies from controls and different histologic groups of MASLD patients using the method described by Turnbull. Red arrows point towards 3,3′-Diaminobenzidine positive granules of ferrous iron, located in sinusoidal cells. The positive control consists of liver tissue derived from a mouse that received an intraperitoneal injection of iron(II)sulphate heptahydrate (300mg/kg bodyweight). Scale bar 20µm, magnification 400x. (B) Scoring of non-heme ferrous iron stain as negative or positive for the presence of 3,3′-Diaminobenzidine positive granules in sinusoidal cells per histologic group.

Figure S6


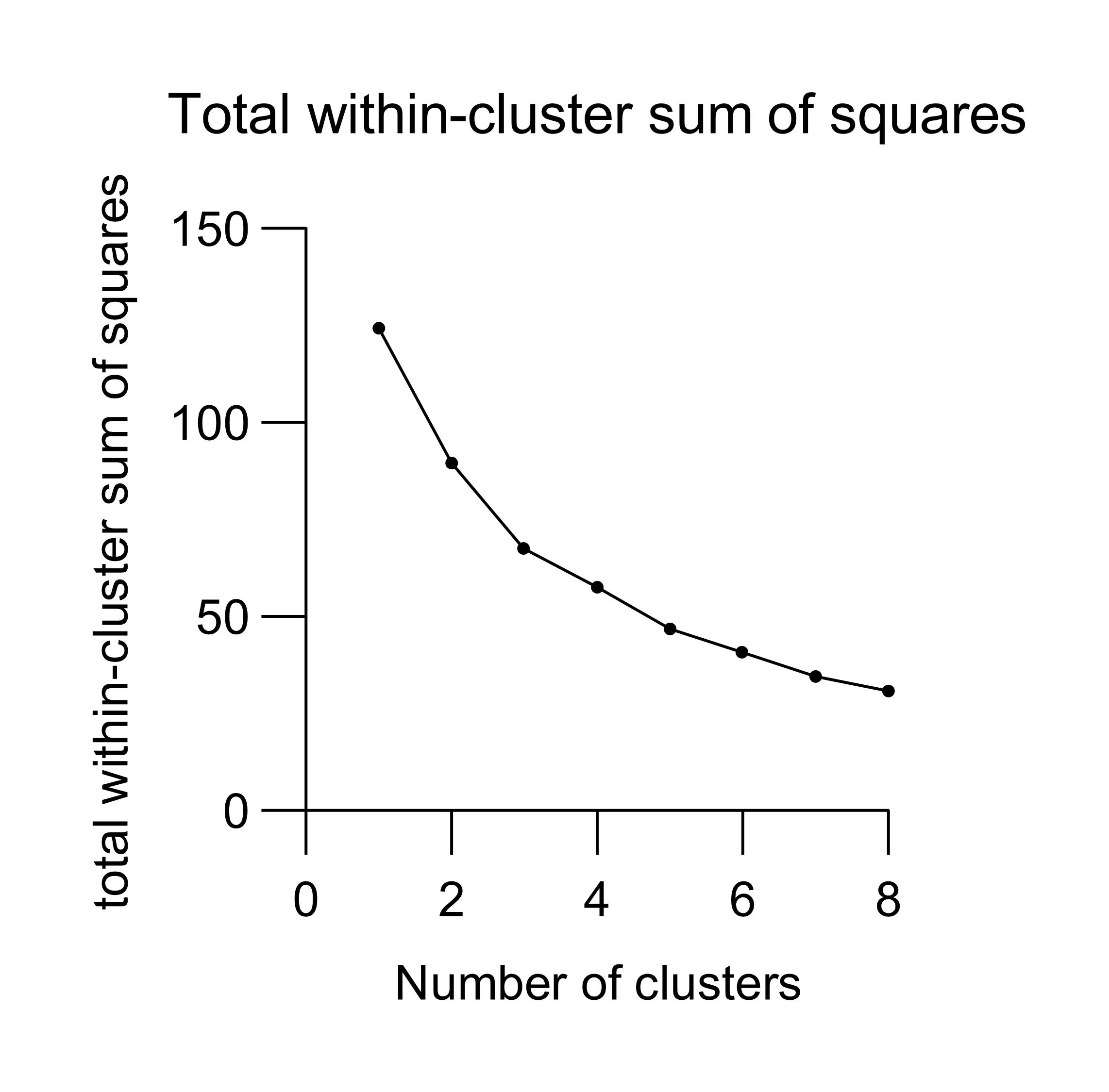


**Fig. S6. Elbow method on the total within-cluster sum of squares metric for unsupervised clustering in human MASLD and controls.**

The optimal number of clusters for the k-prototypes partitioning clustering based on hepatic ferroptosis markers in liver biopsies from human MASLD patients and controls was determined using the total within-cluster sum of squares metric. The latter is calculated for every number of clusters and lower values thereof indicate greater similarity of samples within a cluster. Increasing the number of clusters improves the similarity of samples within one cluster, but only to a given point.

Figure S7


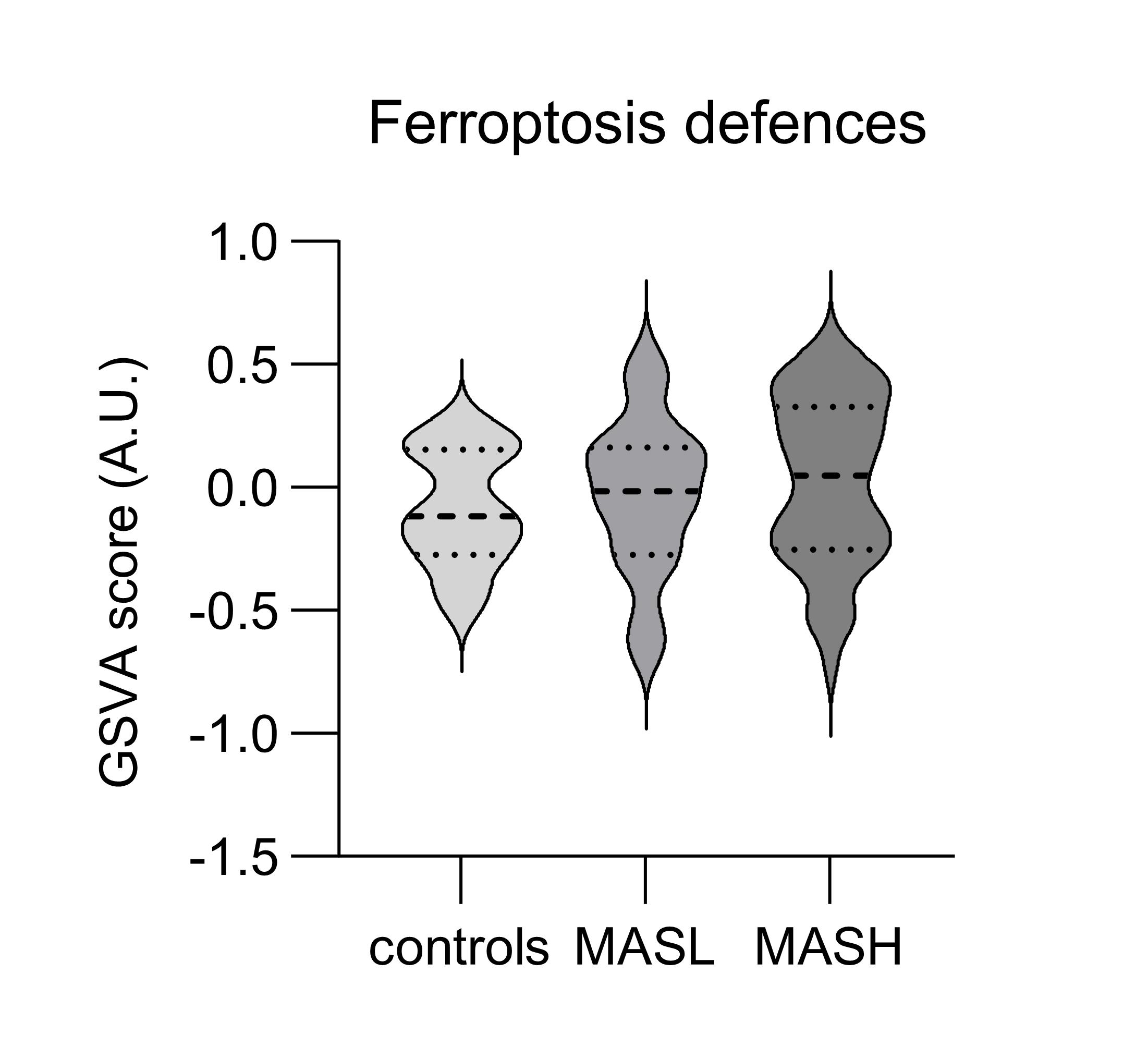

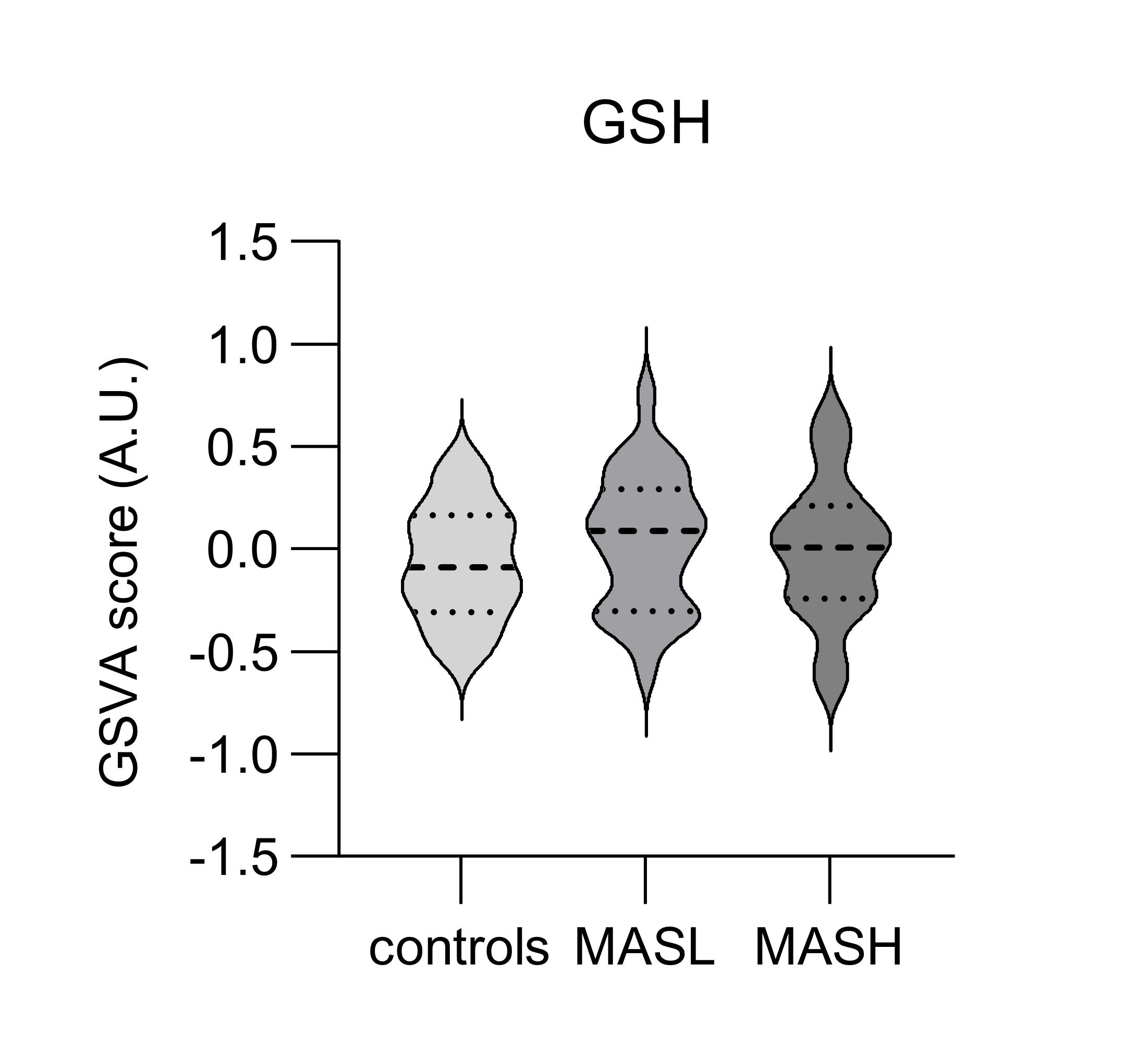

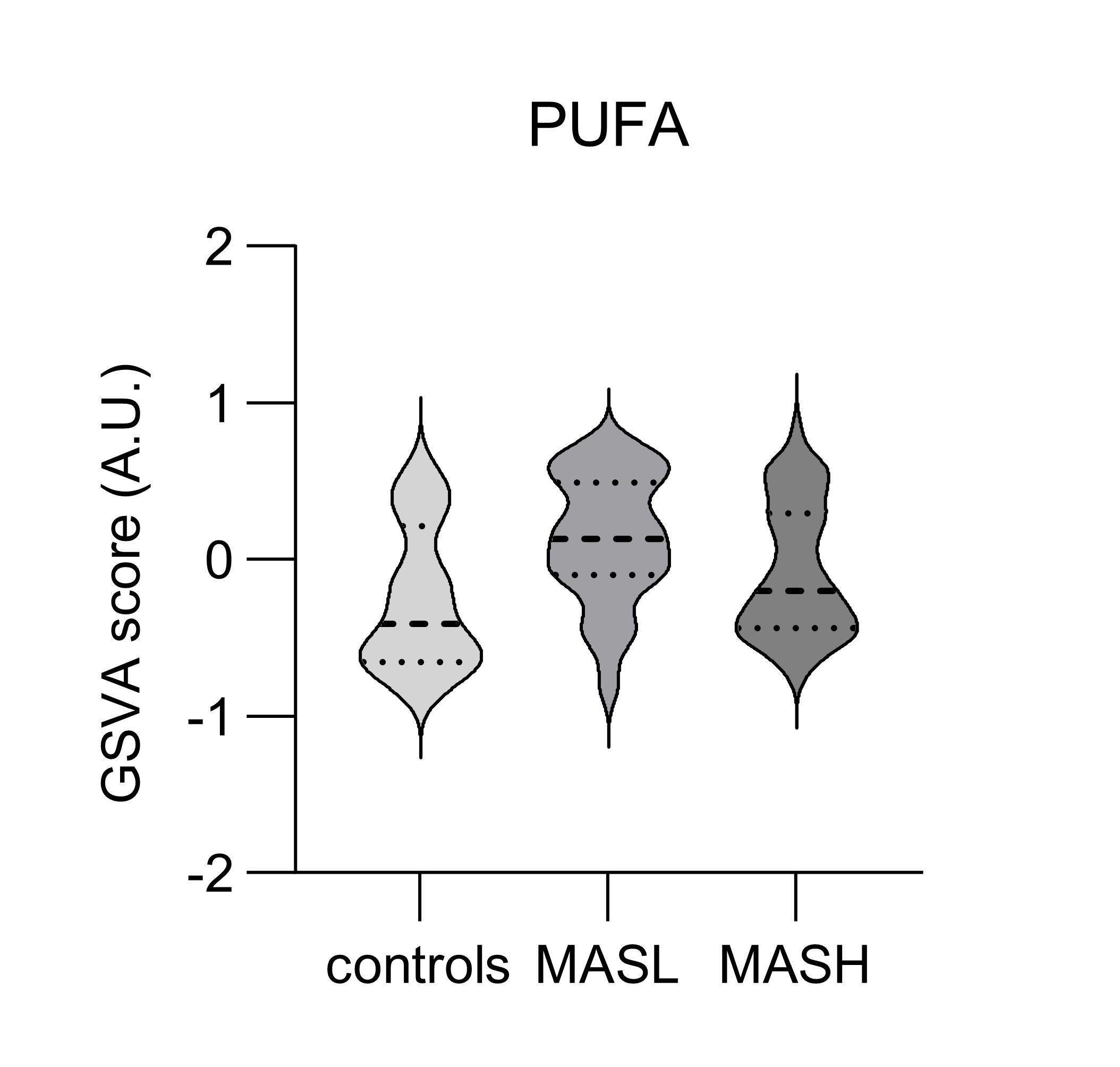

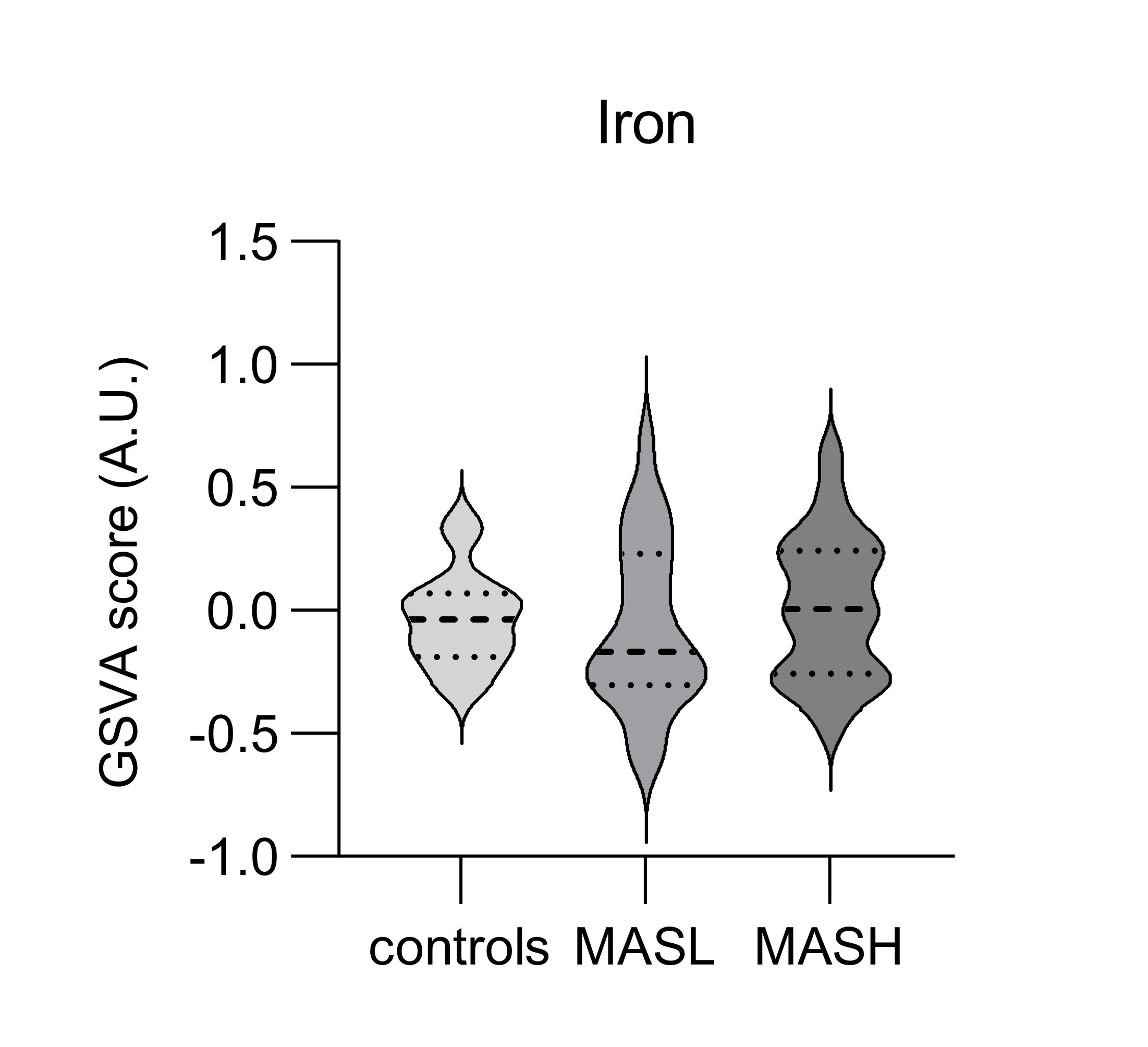


**Fig. S7. Gene set variation analysis for 4 ferroptosis-related gene sets in publicly available database GSE130970 according to liver histology**

The gene set variation analysis (GSVA) score per gene set, *i.e.* ‘ferroptosis defenses’, ‘GSH’, ‘PUFA’ and ‘Iron’, was calculated in every MASLD patient and control of transcriptomics database GSE130970 (n = 78), followed by plotting per histologic class. Violin plots show median and quartiles. One-way ANOVA test was performed.

Figure S8


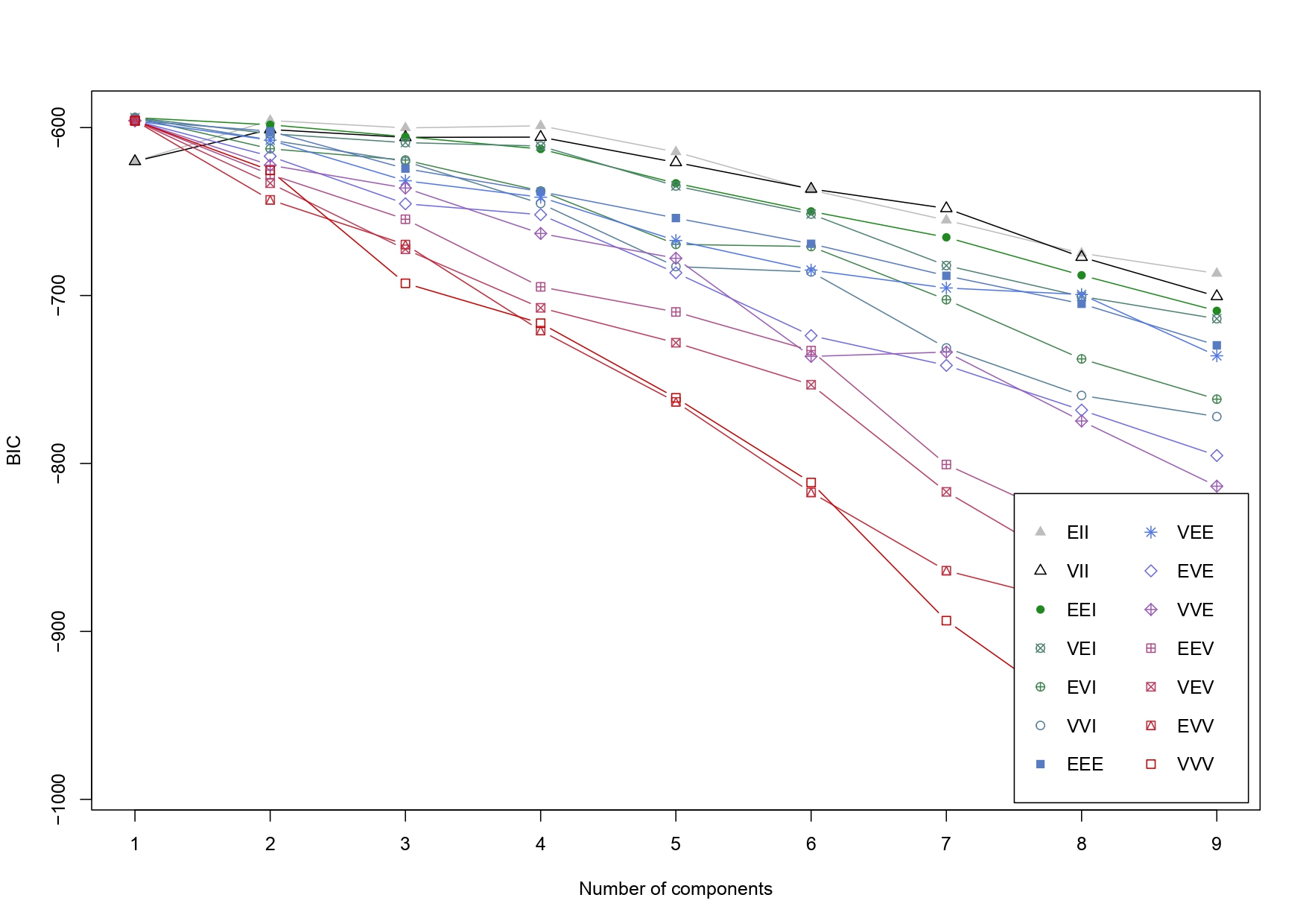

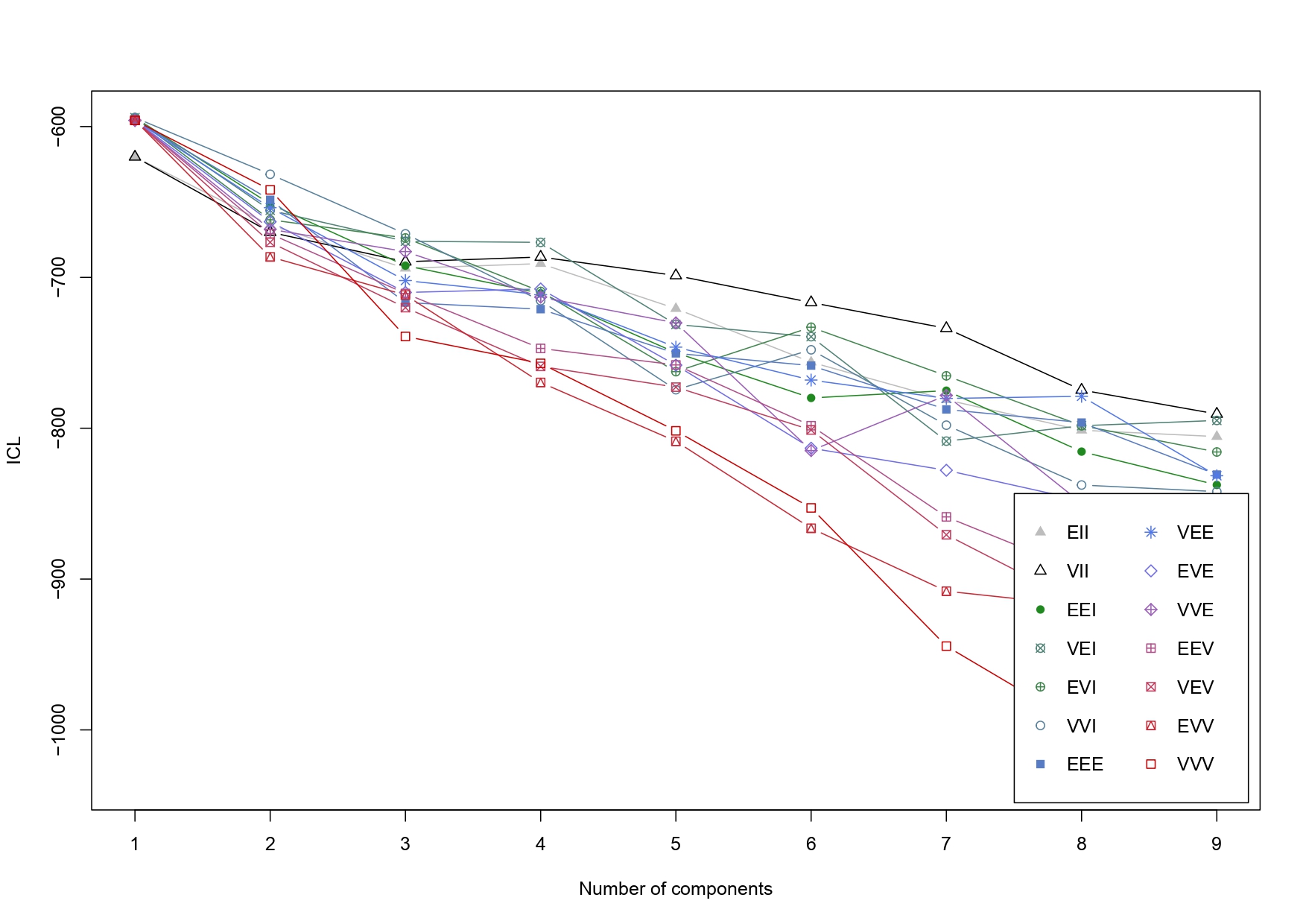

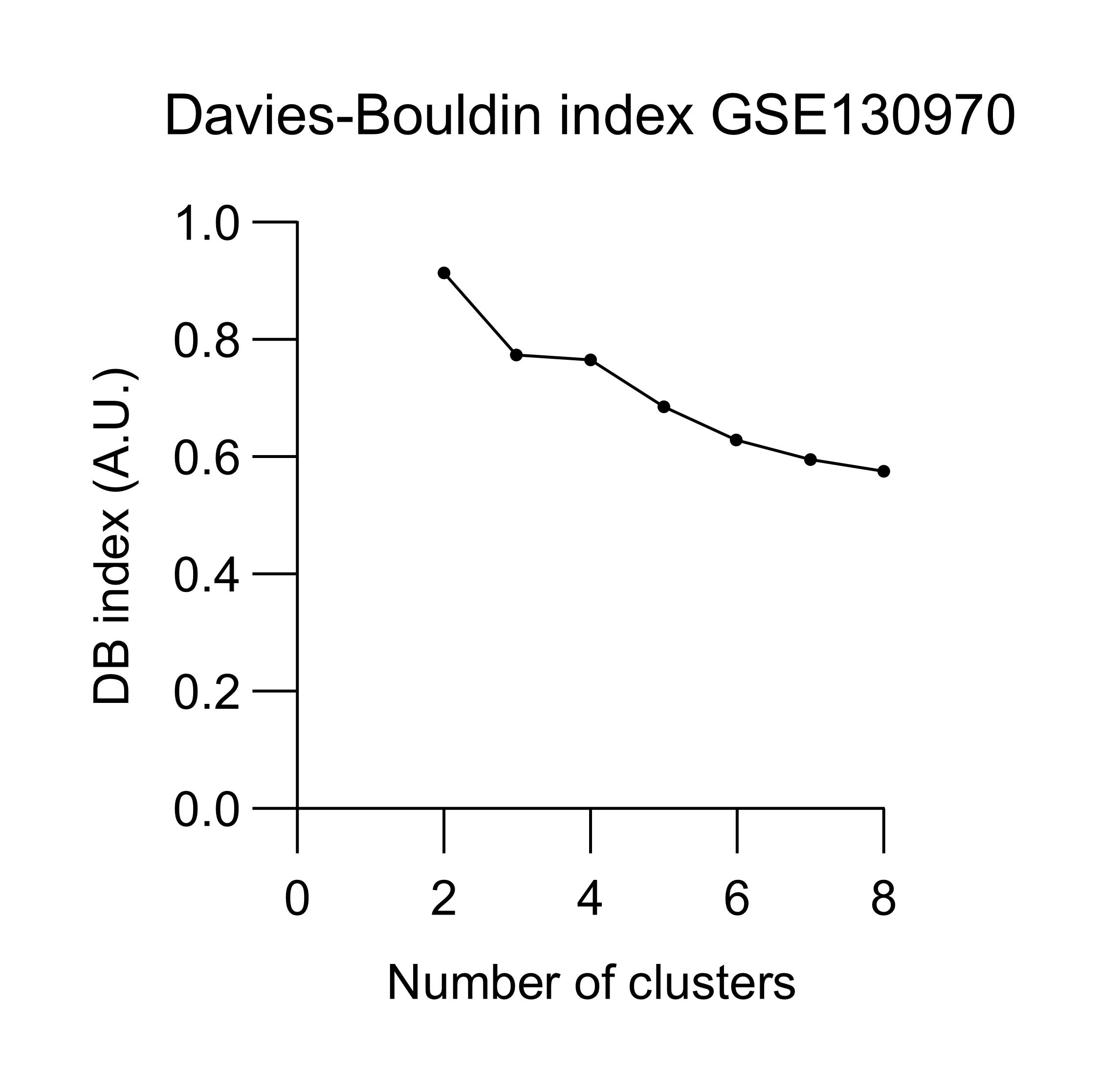


A

C

B


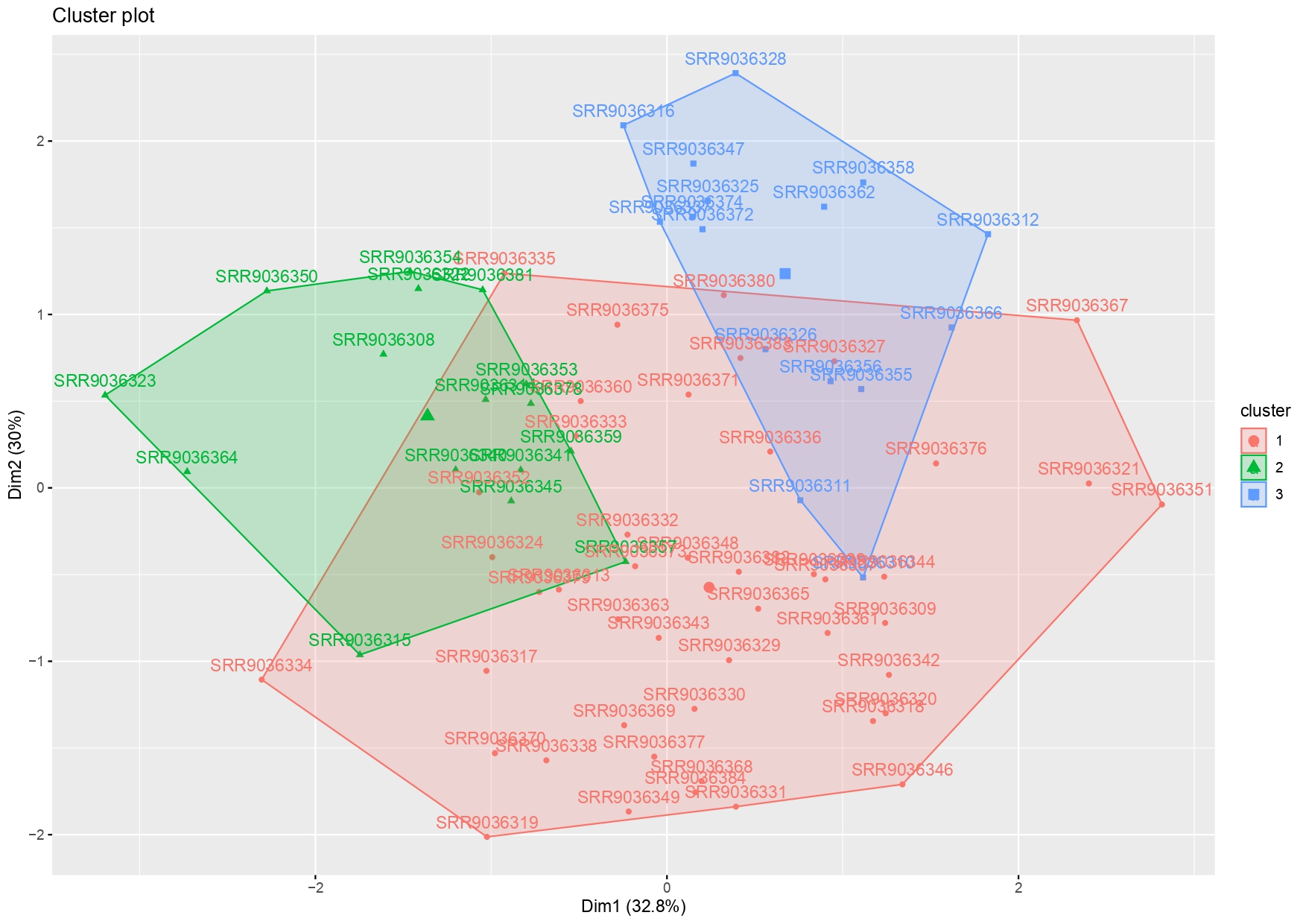


D

**Fig. S8. Unsupervised clustering of MASLD patients and controls from GSE130970 based on ferroptosis-related gene sets by Gaussian Mixture models.**

(A) Values of Bayesian Information Criterion (BIC) were plotted for a given number of clusters by each model of Gaussian Mixture modelling. (B) Likewise, values of the Integrated Complete-data Likelihood (ICL) were plotted per number of clusters by each fitted Gaussian Mixture model. (C) Values of the Davies-Bouldin’s index were plotted for any given number of clusters defined by Gaussian Mixture modelling to perform the elbow method. Lower levels of this index indicate a better separation between the clusters. (D) Principal component analysis (PCA) plot was used to visualize the 3 clusters of MASLD patients and controls constructed by the EII model (spherical distribution, equal volume, equal shape) of Gaussian mixture modelling.

Figure S9


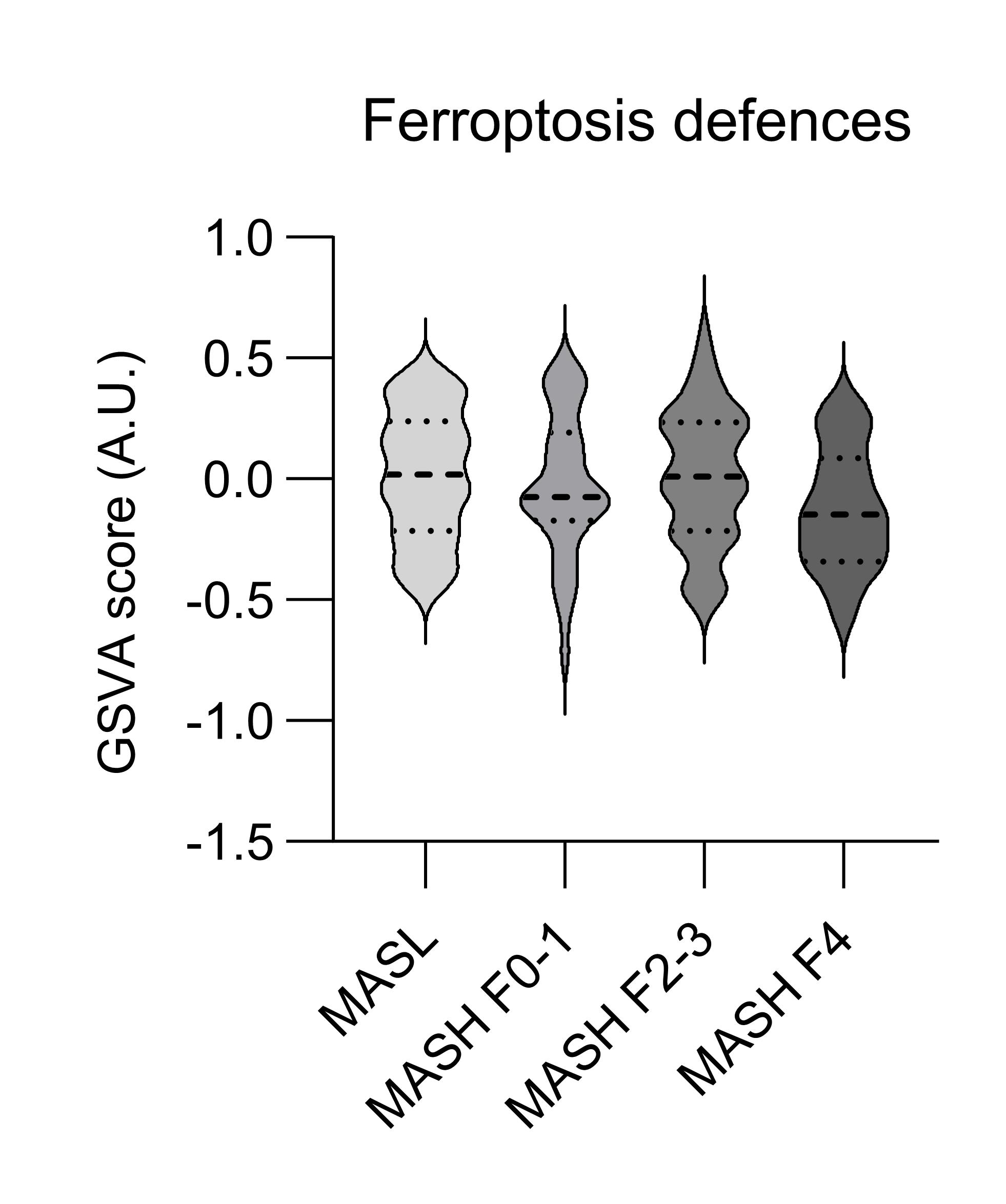

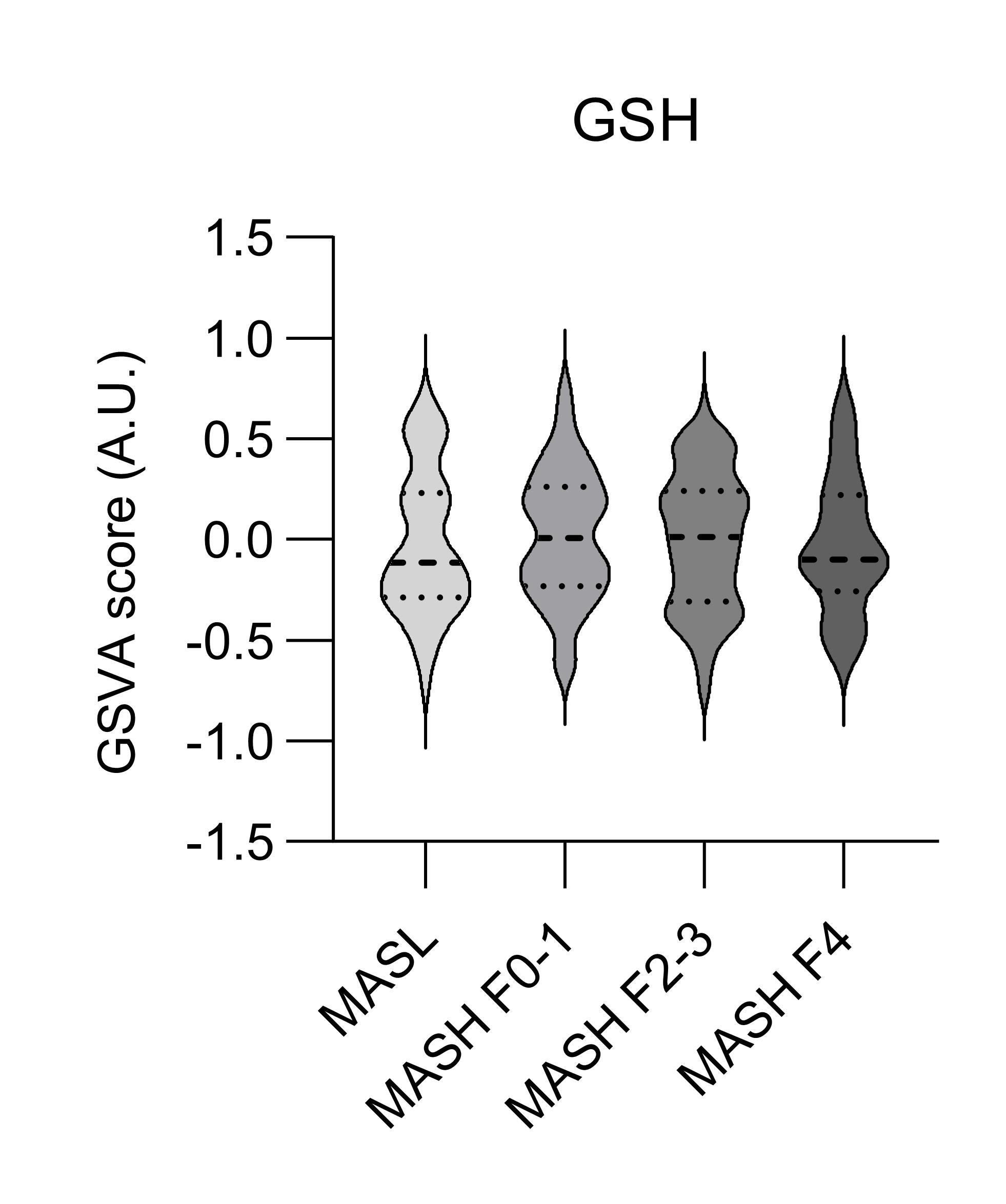

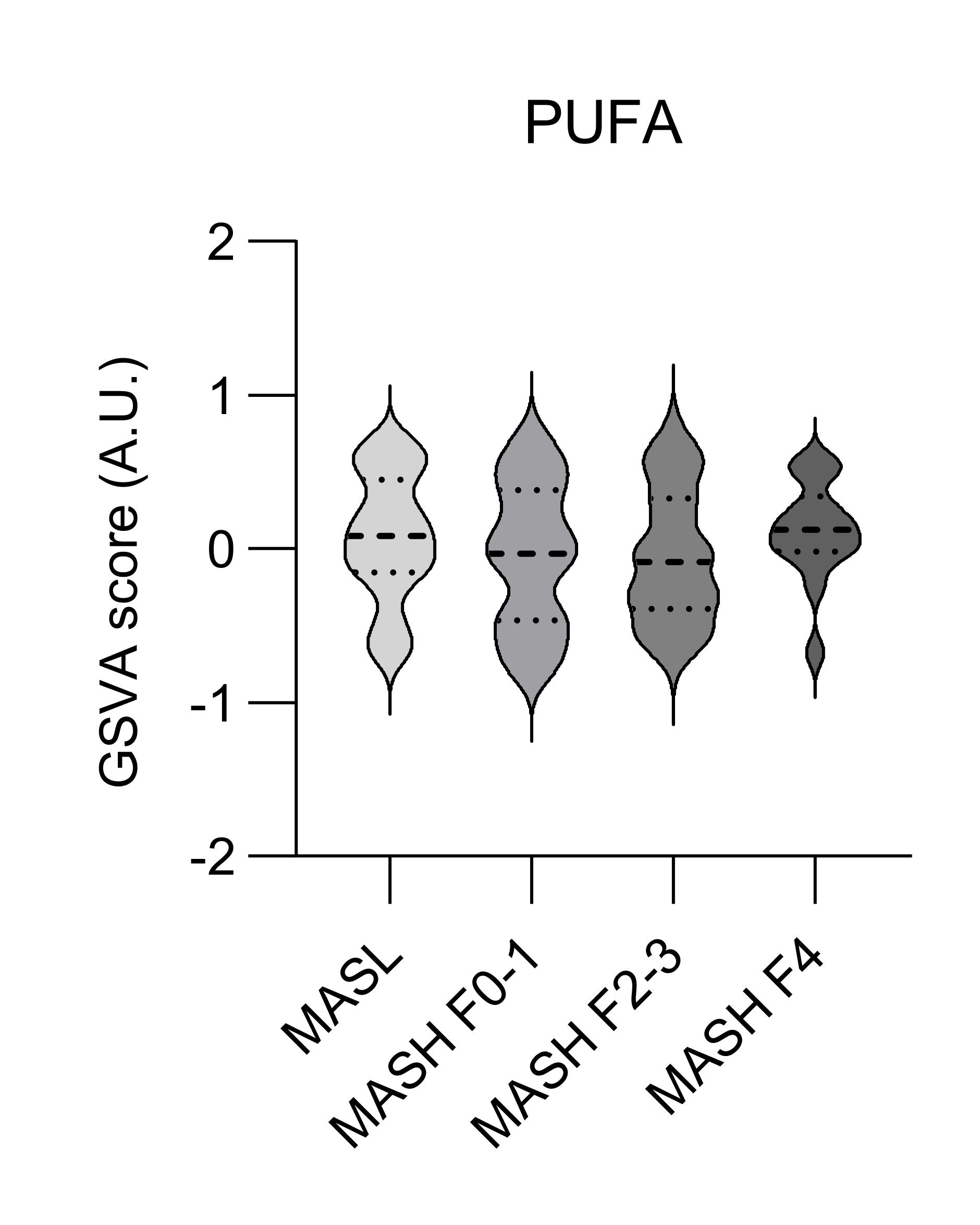

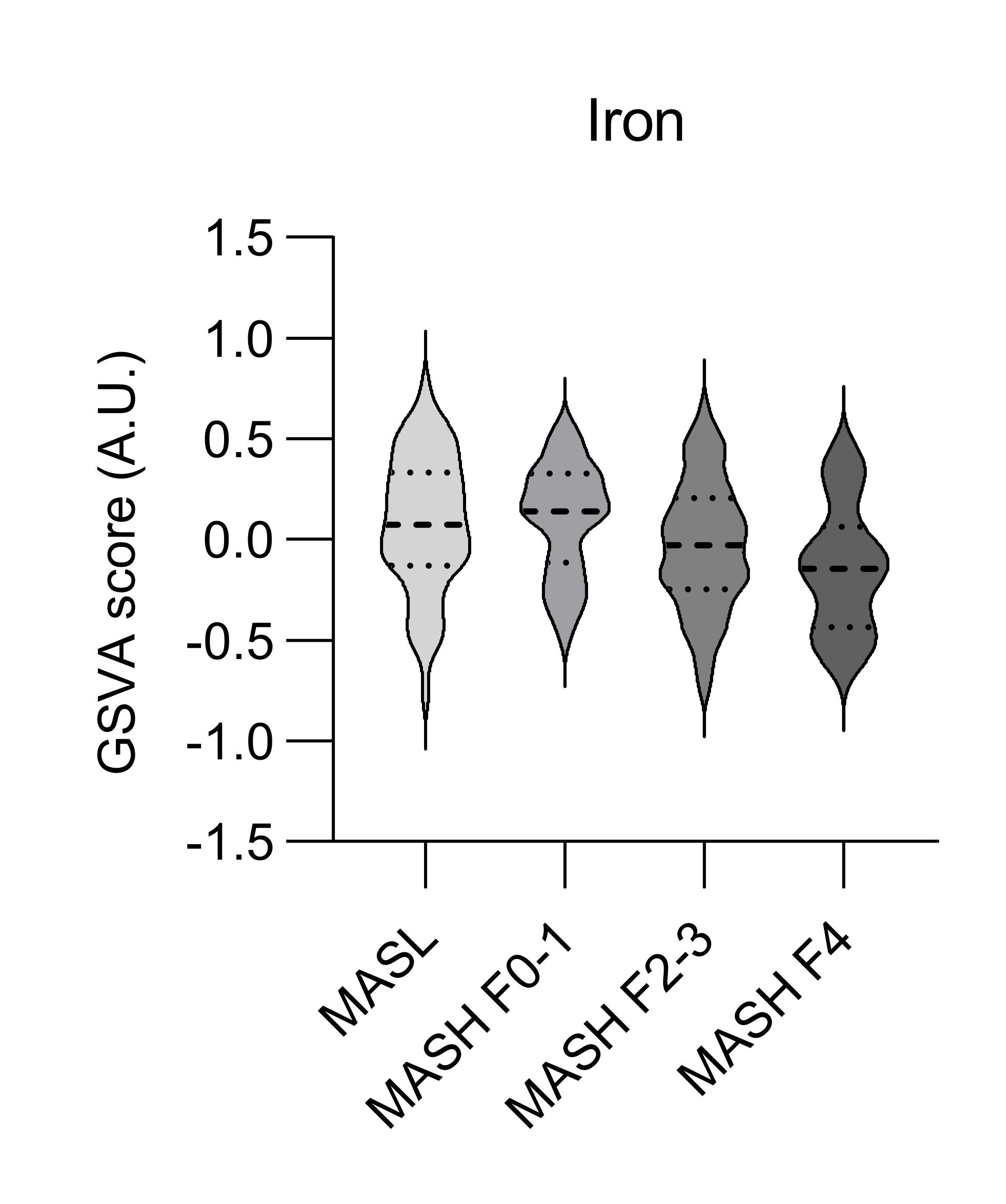


**Fig. S9. Gene set variation analysis for 4 ferroptosis-related gene sets in publicly available database GSE135251 according to liver histology**

The gene set variation analysis (GSVA) score per gene set, *i.e.* ‘ferroptosis defenses’, ‘GSH’, ‘PUFA’ and ‘Iron’, was calculated in every MASLD patient and control of transcriptomics database GSE 135251 (n = 206), followed by plotting per histologic class. Violin plots show median and quartiles. One-way ANOVA test was performed.

Figure S10


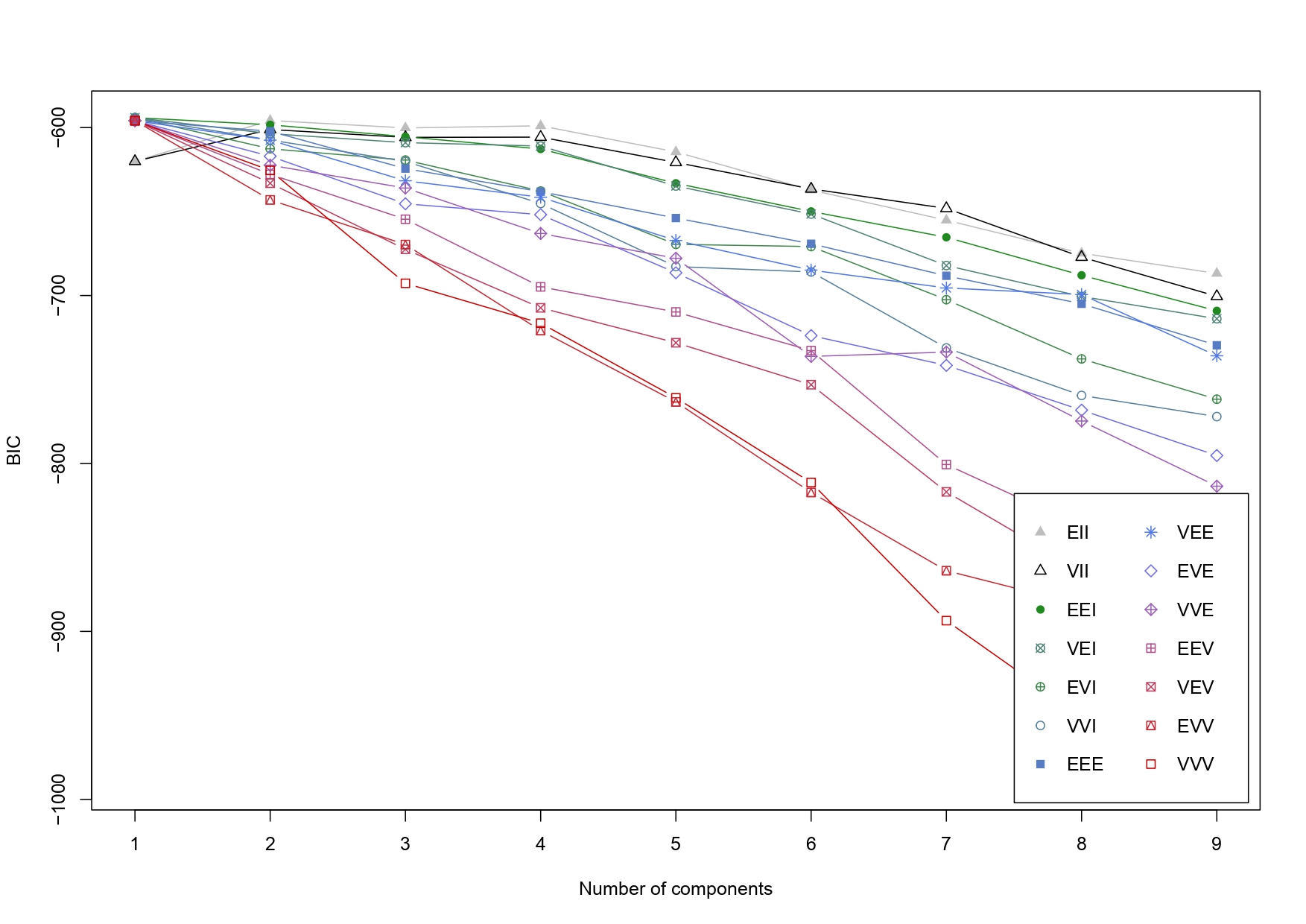


B

A


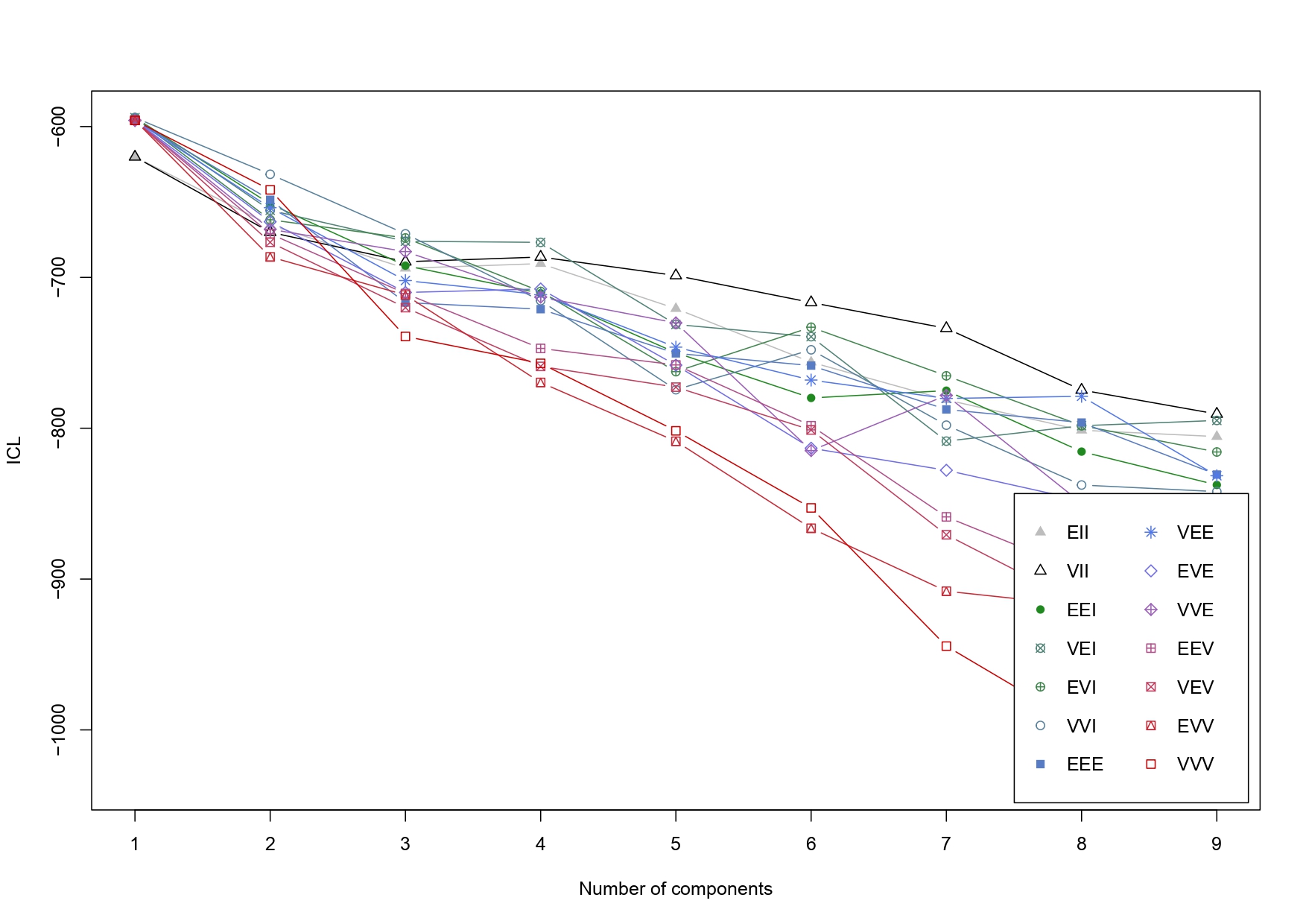

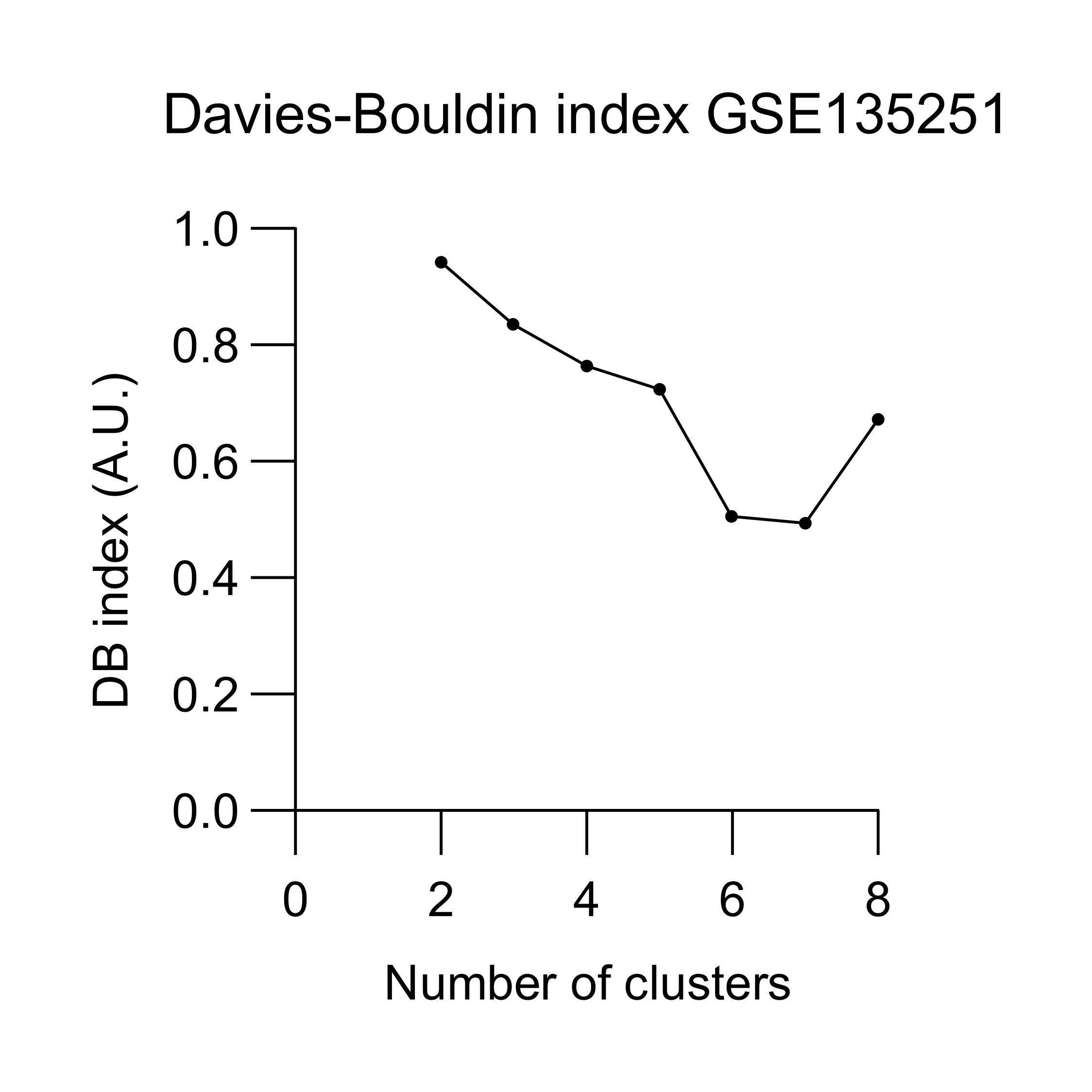


C

D


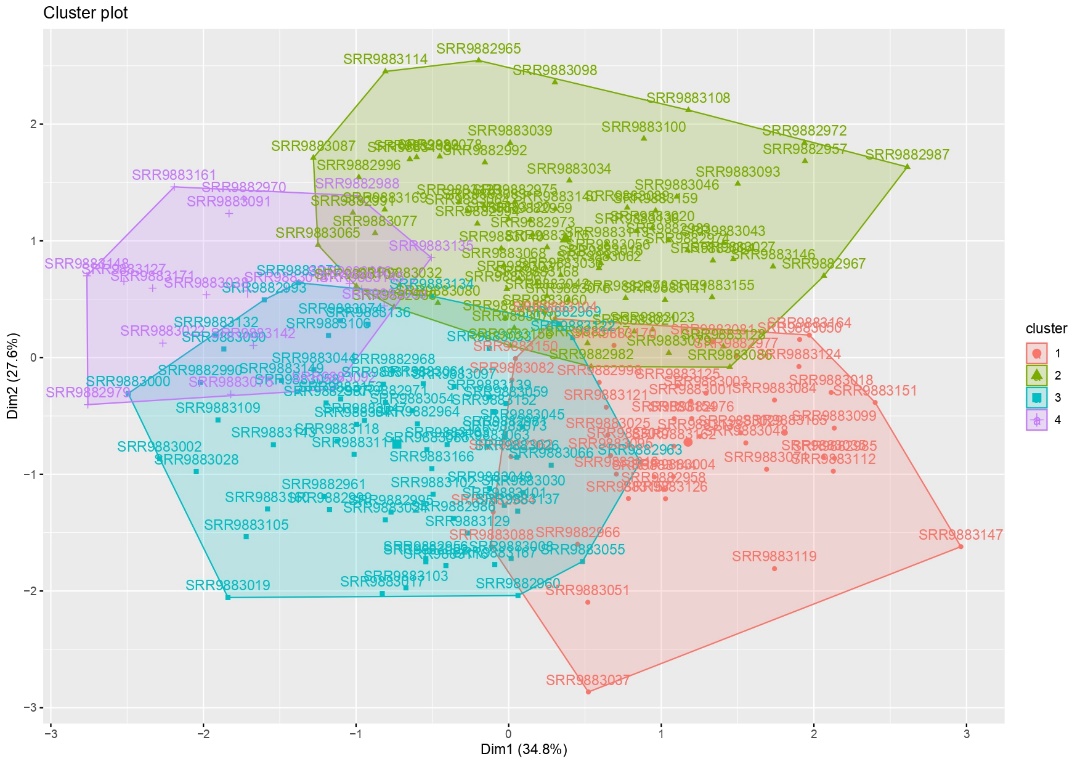


**Fig. S10.** **Unsupervised clustering of MASLD patients from GSE135251 based on ferroptosis-related gene sets by Gaussian Mixture models.**

(A) Values of Bayesian Information Criterion (BIC) were plotted for a given number of clusters by each model of Gaussian Mixture modelling. (B) Likewise, values of the Integrated Complete-data Likelihood (ICL) were plotted per number of clusters by each fitted Gaussian Mixture model. (C) Values of the Davies-Bouldin’s index were plotted for any given number of clusters defined by Gaussian Mixture modelling to perform the elbow method. Lower levels of this index indicate a better separation between the clusters. (D) Principal component analysis (PCA) plot was used to visualize the 4 clusters of MASLD patients constructed by the EII model (spherical distribution, equal volume, equal shape) of Gaussian mixture modelling.

Figure S11


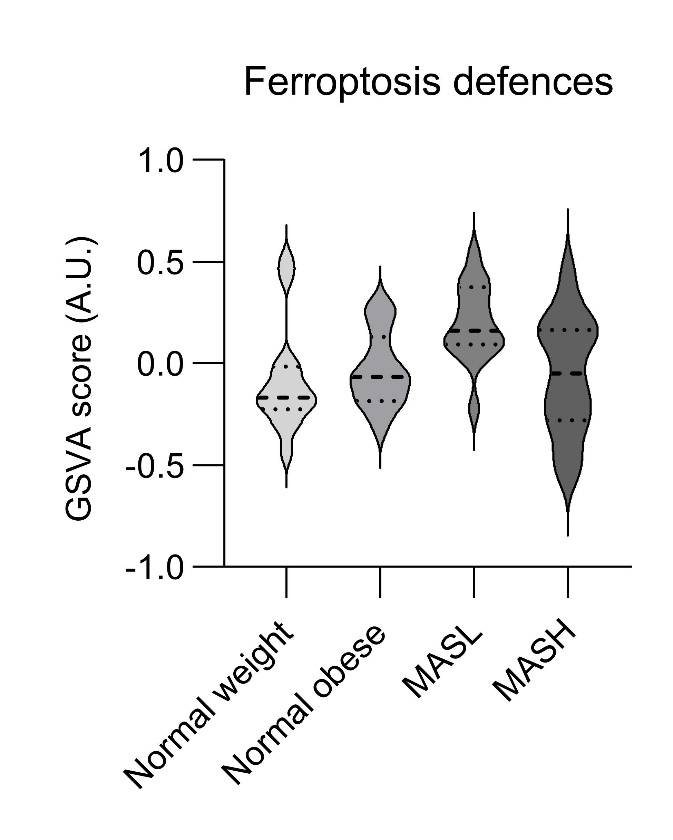

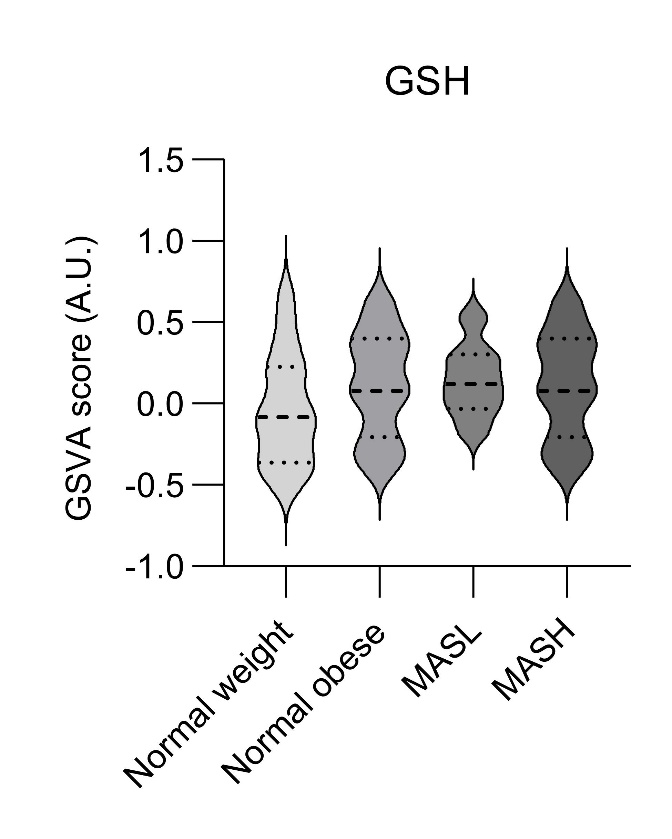

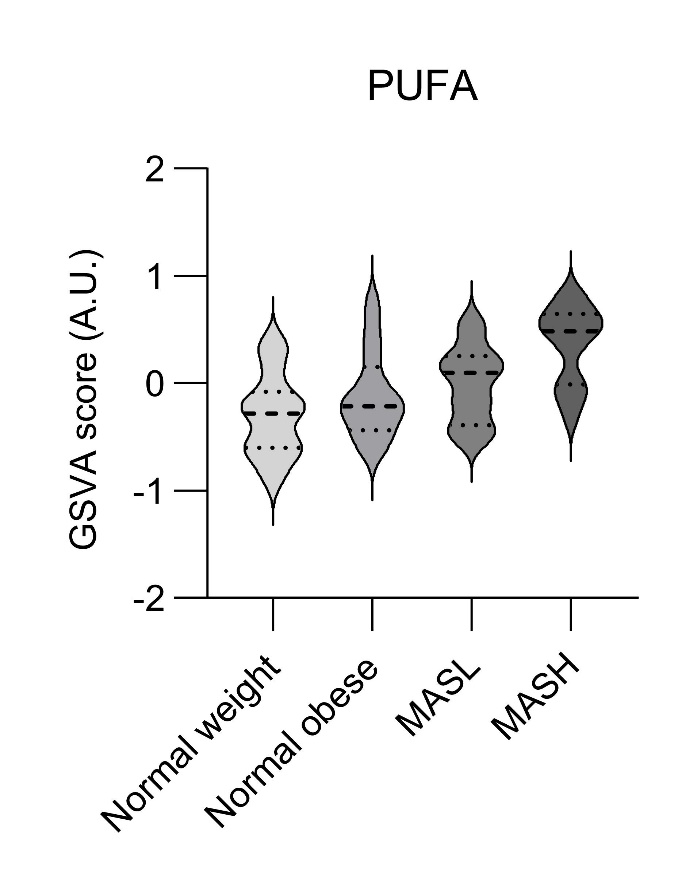

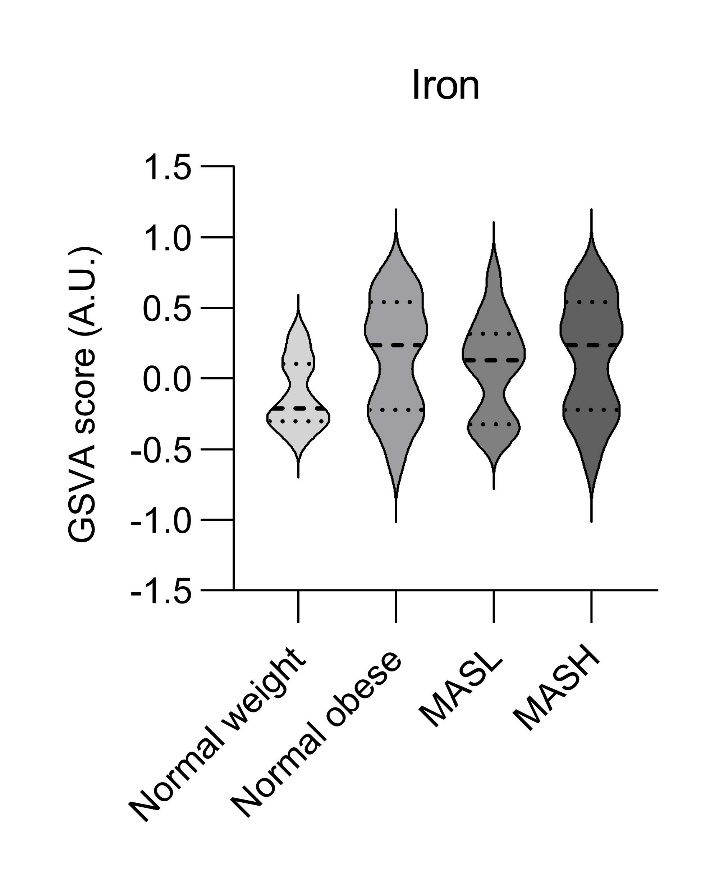


**Fig. S11.** **Gene set variation analysis for 4 ferroptosis-related gene sets in publicly available database GSE126848 according to liver histology**

The gene set variation analysis (GSVA) score per gene set, *i.e.* ‘ferroptosis defenses’, ‘GSH’, ‘PUFA’ and ‘Iron’, was calculated in every MASLD patient and control of transcriptomics database GSE126848 (n = 57), followed by plotting per histologic class. One-way ANOVA test was performed.

Figure S12


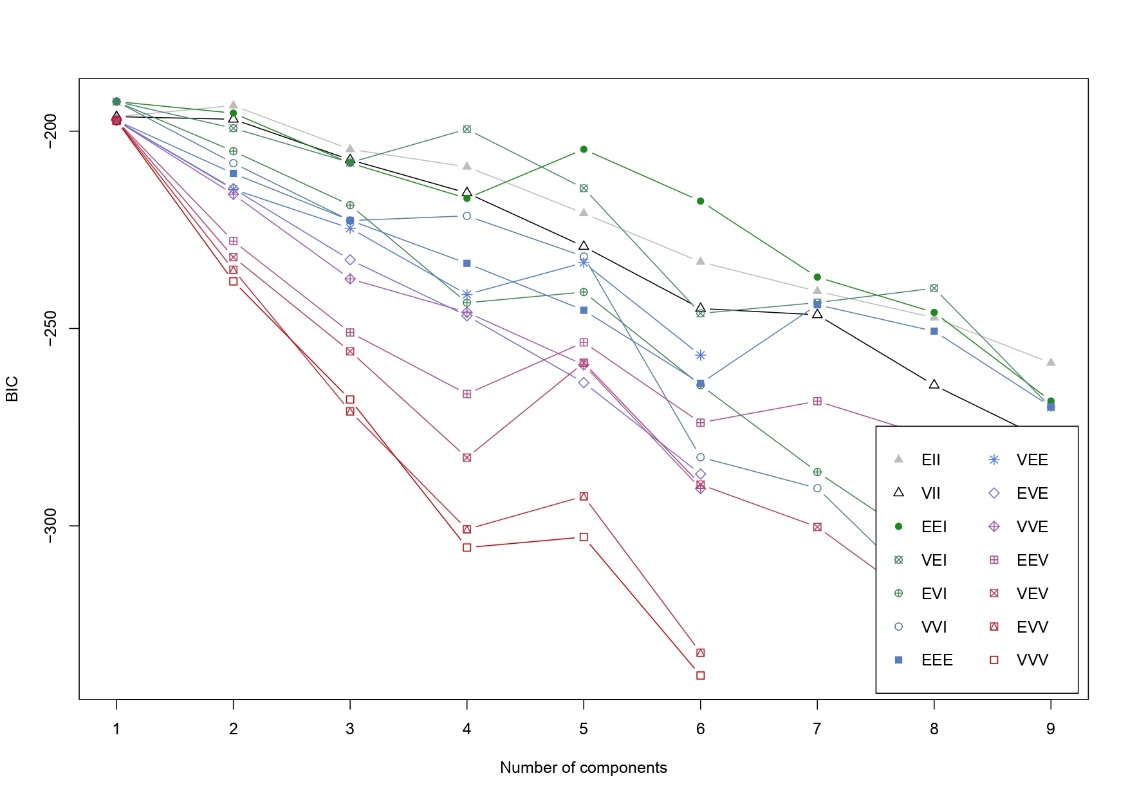


A

B


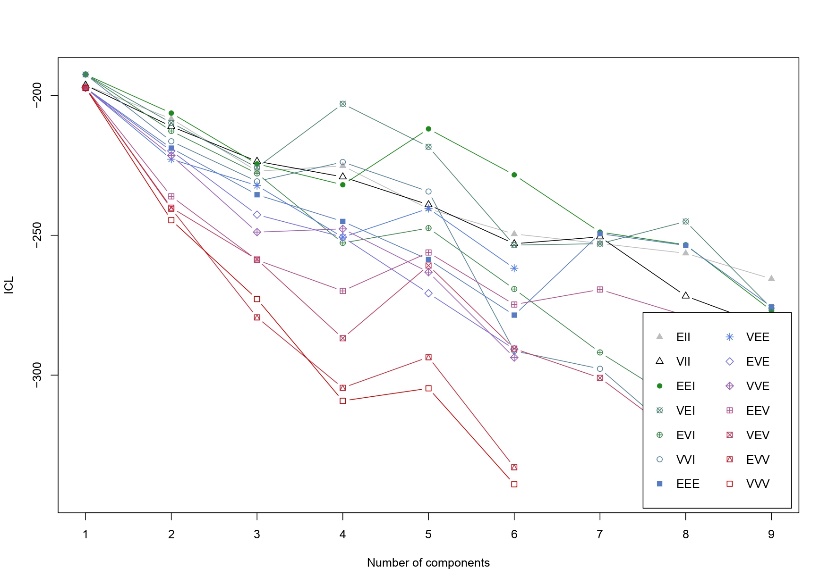

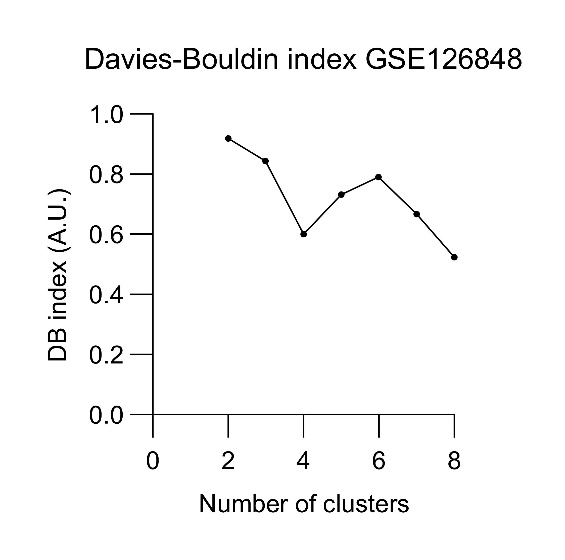

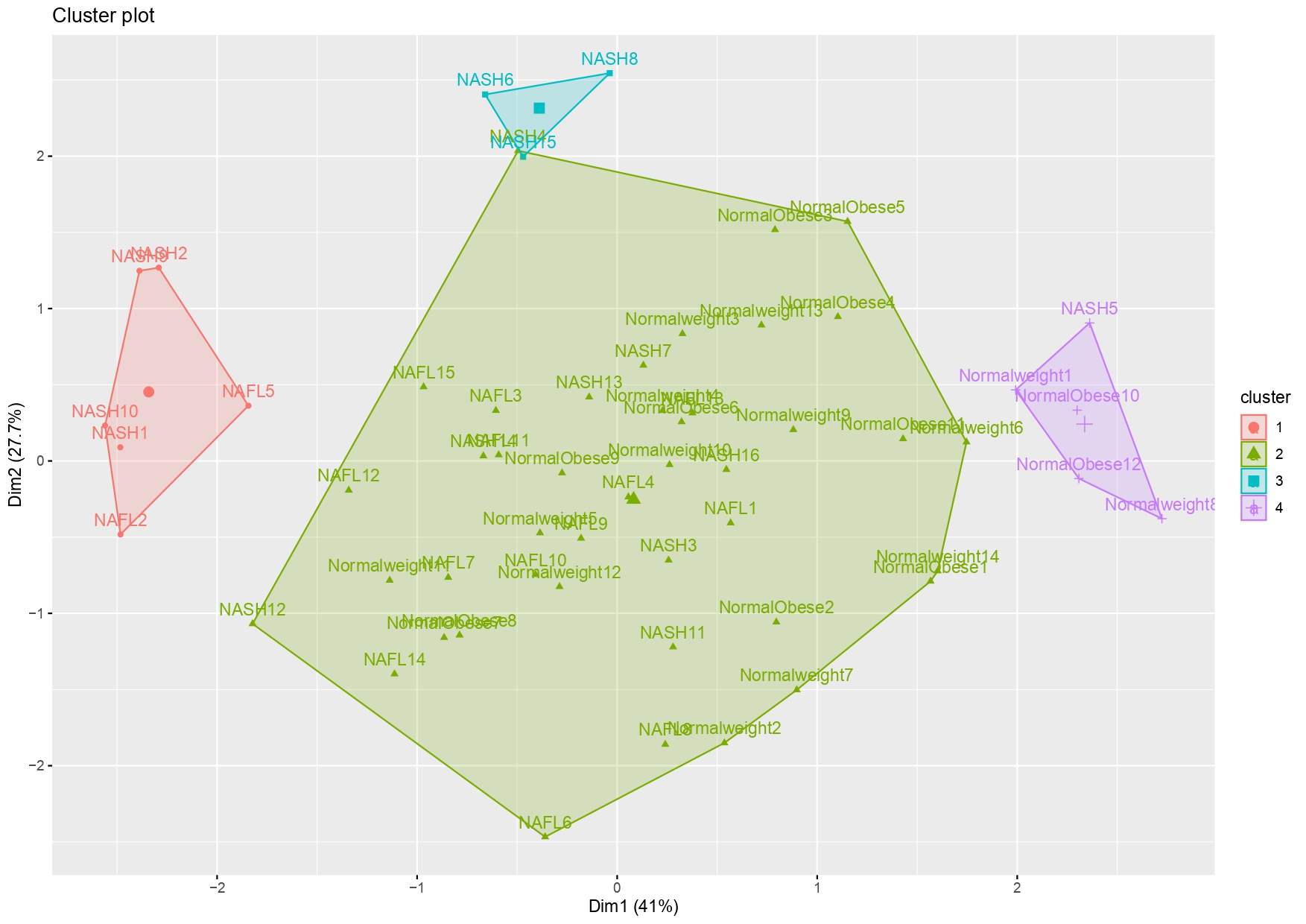


C

D

**Fig. S12.** **Unsupervised clustering of MASLD patients and controls from GSE126848 based on ferroptosis-related gene sets by Gaussian Mixture models.**

(A) Values of Bayesian Information Criterion (BIC) were plotted for a given number of clusters by each model of Gaussian Mixture modelling. (B) Likewise, values of the Integrated Complete-data Likelihood (ICL) were plotted per number of clusters by each fitted Gaussian Mixture model. (C) Values of the Davies-Bouldin’s index were plotted for any given number of clusters defined by Gaussian Mixture modelling to perform the elbow method. Lower levels of this index indicate a better separation between the clusters. (D) Principal component analysis (PCA) plot was used to visualize the 4 clusters of MASLD patients and controls constructed by the VEI model (diagonal distribution, variable volume, equal shape) of Gaussian mixture modelling.

Figure S13

A


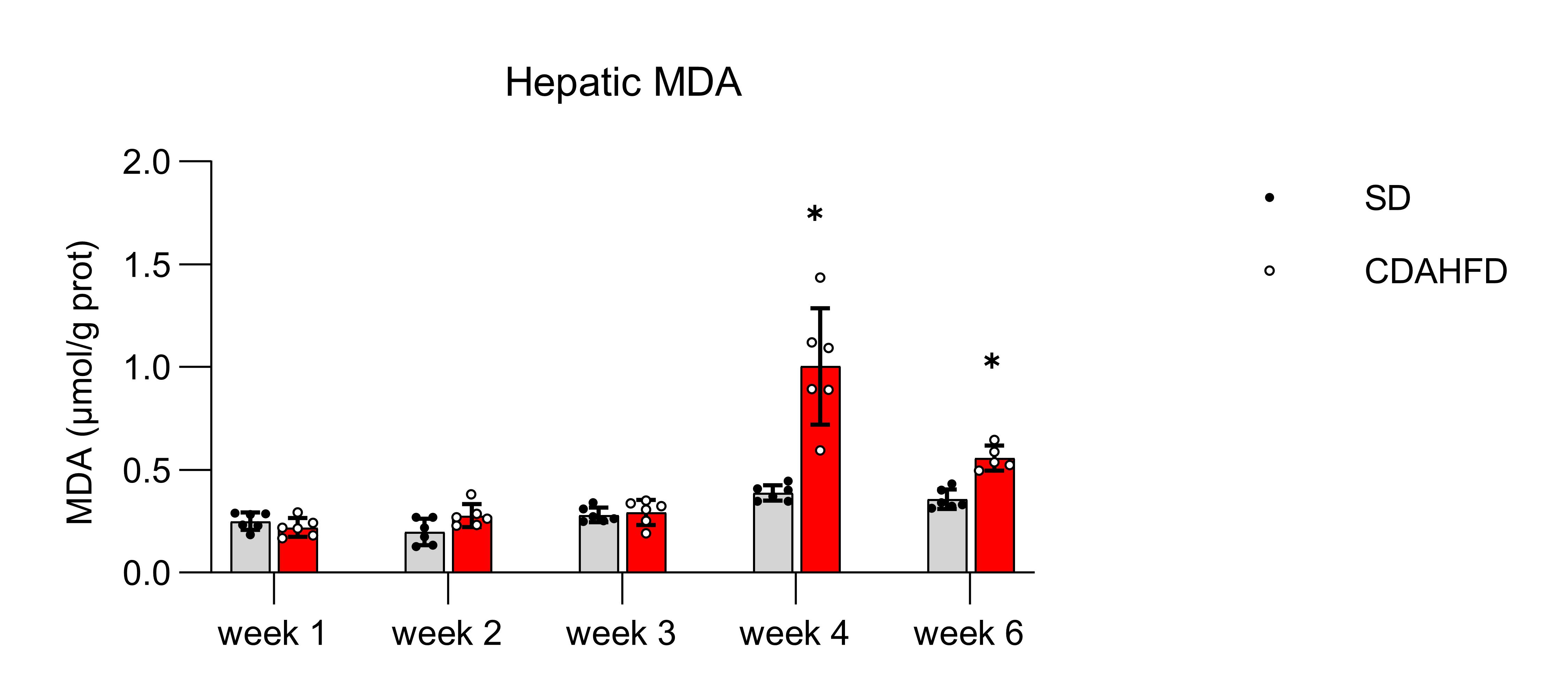

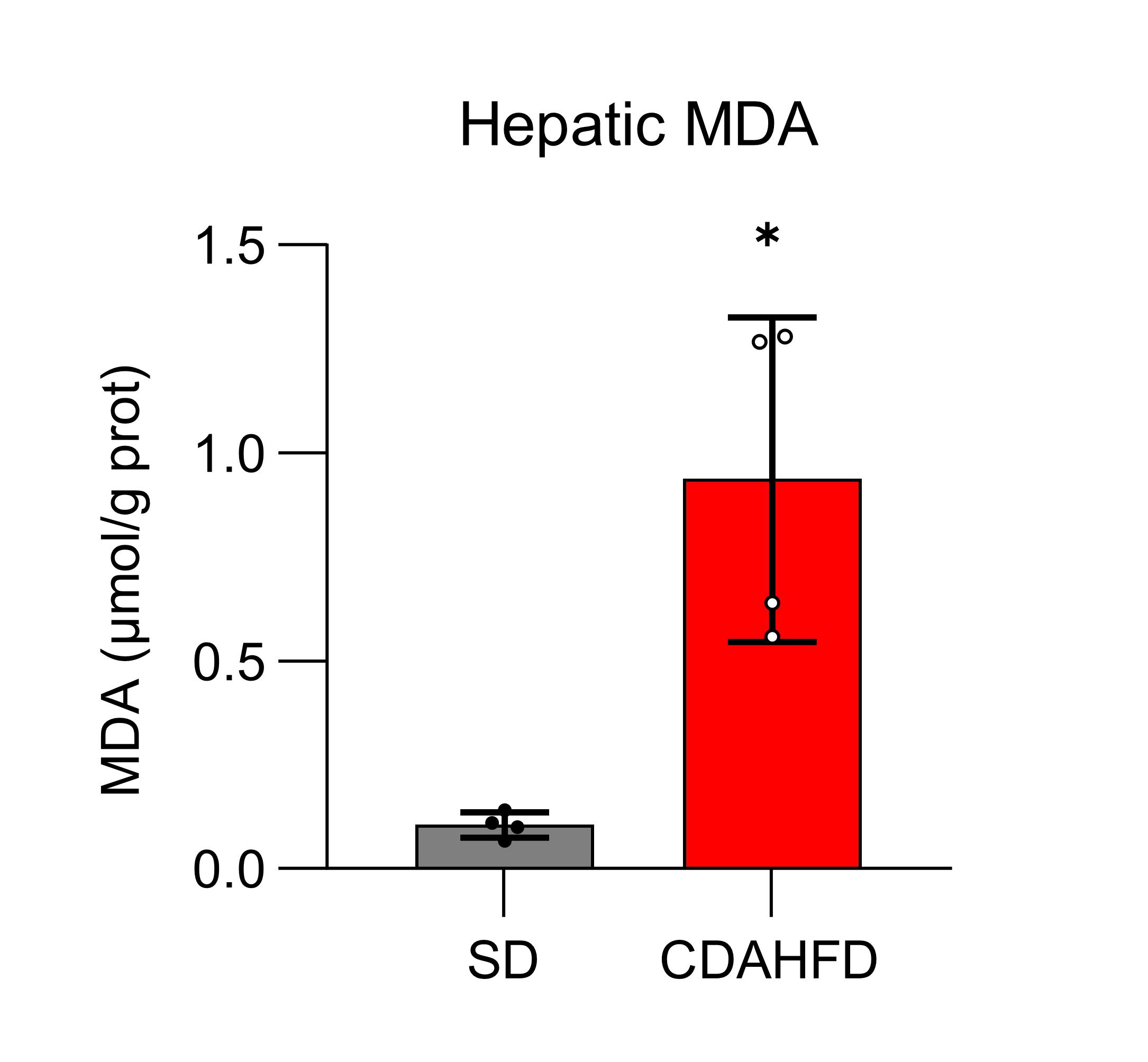


B

SD CDAHFD positive control


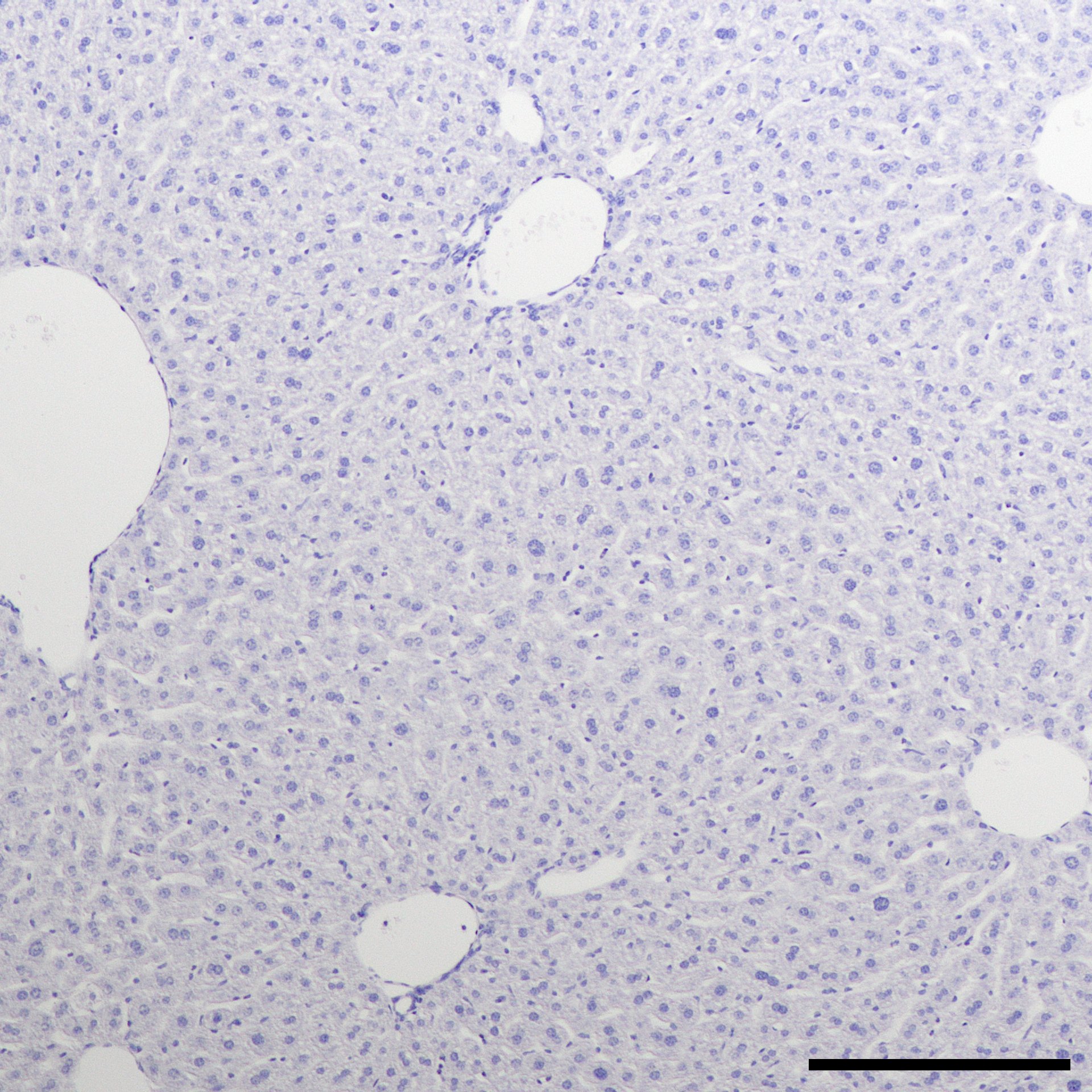

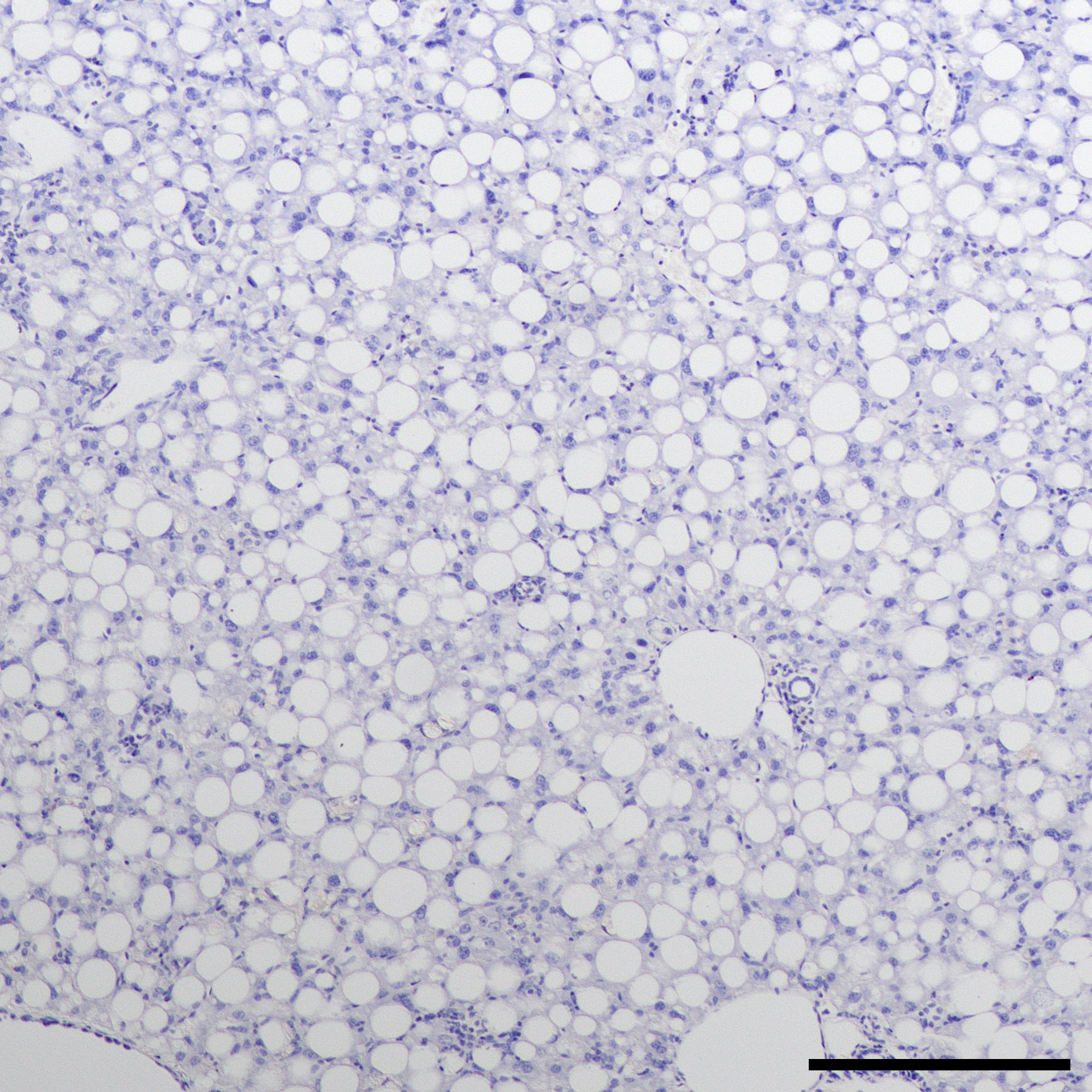

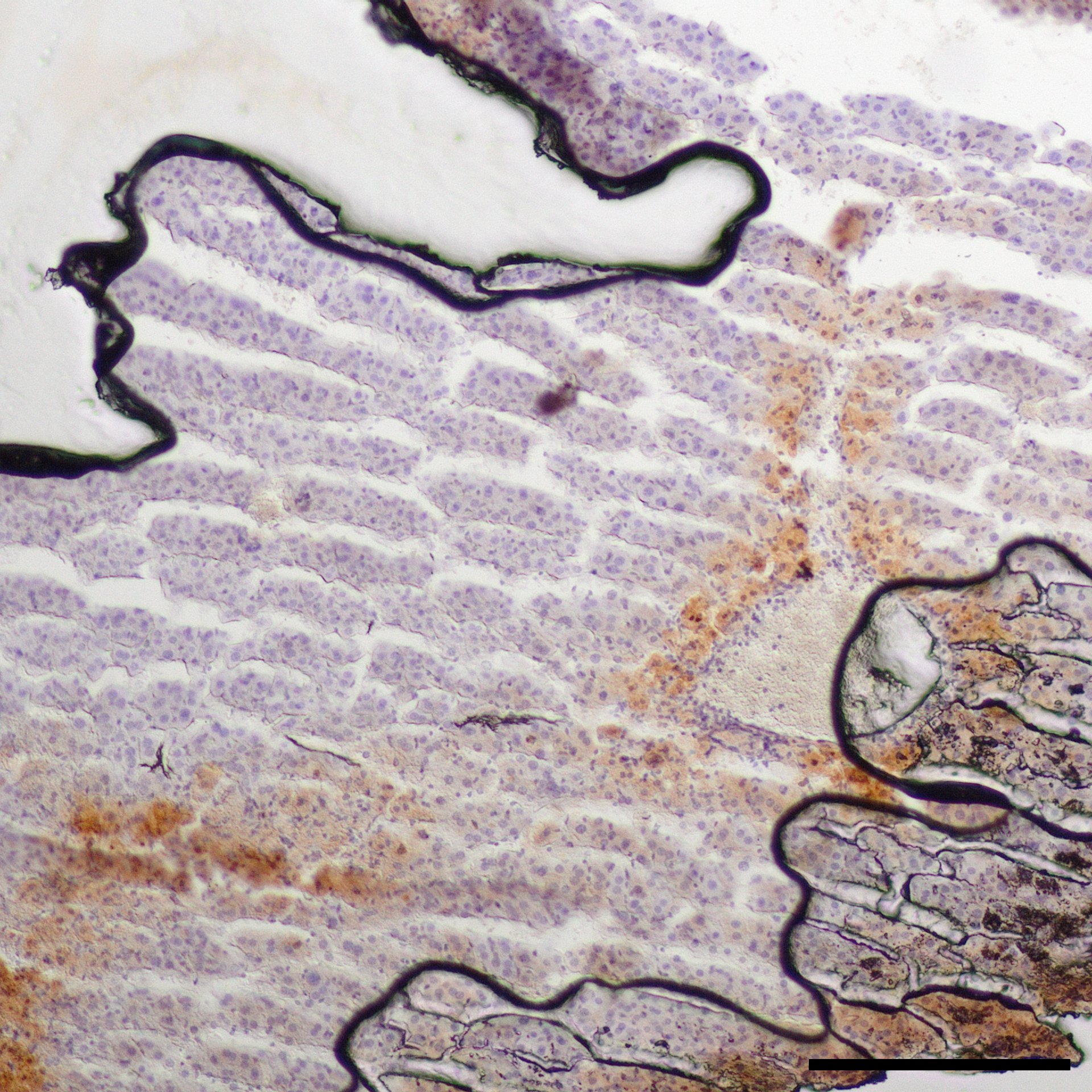


Cleaved caspase-3

Omission of primary antibody


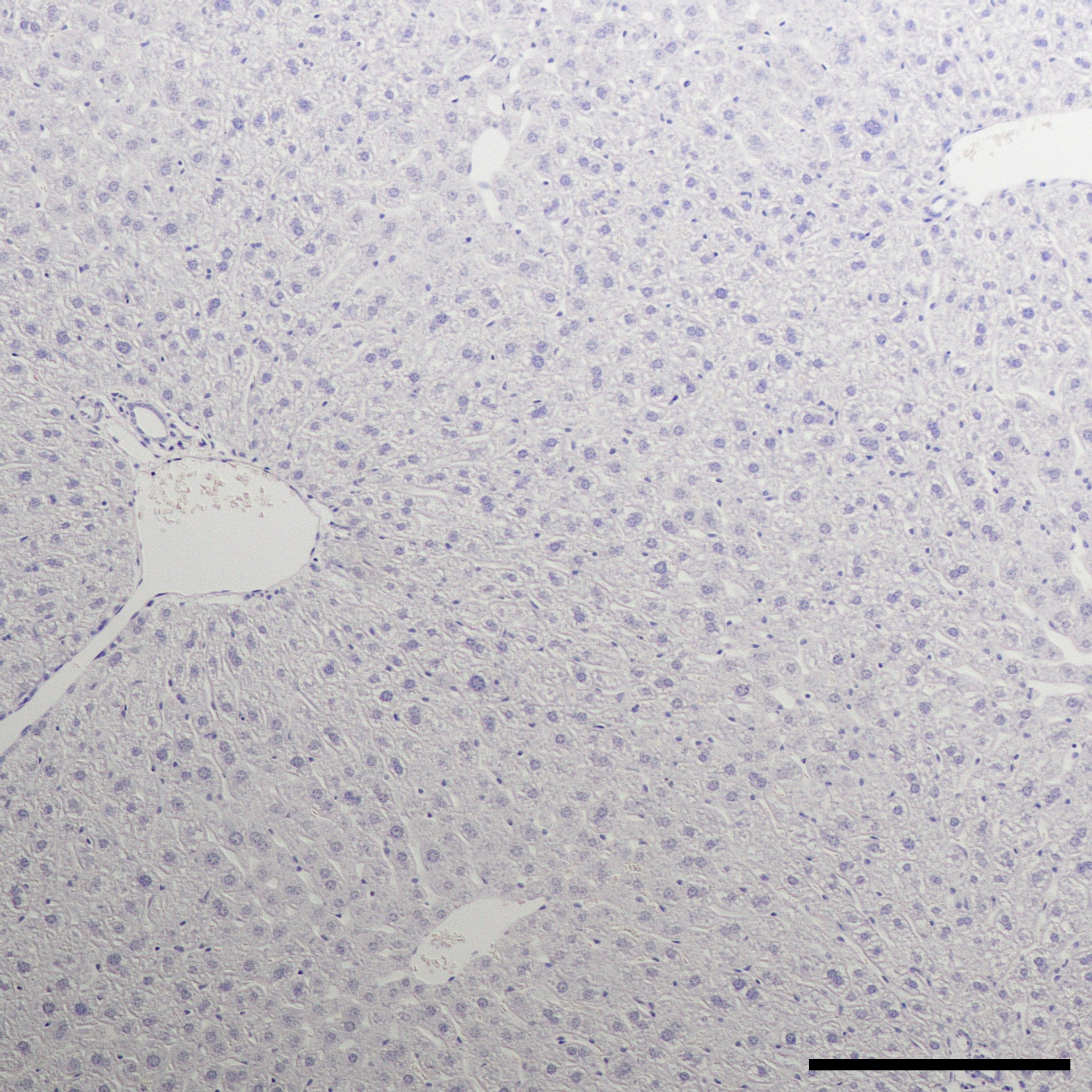


C


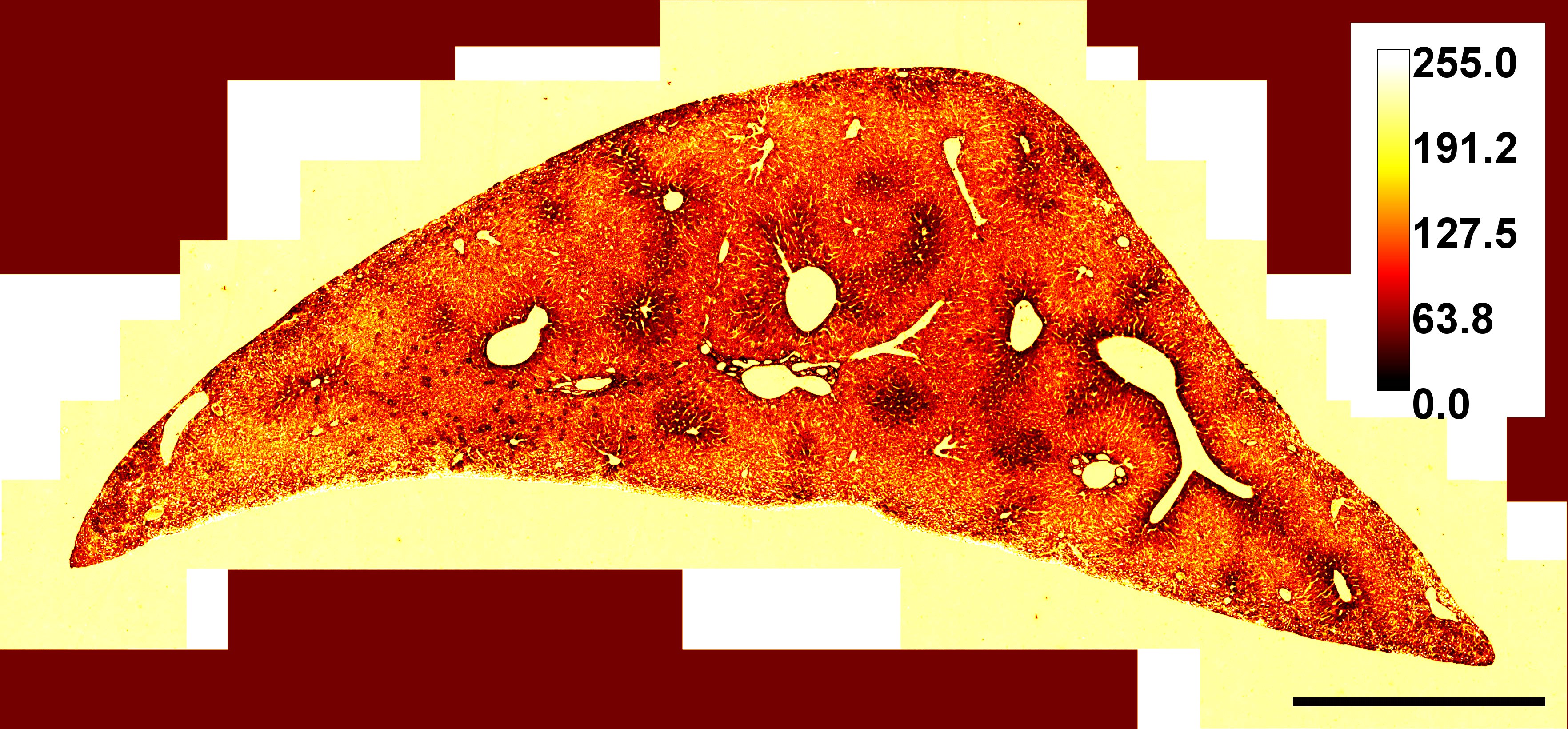

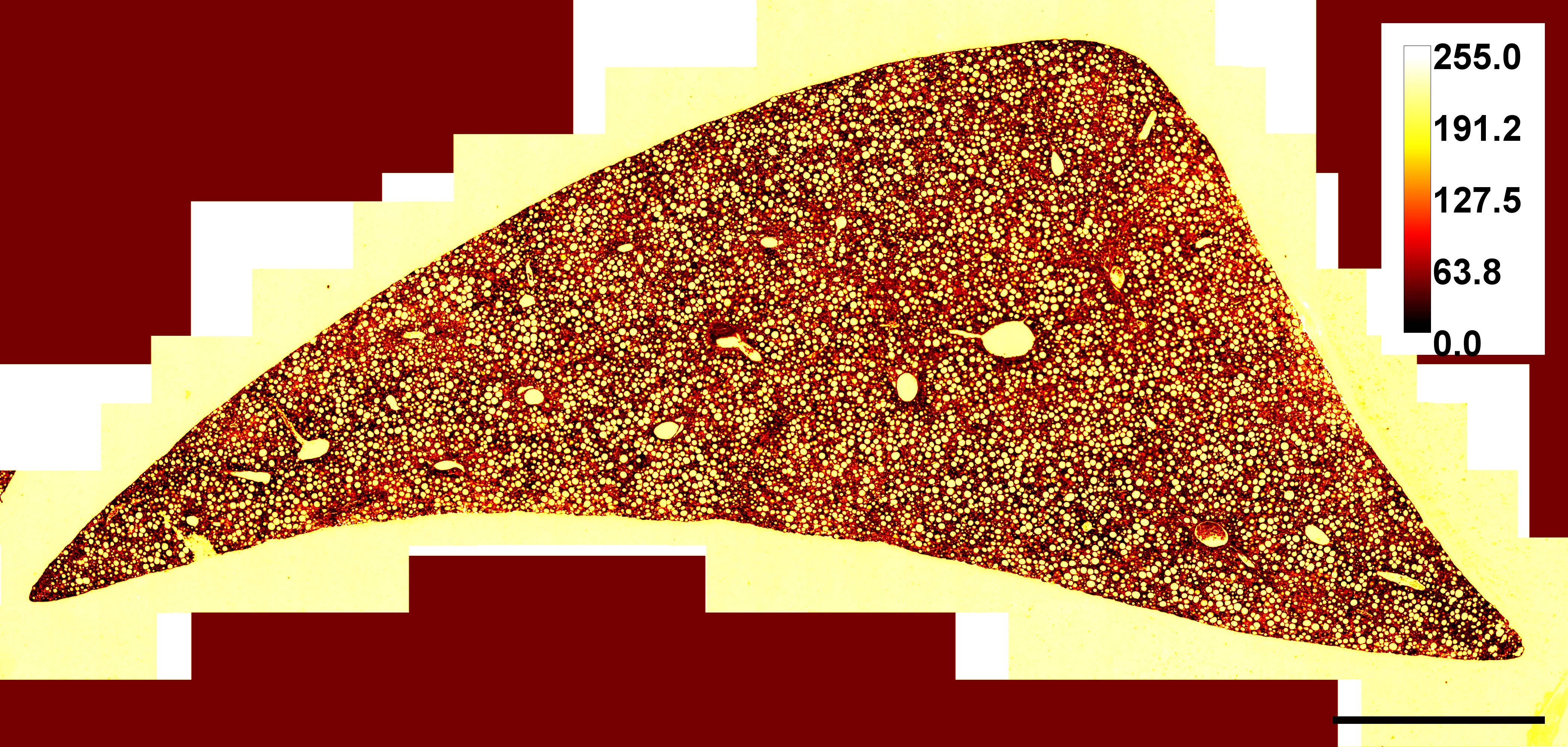


DAB signal 4HNE

CDAHFD

SD


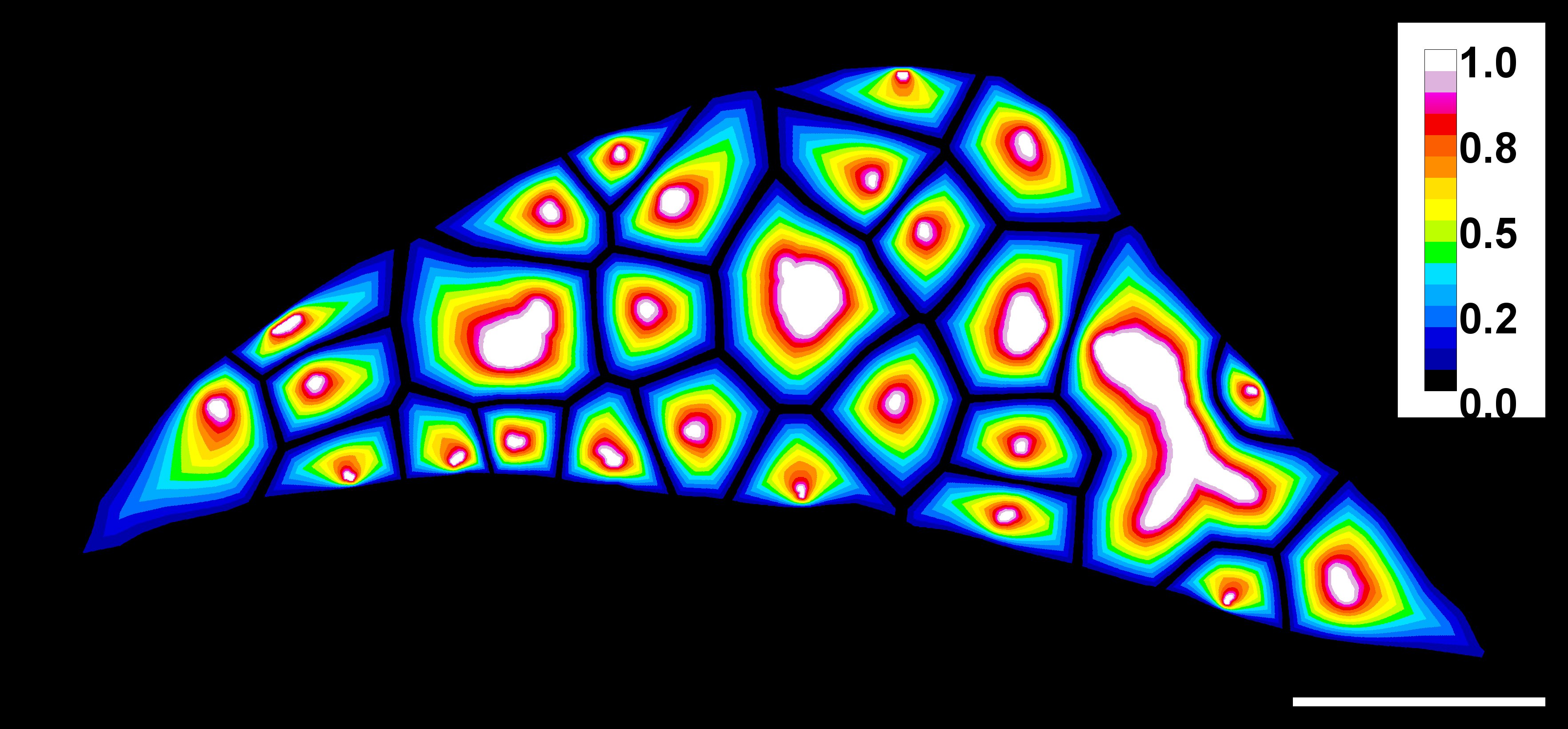

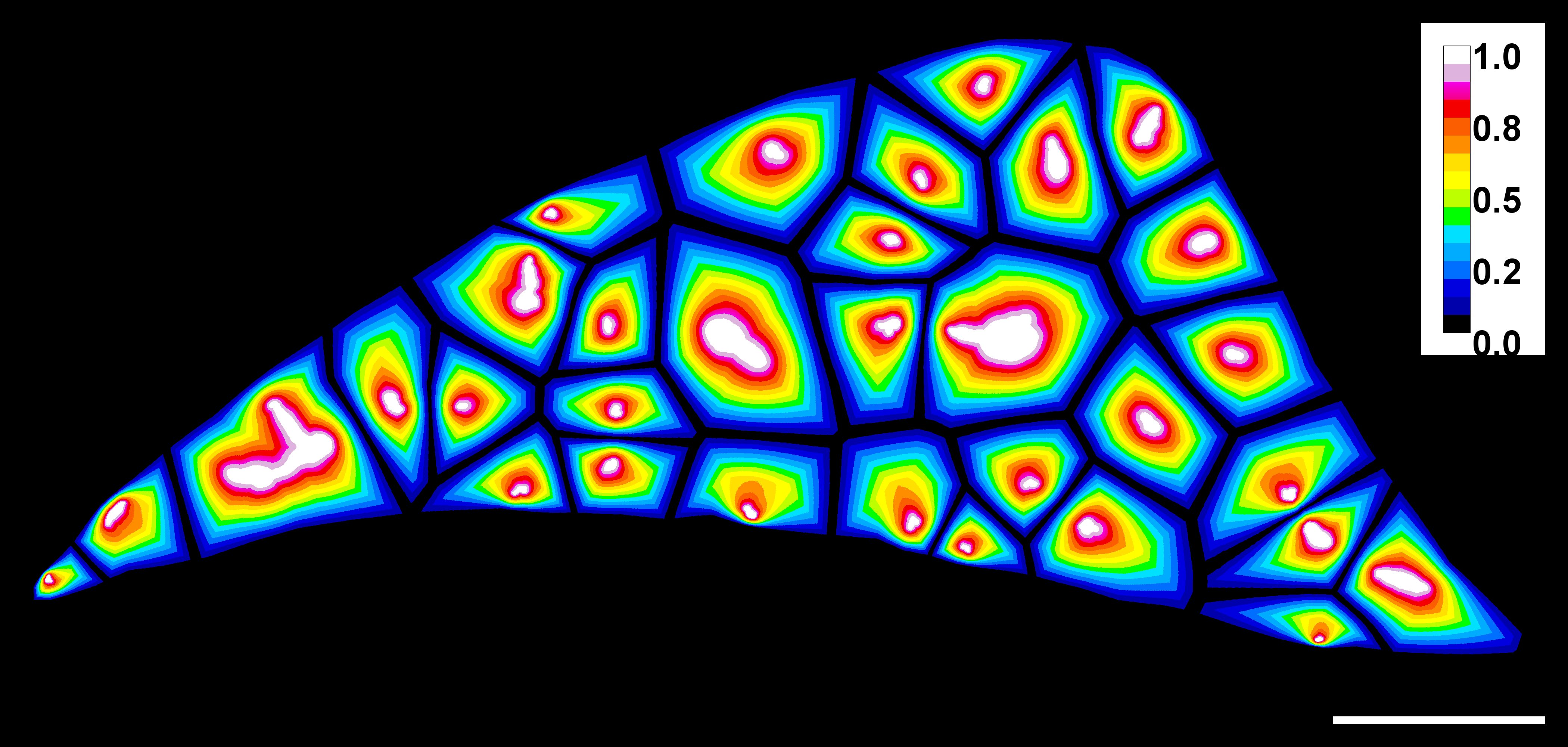


Relative distance map


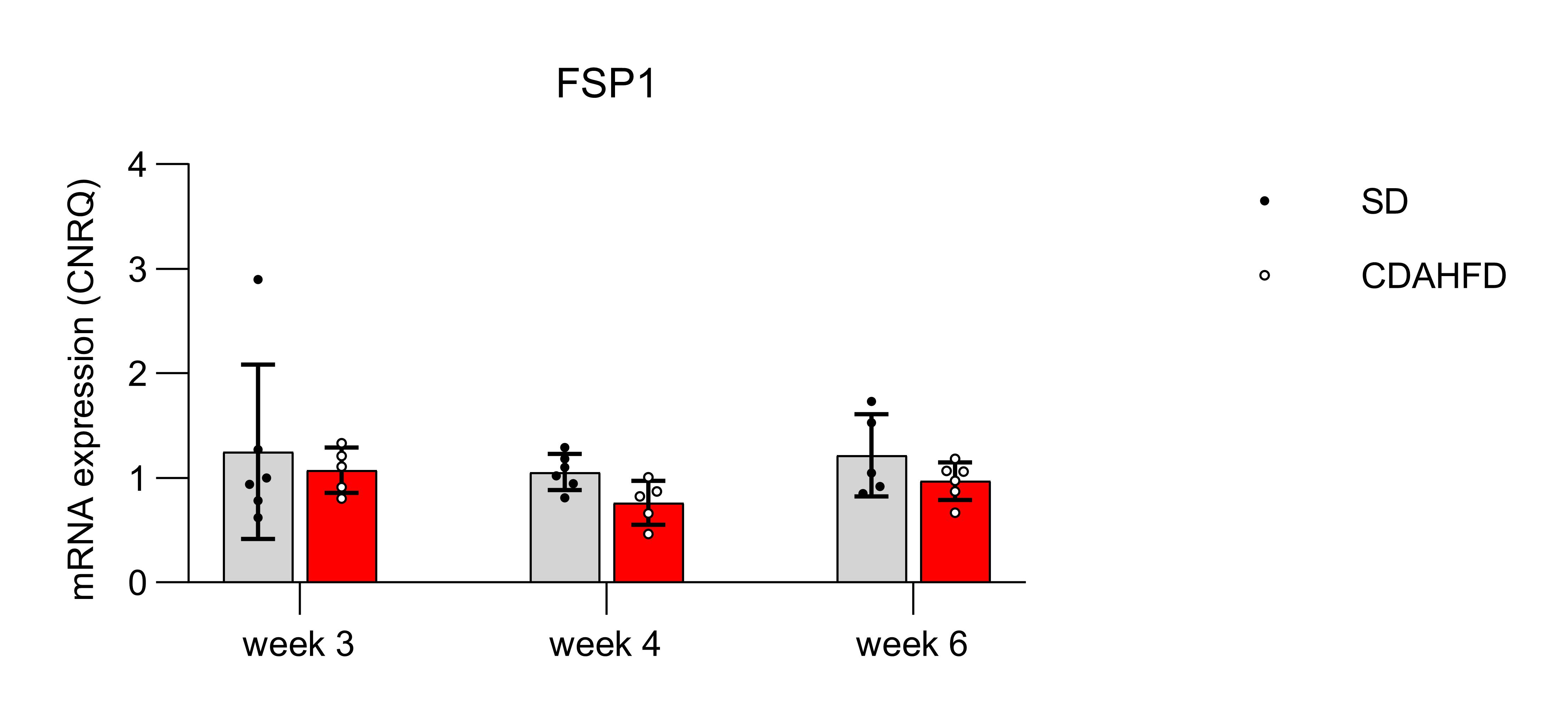

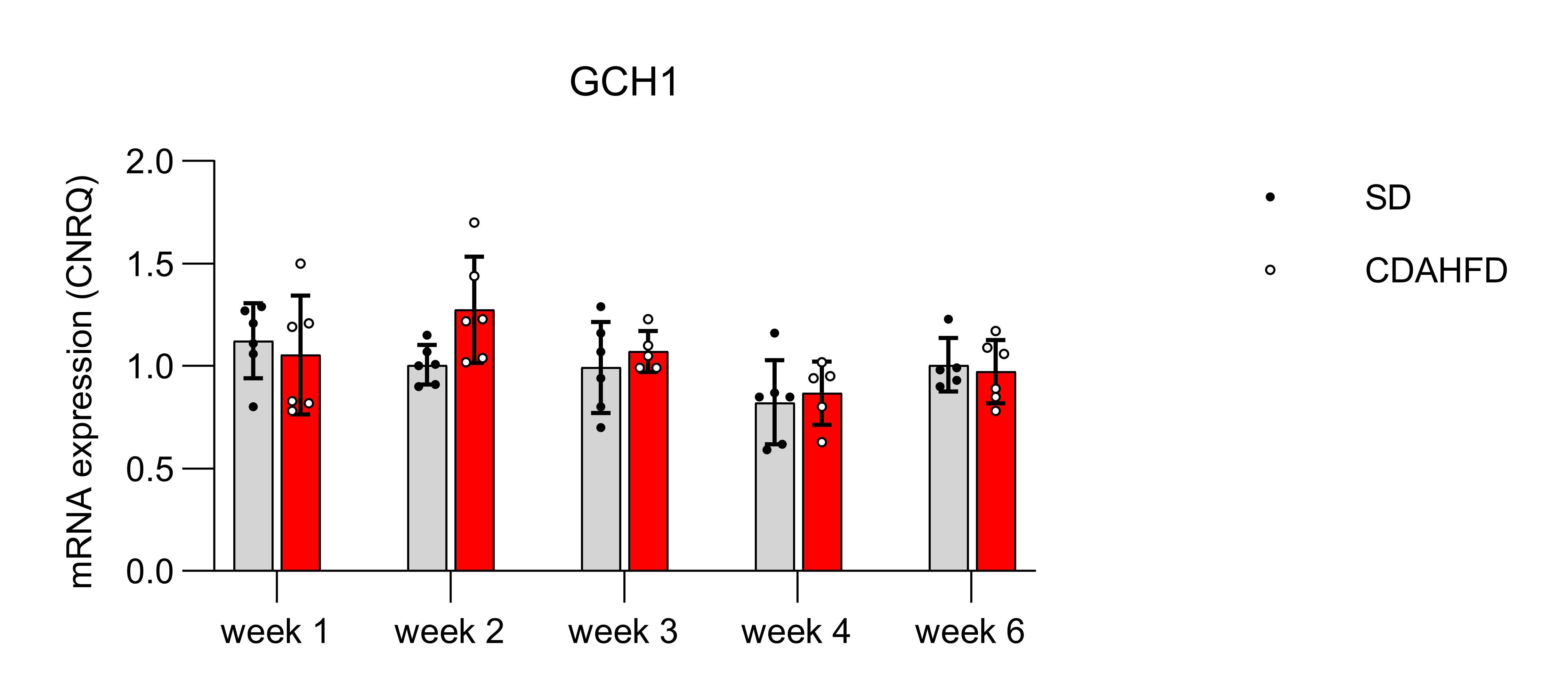

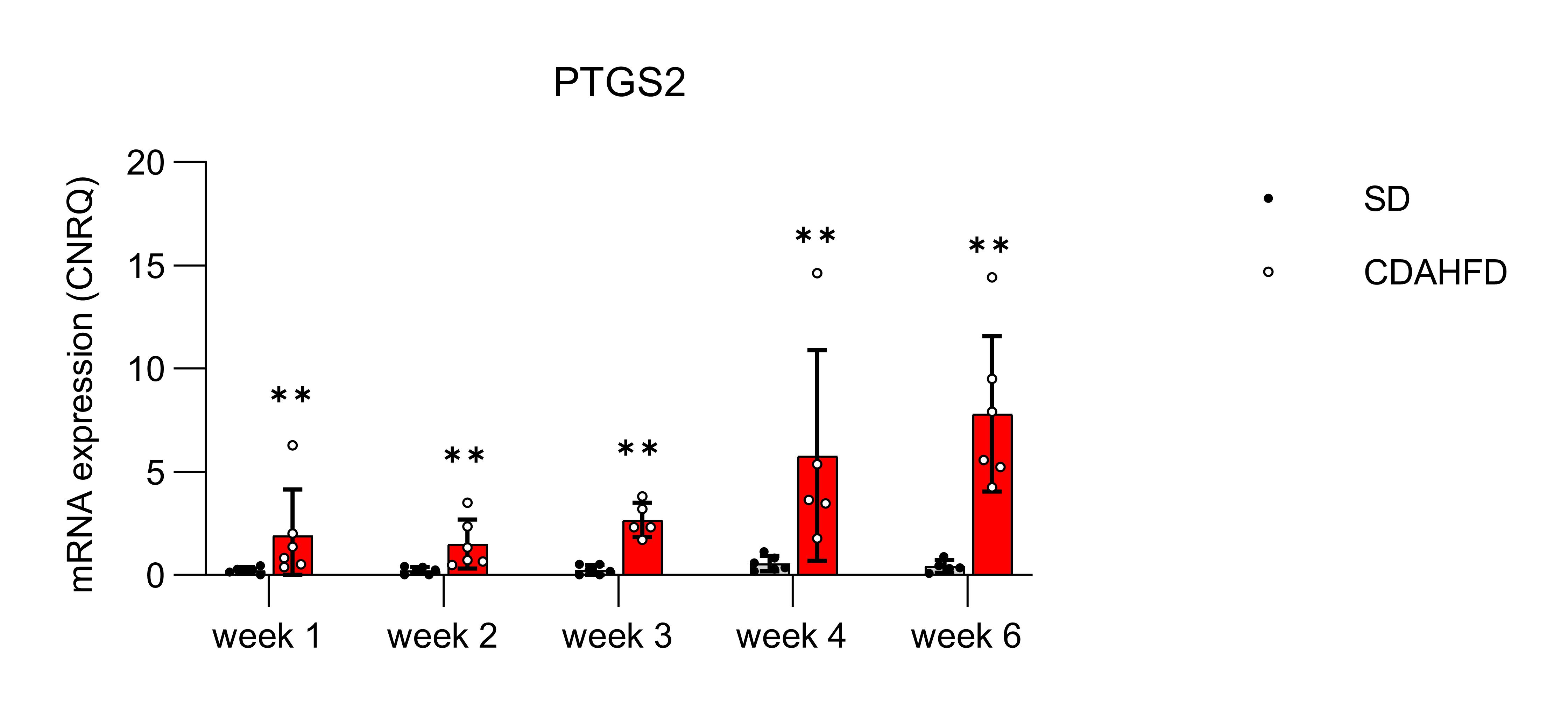

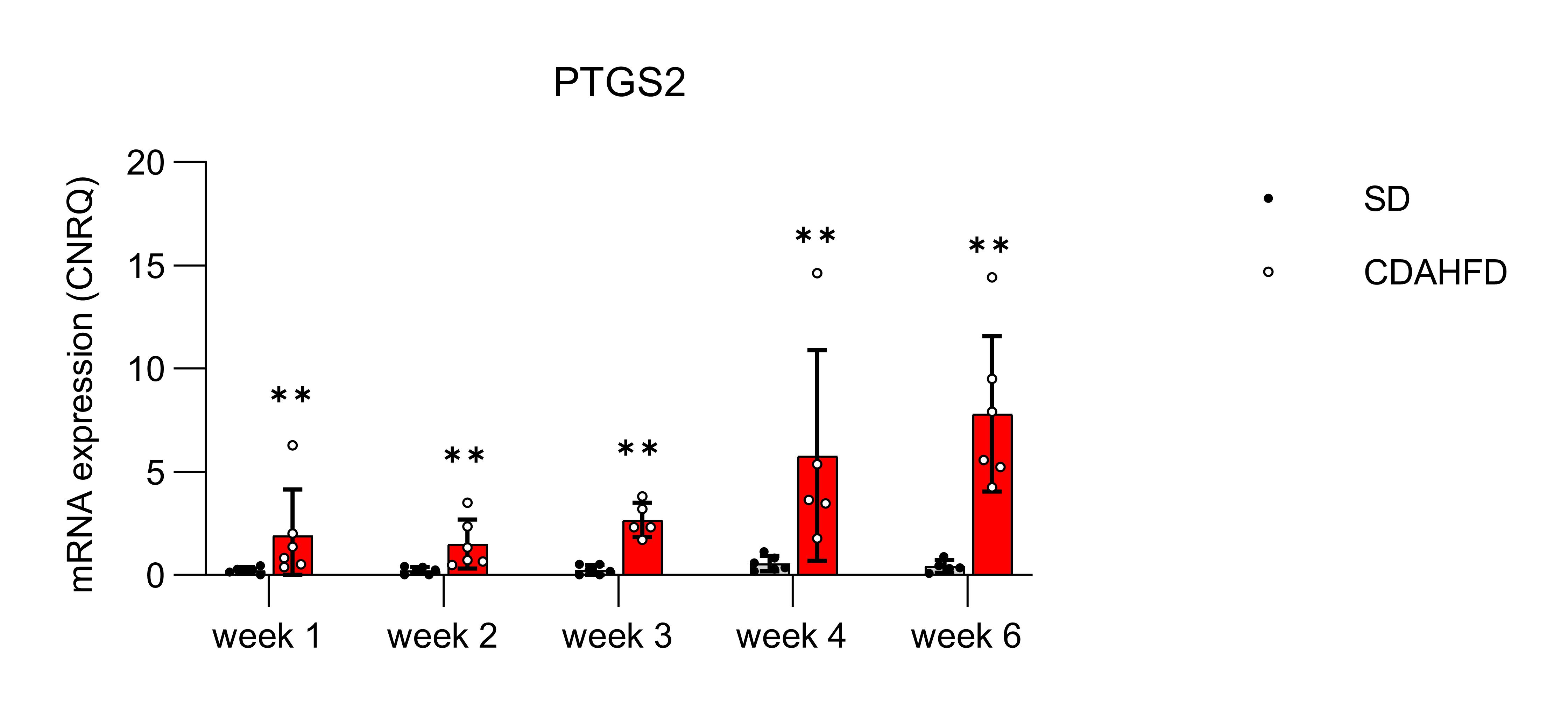


D

**Fig. S13. Detection of ferroptosis markers in CDAHFD-induced MASH model.**

Markers of hepatic cell death and ferroptosis were measured in groups of mice fed the standard diet (SD) or choline-deficient L-amino acid-defined high-fat diet (CDAHFD) with an emphasis on the 4 week timepoint (n = 6 per group). (A) In a second independent experiment, hepatic MDA was measured in mice fed SD or CDAHFD for 4 weeks (n=4). (B) Representative images of IHC for cleaved caspase-3 in liver tissue obtained from mice fed SD or CDAHFD for 4 weeks, as well as a mouse that received paracetamol (300mg/kg bodyweight, A7085, Sigma) via oral gavage 24 hours earlier as positive control. Moreover, liver tissue with omission of primary antibody is shown as control for the detection system used for IHC for 4-hydroxynonenal (4HNE), glutathione peroxidase 4 (Gpx4) and cleaved caspase-3. (C) Representative images of the quantification of immunohistochemistry (IHC) for 4HNE and GPX4 in whole slide liver images per relative distance in reconstructed liver lobules. The upper panel shows representative images of deconvoluted DAB signal for 4HNE, presented with the pseudocolour image “red hot”. The lower panel displays reconstructed lobules in whole-slide liver images with centrilobular veins displayed in white. Each pixel in the whole liver slide received a value ranging from 0 (edge of lobule) to 1 (the central vein), which represents its relative distance within the constructed liver lobule. Each color of the look-up table “16 colours” shows points at the same relative distance between the centre and lobular edge (annotated in black) within these lobules. Scale bar 1000µm. (D) Hepatic mRNA expression of ferroptosis suppressor protein 1 (*Fsp1*), GTP cyclohydrolase 1 (*Gch1*) and prostaglandin-endoperoxide synthase 2 (*Ptgs2*) in mice on SD or CDAHFD on different time points, expressed as calibrated normalized relative quantities (CNRQ). Data presented as mean ± standard deviation. *p <0.05; **p <0.01; Mann-Whitney U test with correction for multiple hypothesis testing.

Figure S14

A


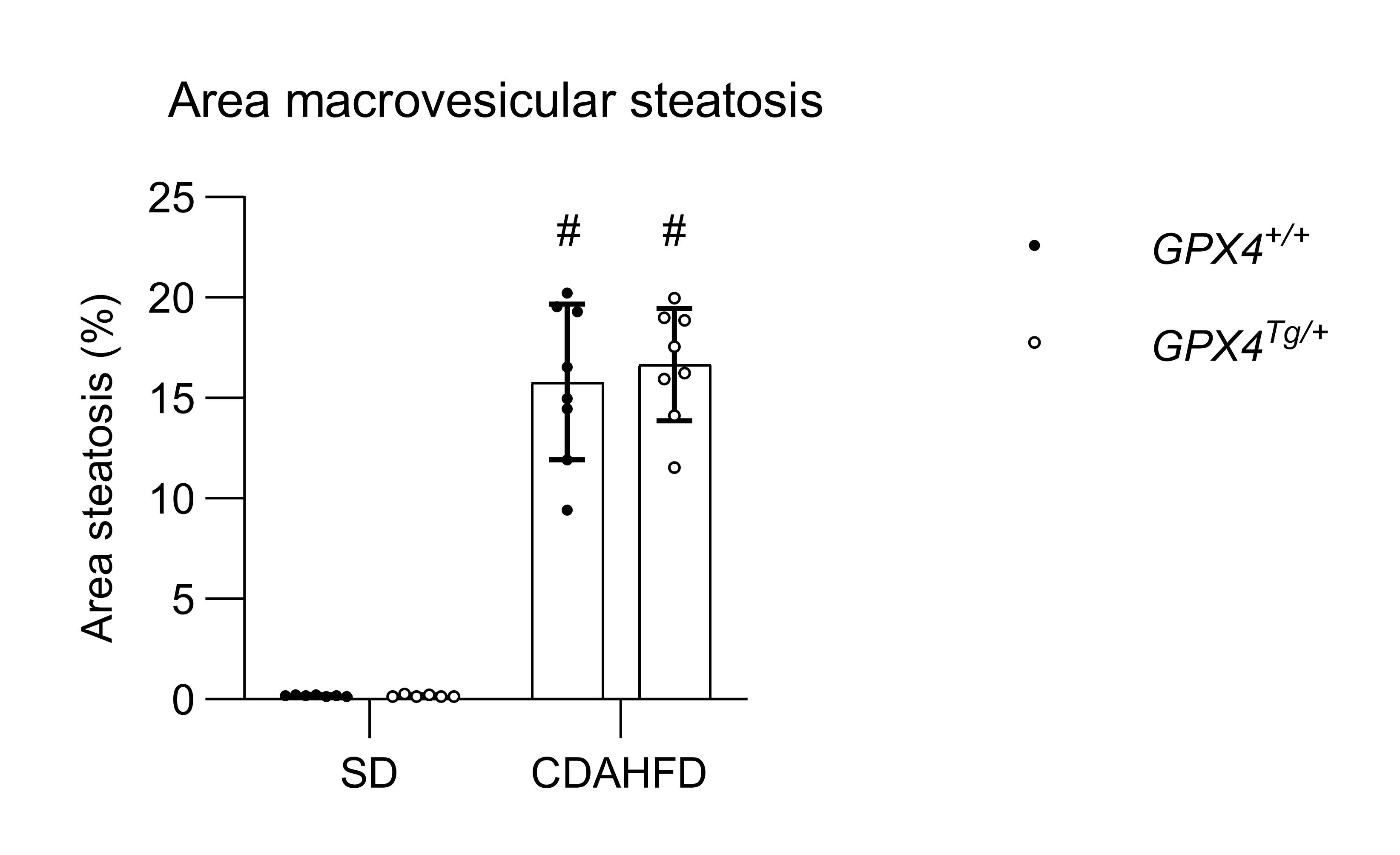

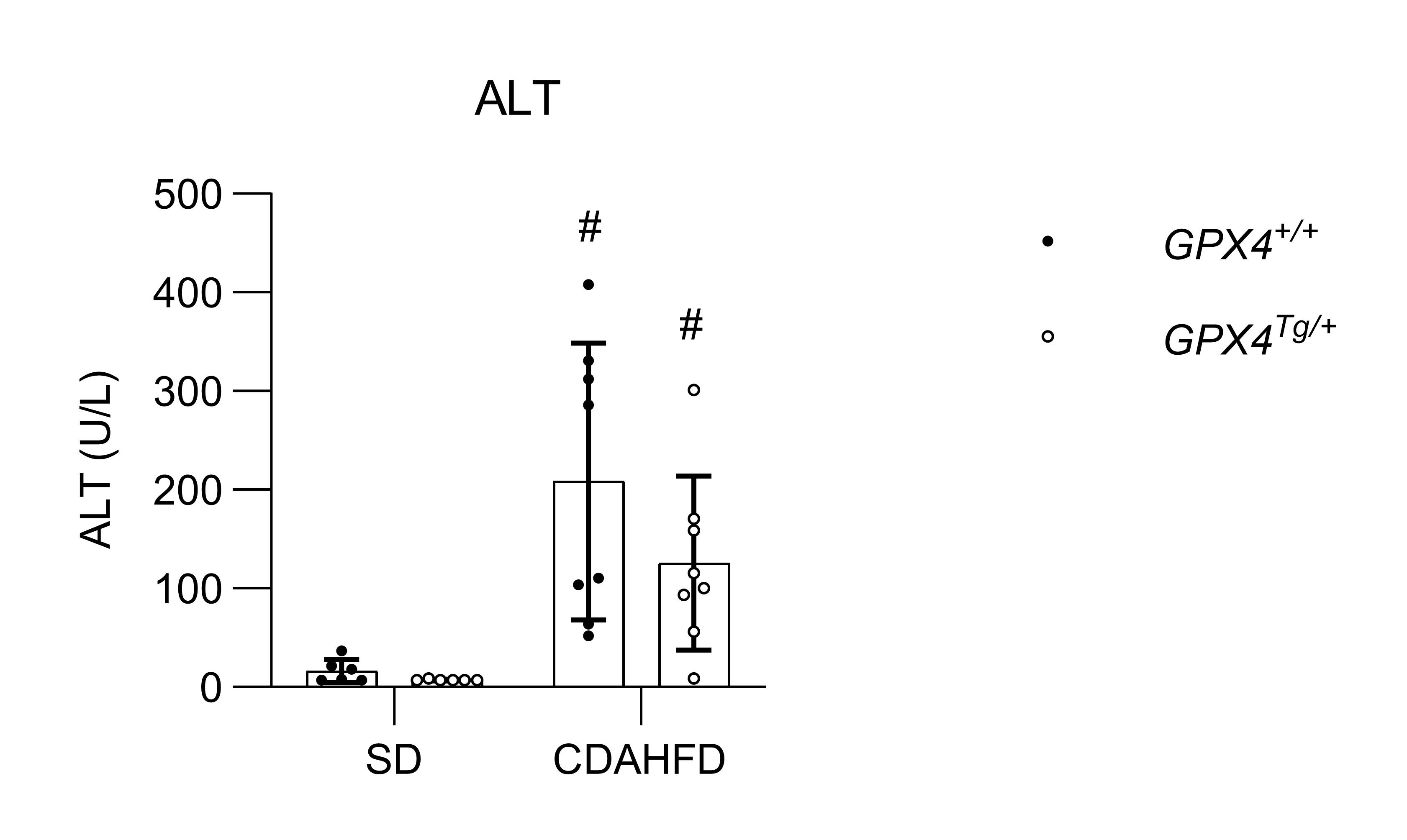

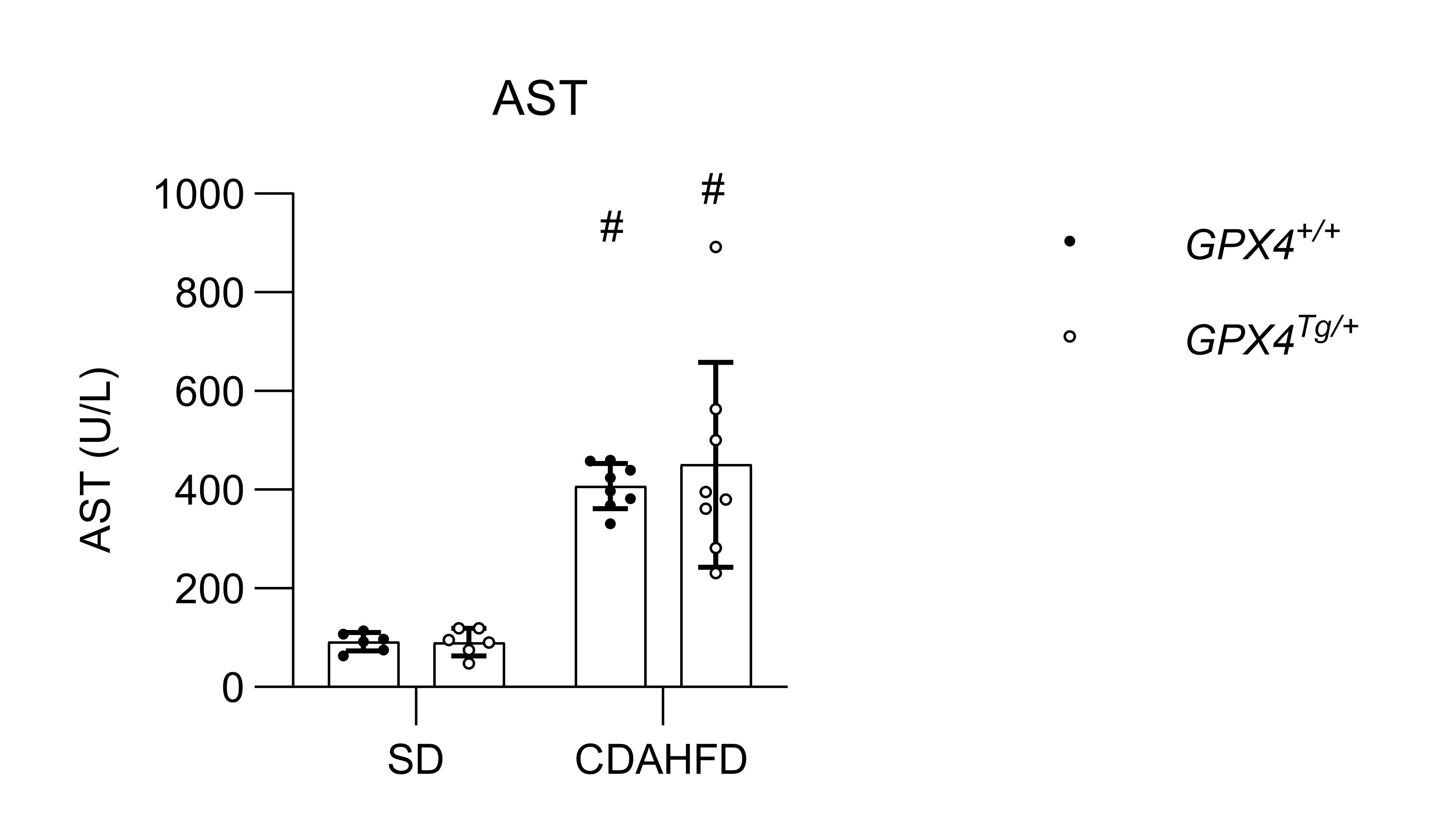

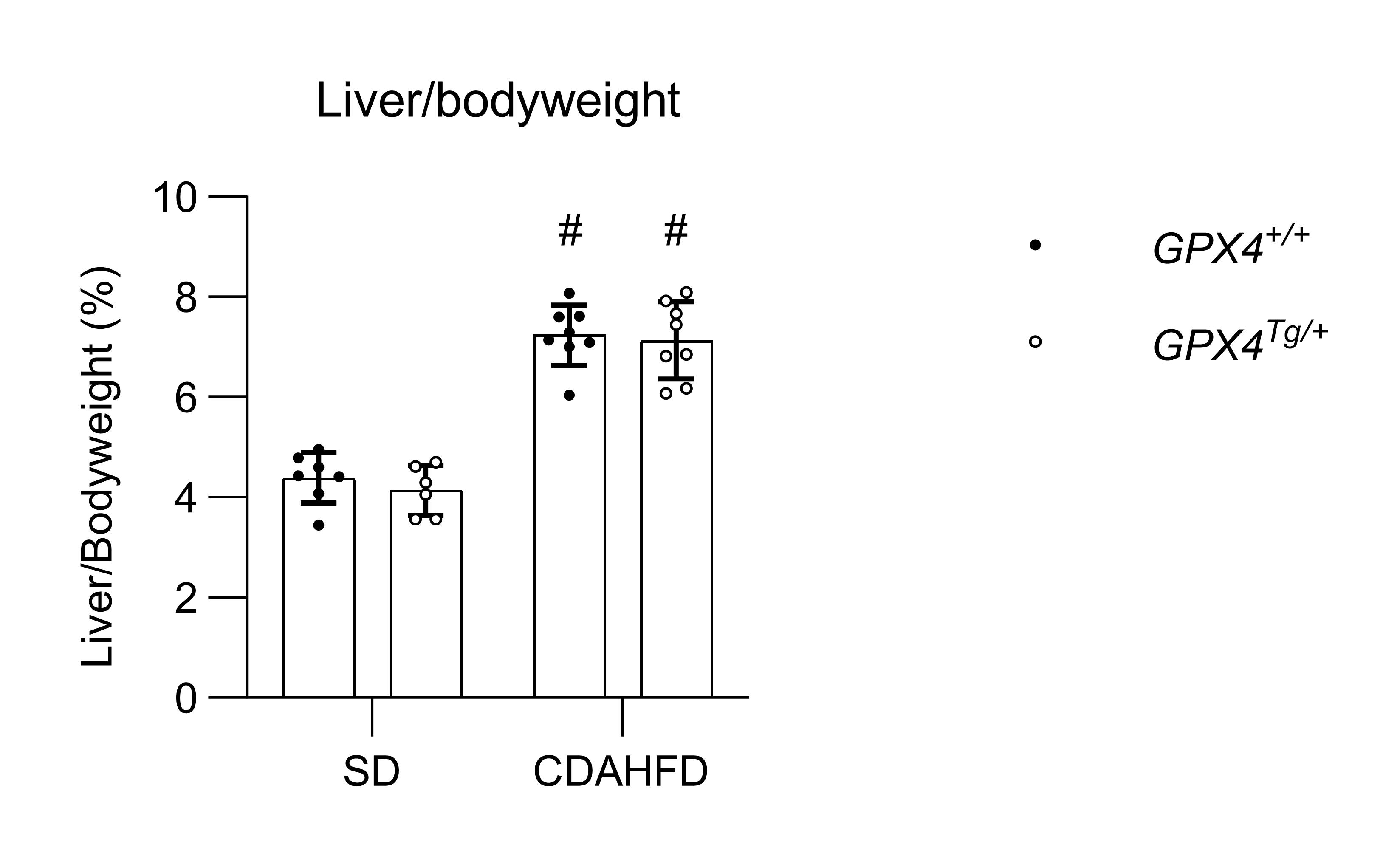


B

C

SD + *Gpx4^+/+^*  SD + *Gpx4^Tg/+^* CDAHFD + *Gpx4^+/+^* CDAHFD + *Gpx4^Tg/+^*


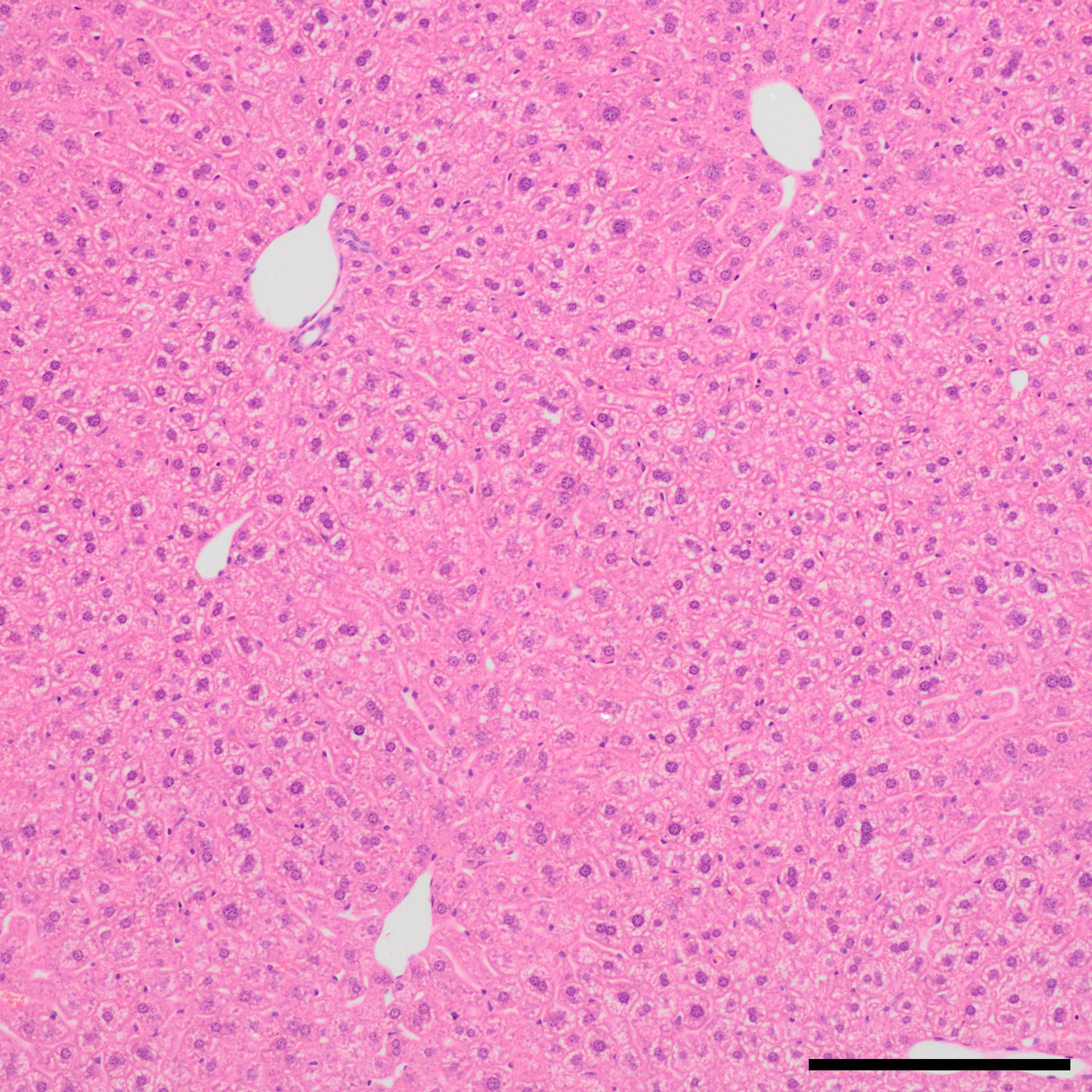

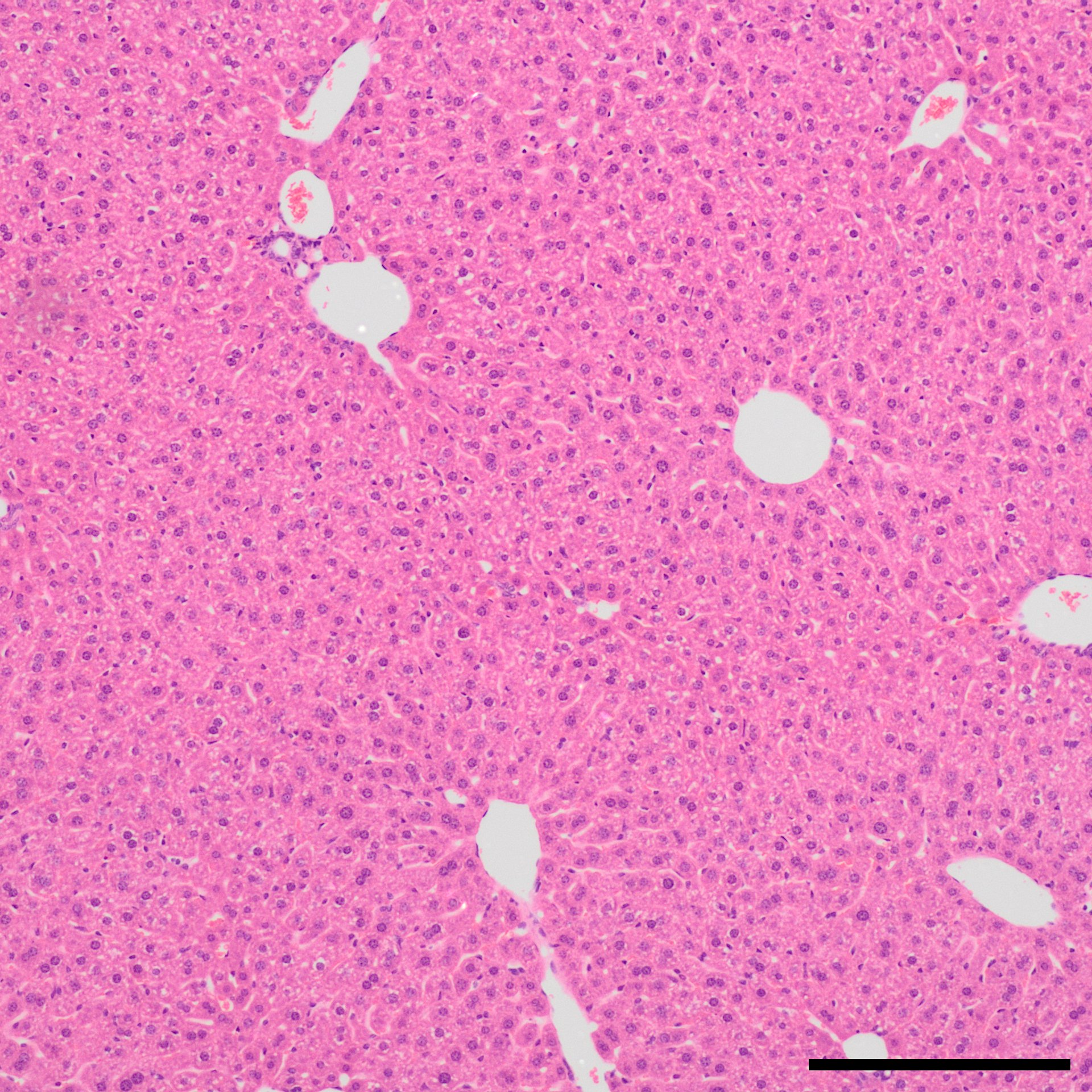

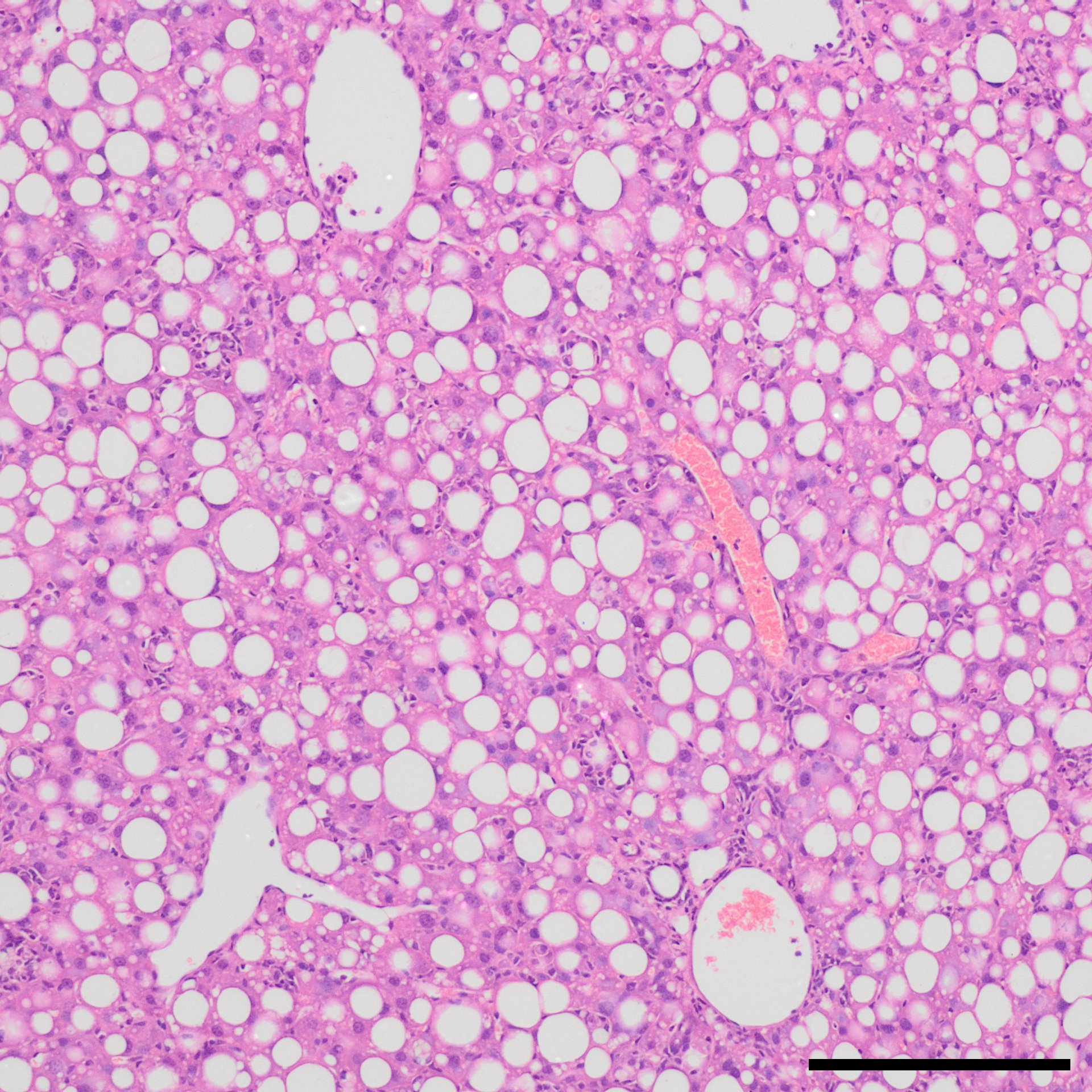

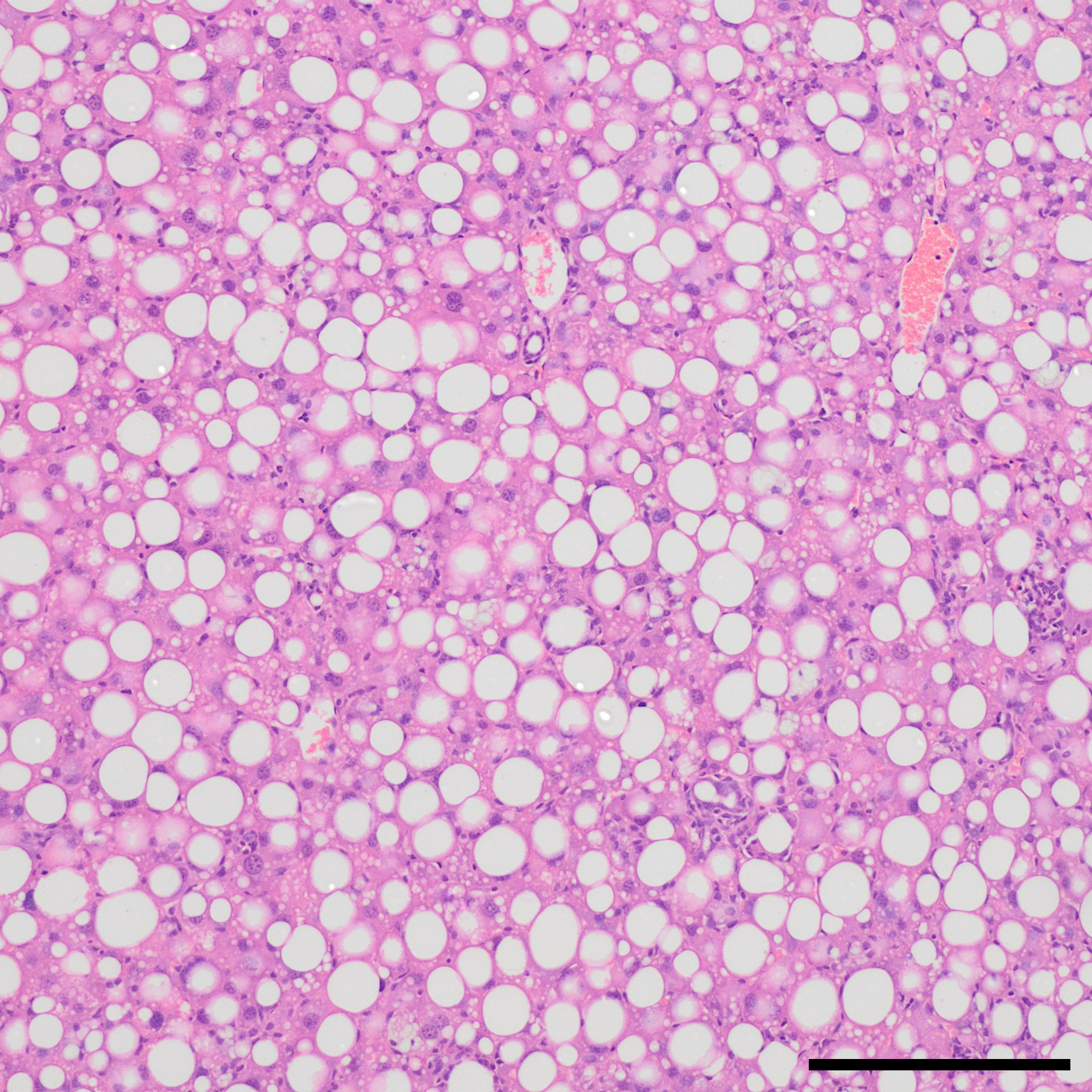


H&E


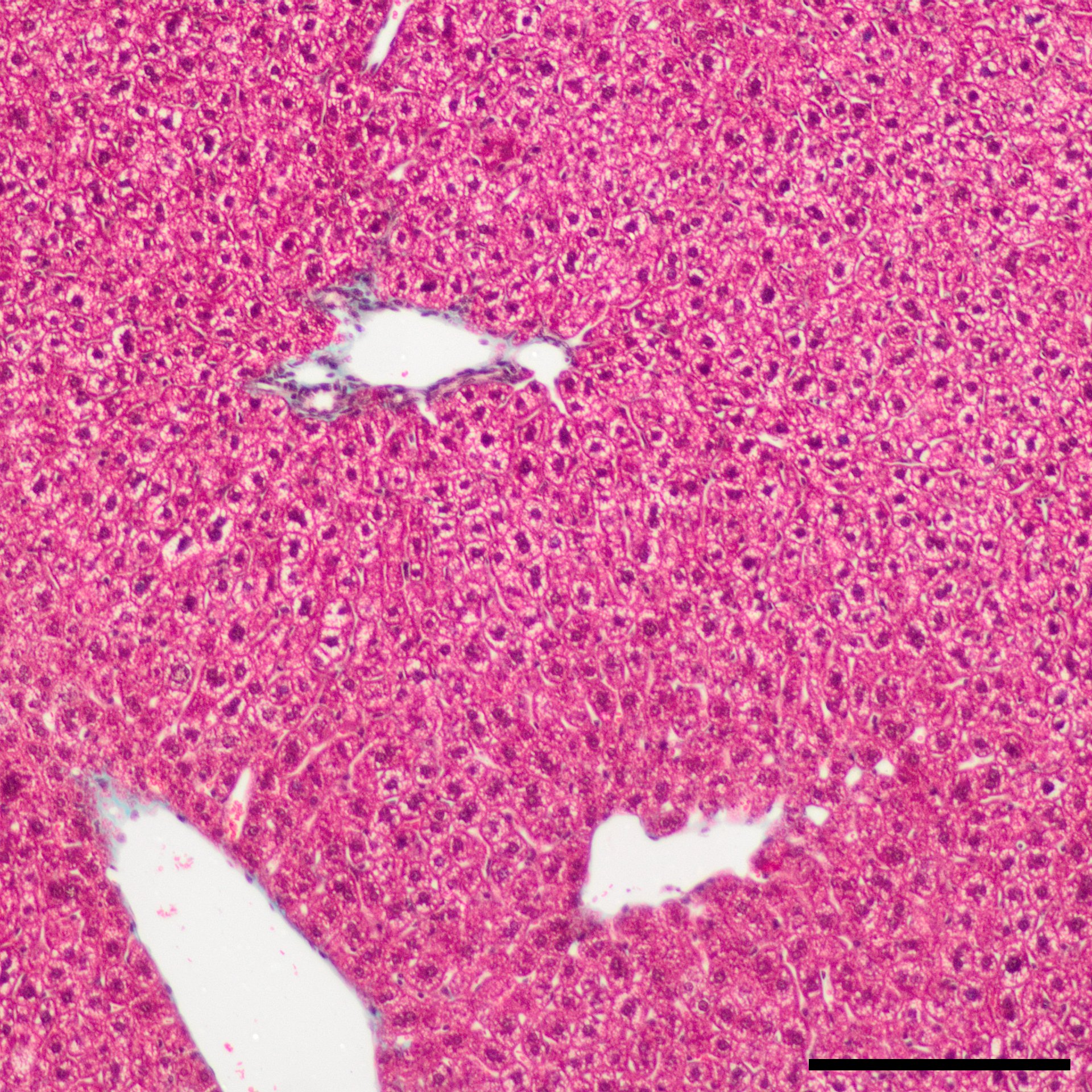

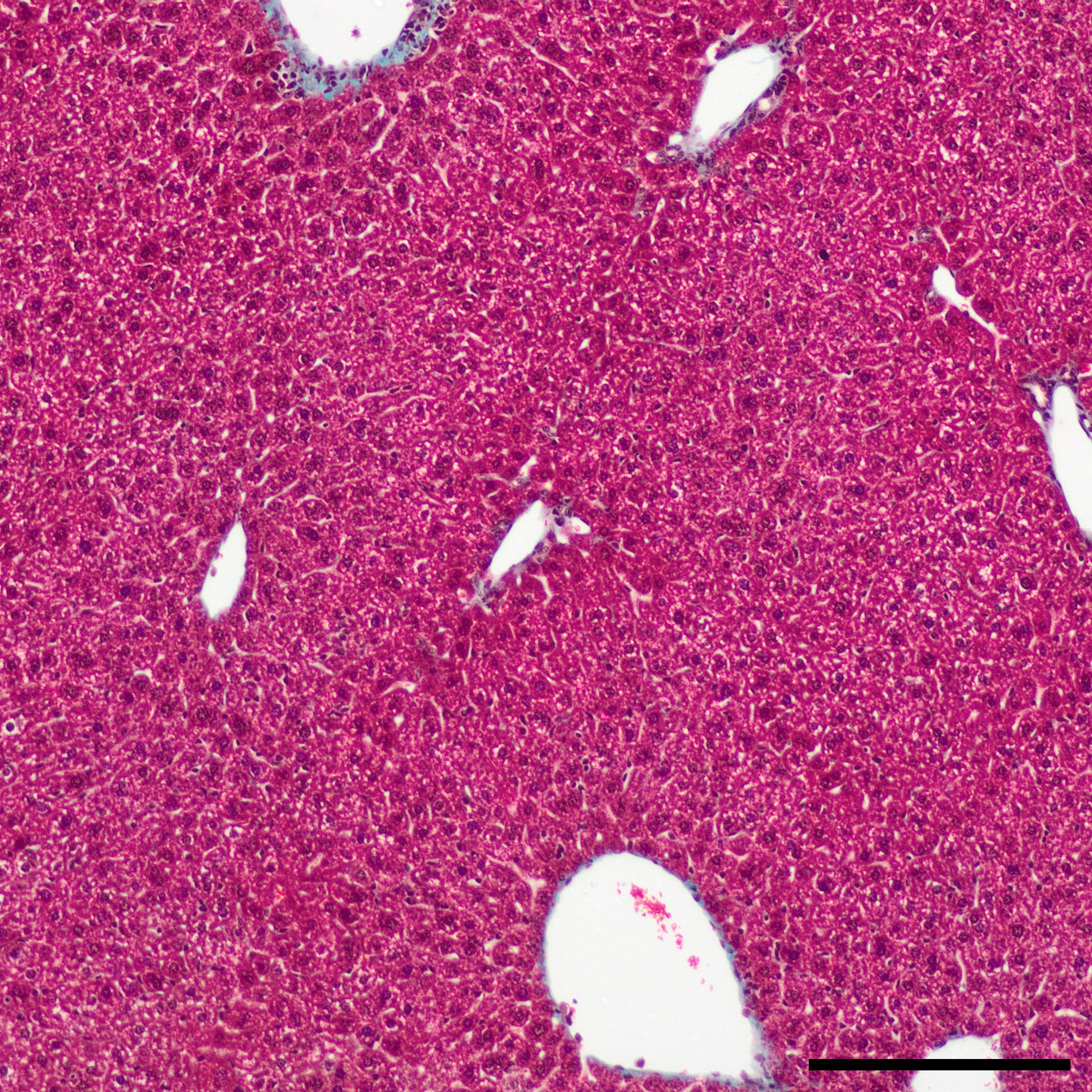

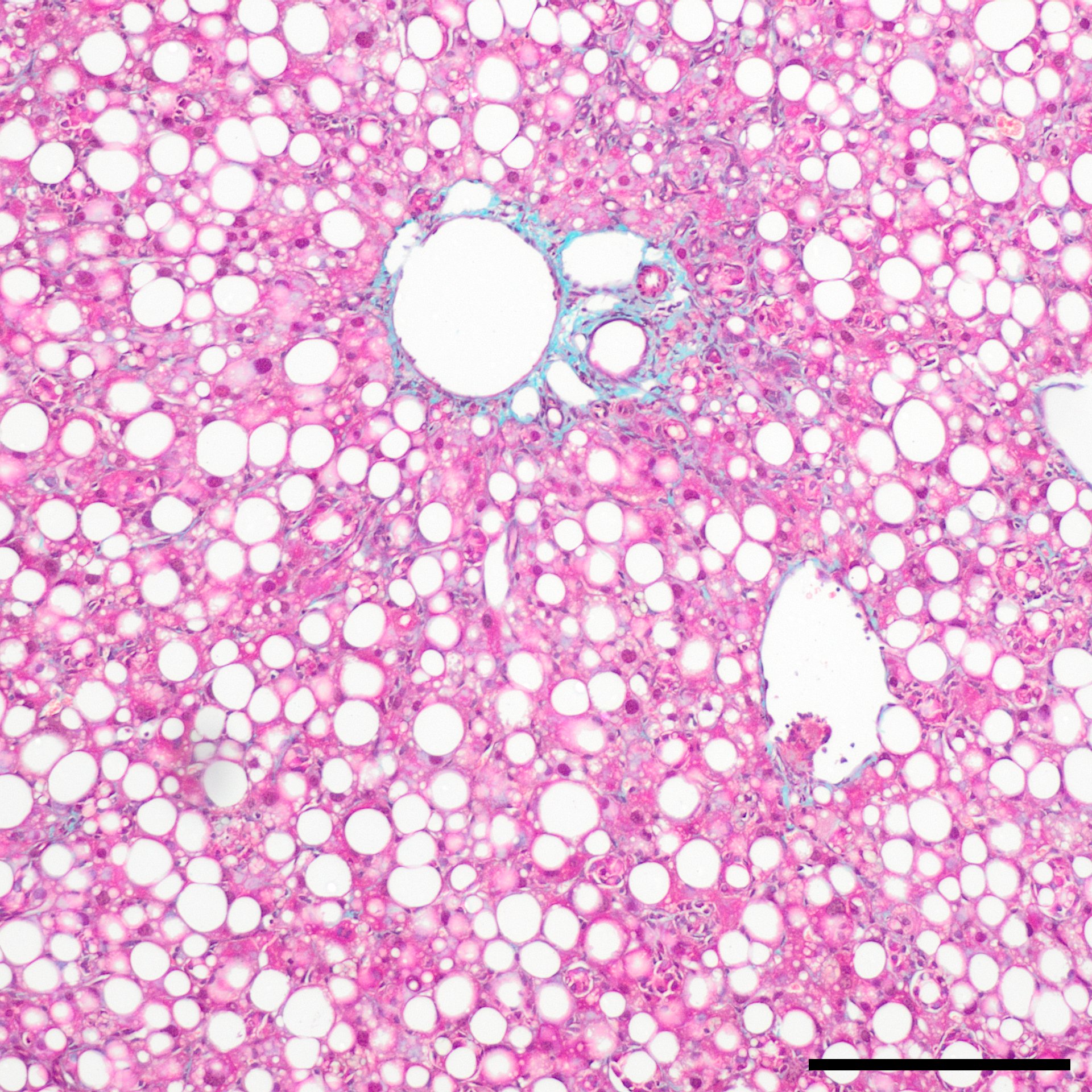

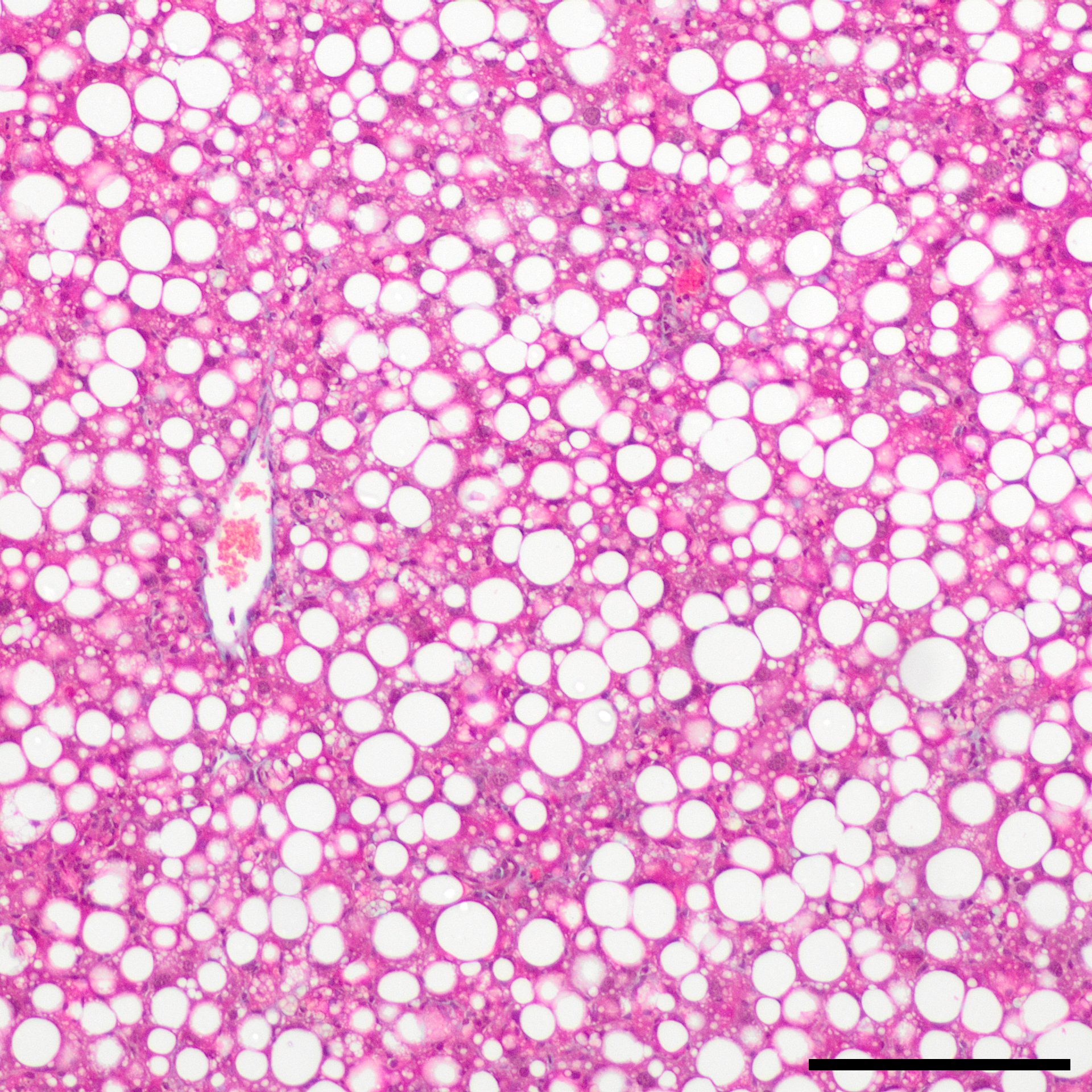


TM

D


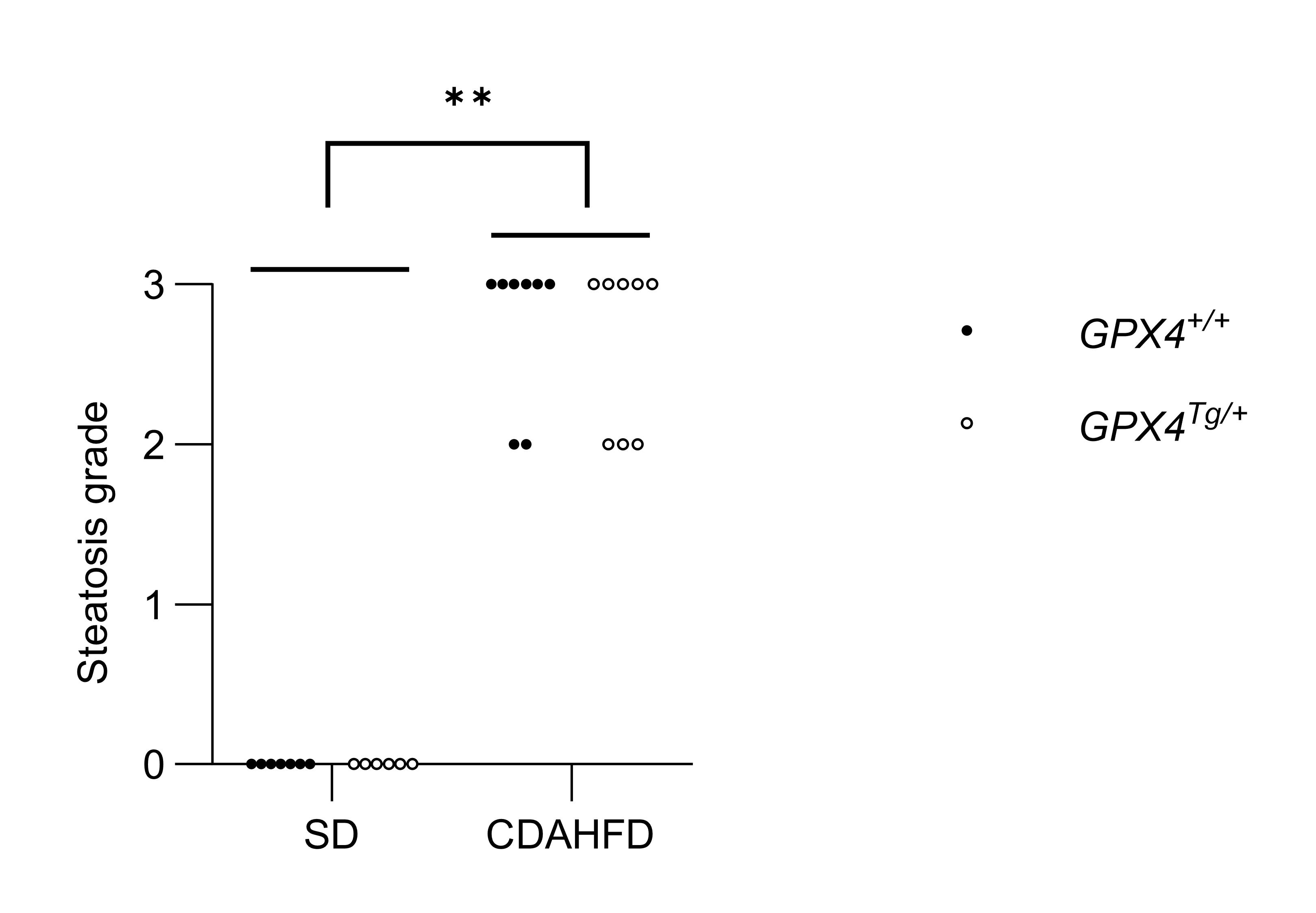

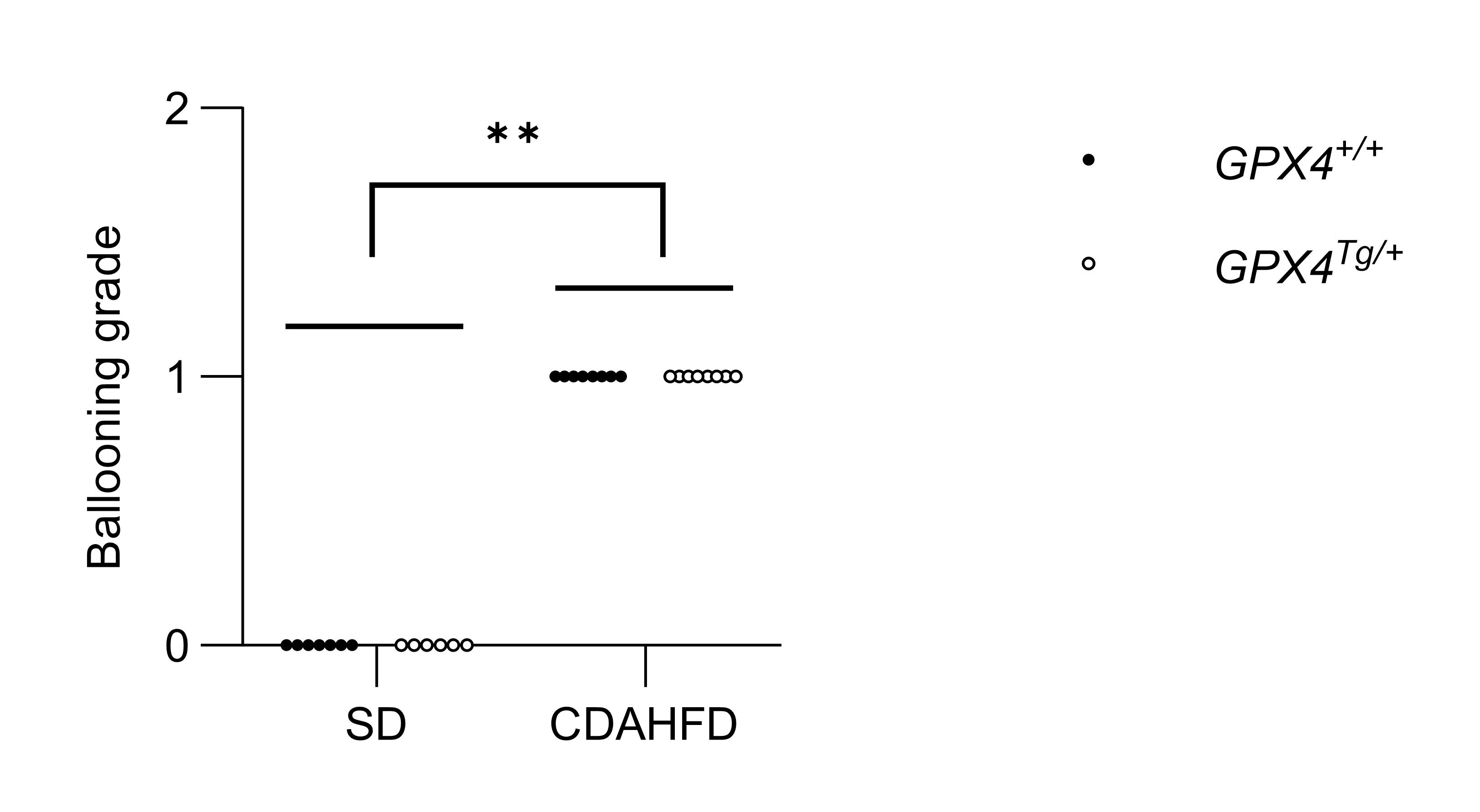

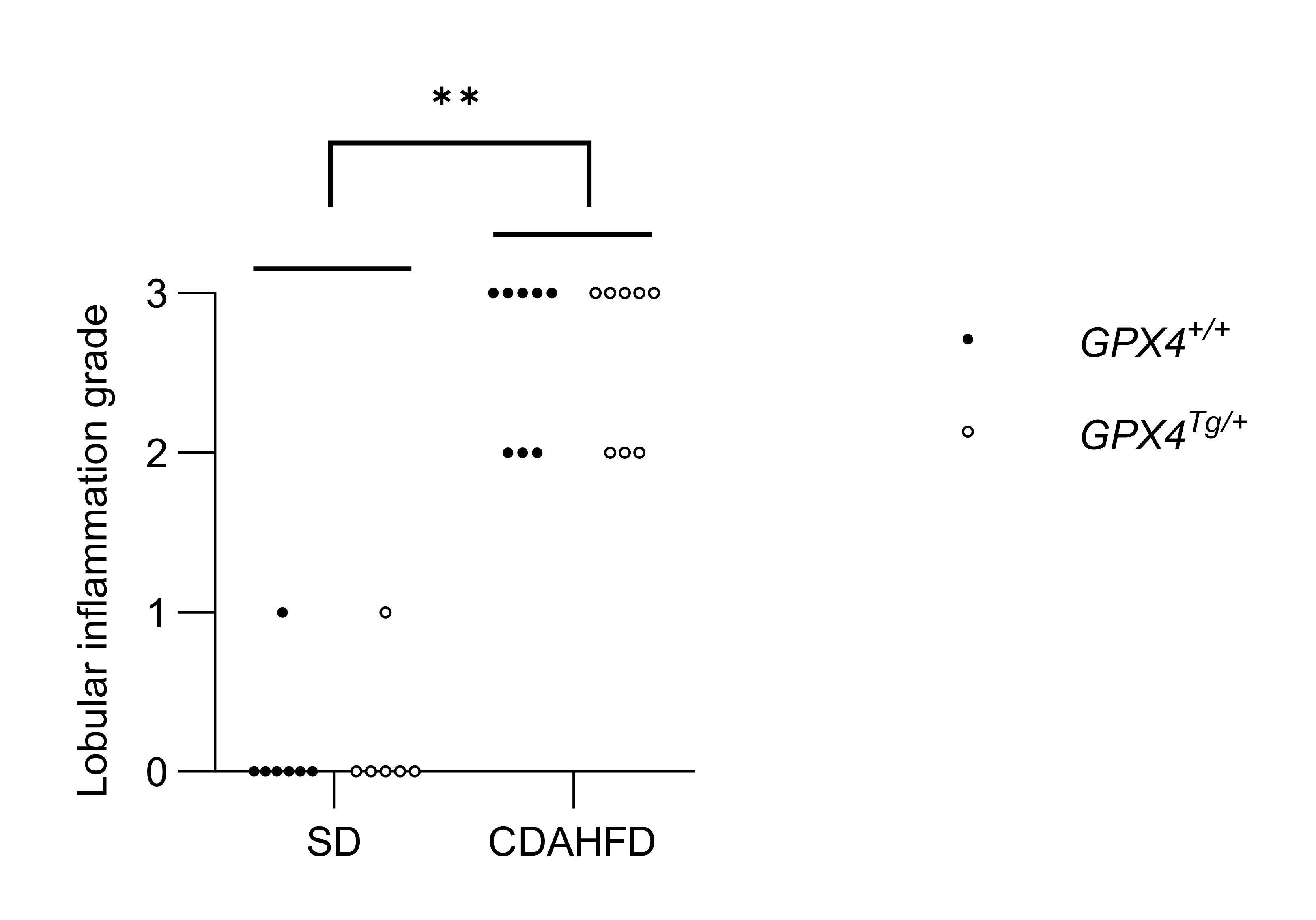

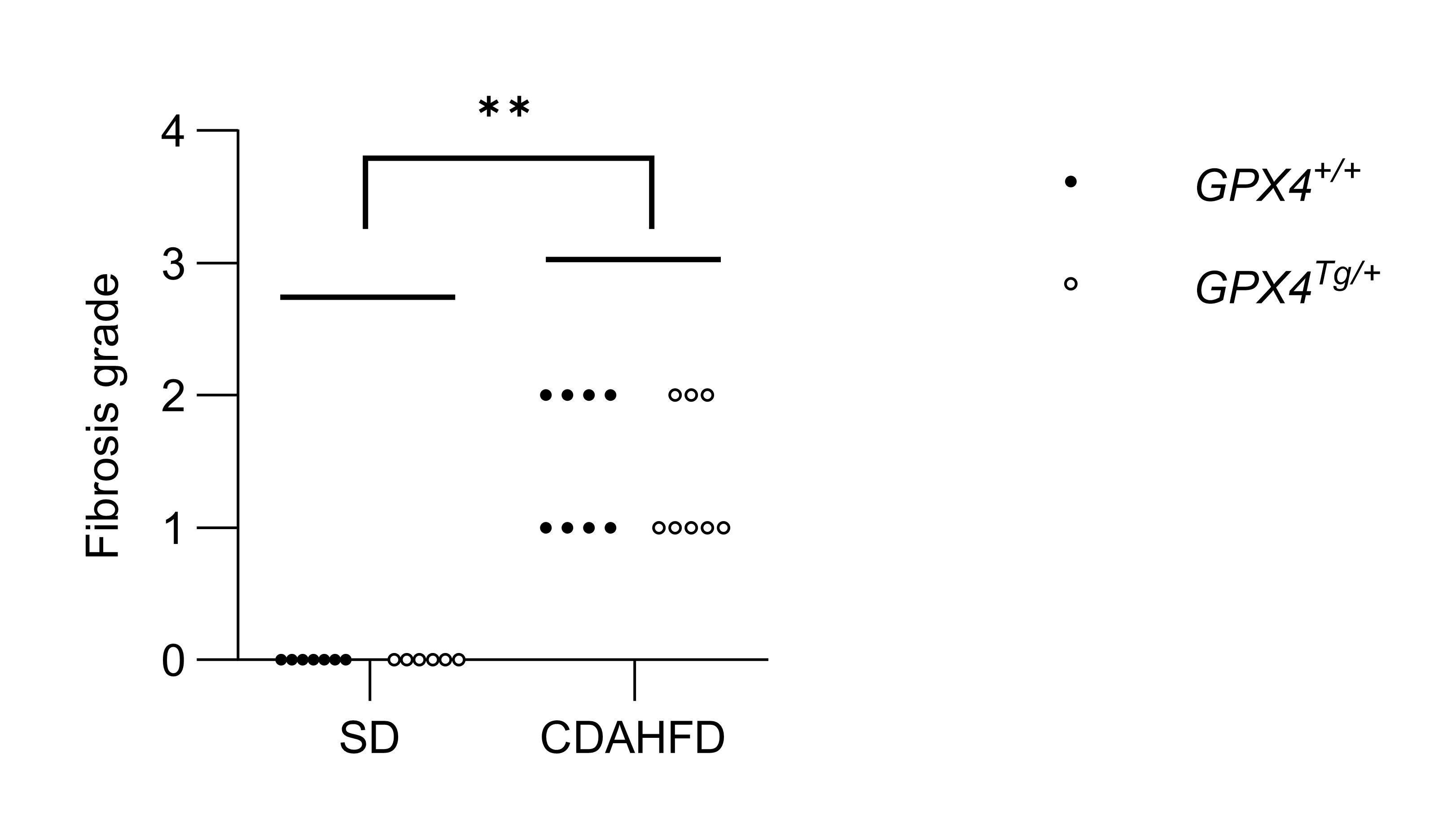


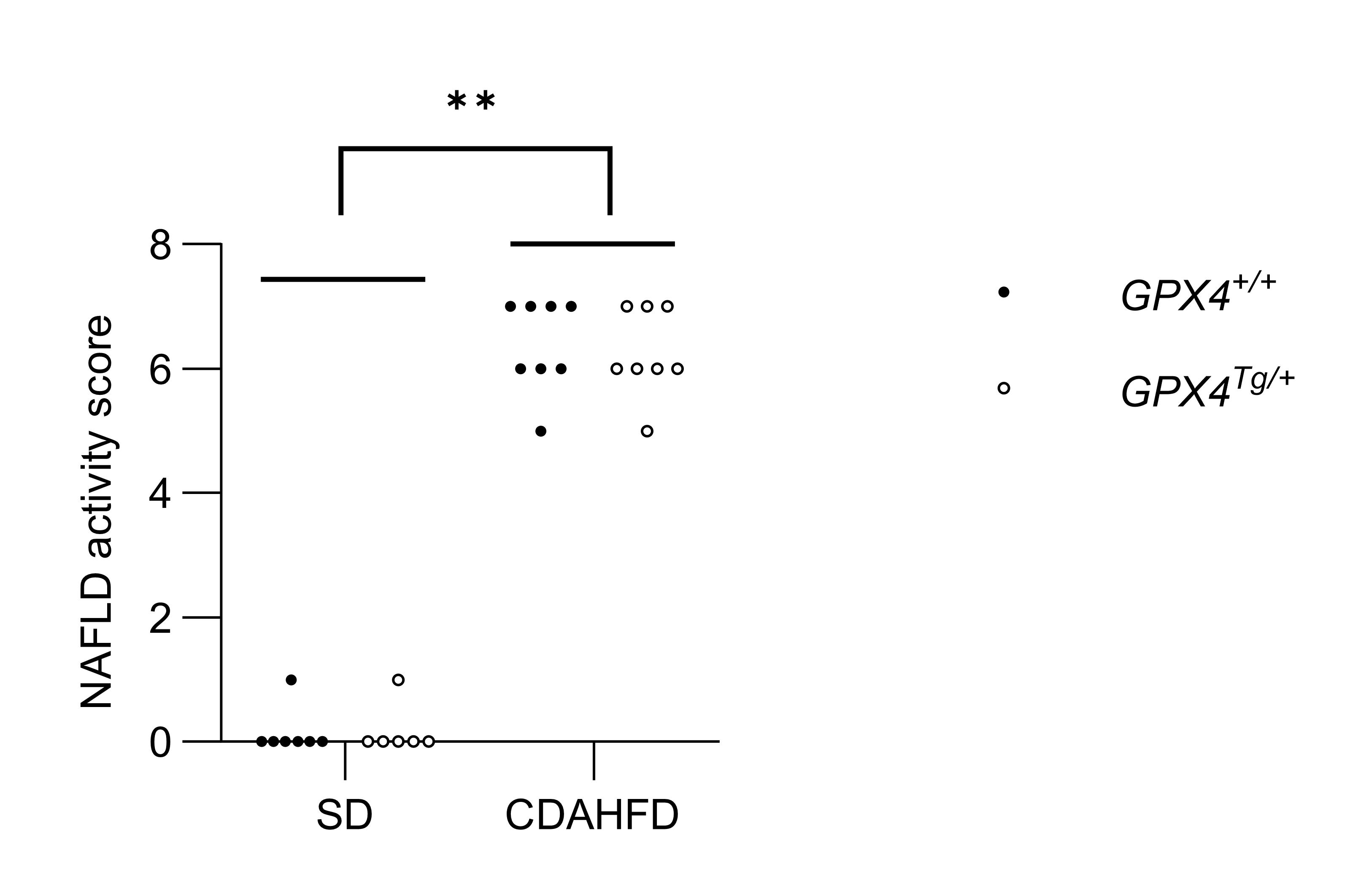

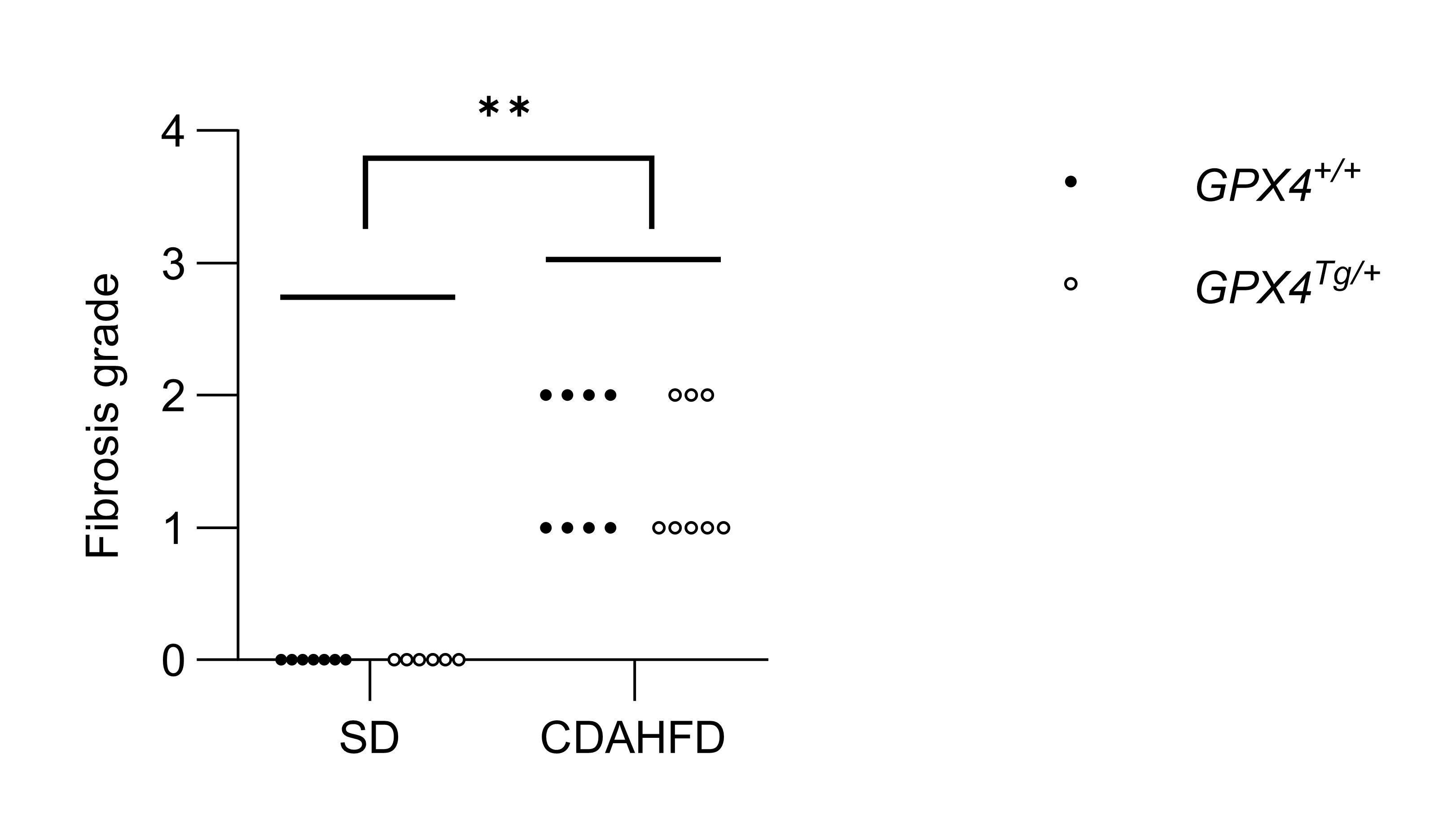

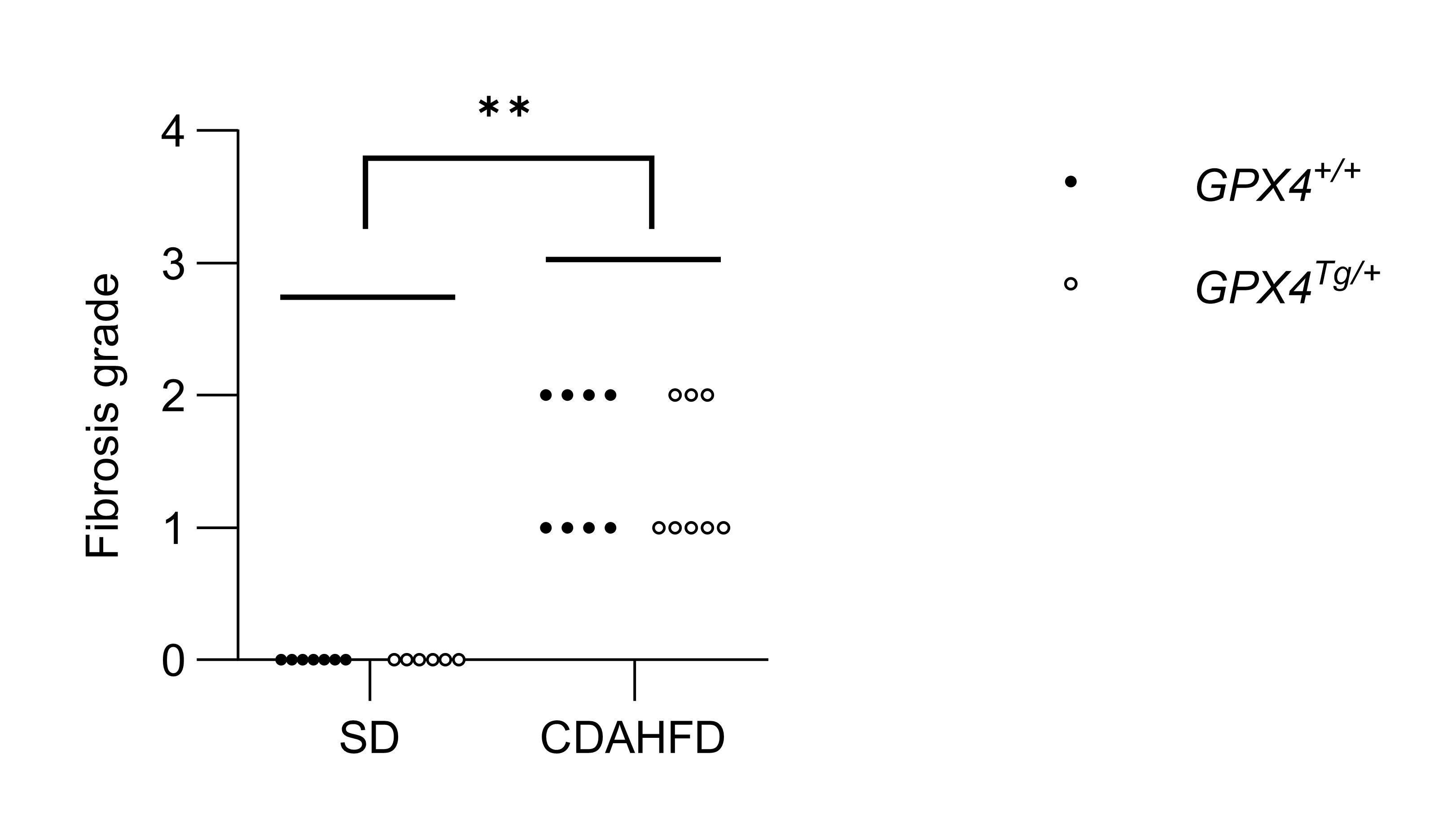


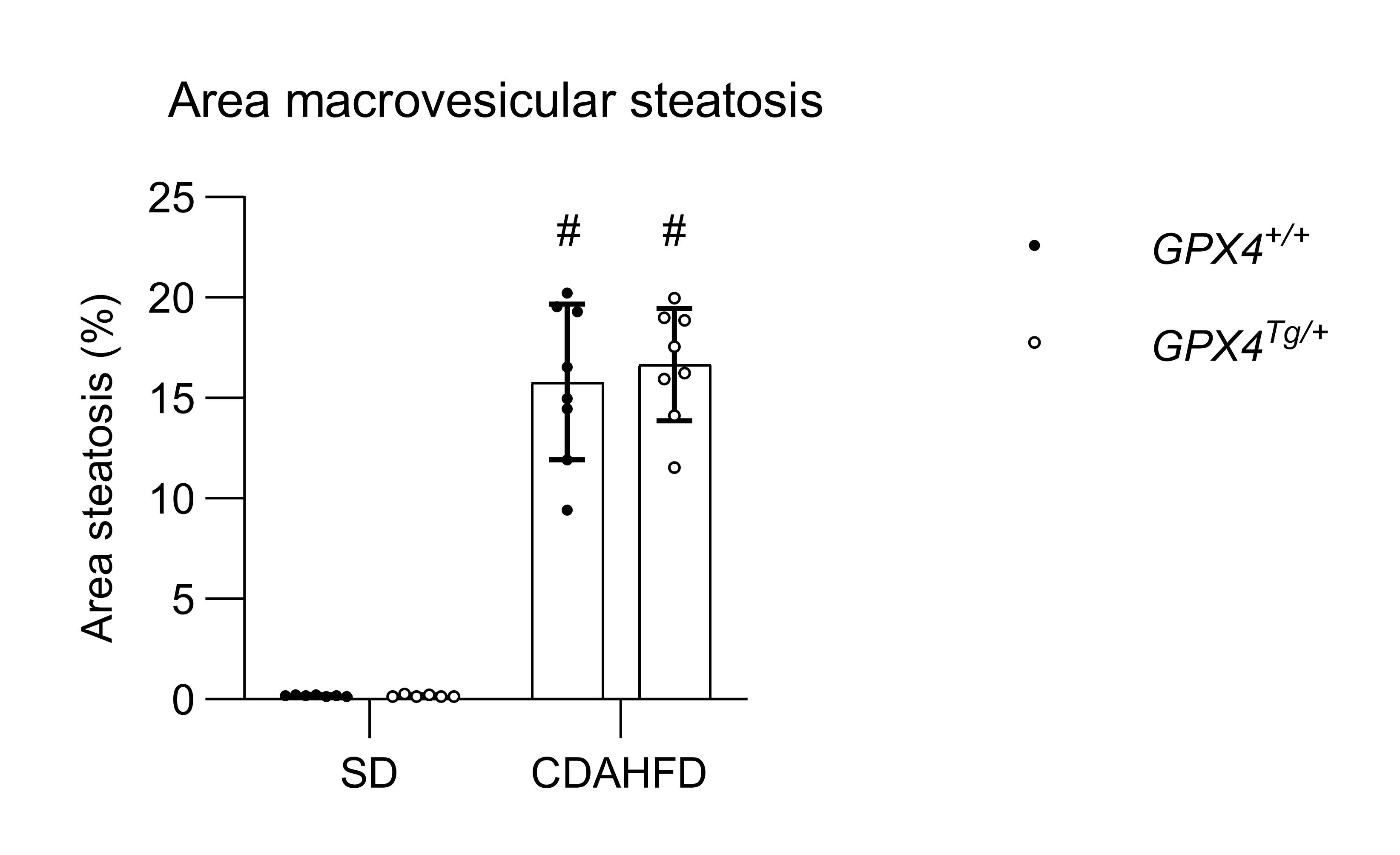

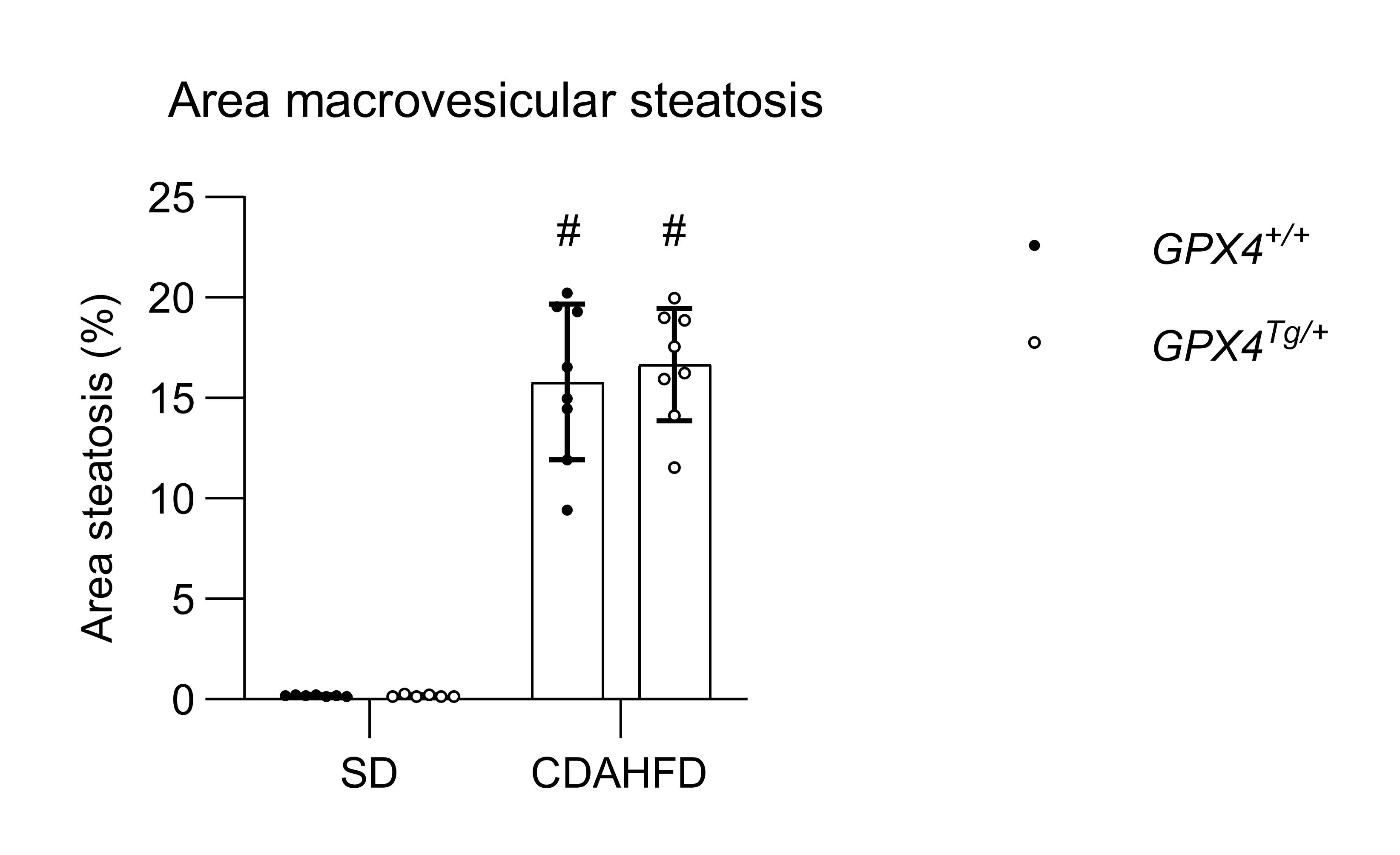


E

**Fig. S14. Effect of glutathione peroxidase 4 overexpression in CDAHFD.**

Mice with whole body glutathione peroxidase 4 overexpression (*Gpx4^Tg/+^*) and their wild-type littermates (*Gpx4^+/+^*) were fed the choline-deficient L-amino acid-defined high-fat diet (CDAHFD) or standard diet (SD) for 4 weeks. (A) Serum alanine aminotransferase (ALT) and aspartate aminotransferase (AST) levels. (B) Liver-on-bodyweight ratio as a measure for hepatomegaly. (C) Representative images of hematoxylin & eosin (H&E) and Masson’s trichome stains in the 4 experimental groups. (D) Scoring of histologic features of steatosis, ballooning, lobular inflammation, NAFLD activity score and fibrosis. (E) Quantification of liver area enveloped by macrovesicular steatosis. Data are presented as mean ± SD (n = 6-8 per group). Two-way ANOVA with post-hoc test if appropriate. **p <0.01; # p<0.05 for the factor diet CDAHFD versus SD. Kruskal-Wallis test with post-hoc testing for ordinal histologic scoring.


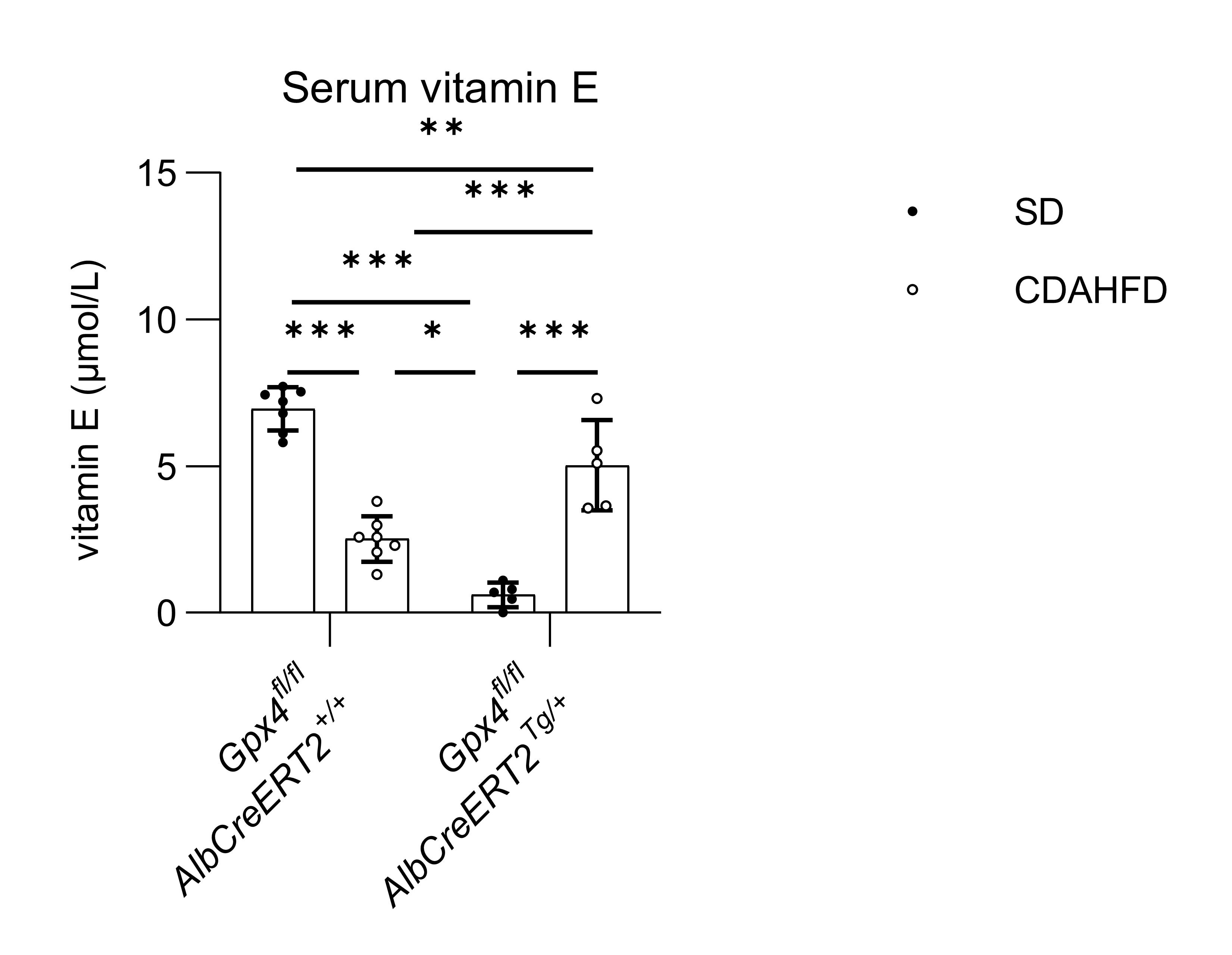
Figure S15

A


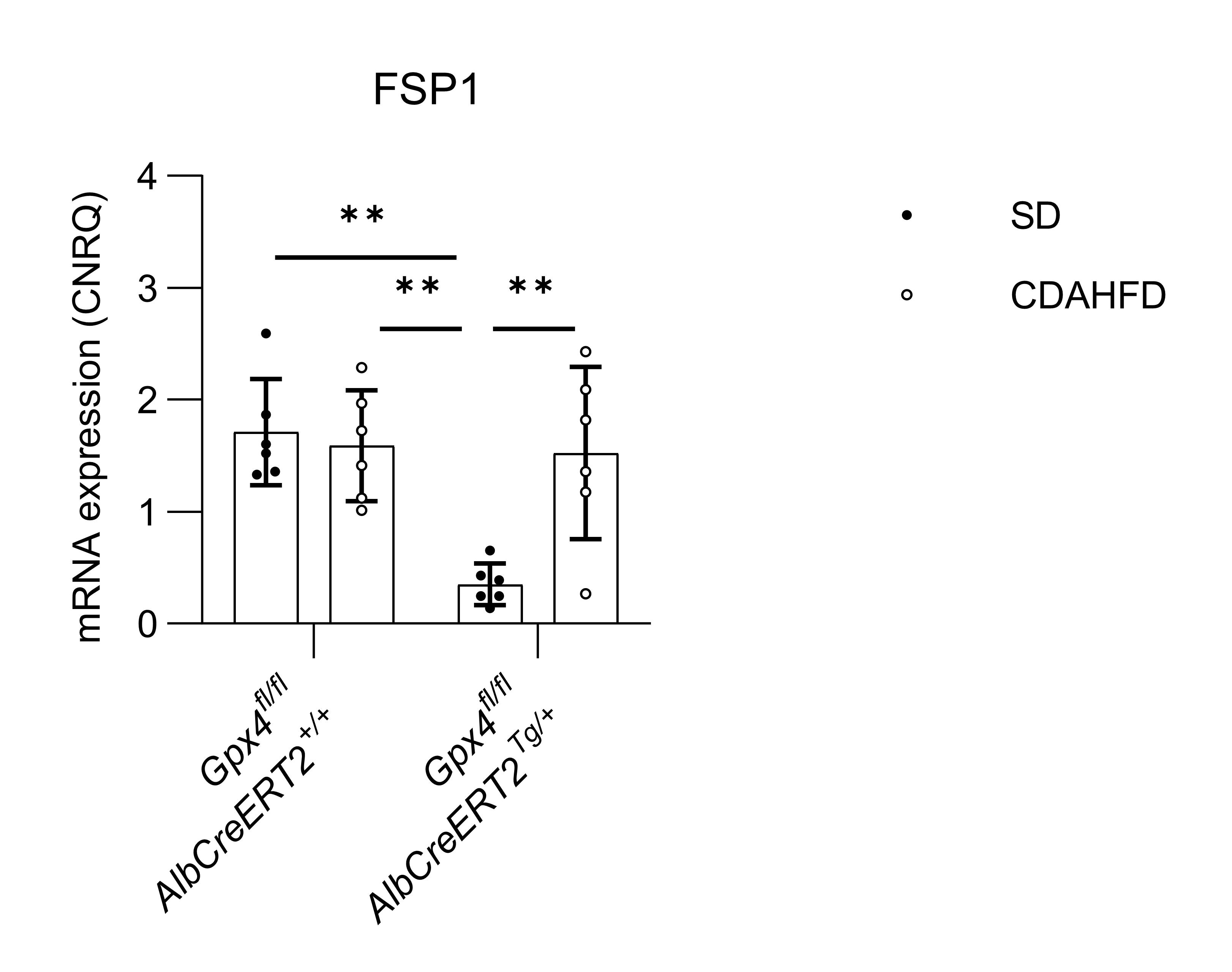

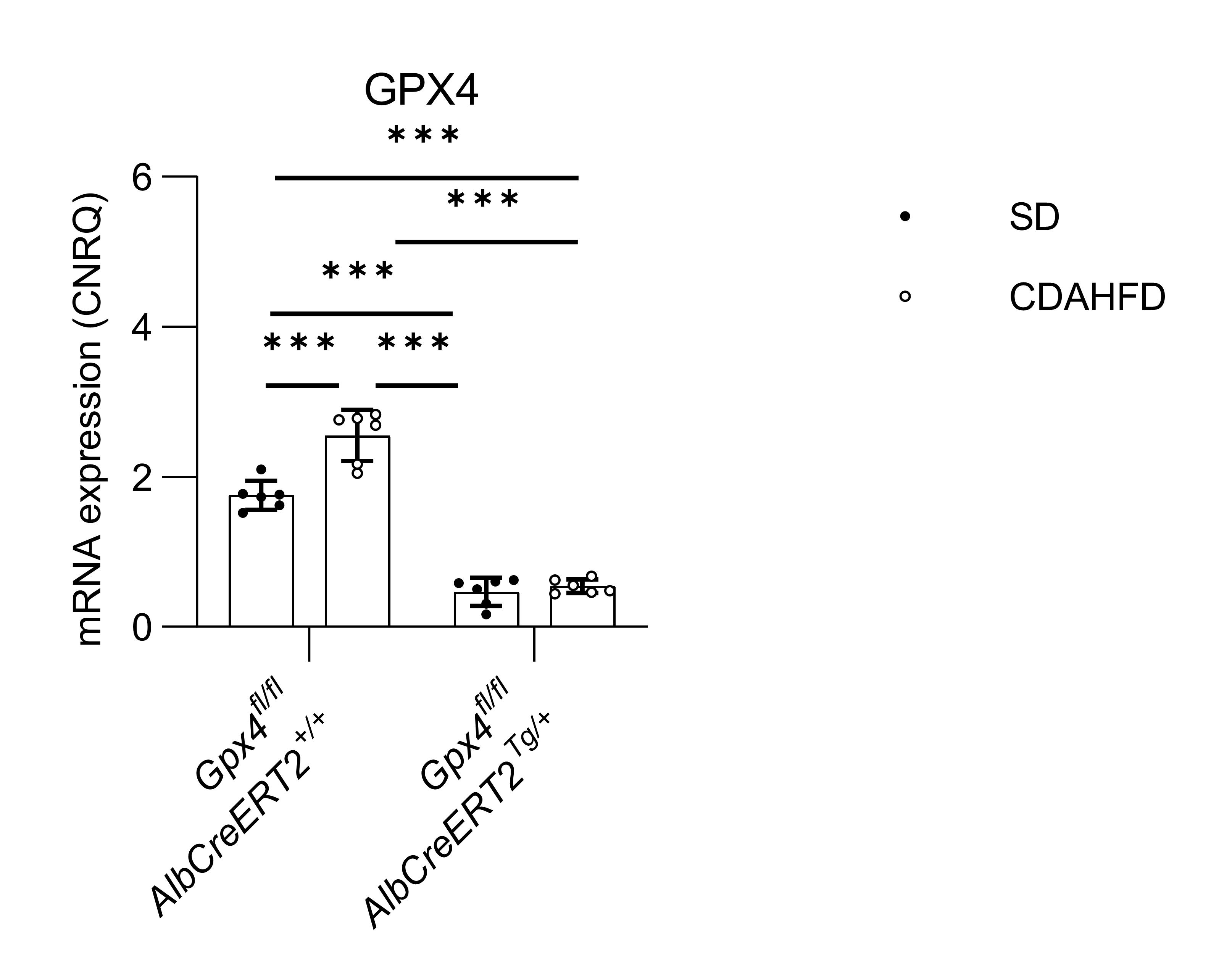

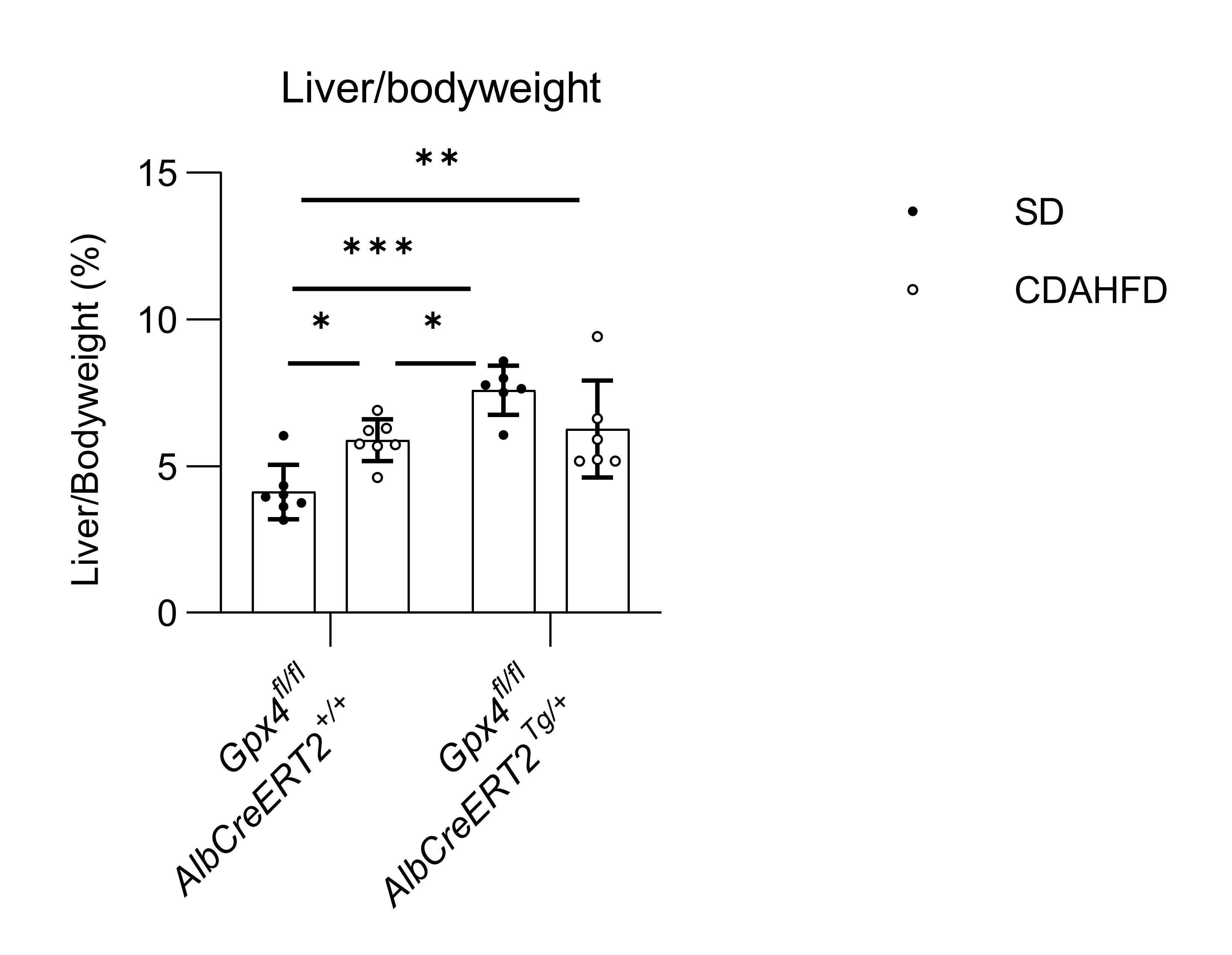


B

C


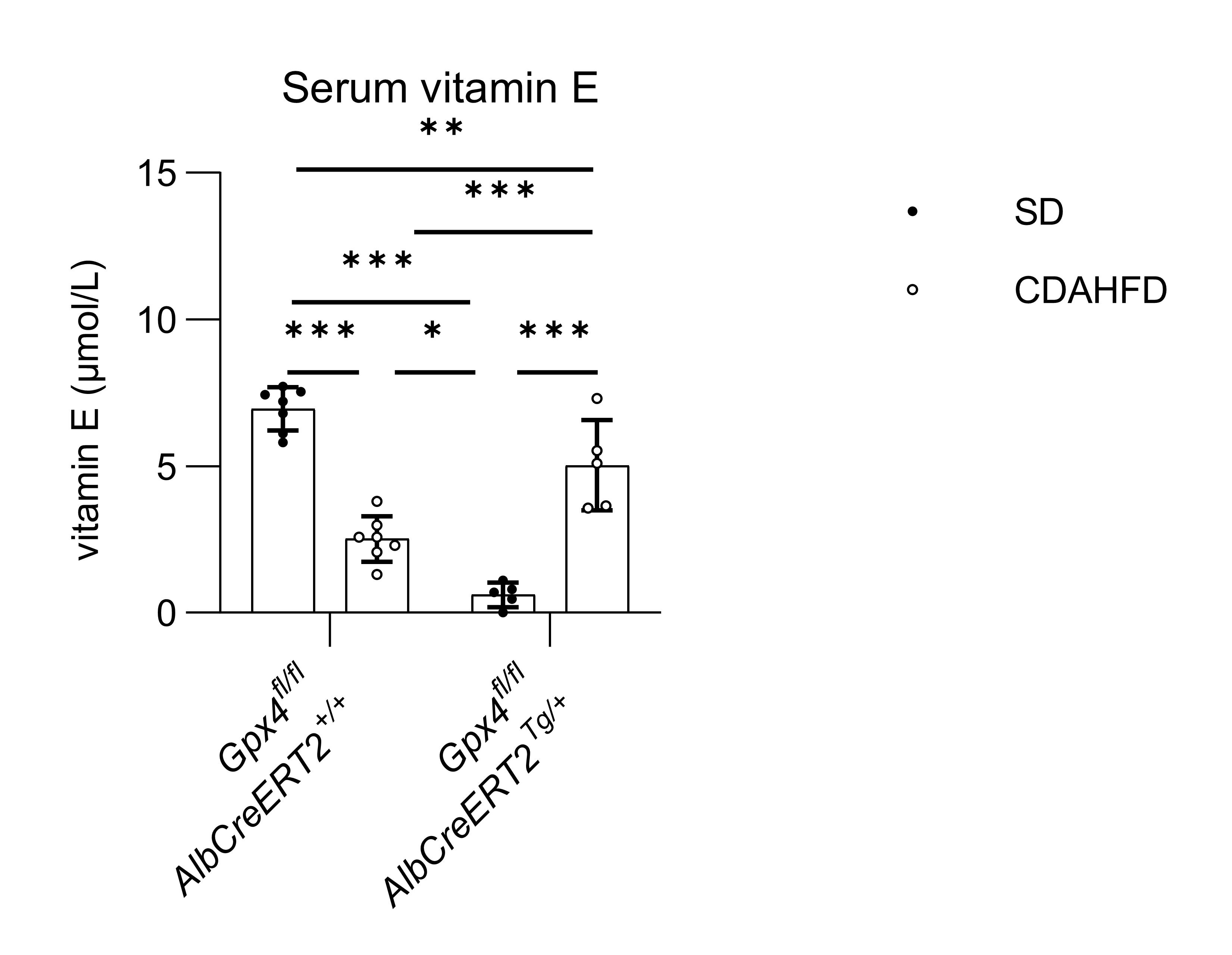

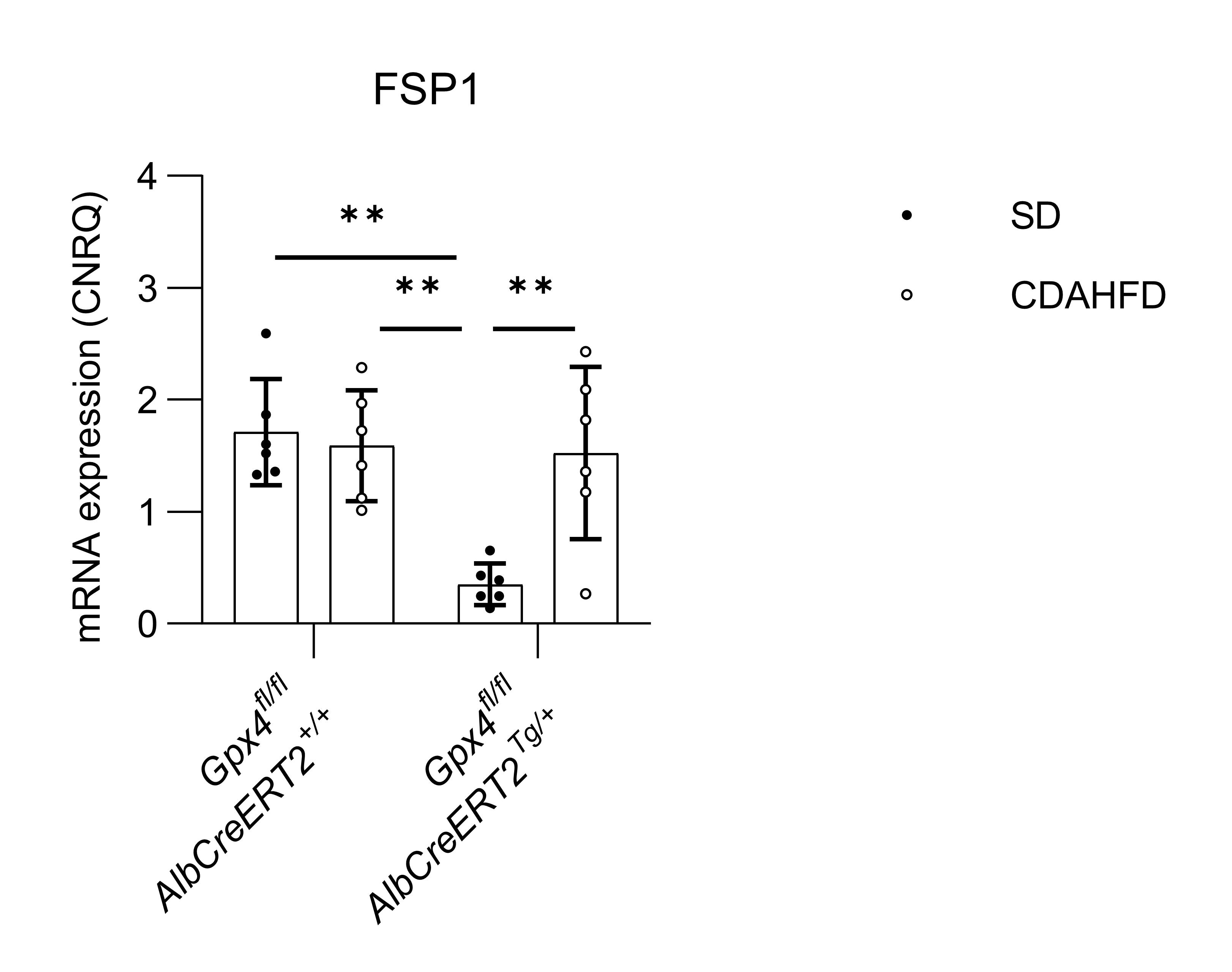

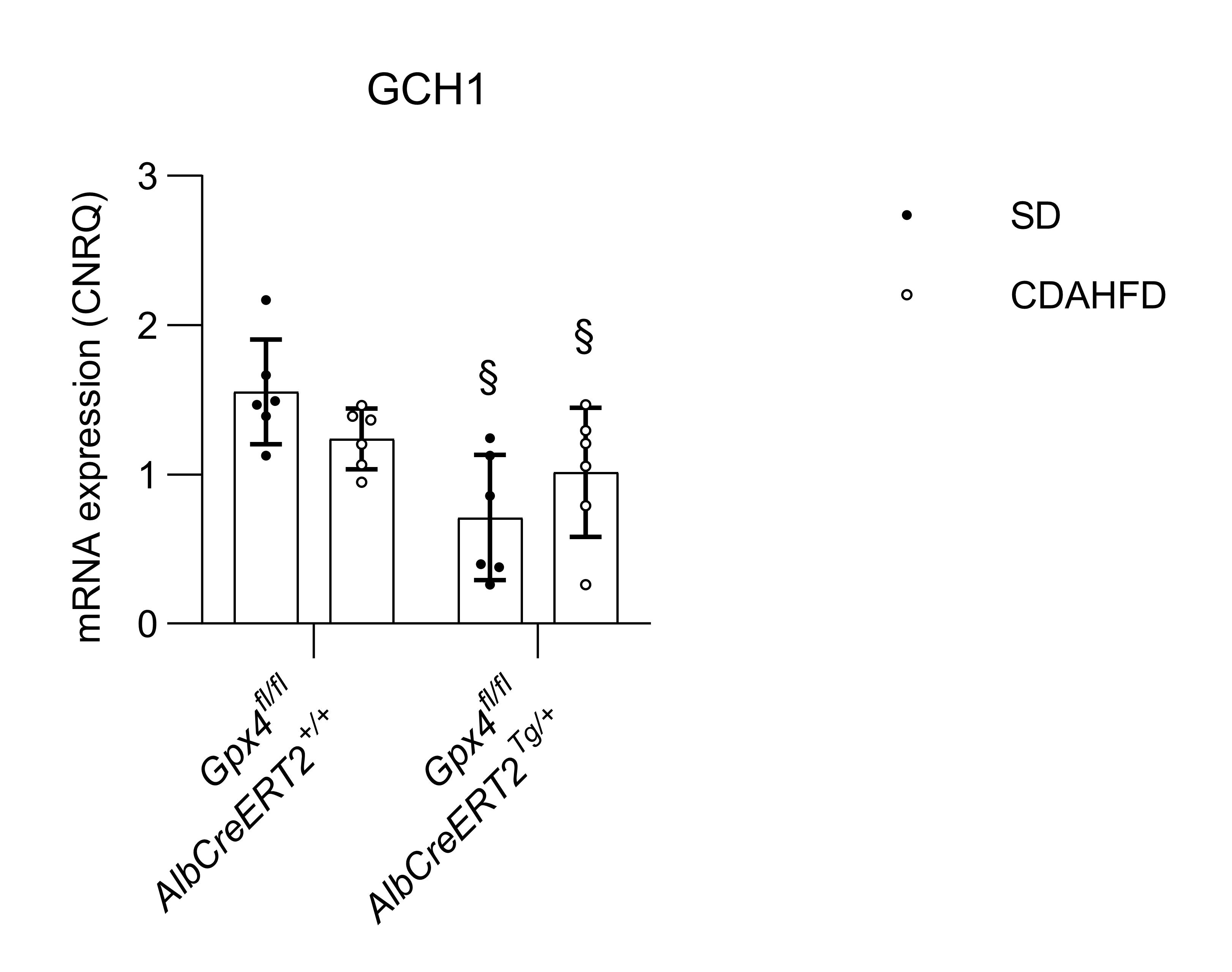


D


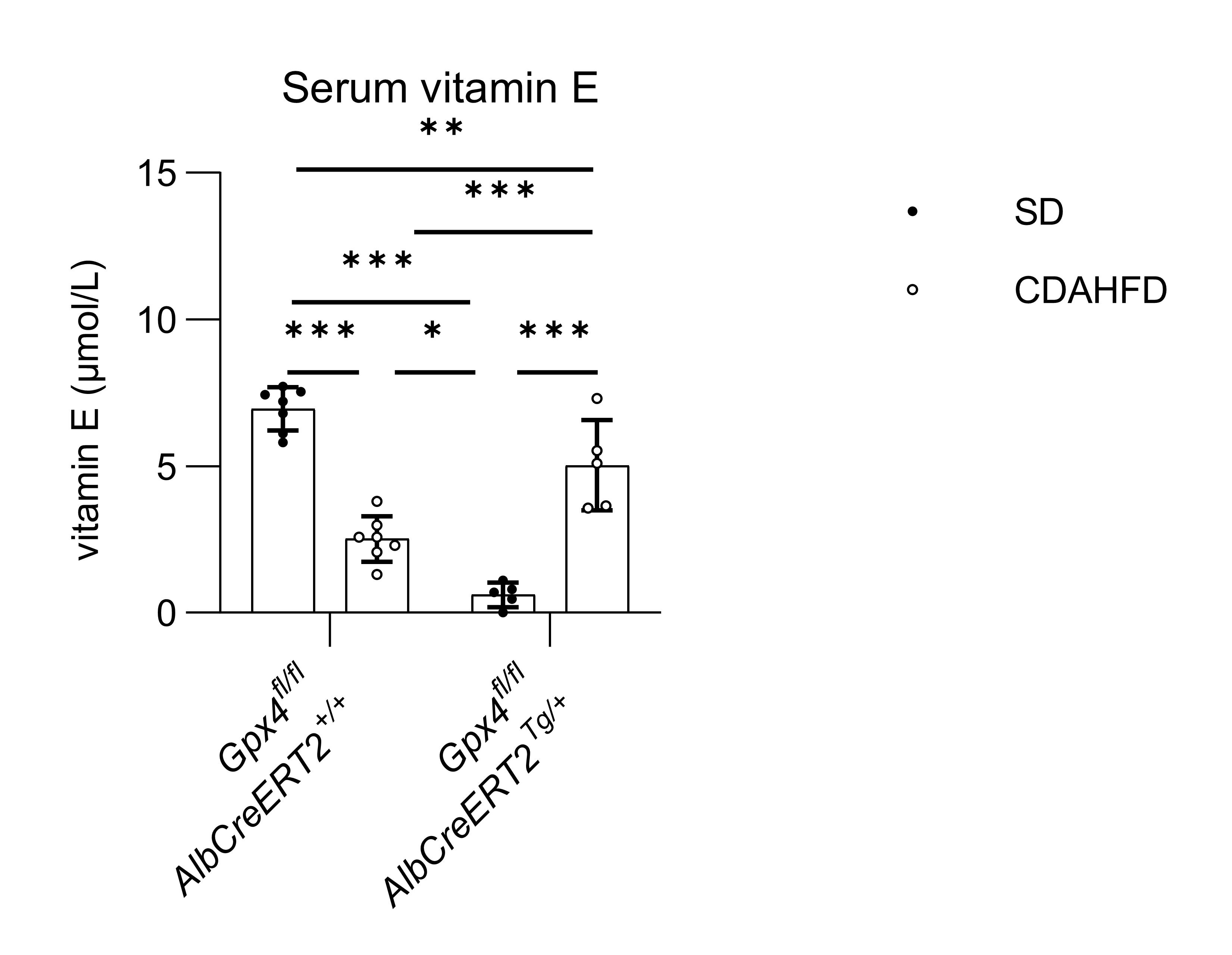


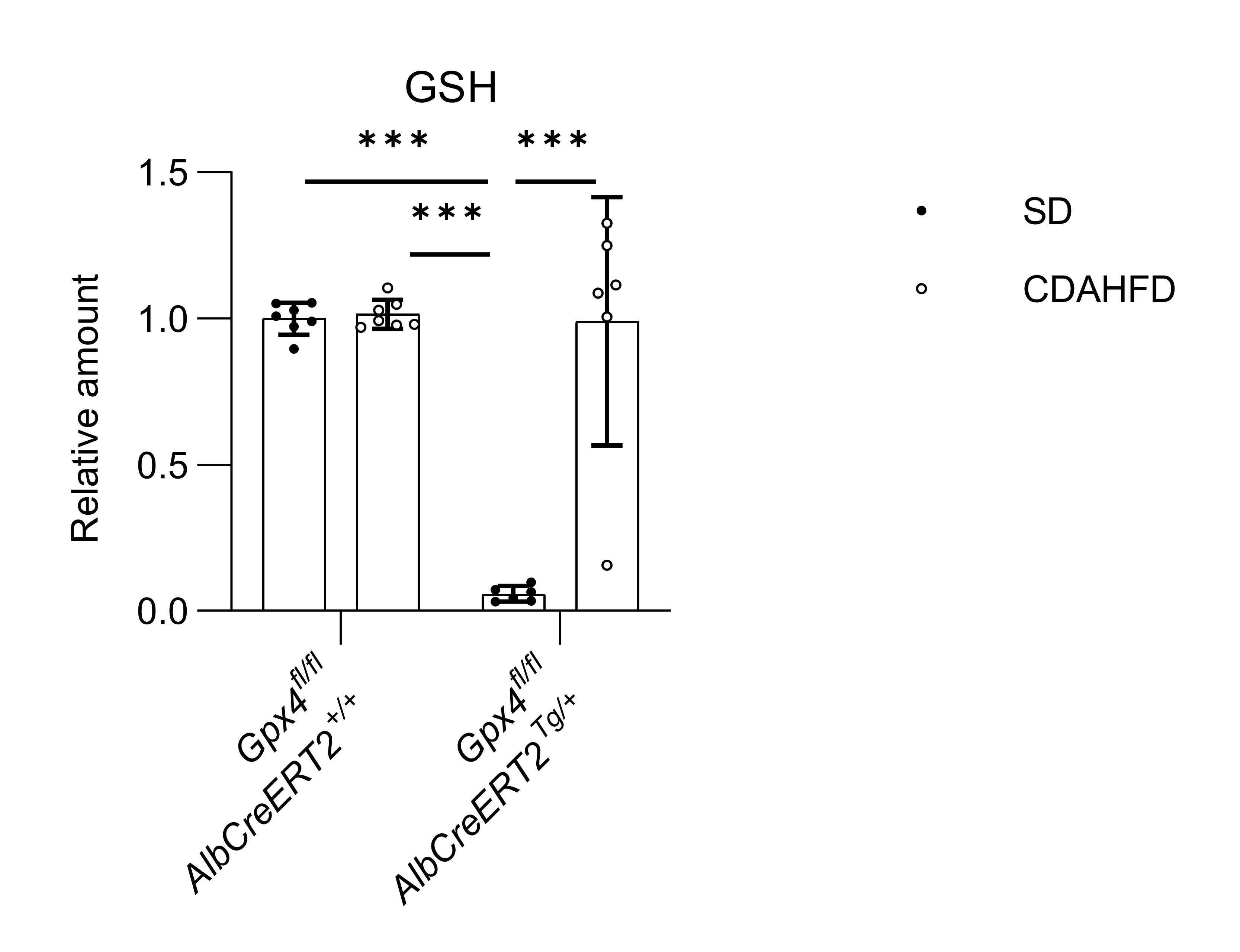

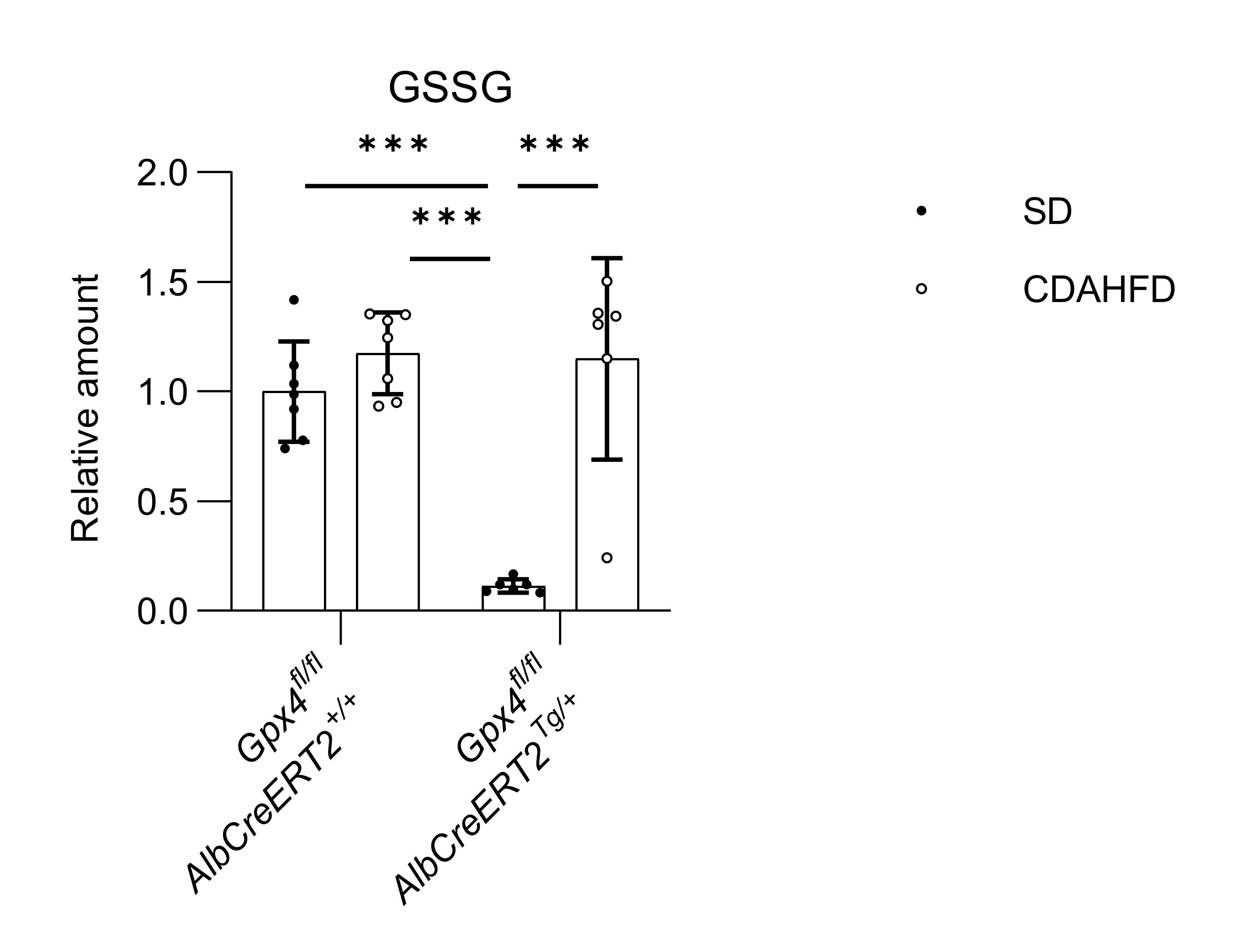

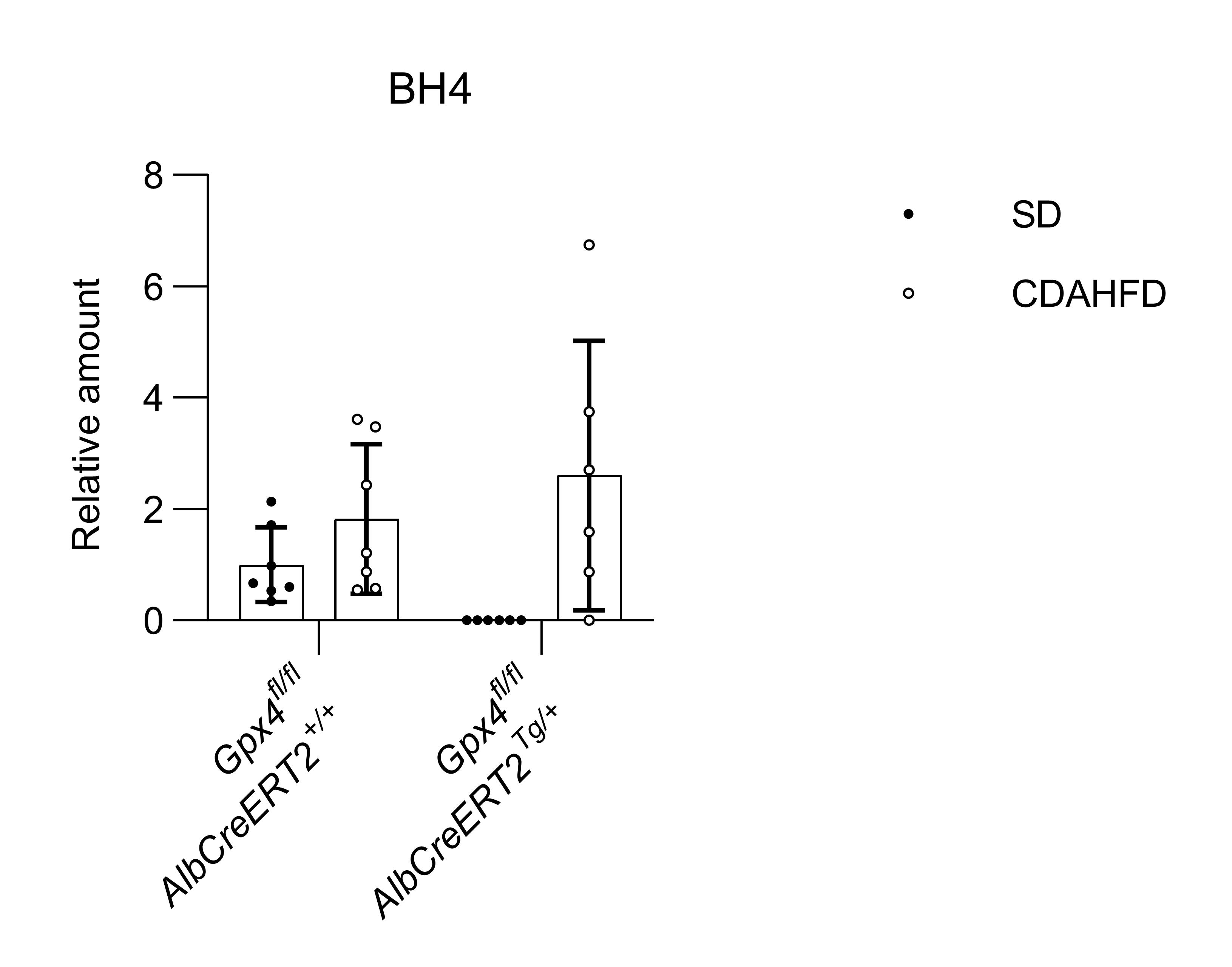

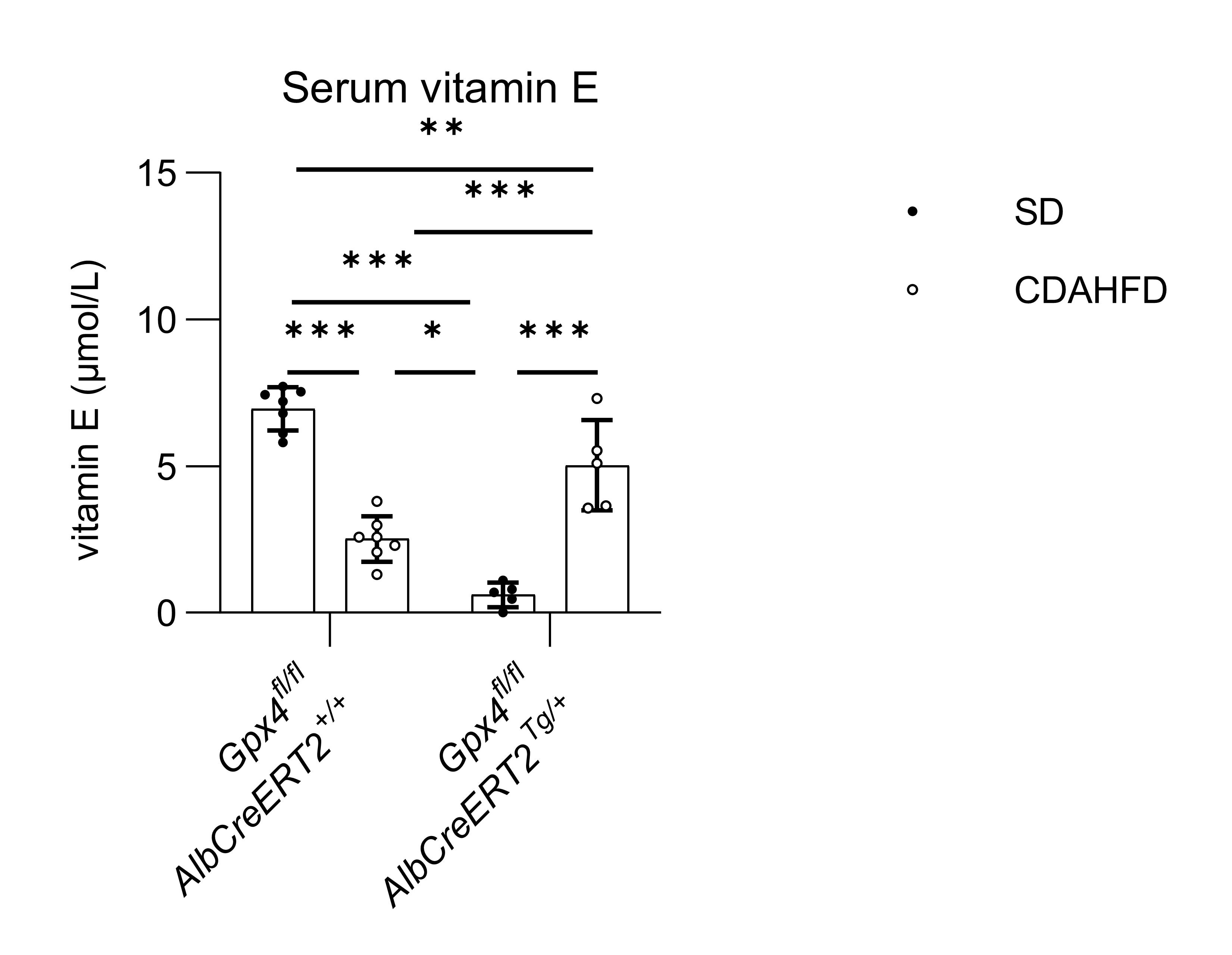


E


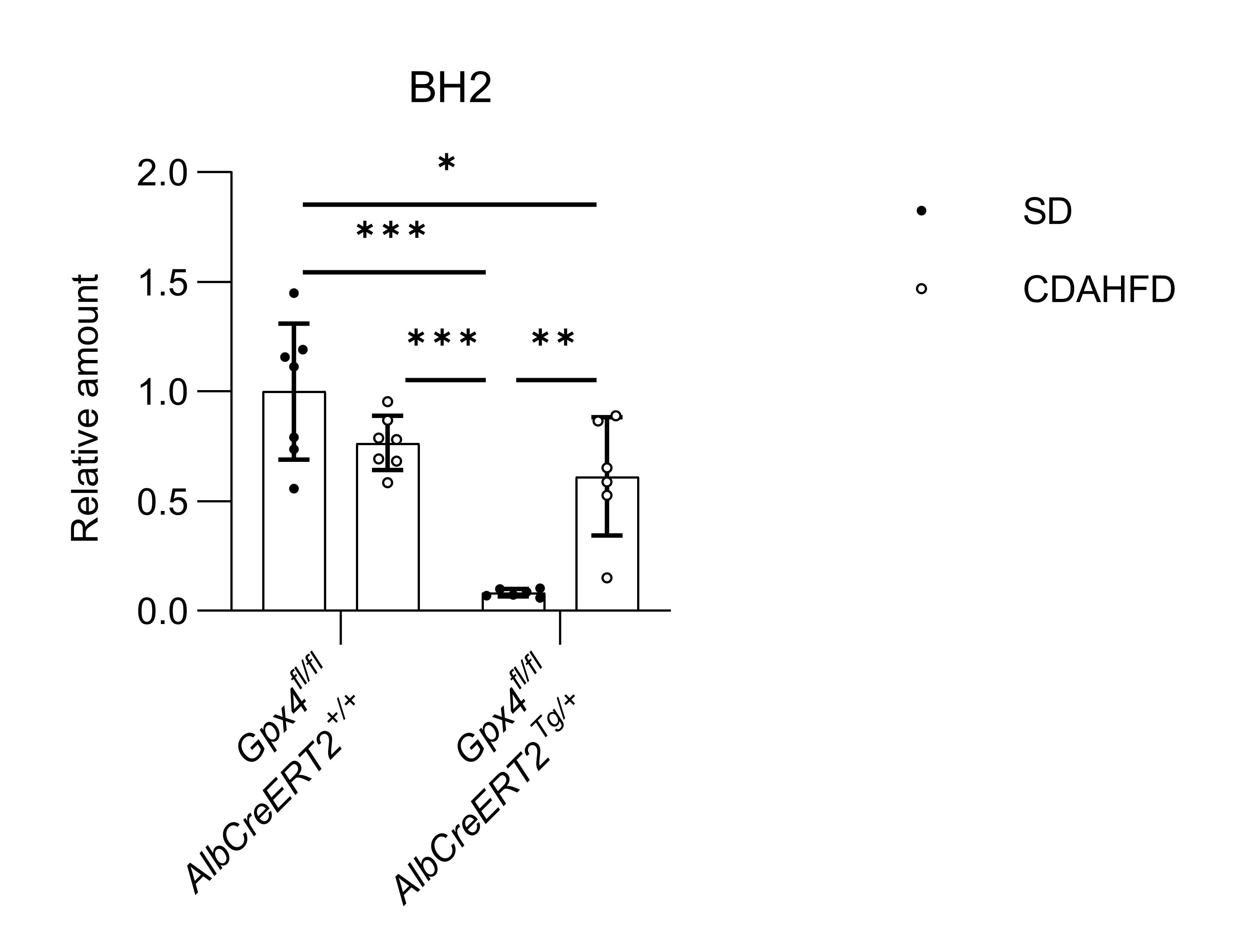

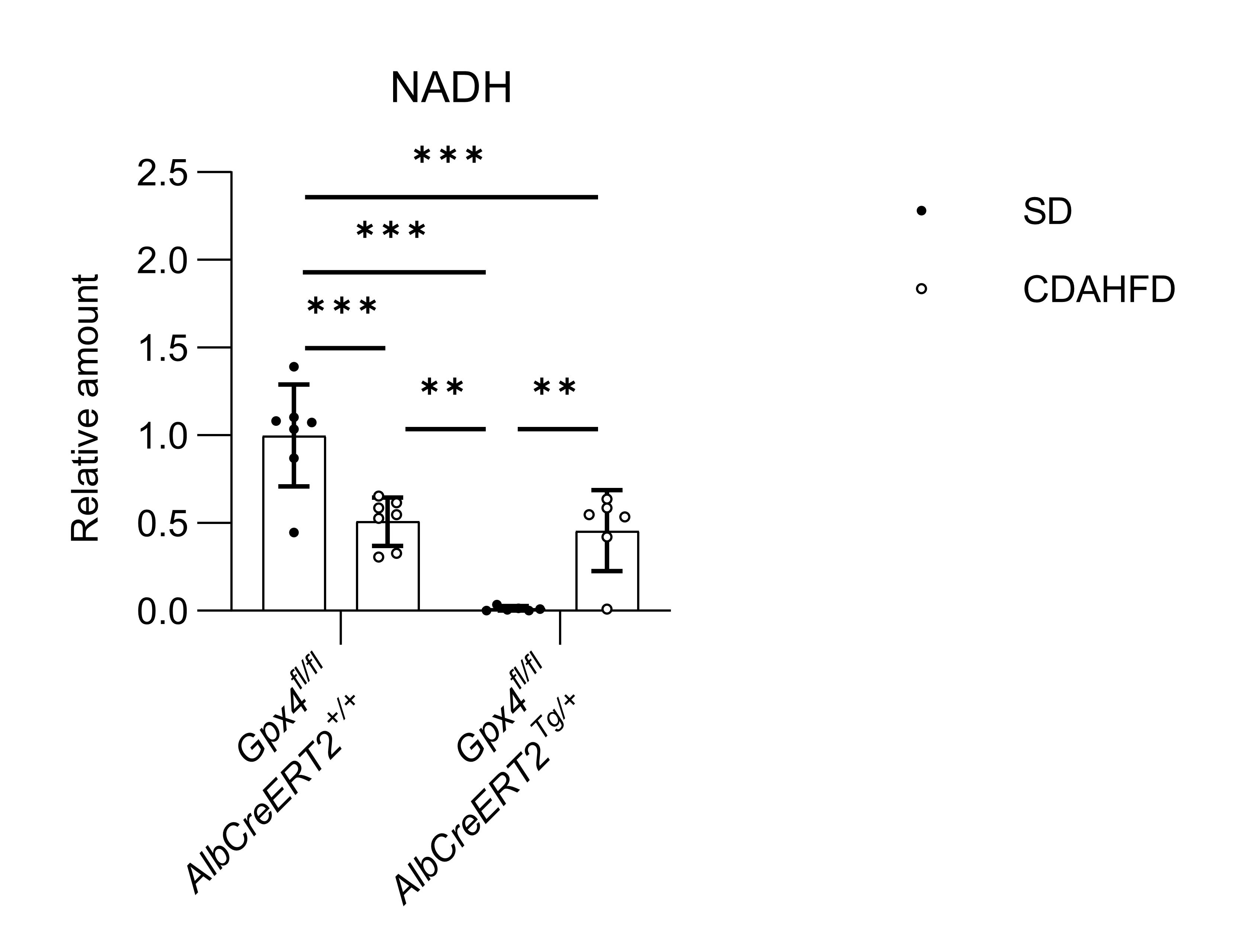

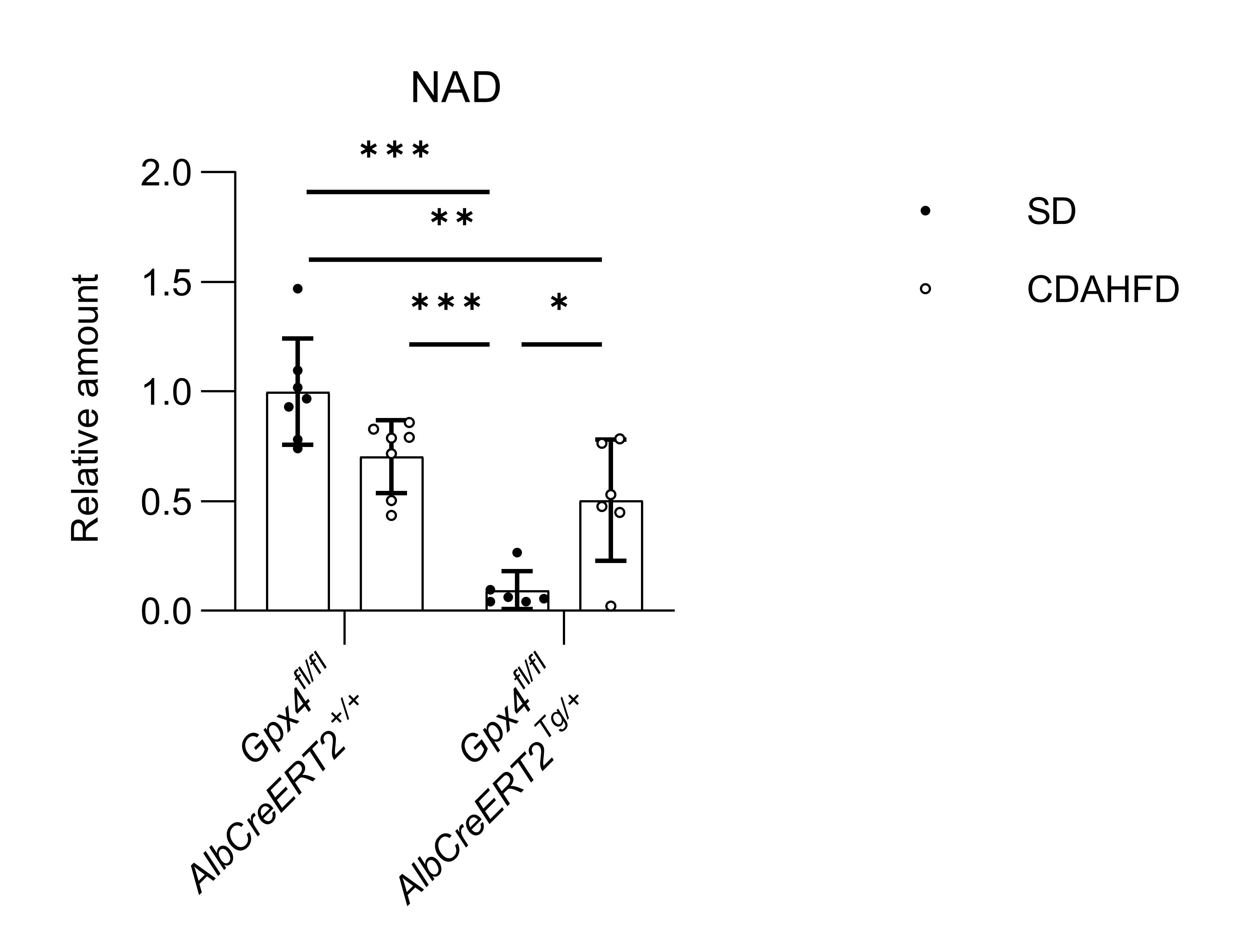

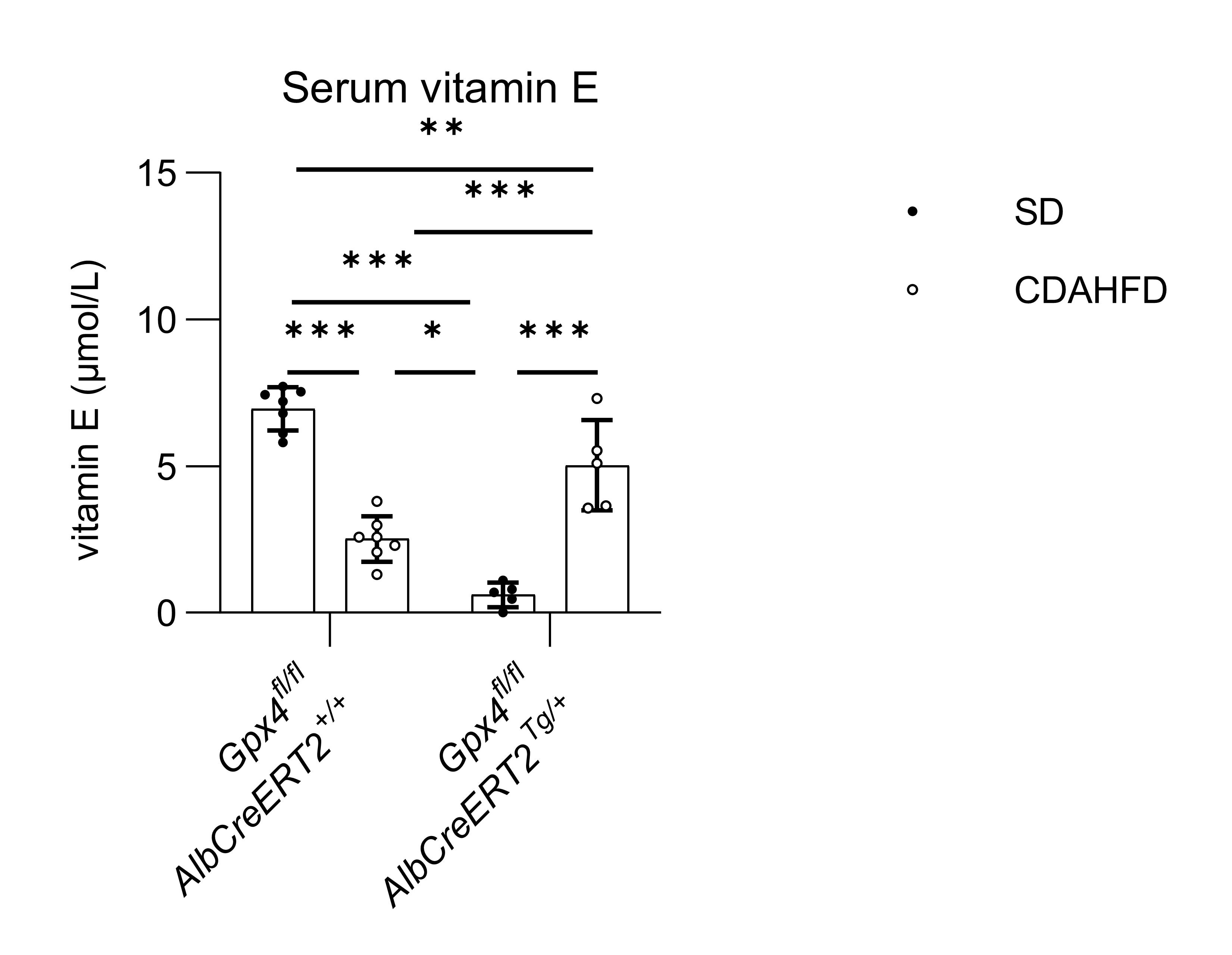


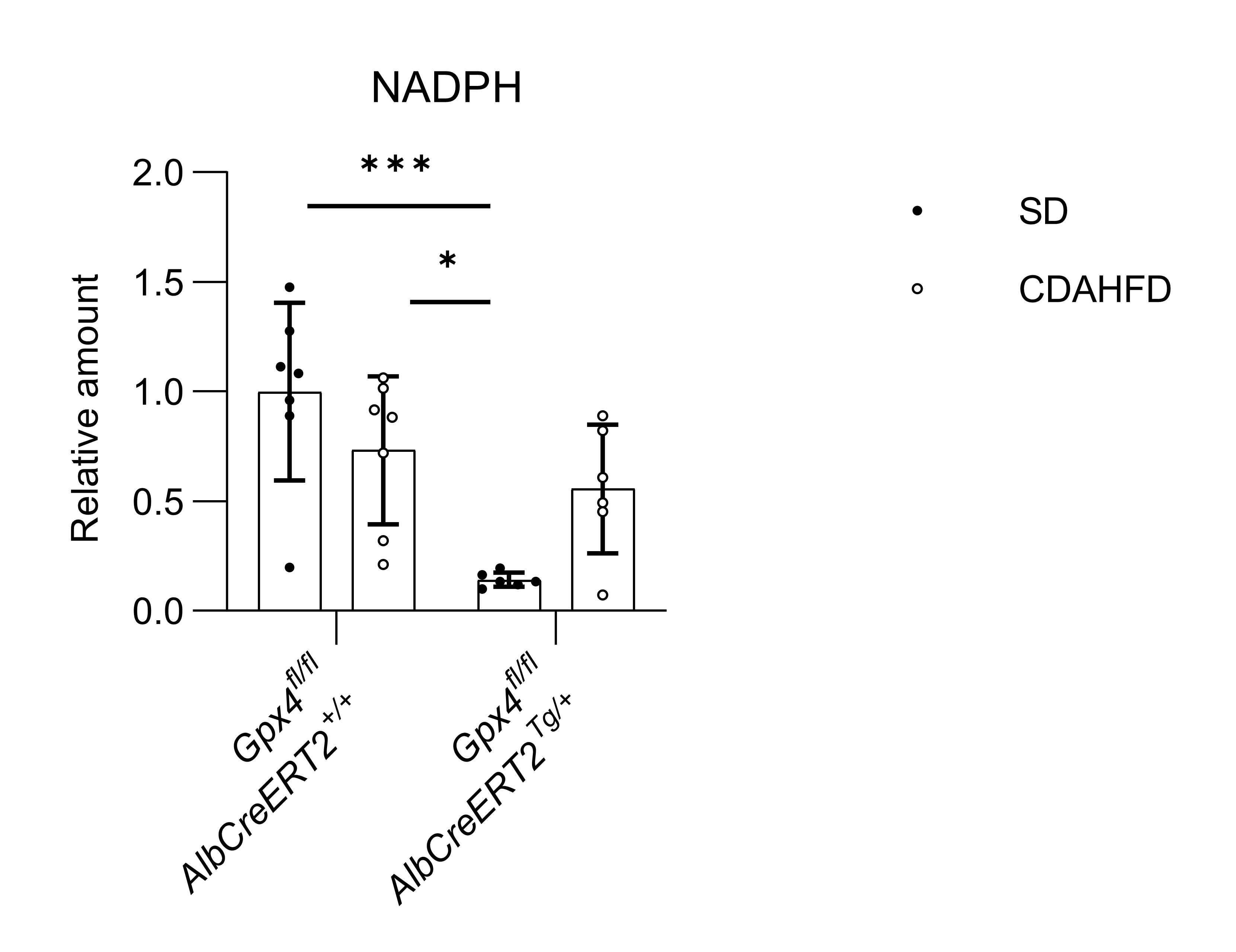

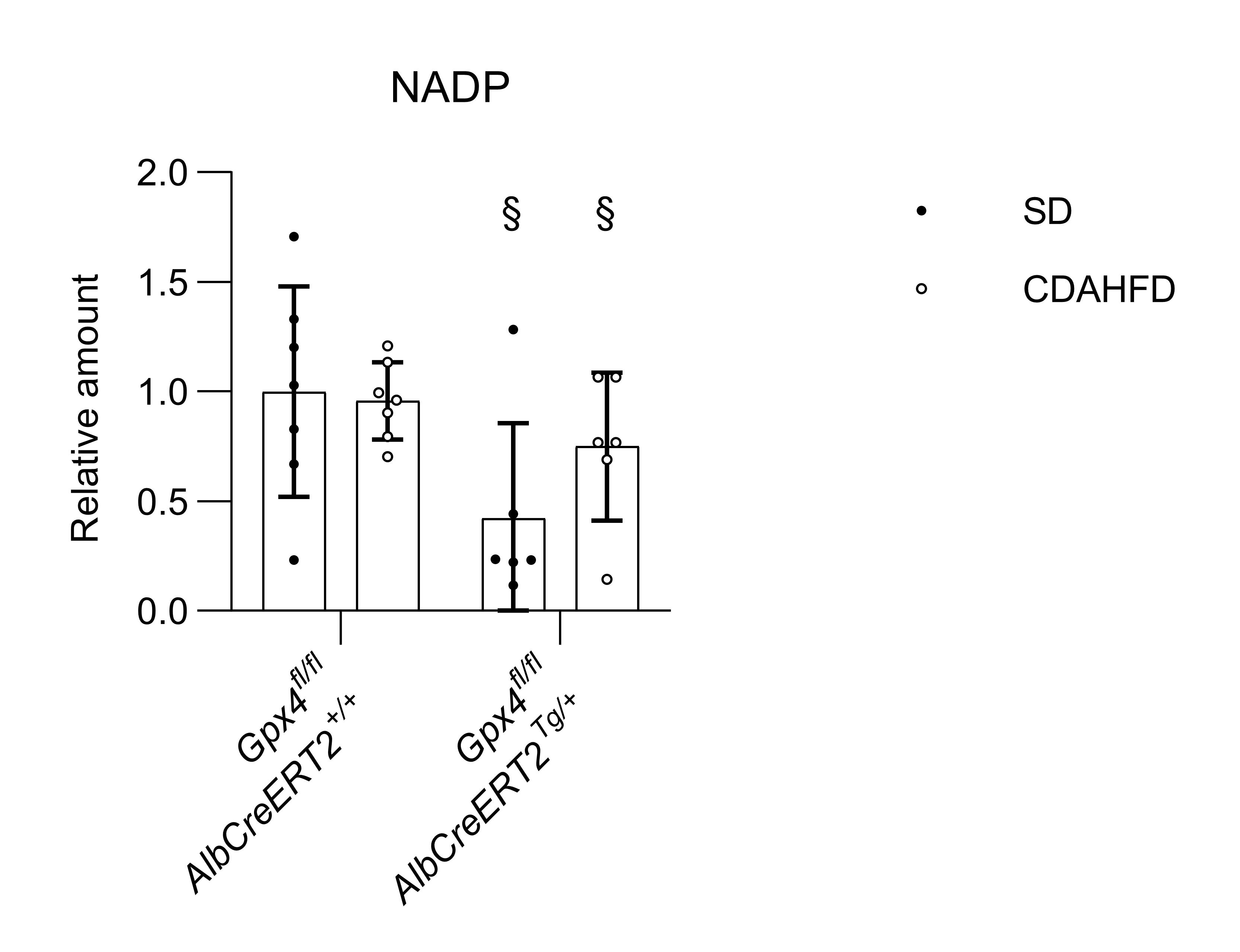

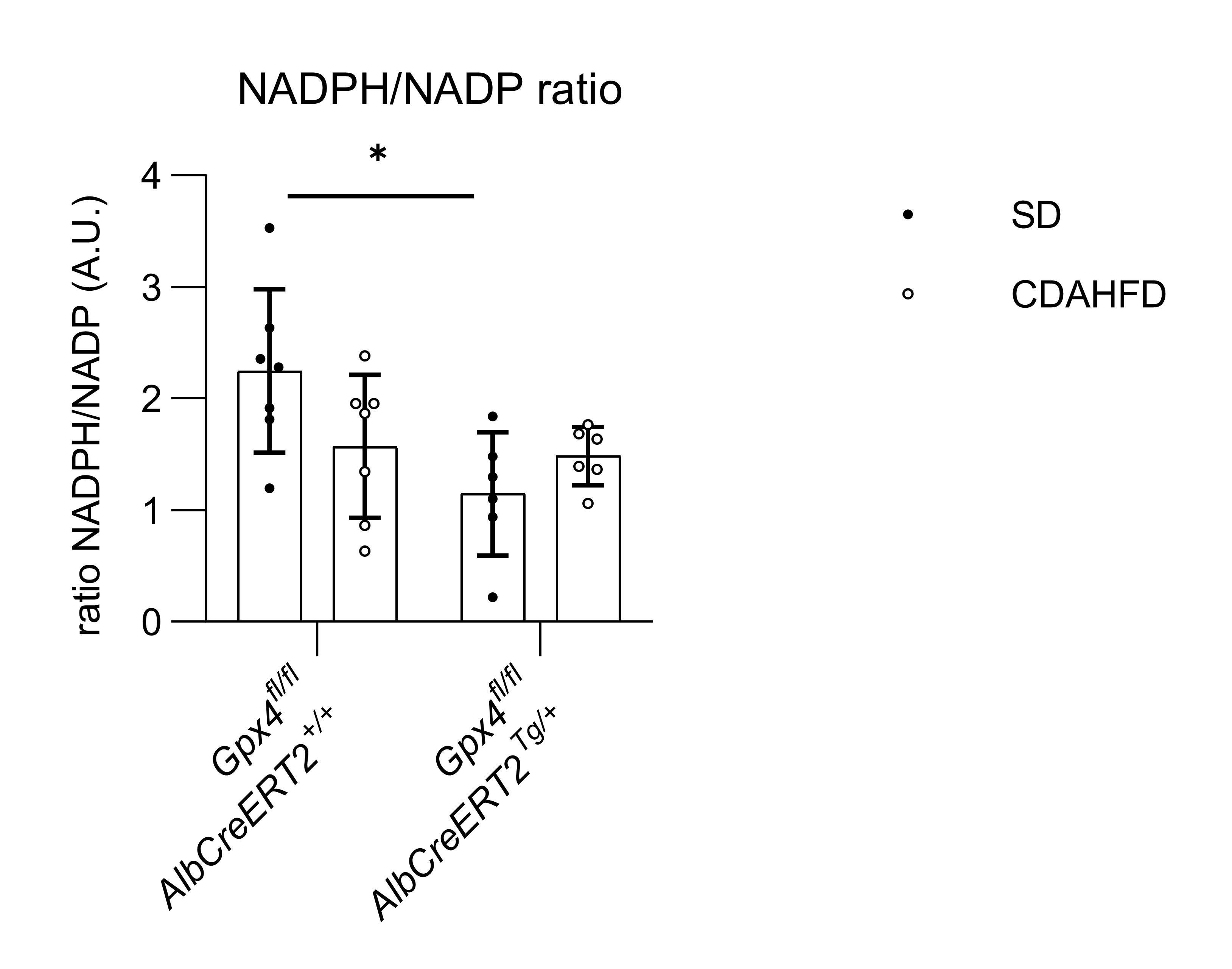


**Fig. S15. Metabolic changes in hepatocyte-specific knock out of glutathione peroxidase 4 in CDAHFD**

Tamoxifen-inducible hepatocyte-specific GPX4 knock out mice (*Gpx4^fl/fl^ AlbCreERT2^Tg/+^*) and their control littermates (*Gpx4^fl/fl^ AlbCreERT2^+/+^*) were fed the choline-deficient L-amino acid-defined high-fat diet (CDAHFD) for a maximum of 8 weeks. After 4 weeks on their respective diets, all animals received intraperitoneal injections with tamoxifen on 5 consecutive days. Animals were sacrificed upon significant morbidity with deterioration of the clinical disease score or at the termination of the experiment. (A) Hepatic mRNA expression of glutathione peroxidase 4 (*Gpx4*). (B) Liver-on-bodyweight ratio as a measure of hepatomegaly. (C) Hepatic mRNA expression of ferroptosis suppressor protein 1 (*Fsp1*) and GTP cyclohydrolase 1 (*Gch1*). (D) Serum vitamin E measured with mass spectrometry. (E) Relative abundancies of reduced glutathione (GSH), oxidized glutathione (GSSG), tetrahydrobiopterin (BH4), 7,8-dihydro-L-biopterin (BH2), reduced nicotinamide adenine dinucleotide (NADH), oxidized nicotinamide adenine dinucleotide (NAD), reduced nicotinamide adenine dinucleotide phosphate (NADPH), oxidized nicotinamide adenine dinucleotide phosphate (NADP) and their ratio (NADPH/NADP), as measured with mass spectrometry in liver tissue from the four experimental groups. Data are presented as mean ± SD (n = 6-7 per group). Two-way ANOVA with post-hoc test if appropriate. *p <0.05; **p <0.01; ***p <0.001; § p<0.05 for the factor genotype *Gpx4^fl/fl^ AlbCreERT2^Tg/+^* on *Gpx4^fl/fl^ AlbCreERT2^+/+^*.

Figure S16

A

D

C

B

E

SD + vehicle SD + UAMC-3203 CDAHFD + vehicle CDAHFD + UAMC-3203

H&E

TM

F

G

**Fig. S16. Effect of preventive ferroptosis inhibition on CDAHFD-induced MASH.**

The role of ferroptosis in MASH was explored using the lead ferroptosis inhibitor UAMC-3203 in the preclinical choline-deficient L-amino acid-defined high-fat diet (CDAHFD) model. (A) Experimental design of administration of UAMC-3203 (12.35mg/kg bodyweight) or 0.9% NaCl (vehicle) via once daily intraperitoneal (i.p.) injection (n = 10 per group) in animals fed CDAHFD or standard diet (SD) for 4 weeks. (B) Hepatic MDA levels in preventive ferroptosis inhibition. (C) Serum alanine aminotransferase (ALT) and aspartate aminotransferase (AST). (D) Liver-on-bodyweight ratio. (E) Representative images of H&E and Masson’s trichrome stains in four preventive experimental groups. Magnification 100x, scale bar 200µm. (F) Scoring of histologic features of steatosis, lobular inflammation, ballooning, NAFLD activity score and fibrosis in preventive ferroptosis inhibition. (G) Quantification of liver area enveloped by macrovesicular steatosis in preventive ferroptosis inhibition. Data presented as mean ± standard deviation. **p <0.01; ***p <.001; # p<0.05 for the factor diet CDAHFD versus SD. Two-way ANOVA with post-hoc test if appropriate. Kruskal-Wallis test with post-hoc testing for ordinal histologic scoring.

Figure S17

SD

HFHFD

A

B

**Fig. S17. Signature of ferroptosis breakdown products in HFHFD.**

Mice were fed the high-fat high-fructose diet (HFHFD) or standard diet (SD) for 20-24 weeks and ferroptosis breakdown products were measured (n = 4-6 per group). (A) Malondialdehyde (MDA) was measured in liver homogenates by means of the N-methyl-2-phenylindole assay. (B) After reconstruction of liver lobules in whole slide liver images, immunohistochemistry for 4-hydroxynonenal (4HNE) was quantified with regards to the relative distance within the lobule. Magnification 100x, scale bar 200µm.

Figure S18

A

B

C

SD+vehicle SD+UAMC-3203 HFHFD+vehicle HFHFD+UAMC-3203

D

F

E

H

I

G

**Fig. S18. Effects of therapeutic ferroptosis inhibition on serum glucose and lipid levels in HFHFD.**

Mice fed the high-fat high-fructose diet (HFHFD) for 24 weeks were randomized to receive treatment with 0.9% NaCl (vehicle) or ferroptosis inhibitor UAMC-3203 (UAMC-3203) via osmotic minipumps during the last 4 weeks on their respective diets. (A) Scoring of histologic features of ballooning and fibrosis. (B) Evolution of absolute bodyweight during total experiment. (C) Macroscopic photos of livers after sacrifice from 4 experimental groups. (D) Liver-on-bodyweight ratio as a measure of hepatomegaly. (E) Serum low-density lipoprotein (LDL) cholesterol. (F) Serum high-density lipoprotein (HDL) cholesterol. (G) Serum triglycerides. (H) Serum free fatty acids. (I) Serum glucose. Data are presented as mean ± SD (n = 9 per group). Data from two independent experiments were combined (n = 9 per group). Generalized estimating equations with correlation structure ‘autoregression 1’ to model longitudinal bodyweights. Analysis of covariance (ANCOVA) with adjustment for relative weight change during treatment and post hoc Tukey’s test if appropriate. # p <0.05 for the factor diet HFHFD versus SD. § p < 0.05 for the factor intervention (UAMC-3203 versus vehicle). ∗p <0.05; ∗∗p <0.01.

Figure S19

Pre ML162 24 hour post 1.25µM ML162

Control

OA+PA

AA

DHA

**Fig. S19. Visualization of cell death in HepG2 cells exposed to GPX4 inhibitor ML162.**

HepG2 cells exposed for 48 hours to control medium or MASH environment supplemented with oleic and palmitic acid (OA+PA), arachidonic acid (AA, ω-6 PUFA, 20:4) or docosahexaenoic acid (DHA, ω-3 PUFA, 22:6) were exposed to 1.25µM of GPX4 inhibitor ML162 for 24 hours. Images of SytoxGreen dye, an indicator of cell death, were superimposed on brightfield microscopy images. Image acquisition was performed before and 24 hours after addition of ML162. Magnification 100x, scale bar 500µm.

Figure S20

A

C

B

D

**Fig. S20. mRNA sequencing reveals altered expression of ferroptosis-related genes in HepG2 exposed to MASH environment with oleic and palmitic acid for 48 hours versus control.**

(A) Principal component analysis to cluster data from 12 samples from three independent experiments. (B) MA plot where adjusted p <0.05 was defined as significant. (C) Volcano plot where adjusted p <0.05 was defined as significant. (D) Heatmap of mRNA expression of 30 ferroptosis-related genes (scaled per row) in HepG2 cells treated with control or MASH environment supplemented with oleic and palmitic (OA+PA) acid. Hierarchical clustering was used to group samples and individual genes (n = 9 per group).

Figure S21

NES = -1.33 NES = -1.19

padj = 0.21 padj = 0.26

**Fig. S21. Gene set enrichment analysis for PUFA and iron gene sets in HepG2 exposed to MASH environment supplemented with oleic acid and palmitic acid versus control.**

Gene set enrichment analysis (GSEA) was used to interrogate possible overrepresentation of genes belonging to the ‘PUFA’ and ‘Iron’ gene sets in the transcriptome of HepG2 cells exposed to MASH environment supplemented with oleic and palmitic acid compared to control. NES: normalized expression score; padj: adjusted p-value.

Figure S22

**Fig. S22. The shares of saturated, monounsaturated and polyunsaturated fatty acids among the total pool of fatty acids in HepG2 cells.**

The percentage of saturated (SFA), monounsaturated (MUFA) and polyunsaturated fatty acids (PUFA) was calculated in HepG2 cells exposed to different media. Media comprised solvent control (Control) or MASH environment supplemented with oleic and palmitic acid (OA+PA), arachidonic acid (AA, ω-6 PUFA, 20:4) or docosahexaenoic acid (DHA, ω-3 PUFA, 22:6).

Figure S23

**Fig. S23. Distribution of fatty acids over different lipid classes in HepG2 cells treated with control or different fatty acids in MASH environment.**

The distribution (in percentage, %) of esterification of fatty acids per lipid class in HepG2 cells is represented in a pie chart per treatment condition, *i.e.* solvent control (Control) or MASH environment supplemented with oleic and palmitic acid (OA+PA), arachidonic acid (AA, ω-6 PUFA, 20:4) or docosahexaenoic acid (DHA, ω-3 PUFA, 22:6). The lipid classes under investigation are cholesterolesters (CE), ceramides (CER), diacylglycerol (DAG), phosphatidylcholine (PC), phosphatidylethanolamine (PE), phosphatidylglycerol (PG), phosphatidylinositol (PI), phospatidylserine (PS) and triacylglycerol (TAG).

Figure S24

**Fig. S24. Saturated, monounsaturated and polyunsaturated fatty acids in different lipid classes in HepG2 treated with control or various fatty acids in MASH environment.**

The percentage of fatty acids pertaining to saturated (SFA), monounsaturated (MUFA) and polyunsaturated (PUFA) fatty acids were plotted per lipid class (rows) in HepG2 cells treated with different media conditions. Media consisted of solvent control (Control) or MASH environment supplemented with oleic and palmitic acid (OA+PA), arachidonic acid (AA, ω-6 PUFA, 20:4) or docosahexaenoic acid (DHA, ω-3 PUFA, 22:6). The lipid classes under investigation are cholesterolesters (CE), ceramides (CER), diacylglycerol (DAG), phosphatidylcholine (PC), phosphatidylethanolamine (PE), phosphatidylglycerol (PG), phosphatidylinositol (PI), phospatidylserine (PS) and triacylglycerol (TAG).

Figure S25

**Fig. S25. Distribution of fatty acids species among different lipid classes in HepG2 cells treated with control or different fatty acids in MASH environment.**

The percentage of esterified fatty acids consisting of a certain fatty acid species (in adjacent columns) in different lipid classes (rows) is plotted per treatment condition. Indeed, HepG2 cells were treated with solvent control (Control) or MASH environment supplemented with oleic and palmitic acid (OA+PA), arachidonic acid (AA, ω-6 PUFA, 20:4) or docosahexaenoic acid (DHA, ω-3 PUFA, 22:6). The lipid classes under investigation are cholesterol esters (CE), ceramides (CER), diacylglycerol (DAG), phosphatidylcholine (PC), phosphatidylethanolamine (PE), phosphatidylglycerol (PG), phosphatidylinositol (PI), phosphatidylserine (PS) and triacylglycerol (TAG).

Figure S26

A

B

**Fig. S26. Analysis of polyunsaturated fatty acids esterified into phospholipids.**

(A) The percentage of phospholipids esterified with one or two polyunsaturated fatty acids (PUFA), i.e. PL-PUFA and PL-PUFA_2_, respectively, was plotted in HepG2 cells exposed to solvent control (Control) or MASH environment supplemented with oleic and palmitic acid (OA+PA), arachidonic acid (AA, ω-6 PUFA, 20:4) or docosahexaenoic acid (DHA, ω-3 PUFA, 22:6). This analysis was repeated for different classes of phospholipids, *i.e.* phosphatidylcholine (PC), phosphatidylethanolamine (PE), phospatidylserine (PS), phosphatidylinositol (PI). (B) Likewise, the percentage of ether phospholipids in the classes phosphatidylcholine (PC) and phosphatidylethanolamine (PE) esterified with one PUFA, *i.e.* PC-ePUFA and PE-ePUFA, respectively, is plotted in HepG2 cells exposed to the same conditions. Data are presented as mean ± SD (n = 3 per group). One-way ANOVA with post-hoc test if appropriate.

**Figure S27**

**Fig. S27. Full and uncropped western blots.**

**Supplementary Tables**

**Table S1. Patient cohort demographics**

| **Characteristics** | **Control (n = 19)** | **MASL (n = 21)** | **MASH F0-1**  **(n = 18)** | **MASH F2-3**  **(n = 18)** | **p-value** | **adjusted p-value** |
| --- | --- | --- | --- | --- | --- | --- |
| Age, year | 38 (27 - 46) | 50 (45 - 60) | 52 (39 - 58) | 54 (37.5 - 59) | 0.004** | 0.009** |
| Gender, male, n(%) | 4 (21.1) | 11 (52.4) | 11 (61.1) | 12 (66.7) | 0.026* | 0.04* |
| BMI, kg/m² | 36.1 (28.5 - 40.4) | 32.5 (29.4 - 39.5) | 30.3 (29.6 - 37.7) | 32.0 (26.7 - 35.3) | 0.656 | 0.656 |
| Type 2 diabetes mellitus, % | 3 (16.7) | 12 (57.1) | 7 (41.2) | 6 (33.3) | 0.076 | 0.106 |
| HOMA-IR | 2.9 (2.3 - 4.1) | 4.6 (4.0 - 6.0) | 5.5 (4.1 - 11.1) | 7.2 (5.4 - 13.1) | 4.04E-04*** | 0.001** |
| Total cholesterol, mg/dL | 189 (179 - 196) | 173 (135 - 212) | 180 (151 - 197) | 164 (136 - 203) | 0.219 | 0.256 |
| LDL cholesterol, mg/dL | 105 (96 - 121) | 102 (76 - 212) | 115 (98 - 127) | 107 (94 - 142) | 0.292 | 0.314 |
| HDL cholesterol, mg/dL | 61 (54 - 71) | 43 (38 - 61) | 40 (35 - 49) | 40.5 (34.3 - 49) | 5.05E-04*** | 0.001** |
| Ferritin, µg/L | 70 (25 - 70) | 80 (25 - 251) | 165 (122 - 232) | 173 (87 - 338) | 0.019* | 0.033* |
| Perls' stain, positive, n(%) | 0 (0) | 3 (14.3) | 0 (0) | 1 (5.6) | 0.167 | 0.213 |
| AST, U/L | 18 (15 - 22) | 25 (23 - 47) | 36 (28 - 44) | 43 (26.5 - 55) | 5.62600E-05*** | 3.938E-04** |
| ALT, U/L | 31 (25 - 38) | 38 (30 - 57) | 53 (47 - 76) | 51 (37.5 - 101) | 0.005** | 0.009** |
| Cytokeratin-18 M65, U/L | 136.3 (97.8 - 158.6) | 220.6 (185.8 - 327.9) | 242.8 (177.9 - 417.3) | 355.2 (241.8 - 735.6) | 3.51E-04*** | 0.001** |
| Cytokeratin-18 M30, U/L | 115.4 (93.0 - 133.4) | 184.0 (149.3 - 272.1) | 267.4 (174.8 - 359.2) | 401.7 (193.6 - 655.1) | 4.978E-07*** | 6.97E-06*** |

Data presented as median (interquartile range) unless otherwise specified. Kruskal-Wallis and Fisher’s exact tests were used to compare patient characteristics among different histologic groups. *P <0.05; **p <0.01; ***p <0.001. BMI: body mass index; HOMA-IR: homeostasis model assessment–insulin resistance; LDL: low density lipoprotein; HDL: high-density lipoprotein; AST: aspartate aminotransferase; ALT: alanine aminotransferase.

**Table S2. Ferroptosis-related genes incorporated into 4 gene sets for gene set enrichment/variation analysis.**

| **Ferroptosis defenses** | | **GSH** | | **PUFA** | | **Iron** |  |
| --- | --- | --- | --- | --- | --- | --- | --- |
| **Gene symbol** | **Gene name** | **Gene symbol** | **Gene name** | **Gene symbol** | **Gene name** | **Gene symbol** | **Gene name** |
| *GPX4* | glutathione peroxidase 4 | *SLC7A11* | solute carrier family 7 member 11 | *ACSL4* | acyl-CoA synthetase long chain family member 4 | *TFRC* | transferrin receptor |
| *AIFM2* | apoptosis-inducing factor 2 | *TXNRD1* | thioredoxin reductase 1 | *LPCAT3* | lysophosphatidylcholine acyltransferase 3 | *NCOA4* | nuclear receptor coactivator 4 |
| *DHODH* | dihydroorotate dehydrogenase | *GCLC* | glutamate-cysteine ligase catalytic subunit | *ELOVL2* | ELOVL fatty acid elongase 2 | *STEAP3* | STEAP3 metalloreductase |
| *DHFR* | dihydrofolate reductase | *GCLM* | glutamate-cysteine ligase modifier subunit | *ELOVL5* | ELOVL fatty acid elongase 5 | *SLC11A2* | solute carrier family 11 member 2 |
| *GCH1* | GTP cyclohydrolase 1 | *GSS* | glutathione synthetase | *FADS2* | fatty acid desaturase 2 | *HAMP* | hepcidin antimicrobial peptide |
| *HMOX1* | heme oxygenase 1 | *GSR* | glutathione-disulfide reductase | *FADS1* | fatty acid desaturase 1 | *ALOX15* | arachidonate 15-lipoxygenase |
| *KEAP1* | kelch-like ECH-associated protein 1 | *CBS* | cystathionine beta-synthase |  |  | *PEBP1* | phosphatidylethanolamine binding protein 1 |
| *NFE2L2* | nuclear factor erythroid 2-related factor 2 | *CTH* | cystathionine gamma-lyase |  |  |  |  |
| *AKR1C1* | aldo-keto reductase family 1 member C1 |  |  |  |  |  |  |

**Table S3. Pharmacokinetic properties of Fer-analogue UAMC-3203 in mice.**

| **Route** | **Matrix** | **t½ (hr)** | **C_0_ (ng/mL)** | **AUC_0-last_ (hr*ng/mL )** |
| --- | --- | --- | --- | --- |
| IV | Plasma | 0.51 | 5905 | 3670 |
|  |  |  |  |  |
|  | Tissue sampling time (h) | 0.5h  (ng/mL or ng/g) | 4h  (ng/mL or ng/g) | 24h  (ng/mL or ng/g) |
|  | Liver | 245 | 2643 | 21133 |
|  | Lung | 146 | 773 | 4503 |
|  | Heart | BLQ | 764 | 4633 |
|  | Kidney | 250 | 9670 | 40800 |
|  | Brain | BLQ | 189 | 361 |
|  | Plasma | 1270 | 190 | 10.3 |

Concentration of UAMC-3203 in plasma and tissue samples at time 0 (C_0_), 0.5, 4 and 24 hours, its elimination half-life (t^1/2^) and its total area under the curve (AUC_0-last_) following intravenous (IV) dosing at 10 mg/kg.

**Table S4. Differential expression analysis of ferroptosis-related genes in HepG2 cells treated with MASH environment supplemented with oleic acid and palmitic acid versus control.**

| **Gene symbol** | **Gene name** | **log2FoldChange** | **Fold Change** | **lfcSE** | **pvalue** | **padj** |
| --- | --- | --- | --- | --- | --- | --- |
| **Ferroptosis defences** | | | | | | |
| *GPX4* | glutathione peroxidase 4 | -0.2444355 | 0.84414605 | 0.08553097 | 0.0042651 | 0.00666045 |
| ***AIFM2*** | apoptosis-inducing factor 2 | 0.6018551 | **1.51766682** | 0.1399513 | 1.7044E-05 | **3.4166E-05** |
| *DHODH* | dihydroorotate dehydrogenase | -1.0505674 | 0.48277825 | 0.1253546 | 5.2585E-17 | 2.5022E-16 |
| *DHFR* | dihydrofolate reductase | -1.8133561 | 0.28452827 | 0.09284436 | 5.9693E-85 | 4.9531E-83 |
| *GCH1* | GTP cyclohydrolase 1 | -0.5549796 | 0.68066666 | 0.09655537 | 9.0419E-09 | 2.4049E-08 |
| ***HMOX1*** | heme oxygenase 1 | -1.6783082 | **0.31244882** | 0.18856772 | 5.5699E-19 | **2.9901E-18** |
| ***KEAP1*** | kelch-like ECH-associated protein 1 | -0.6993717 | **0.61584033** | 0.05974292 | 1.1826E-31 | **1.22E-30** |
| *NFE2L2* | nuclear factor erythroid 2-related factor 2 | -1.0700574 | 0.47630006 | 0.07638918 | 1.3933E-44 | 2.6484E-43 |
| ***AKR1C1*** | aldo-keto reductase family 1 member C1 | -2.4384443 | **0.18448248** | 0.39302174 | 5.4924E-10 | **1.6151E-09** |
| **GSH** | | | | | | |
| *SLC7A11* | solute carrier family 7 member 11 | 0.00517781 | 1.00359543 | 0.10432092 | 0.9604145 | 0.96708249 |
| *TXNRD1* | thioredoxin reductase 1 | -0.4867512 | 0.71363032 | 0.05115701 | 1.8198E-21 | 1.1193E-20 |
| ***GCLC*** | glutamate-cysteine ligase catalytic subunit | -0.880368 | **0.54322886** | 0.13111752 | 1.8892E-11 | **6.2069E-11** |
| *GCLM* | glutamate-cysteine ligase modifier subunit | -0.4806921 | 0.71663376 | 0.08496978 | 1.5385E-08 | 4.0074E-08 |
| *GSS* | glutathione synthetase | -0.0367706 | 0.97483464 | 0.04682881 | 0.43232886 | 0.48139121 |
| ***GSR*** | glutathione-disulfide reductase | -1.0156954 | **0.49458985** | 0.07432324 | 1.6224E-42 | **2.7917E-41** |
| *CBS* | cystathionine beta-synthase | -0.2287379 | 0.85338111 | 0.1223083 | 0.06145951 | 0.08084214 |
| ***CTH*** | cystathionine gamma-lyase | -0.64753 | **0.6383723** | 0.1208242 | 8.3557E-08 | **2.049E-07** |
| **PUFA** | | | | | | |
| *ACSL4* | acyl-CoA synthetase long chain family member 4 | -0.5553851 | 0.6804754 | 0.08412723 | 4.0639E-11 | 1.3037E-10 |
| *LPCAT3* | lysophosphatidylcholine acyltransferase 3 | -0.4180375 | 0.74844206 | 0.08517595 | 9.2043E-07 | 2.0801E-06 |
| ***ELOVL2*** | ELOVL fatty acid elongase 2 | -1.1113645 | **0.46285605** | 0.14146036 | 3.9544E-15 | **1.6699E-14** |
| *ELOVL5* | ELOVL fatty acid elongase 5 | 0.31081191 | 1.24040557 | 0.06762277 | 4.3014E-06 | 9.1067E-06 |
| *FADS2* | fatty acid desaturase 2 | 0.04824845 | 1.03400879 | 0.14483862 | 0.73904472 | 0.77318135 |
| ***FADS1*** | fatty acid desaturase 1 | -0.8068575 | **0.57162562** | 0.08962746 | 2.2094E-19 | **1.2206E-18** |
| **Iron** | | | | | | |
| *TFRC* | transferrin receptor | -0.1260579 | 0.91633185 | 0.07213293 | 0.0805371 | 0.10359915 |
| ***NCOA4*** | nuclear receptor coactivator 4 | -0.9205331 | **0.52831378** | 0.12020274 | 1.886E-14 | **7.6071E-14** |
| *STEAP3* | STEAP3 metalloreductase | -0.579864 | 0.66902683 | 0.07357949 | 3.2533E-15 | 1.3806E-14 |
| ***SLC11A2*** | solute carrier family 11 member 2 | 0.71867197 | **1.64566647** | 0.07918074 | 1.1228E-19 | **6.2875E-19** |
| ***HAMP*** | hepcidin antimicrobial peptide | -3.6957062 | **0.07717588** | 0.53229691 | 3.8402E-12 | **1.3174E-11** |
| ***PEBP1*** | phosphatidylethanolamine binding protein 1 | -0.8640898 | **0.54939291** | 0.13206887 | 6.0409E-11 | **1.9166E-10** |

Differential expression of ferroptosis-related genes pertaining to 4 genesets between HepG2 treated with MASH environment and on those treated with control medium. Highlighted in bold are genes with foldchange │1.5│and adjusted p-value <0.05.

LfcSE, standard error of log fold change; Padj, adjusted p-value.

**Supplementary references**

1 American Diabetes Association. Classification and Diagnosis of Diabetes : Standards of Medical Care in Diabetes — 2022. *Diabetes Care* 2022; **45**: S17–S38.

2 Matthews DR, Hosker JP, Rudenski AS, Naylor BA, Treacher DF, Turner RC. Homeostasis model assessment: insulin resistance and β-cell function from fasting plasma glucose and insulin concentrations in man. *Diabetologia* 1985; **28**: 412–419.

3 Van Eyck A, Kwanten WJ, Peleman C, Makhout S, Van Laere S, Van De Maele K *et al.* The role of adipose tissue and subsequent liver tissue hypoxia in obesity and early stage metabolic dysfunction associated steatotic liver disease. *Int J Obes* 2024; **48**: 512–522.

4 Van Herck MA, Vonghia L, Kwanten WJ, Julé Y, Vanwolleghem T, Ebo DG *et al.* Diet reversal and immune modulation show key role for liver and adipose tissue T cells in murine nonalcoholic steatohepatitis. *Cmgh* 2020; **10**: 467–490.

5 Van Coillie S, Van San E, Goetschalckx I, Wiernicki B, Mukhopadhyay B, Tonnus W *et al.* Targeting ferroptosis protects against experimental (multi)organ dysfunction and death. *Nat Commun* 2022; **13**: 1–14.

6 Van San E, Debruyne AC, Veeckmans G, Tyurina YY, Tyurin VA, Zheng H *et al.* Ferroptosis contributes to multiple sclerosis and its pharmacological targeting suppresses experimental disease progression. *Cell Death Differ* 2023; **30**: 2092–2103.

7 Kleiner DE, Brunt EM, Van Natta M, Behling C, Contos MJ, Cummings OW *et al.* Design and validation of a histological scoring system for nonalcoholic fatty liver disease. *Hepatology* 2005; **41**: 1313–1321.

8 Chalasani N, Younossi Z, Lavine JE, Diehl AM, Brunt EM, Cusi K *et al.* The diagnosis and management of non-alcoholic fatty liver disease: Practice guideline by the American Association for the Study of Liver Diseases, American College of Gastroenterology, and the American Gastroenterological Association. *Am J Gastroenterol* 2012; **107**: 811–826.

9 Munsterman ID, van Erp M, Weijers G, Bronkhorst C, de Korte CL, Drenth JPH *et al.* A Novel Automatic Digital Algorithm that Accurately Quantifies Steatosis in NAFLD on Histopathological Whole-Slide Images. *Cytom Part B - Clin Cytom* 2019; **96**: 521–528.

10 Schindelin J, Arganda-Carreras I, Frise E, Kaynig V, Longair M, Pietzsch T *et al.* Fiji: An open-source platform for biological-image analysis. *Nat Methods* 2012; **9**: 676–682.

11 Hametner S, Wimmer I, Haider L, Pfeifenbring S, Brück W, Lassmann H. Iron and neurodegeneration in the multiple sclerosis brain. *Ann Neurol* 2013; **74**: 848–861.

12 Toyokuni S, Miyake N, Hiai H, Hagiwara M, Kawakishi S, Osawa T *et al.* The monoclonal antibody specific for the 4-hydroxy-2-nonenal histidine adduct. *FEBS Lett* 1995; **359**: 189–191.

13 Zheng H, Jiang L, Tsuduki T, Conrad M, Toyokuni S. Embryonal erythropoiesis and aging exploit ferroptosis. *Redox Biol* 2021; **48**: 102175.

14 Schutt F, Bergmann M, Holz FG, Kopitz J. Proteins modified by malondialdehyde, 4-hydroxynonenal, or advanced glycation end products in lipofuscin of human retinal pigment epithelium. *Investig Ophthalmol Vis Sci* 2003; **44**: 3663–3668.

15 Bankhead P, Loughrey MB, Fernández JA, Dombrowski Y, McArt DG, Dunne PD *et al.* QuPath: Open source software for digital pathology image analysis. *Sci Rep* 2017; **7**: 1–7.

16 Schmidt U, Weigert M, Coleman B, Myers G. Cell Detection with Star-convex Polygons. In: *International Conference on Medical Image Computing and Computer-Assisted Intervention (MICCAI)*. Granada, Spain, 2018 doi:10.1007/978-3-030-00934-2_30.

17 R Core Team. R: A Language and Environment for Statistical Computinng. R Found. Stat. Comput. Vienna. 2018.https://www.r-project.org.

18 Peleman C, De Vos WH, Pintelon I, Driessen A, Van Eyck A, Van Steenkiste C *et al.* Zonated quantification of immunohistochemistry in normal and steatotic livers. *Virchows Arch* 2023; **482**: 1035–1045.

19 Schwen LO, Homeyer A, Schwier M, Dahmen U, Dirsch O, Schenk A *et al.* Zonated quantification of steatosis in an entire mouse liver. *Comput Biol Med* 2016; **73**: 108–118.

20 Erdelmeier I, Gérard-Monnier D, Régnard K, Moze-Henry N, Yadan JC, Chaudière J. Reactions of 1-methyl-2-phenylindole with malondialdehyde and 4- hydroxyalkenals. Analytical applications to a colorimetric assay of lipid peroxidation. *Chem Res Toxicol* 1998; **11**: 1176–1183.

21 Boeckmans J, Natale A, Rombaut M, Buyl K, Cami B. Human hepatic in vitro models reveal distinct anti-NASH potencies of PPAR agonists. *Cell Biol Toxicol* 2021; **37**: 293–311.

22 Grootjans S, Hassannia B, Delrue I, Goossens V, Wiernicki B, Dondelinger Y *et al.* A real-time fluorometric method for the simultaneous detection of cell death type and rate. *Nat Protoc* 2016; **11**: 1444–1454.

23 Magtanong L, Ko P, To M, Nomura DK, Olzmann JA, Dixon SJ *et al.* Exogenous Monounsaturated Fatty Acids Promote a Ferroptosis-Resistant Cell State. *Cell Chem Biol* 2019; **26**: 420–432.

24 Talebi A, de Laat V, Spotbeen X, Dehairs J, Rambow F, Rogiers A *et al.* Pharmacological induction of membrane lipid poly-unsaturation sensitizes melanoma to ROS inducers and overcomes acquired resistance to targeted therapy. *J Exp Clin Cancer Res* 2023; **42**: 1–16.

25 Szepannek G. ClustMixType: User-friendly clustering of mixed-type data in R. *R J* 2019; **10**: 200–208.

26 Edgar R, Domrachev M, Lash AE. Gene Expression Omnibus: NCBI gene expression and hybridization array data repository. *Nucleic Acids Res* 2002; **30**: 207–210.

27 Govaere O, Cockell S, Tiniakos D, Queen R, Younes R, Vacca M *et al.* Transcriptomic profiling across the nonalcoholic fatty liver disease spectrum reveals gene signatures for steatohepatitis and fibrosis. *Sci Transl Med* 2020; **12**: 1–18.

28 Hoang SA, Oseini A, Feaver RE, Cole BK, Asgharpour A, Vincent R *et al.* Gene Expression Predicts Histological Severity and Reveals Distinct Molecular Profiles of Nonalcoholic Fatty Liver Disease. *Sci Rep* 2019; **9**: 1–14.

29 Suppli MP, Rigbolt KTG, Veidal SS, Heebøll S, Eriksen PL, Demant M *et al.* Hepatic transcriptome signatures in patients with varying degrees of nonalcoholic fatty liver disease compared with healthy normal-weight individuals. *Am J Physiol - Gastrointest Liver Physiol* 2019; **316**: 462–472.

30 Leek JT. Svaseq: Removing batch effects and other unwanted noise from sequencing data. *Nucleic Acids Res* 2014; **42**: 1–9.

31 Hänzelmann S, Castelo R, Guinney J. GSVA: Gene set variation analysis for microarray and RNA-Seq data. *BMC Bioinformatics* 2013; **14**: 1–15.

32 Scrucca L, Fop M, Murphy BT, Raftery AE. mclust 5: Clustering, Classification and Density Estimation Using Gaussian Finite Mixture Models. *R J* 2016; **8**: 289–317.

33 Neath AA, Cavanaugh JE. The Bayesian information criterion: Background, derivation, and applications. *WIREs Comput Stat* 2012; **4**: 199–203.

34 Biernacki C, Celeux G, Govaert G. Assessing a Mixture Model for Clustering with the Integrated Completed Likelihood. *IEEE Trans Pattern Anal Mach Intell* 2000; **22**: 719–725.

35 Davies DL, Bouldin DW. A Cluster Separation Measure. *IEEE Trans Pattern Anal Mach Intell* 1979; : 224–227.

36 Rosenbaum JT, Choi D, Harrington CA, Wilson DJ, Grossniklaus HE, Sibley CH *et al.* Gene expression profiling and heterogeneity of nonspecific orbital inflammation affecting the lacrimal gland. *JAMA Ophthalmol* 2017; **135**: 1156–1162.

37 Strigo IA, Simmons AN, Giebler J, Schilling JM, Moeller-Bertram T. Unsupervised learning for prognostic validity in patients with chronic pain in transdisciplinary pain care. *Sci Rep* 2023; **13**: 7581.

38 Bornschein J, Wernisch L, Secrier M, Miremadi A, Perner J, MacRae S *et al.* Transcriptomic profiling reveals three molecular phenotypes of adenocarcinoma at the gastroesophageal junction. *Int J Cancer* 2019; **145**: 3389–3401.
