# Supplementary figures and images for: Ferroptosis is a targetable detrimental factor in metabolic dysfunction-associated steatotic liver disease
Source: Cell Death Differ. 2024 Jul 26;31(9):1113–26. doi: 10.1038/s41418-024-01348-9 (PMC11369286; doi:10.1038/s41418-024-01348-9)

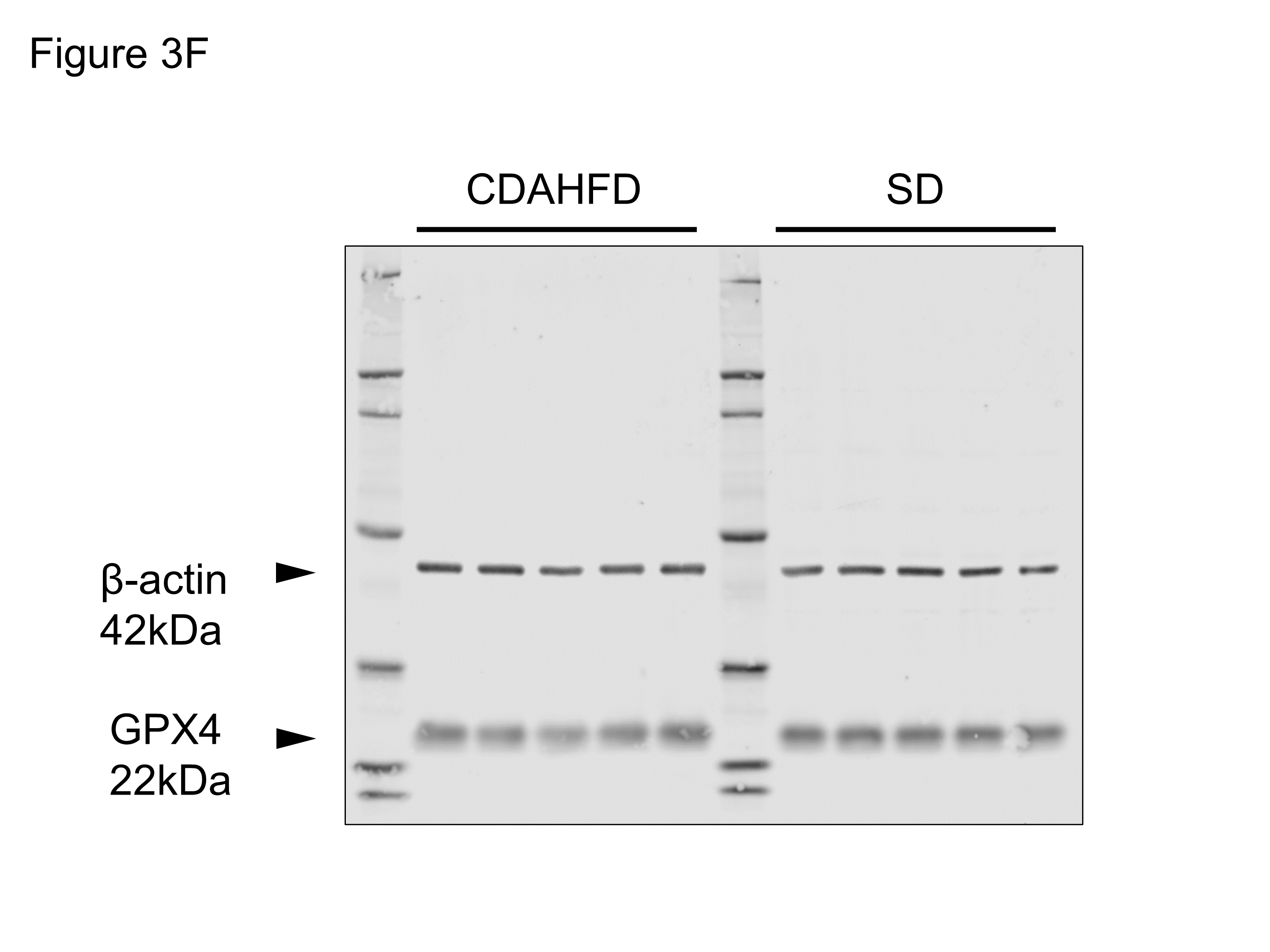

Supplement: Supplementary file 2 — Original western blot 3F [file 41418_2024_1348_MOESM2_ESM.png]

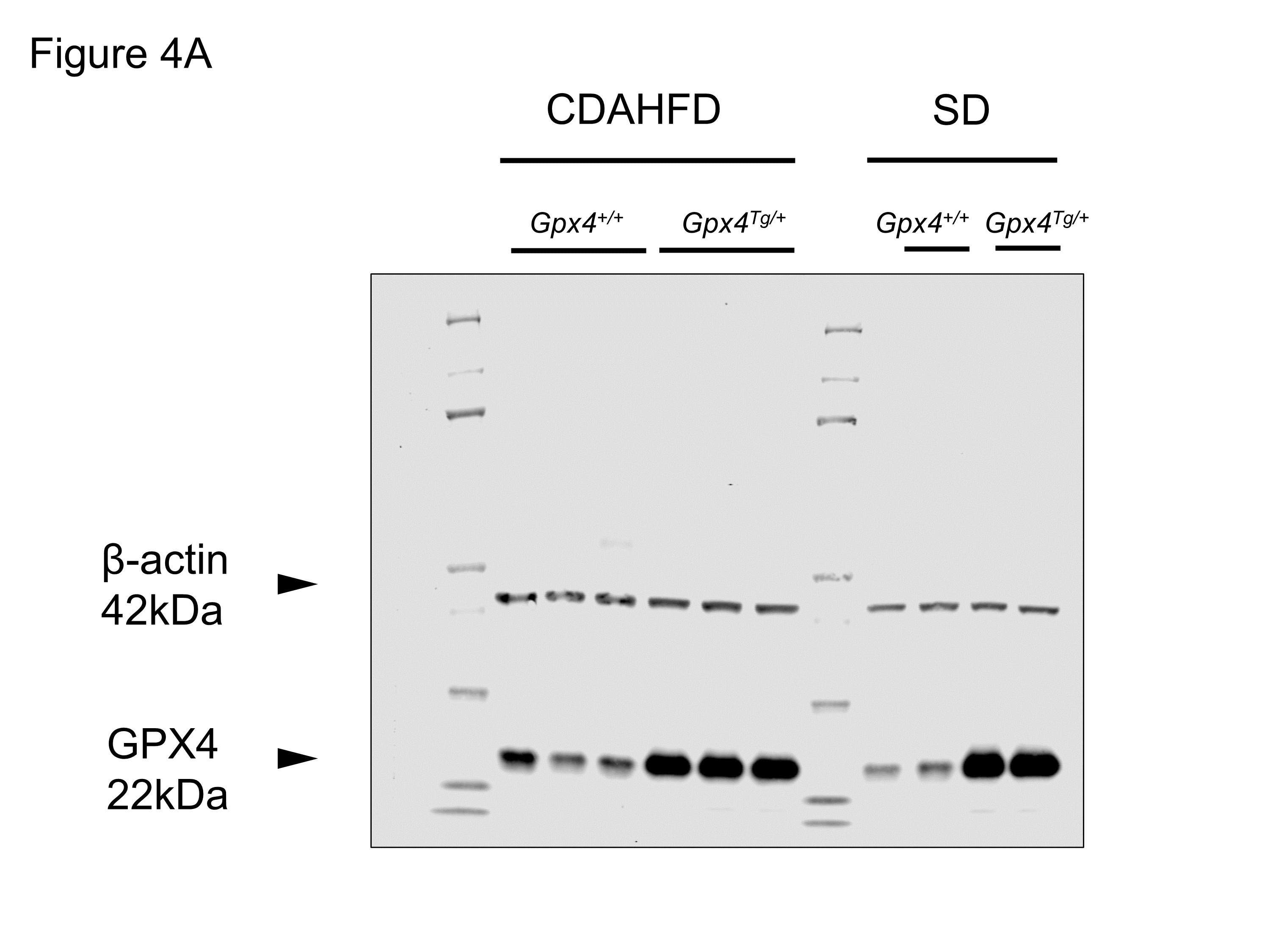

Supplement: Supplementary file 3 — Original western blot 4A [file 41418_2024_1348_MOESM3_ESM.png]

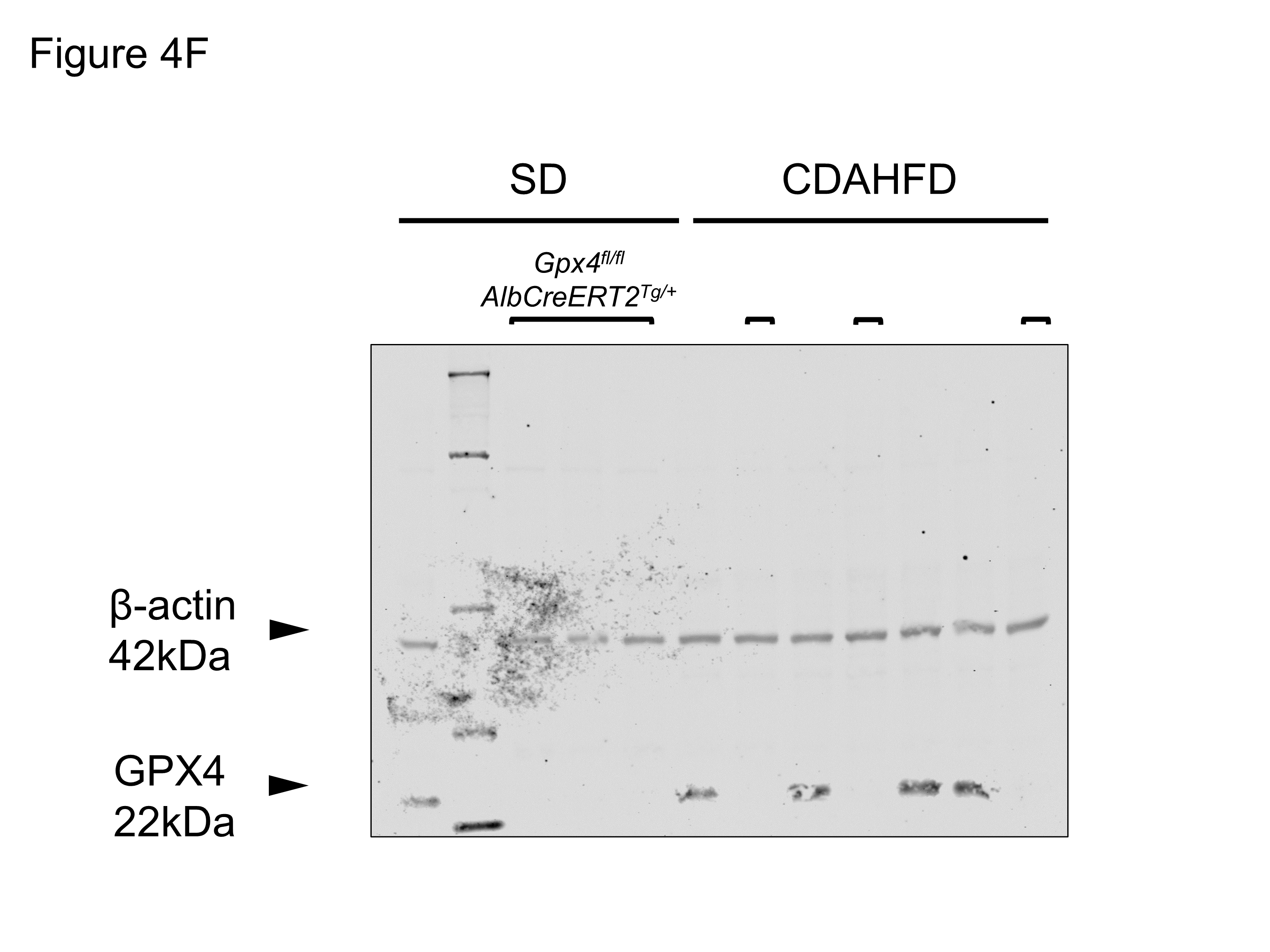

Supplement: Supplementary file 4 — Original western blot 4F [file 41418_2024_1348_MOESM4_ESM.png]
